# General and selective synthesis of primary amines using Ni-based homogeneous catalysts†

**DOI:** 10.1039/d0sc01084g

**Published:** 2020-03-25

**Authors:** Kathiravan Murugesan, Zhihong Wei, Vishwas G. Chandrashekhar, Haijun Jiao, Matthias Beller, Rajenahally V. Jagadeesh

**Affiliations:** Leibniz-Institut für Katalyse e. V. Albert Einstein-Str. 29a 18059 Rostock Germany haijun.jiao@catalysis.de matthias.beller@catalysis.de jagadeesh.rajenahally@catalysis.de

## Abstract

The development of base metal catalysts for industrially relevant amination and hydrogenation reactions by applying abundant and atom economical reagents continues to be important for the cost-effective and sustainable synthesis of amines which represent highly essential chemicals. In particular, the synthesis of primary amines is of central importance because these compounds serve as key precursors and central intermediates to produce value-added fine and bulk chemicals as well as pharmaceuticals, agrochemicals and materials. Here we report a Ni-triphos complex as the first Ni-based homogeneous catalyst for both reductive amination of carbonyl compounds with ammonia and hydrogenation of nitroarenes to prepare all kinds of primary amines. Remarkably, this Ni-complex enabled the synthesis of functionalized and structurally diverse benzylic, heterocyclic and aliphatic linear and branched primary amines as well as aromatic primary amines starting from inexpensive and easily accessible carbonyl compounds (aldehydes and ketones) and nitroarenes using ammonia and molecular hydrogen. This Ni-catalyzed reductive amination methodology has been applied for the amination of more complex pharmaceuticals and steroid derivatives. Detailed DFT computations have been performed for the Ni-triphos based reductive amination reaction, and they revealed that the overall reaction has an inner-sphere mechanism with H_2_ metathesis as the rate-determining step.

## Abstract 2

A Ni-triphos based homogeneous catalyst enabled the synthesis of all kinds of primary amines by reductive amination of carbonyl compounds with ammonia and hydrogenation of nitroarenes.

## Graphical abstract

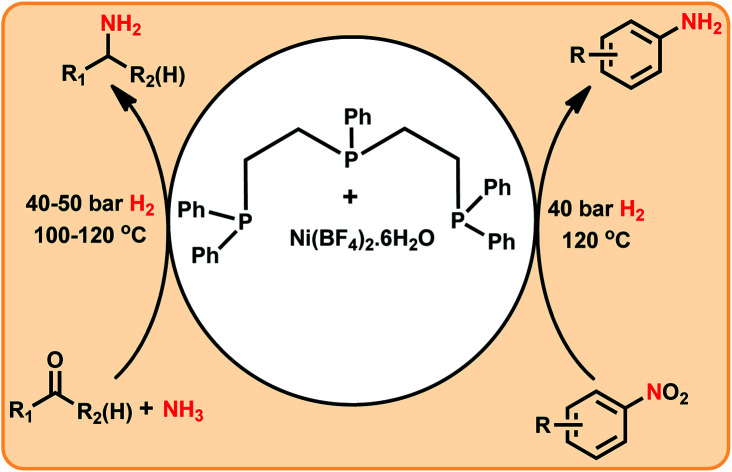

## Introduction

Catalytic reductive aminations and hydrogenations constitute essential processes widely applied in research laboratories and industries for the synthesis of fine and bulk chemicals as well as molecules used in life sciences.^1^ In particular, the selective and efficient synthesis of amines by applying these processes starting from inexpensive and easily available starting materials, and green and abundant reagents using non-noble metal-based catalysts continues to be an important goal of chemical research. In general, amines are highly essential fine and bulk chemicals used in chemistry, medicine, biology and materials.^2^ Noteworthily, amine functionalities constitute integral parts of a large number of life science molecules and play significant roles in their activities.^2^ As an example, more than 75% of 200 top selling drugs of the year 2018 contain amine/nitrogen moieties.^2*e*^ Among different kinds of amines, primary amines are highly valued because these compounds serve as key precursor and central intermediates for the synthesis of advanced chemicals, pharmaceuticals, agrochemicals and materials. The reductive amination of carbonyl compounds with ammonia^3^ and the hydrogenation of nitroarenes^4^ are found to be more expedient processes to synthesize benzylic, aliphatic and aromatic primary amines.^3,4^ Notably for the advancement of more sustainable and cost-effective synthesis of this class of amines, the development of base metal catalysts is highly desired and continues to attract scientific interest. Catalytic reductive aminations, especially for the synthesis of primary amines are challenging processes and are often non-selective and suffer from side reactions such as over alkylation and reduction to the corresponding alcohols.^3^ Hence in order to perform these reactions in an efficient and highly selective manner the development of suitable catalysts is of central importance. Regarding potential catalysts for reductive amination to prepare primary amines from carbonyl compounds using ammonia and molecular hydrogen, mainly heterogeneous catalysts based on Rh,^3*k*,*s*^ Ru,^3*k*–*m*,*q*,*r*^ Ni^3*d*,*h*,*n*^ and Co^3*a*^ are applied. Compared to that of heterogeneous materials, the design of homogeneous catalysts for this reaction is highly challenging due to the inactivation of metal complexes in the presence of ammonia by forming Werner-amine type complexes and common problems such as formation of secondary and/or tertiary amines and over hydrogenation of carbonyl compounds to corresponding alcohols associated with reductive aminations.^3^ Nevertheless, in recent years, a few Rh,^3*i*^ Ru,^3*c*,*f*,*g*,*j*^ Ir^3*p*^ and Co^3*e*^-based complexes have been established to catalyze the synthesis of primary amines from carbonyl compounds and ammonia. Despite these achievements, still the development of base metal homogeneous catalysts for the synthesis of benzylic and aliphatic primary amines is highly desired and attracts scientific interest.

The catalytic hydrogenation of nitroarenes represents an indispensable and widely applied process for the synthesis of aromatic amines (anilines).^1*c*,*d*,4^ In general this reaction mainly relies on heterogeneous catalysts. Unfortunately, homogeneous catalysts for the hydrogenation of nitro compounds are scarcely explored and using them remains a challenge.^4*c*–*f*^ Hence the development of suitable homogeneous catalysts, especially based on non-noble metals, for the synthesis of functionalized anilines continues to be important is also of significant interest. Here, we report a Ni-based complex for both reductive amination of carbonyl compounds with ammonia and hydrogenation of nitroarenes for the general synthesis of all kinds of primary amines.

Ni-based complexes are well known to catalyze a number of synthetic processes^5–11^ including hydrogenations,^6^ CH-activations,^7^ coupling reactions^8^ and amination of alcohols,^8,9^ as well as polymerizations^10^ and photo-redox reactions.^11^ The key to success for this Ni-catalysis in organic synthesis is the use of a broad variety of complexes based on specific ligands.^5–11^ Although Ni-based homogeneous catalysts are well recognized for a variety of reactions, they are still underdeveloped for reductive aminations as well as for the hydrogenation of nitro compounds.^5–11^ To the best of our knowledge until now there has been no homogeneous Ni-based catalyst known for both of these reactions to synthesize primary amines. Certainly, there is potential interest in the development of homogeneous Ni-based reductive amination and hydrogenation catalysts for the general and selective synthesis of primary amines. In addition to synthetic applications, it is also important to know the mode of action of Ni-complexes, formation of catalytically active species, and reactivity and selectivity towards reductive amination as well as their compatibility with ammonia. In this respect, here we report a Ni-linear triphos (bis(diphenylphosphinoethyl)phenylphosphine) complex as the first homogeneous Ni-based catalyst for both reductive amination and hydrogenation of nitroarenes. Remarkably, this Ni-triphos complex enabled the synthesis of functionalized and structurally diverse benzylic, heterocyclic, and aliphatic linear and branched primary amines as well as aromatic primary amines starting from inexpensive and easily accessible carbonyl compounds (aldehydes and ketones) and nitroarenes using ammonia and molecular hydrogen. In addition, we also performed DFT studies to know the active catalytic species and mode of reactivity as well as to propose the plausible Ni-based reductive amination mechanism.

## Results and discussion

### Design of the Ni-catalyst for the reductive amination reaction

In recent years triphos-based non-noble metal complexes have emerged as promising catalysts for hydrogenation^6*a*,12*a*–*c*,12*f*–*k*^ and amination reactions.^3*e*,12*d*,*e*^ Due to the strong coordination nature to the central metal atom, triphos-based complexes are suitable to activate hydrogen and ammonia. Inspired by previous amination^3*e*,12*d*,*e*^ and hydrogenation ^6*a*,12*a*–*c*,12*f*–*k*^ processes, we started to design tridentate-Ni complexes for the synthesis of different kinds of primary amines by reductive amination of carbonyl compounds and hydrogenation of nitroarenes.

First we tested the combination of Ni(BF_4_)_2_·6H_2_O and linear triphos (**L1**; bis(diphenylphosphinoethyl) phenylphosphine) as the *in situ* catalyst for the reductive amination of veratraldehyde **1** (3,4-dimethoxy benzaldehyde) to veratrylamine **2** (3,4-dimethoxy benzylamine) in the presence of ammonia and molecular hydrogen as a benchmark reaction (Table 1). Gratifyingly, this *in situ* complex exhibited high activity and selectivity for the reductive amination of veratraldehyde **1** and obtained 96% of the desired product, veratrylamine **2** (Table 1, entry 1). In order to know the reactivity of other tridentate ligands, we tested different PNP, POP and NNN ligands (Table 1, entries 2 to 7). All these tested tridentate ligands (**L2–L7**) showed poor activity with a conversion of up to 25% and in none of these cases the formation of primary amine **1** was observed (Table 1, entries 2 to 7). Notably, the Ni-triphos system was well tolerated with ammonia and due to strong coordination, and this PPP-ligand was found to be appropriate to avoid the formation of a Werner-type ammonia complex. Applying this active system (Ni(BF_4_)_2_–**L1**), next we performed the optimization of the benchmark reaction by evaluating the effect of hydrogen pressure, reaction temperature and catalyst loading (Table 1; entries 8–10). These results revealed that in order to achieve the best yield of **2**, a 40 bar hydrogen pressure, 5 bar ammonia pressure, 100 °C reaction temperature and 4 mol% catalyst (1 : 1 Ni(BF_4_)_2_·6H_2_O–**L1**) are required. Further to know the effect of solvents, different polar (MeOH, EtOH, *t*-BuOH, *t*-amyl alcohol, TFE) and non-polar solvents (toluene, THF) were tested (Table S1†). Among these solvents, trifluroethanol (TFE) was found to be the best solvent. However, in other tested solvents, we did not observe the formation of the desired product, primary amine **2**.

**Table 1:** Ni-catalyzed reductive amination of veratraldehyde: activity and selectivity of Ni-complexes^a^

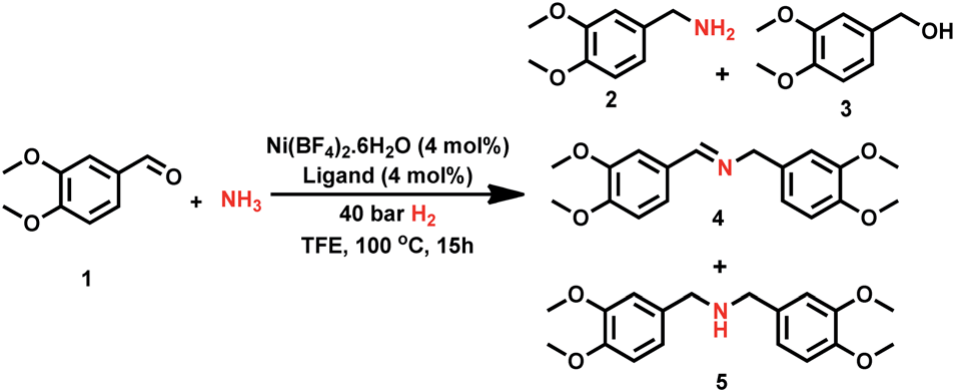						
Entry	Ligand	Conv. (%)	Yield (%)			
			**2**	**3**	**4**	**5**
1^a^	**L1**	>99	96	—	3	—
2^a^	**L2**	15	—	—	13	—
3^a^	**L3**	23	—	—	21	—
4^a^	**L4**	20	—	—	18	—
5^a^	**L5**	12	—	—	10	—
6^a^	**L6**	5	—	—	3	—
7^a^	**L7**	5	—	—	3	—
8^b^	**L1**	>99	92	—	6	—
9^c^	**L1**	>99	91	—	7	—
10^d^	**L1**	>99	93	—	5	—
11^a^	—	10	—	—	8	—
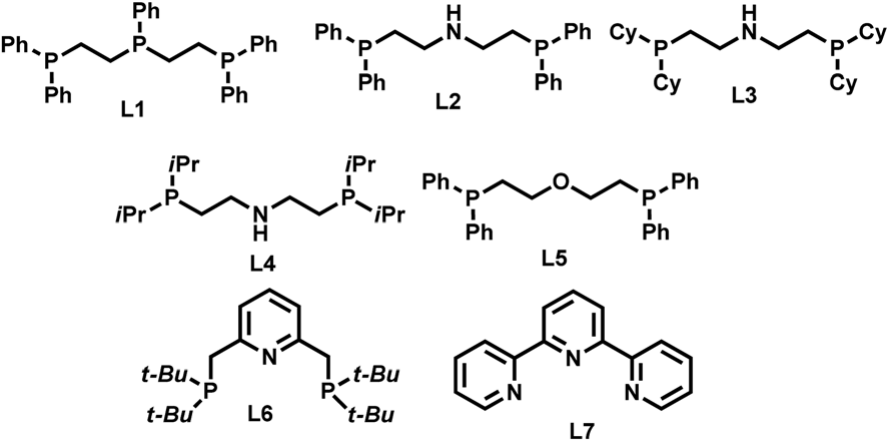						

a Reaction conditions: 0.5 mmol veratraldehyde, 4 mol% Ni(BF_4_)_2_·6H_2_O, 4 mol% ligand, 5 bar NH_3_, 40 bar H_2_ 2 mL trifluoroethanol (TFE), 100 °C, 15 h, and GC yields using *n*-hexadecane as the standard.

b Same as ‘a’ with 20 bar H_2_.

c Same as ‘a’ at 80 °C.

d Same as ‘a’ with 3 mol% catalyst.

After having obtained the best results with the *in situ* generated, Ni(BF_4_)_2_·6H_2_O–**L1** system, we were interested in preparing a molecularly defined complex and testing its activity in the model reaction. Unfortunately, we could not isolate Ni(BF_4_)_2_–**L1** complex due its stability problem. However, the NiCl_2_–**L1** complex was prepared, isolated and tested in the benchmark reaction (see the ESI†)^6*a*^. Noteworthily, this defined complex also exhibited similar activity to the *in situ* Ni(BF_4_)_2_·6H_2_O–**L1** complex and obtained 97% of veratrylamine **2**. In addition, the *in situ* NiCl_2_–**L1** complex also showed good activity and obtained 90% of the desired primary amine **2**. We attempted to isolate a nickel hydride complex; however, we could not due to its highly unstable nature. In order to know the nature of the reaction, catalytic poisoning tests were performed with Hg and PPh_3_ (Table S3†). Addition of either Hg or PPh_3_ to the reaction under standard conditions did not affect either the activity or selectivity of the active Ni-complex. These results showed that the reaction proceeds *via* homogeneous catalysis.

### DFT computational study

Parallel to our experimental studies we carried out detailed density functional theory computations on the reductive amination reaction mechanisms. Since both *in situ* Ni(BF_4_)_2_·6H_2_O-triphos (**L1**) and the well-defined NiCl_2_–**L1** complexes showed the same activity and selectivity, we used this well-defined complex as the pre-catalyst, which can be converted with NH_3_ to the active catalyst bearing the Ni–H bond [**L1**NiH]^+^(**I**). In our computations we used the real-size catalyst as well as phenylmethanimine (Ph–CH=NH) for benzaldehyde as the substrate for the reaction. To evaluate the effect of the van der Waals dispersion correction (GD3BJ) and solvation (SMD) of 2,2,2-trifluoroethanol (TFE) we tested different combinations and several functionals. Based on these testing results, we discussed our results including dispersion and solvation (B3PW91–GD3BJ–SMD). The computational details for the models and methods are listed in the ESI(S7).†

**Fig. 1:**
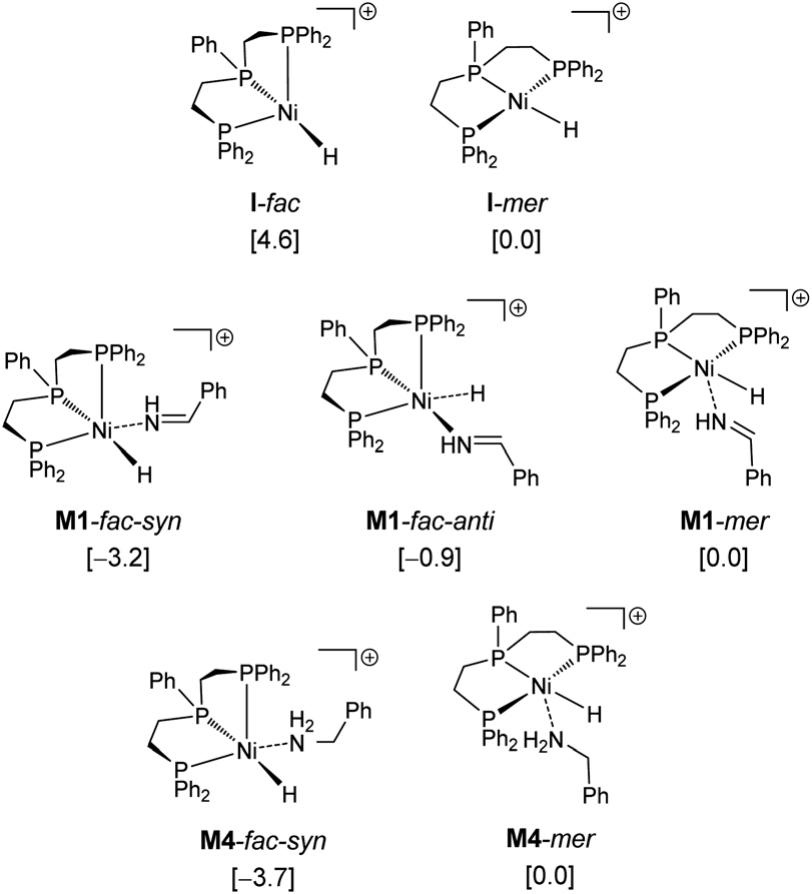
Relative energies (kcal mol^−1^) of the Ni-catalyst and Ni-complexes.

Since complex **I** can have *fac* (facial) and *mer* (meridional) conformations, we first computed their relative energy (Fig. 1). It is found that the **I**-*mer* is more stable than **I**-*fac* by 4.6 kcal mol; and **I**-*mer* should be the dominant isomer under equilibrium (>99.9%). Since complex **I** has 16 valence electrons, we tested the stability of the triplet state, and the singlet state is more stable than the corresponding triplet state by 51.8 and 50.2 kcal mol^−1^ for the *fac*- and *mer*-coordination, respectively. Under the coordination of imines, however, the complex (**M1**-*fac-syn*) from **I**-*fac* is more stable than that (**M1**-*mer*) from **I**-*mer* by 3.2 kcal mol^−1^ (>99%), indicating that **M1**-*fac-syn* should be the major component in the reaction medium and this indicates the stability change upon substrate coordination. Indeed, imine coordination to **I**-*mer* is slightly endergonic by 0.2 kcal mol^−1^, while imine coordination to **I**-*fac* is exergonic by 7.6 kcal mol^−1^. It is also noted that **M1**-*fac-syn* is more stable than **M1**-*fac-anti* by 2.4 kcal mol^−1^.

**Fig. 2:**
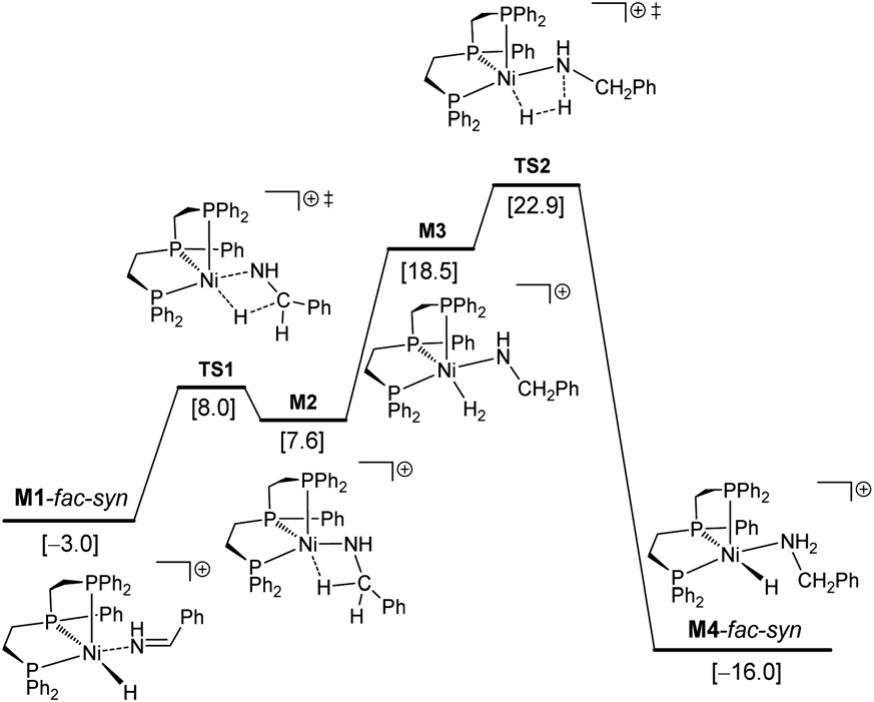
Potential free energy surface (kcal mol^−1^) with the reference of **I**-*mer*, Ph–CH=NH and H_2_.

Therefore, we used **M1**-*fac-syn* for our computations. It is also noted that only the potential energy surface of the *fac*-coordination has been obtained; and it is not possible to have the potential energy surface for the *mer*-coordination. For the *mer*-coordination, only the complexes of imine and amine coordination have been found and they have higher energy and are less stable than the *fac*-coordination. The computed potential free energy surface is shown in Fig. 2.

In **M1**-*fac-syn*, imine coordination is the nitrogen lone pair instead of the expected C=N double bond. Starting from **M1**-*fac-syn*, the first step is the transfer of the hydride to the carbon center of the C=N double bond *via* the transition state (**TS1**) due to the different electronegativities of C and N atoms. This hydride transfer results in the formation of an amido complex with C–H agostic interaction with the Ni center, **M2**. This hydride transfer needs a free energy barrier of 11.0 kcal mol^−1^ and is endergonic by 10.6 kcal mol^−1^. The next step is the breaking of the C–H agostic interaction *via* H_2_ coordination with the formation of intermediate **M3**, and this step is endergonic by 10.9 kcal mol^−1^. In **M3**, molecular H_2_ coordination has been found instead of oxidative addition. Having formed **M3** with molecular H_2_ coordination, the next step is metathesis (hydrogenolysis) instead of reductive elimination and the formed amine (Ph–CH_2_–NH_2_) still coordinates to the Ni center, **M4**. This step needs a free energy barrier of 4.4 kcal mol^−1^ and is strongly exergonic by 34.5 kcal mol^−1^.

On the basis of **I**-*mer* and Ph–CH=NH, the apparent free energy barrier is 22.9 kcal mol^−1^, and this barrier is in reasonable agreement with an applied high reaction temperature of 100–120 °C and a long reaction time of 24 hours. In addition, the endergonic molecular H_2_ coordination and the high apparent barrier reasonably explain the need for a high H_2_ pressure of 40–50 bar. It is noted that the computed apparent barrier is 41.6 kcal mol^−1^ in the gas phase, and 41.9 kcal mol^−1^ under the consideration of the solvation effect. In the case of van der Waals dispersion correction, the apparent barrier becomes 19.2 in the gas phase. In the case of solvation and dispersion corrections, the apparent barrier is 22.9 kcal mol^−1^. This demonstrates the effect of dispersion correction. In addition, we tested other functional methods including solvation and dispersion corrections and found that the apparent barrier is 28.1, 21.1, 25.4 and 27.8 kcal mol^−1^ for B3LYP, BP86, M06L and MN15, respectively.

**Scheme 1:**
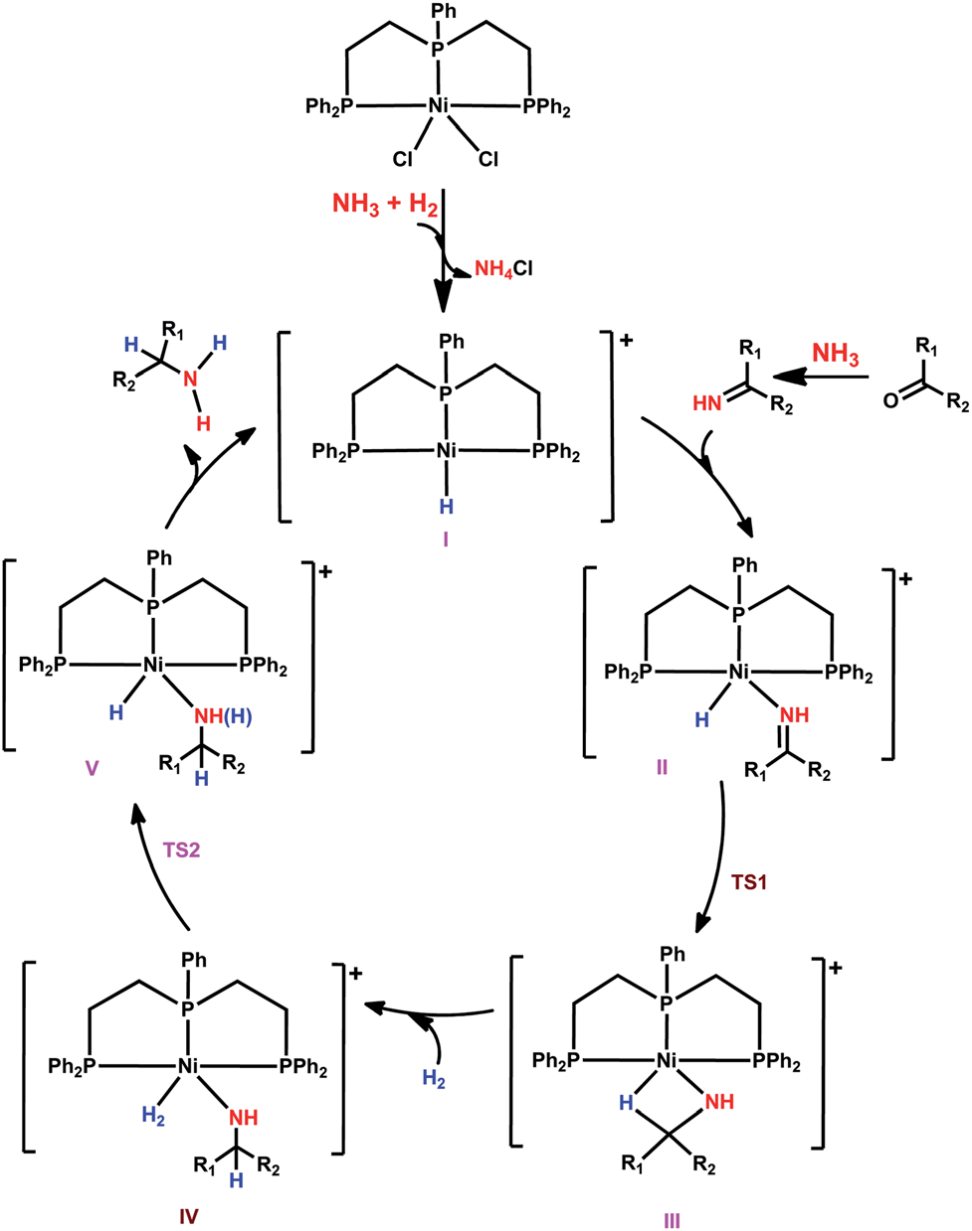
Plausible reaction mechanism for the NiCl_2_-triphos catalyzed reductive amination for the synthesis of primary amines.

Having studied the catalytically active species by DFT calculations, we proposed the plausible mechanism for the Ni-triphos catalyzed reductive amination of carbonyl compounds with ammonia and molecular hydrogen (Scheme 1). After the generation of the active catalyst with the Ni–H functionality (**I**), the mechanism has three main steps, (i) substrate coordination (**II**) followed by the Ni–H selective insertion into the C=N double bond (**TS1**) and the formation of agostically interacting intermediate (**III**); and (ii) H_2_ coordination (**IV**) followed by H_2_ metathesis (**TS2**) and the formation of amine coordinated intermediate (**V**). The final step is the release of the amine and regeneration of the active catalyst. Overall, the reaction has an inner-sphere mechanism and the rate-determining step is H_2_ metathesis, and the apparent barrier is about 23 kcal mol^−1^. All this reasonably explains the need for a high H_2_ pressure of 40–50 bar and the long reaction time (Table 1).

### Synthesis of benzylic and aliphatic linear primary amines

**Scheme 2:**
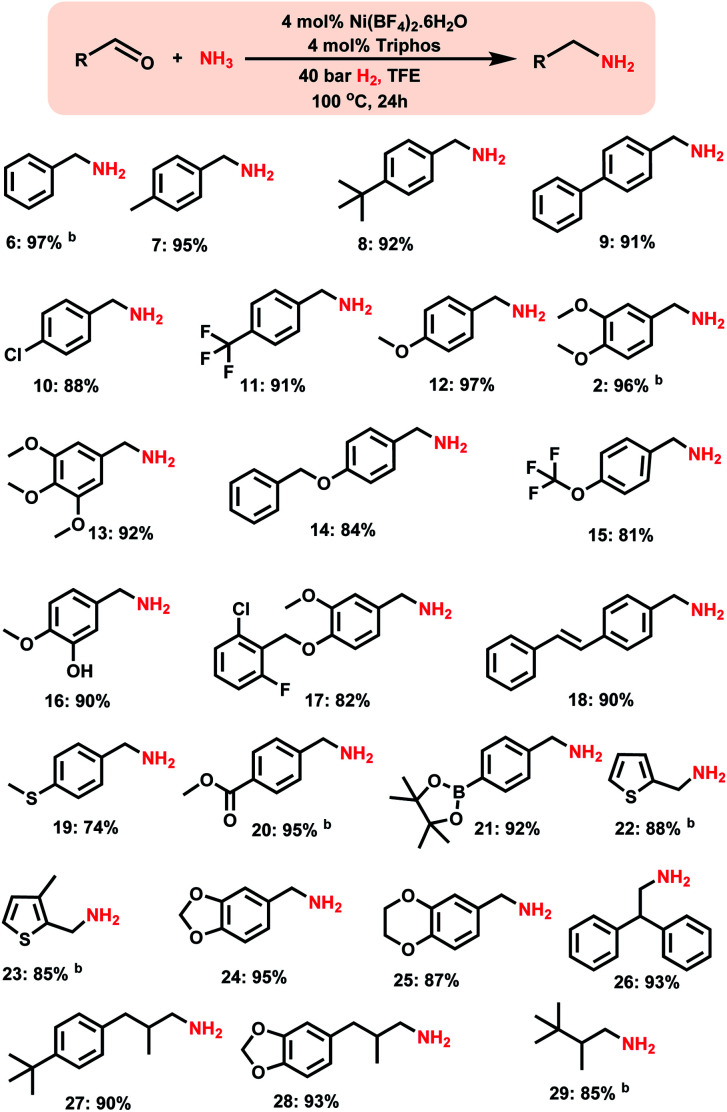
Synthesis of linear primary amines using the Ni-triphos complex.^*a a*^Reaction conditions: 0.5 mmol aldehyde, 4 mol% Ni(BF_4_)_2_·6H_2_O, 4 mol% triphos (**L1**), 5–7 bar NH_3_, 40 bar H_2_, 2 mL degassed trifluoroethanol (TFE), 100 °C, 24 h, and isolated yields. ^*b*^GC yields using *n*-hexadecane as the standard. Isolated as free amines and converted to hydrochloride salts. The corresponding hydrochloride salts were subjected to NMR analysis.

**Scheme 3:**
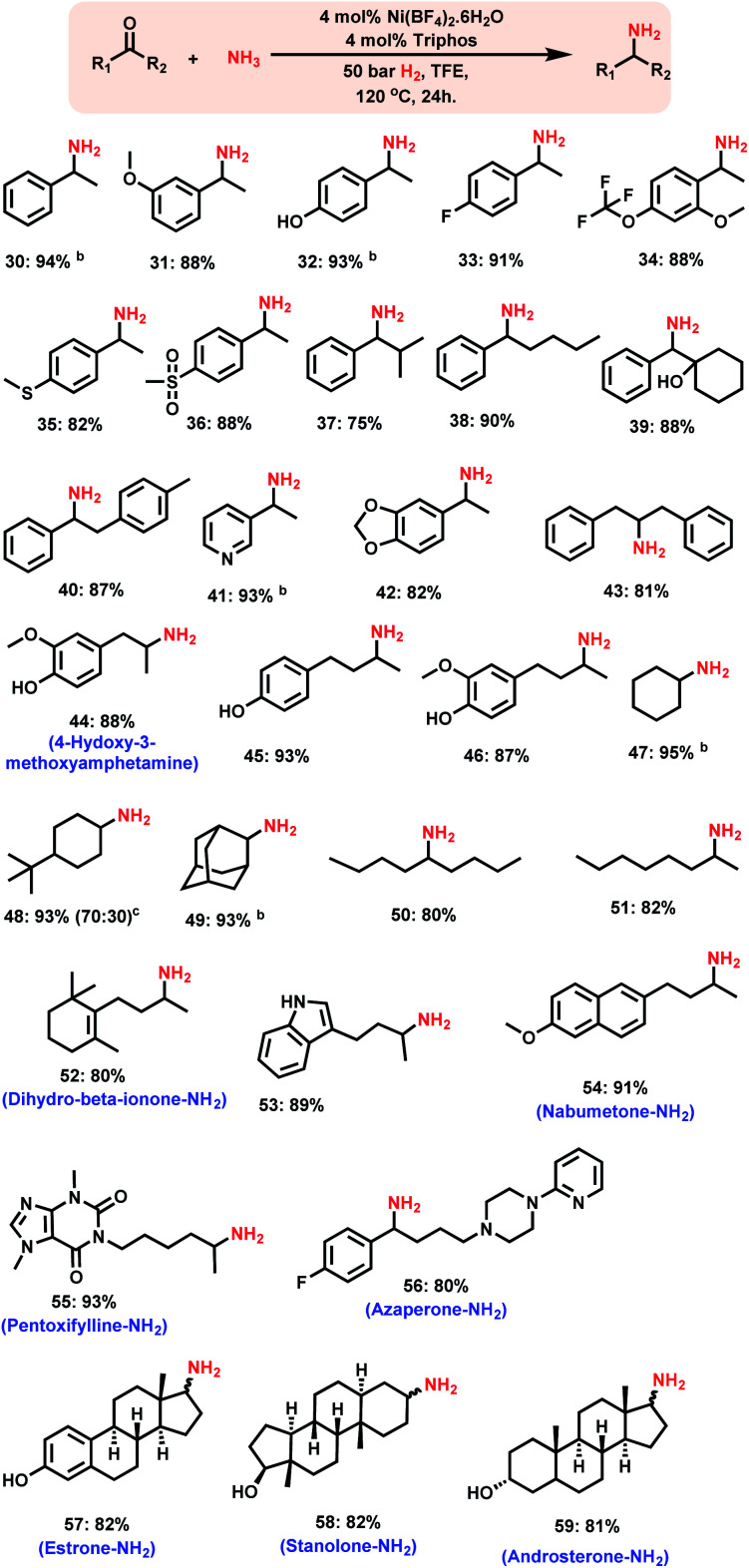
Nickel-triphos catalysed synthesis of branched primary amines.^*a a*^Reaction conditions: 0.5 mmol ketone, 4 mol% Ni(BF_4_)_2_·6H_2_O, 4 mol% triphos (**L1**), 5–7 bar NH_3_, 50 bar H_2_, 2 mL degassed trifluoroethanol (TFE), 120 °C, 24 h, and isolated yields. ^*b*^GC yields using *n*-hexadecane as the standard. Isolated as free amines and converted to hydrochloride salts. The corresponding hydrochloride salts were subjected to NMR analysis.

After having investigated the nickel-triphos (**L1**) as the most active catalyst system, we explored its (*in situ* system) general applicability for the preparation of various primary amines starting from carbonyl compounds. As shown in Schemes 2 and 3 this Ni-triphos catalyst allowed for the amination of both aldehydes and ketones with ammonia in the presence of molecular hydrogen and obtained structurally diverse and functionalized linear and branched primary amines. Simple aldehydes and substrate bearing electron -donating and -withdrawing groups including halide substituted ones were reacted smoothly and the corresponding primary amines were obtained in good to excellent yields (Scheme 2, products **6–11**). For any given amination/hydrogenation catalyst, achieving a high degree of chemoselectivity is challenging and important in organic synthesis and drug discovery.

To demonstrate this aspect, reductive amination of various functionalized aldehydes was performed. Interestingly, the functionalized aldehydes containing ether, phenolic, C–C double bond, ester, boronic acid ester and thioether groups were all highly selectively aminated and the corresponding linear primary amines were obtained in up to 96% yield, without the reduction of other functional groups (Scheme 2, products **12–21**). In addition, heterocyclic primary amines were prepared in up to 95% yield (Scheme 2, products **22–25** and **28**). Primary amines of 3,4-methylenedioxy and benzo-1,4-dioxane, which represent versatile motifs in many drugs and natural products, were prepared in up to 96% yield (Scheme 2, products **24** to **25** and **28**). Further, aliphatic aldehydes, which are difficult to react were also aminated and the corresponding primary amines were obtained in good to excellent yields (Scheme 2; products **26–29**).

### Synthesis of branched primary amines from ketones

Compared to that of linear primary amines, the synthesis of branched primary amines starting from ketones is more challenging, because the reduction of corresponding imines from ketones is more difficult than that of the imines of corresponding aldehydes.

Remarkably, the Ni-triphos complex is highly active and selective for the reductive amination of ketones with ammonia and hydrogen. As a result, all kinds of ketones were efficiently aminated to produce corresponding branched primary amines in high yields (Scheme 3). In addition to the simple and functionalized ketones, the ones bearing easily coordinating groups such as –NH, and –OH phenolic groups as well as pyridines to metals were also smoothly reacted with ammonia and gave corresponding primary amines in up to 94% yield (Scheme 3, products **41**, **44–46**, **53** and **56**). In addition, the synthesis of various aliphatic branched primary amines, which are difficult to prepare,^3*i*,*n*^ was performed with different ketones using this Ni-triphos system (Scheme 3, products **44–51**). A more valuable application of this Ni-based protocol has been demonstrated by performing the amination of structurally complex life science molecules and steroid derivatives (Scheme 3). Gratifyingly, by applying the Ni-triphos catalyst the –NH_2_ moiety has been introduced in nabumetone, pentoxifylline, azaperone, estrone, androsterone and stanolone (Scheme 3, products **54–60**).

### Synthesis of aromatic primary amines by Ni-catalyzed hydrogenation of nitroarenes

**Scheme 4:**
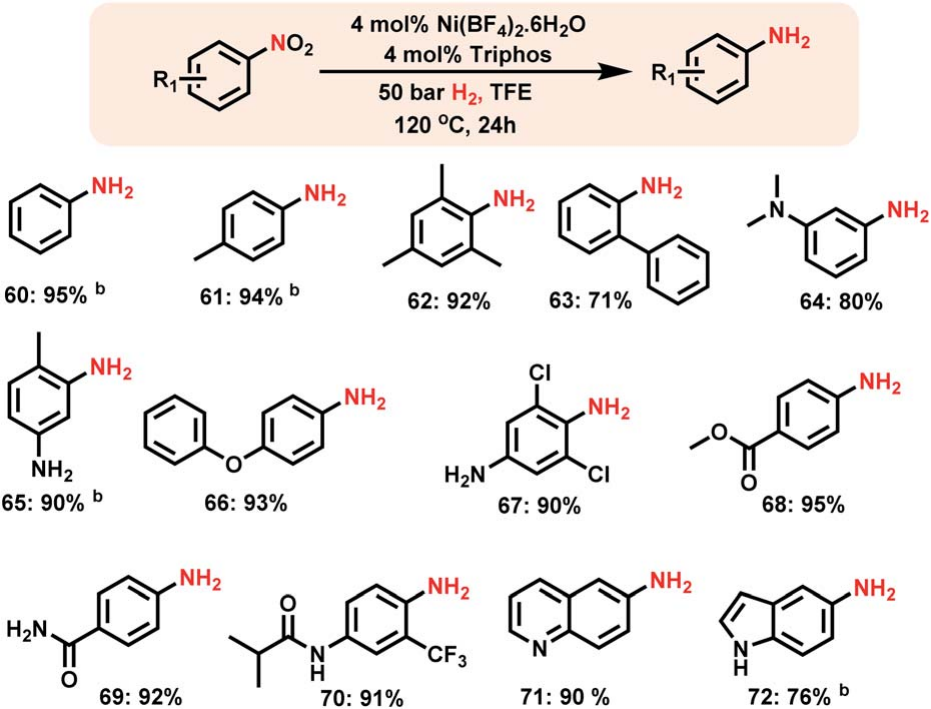
Homogeneous Ni-catalyzed synthesis of aromatic primary amines.^*a a*^Reaction conditions: 0.5 mmol substrate, 4 mol% Ni(BF_4_)_2_·6H_2_O, 4 mol% triphos (**L1**), 50 bar H_2_, 2 mL degassed trifluoroethanol (TFE), 120 °C, 24 h, and ^*b*^GC yields using *n*-hexadecane as the standard.

The design of homogeneous catalysts for the hydrogenation of nitroarenes to anilines continues to be challenging.^4*c*–*f*^ Here we explored the applicability of Ni-triphos as a homogeneous catalyst for the hydrogenation of nitroarenes. Advantageously this *in situ* generated Ni–**L1** complex also exhibited excellent activity for the hydrogenation of nitroarenes (Scheme 4).

Nitrobenzenes containing both electron-donating and -withdrawing groups were selectively hydrogenated to obtain corresponding anilines in up to 95% yield (Scheme 4, products **62** to **72**). Functionalized nitroarenes containing esters, amides and ethers as well as halide groups were hydrogenated to anilines by tolerating these functional groups without being reduced (Scheme 4, products **66–70**).

## Conclusions

In conclusion, for the first time we introduced a homogeneous Ni-based catalyst for both reductive amination of carbonyl compounds and hydrogenation of nitroarenes to prepare all kinds of primary amines. The key to success for this synthesis is the use of a linear triphos-ligated Ni-complex. By applying this Ni-based homogeneous catalyst, starting from inexpensive and easily available carbonyl compounds and nitroarenes using abundant and atom economical reagents such as ammonia and molecular hydrogen, commercially and industrially important as well as pharmaceutically relevant aromatic, heterocyclic, and aliphatic primary amines were synthesized in good to excellent yields. DFT computations revealed that the overall reaction has an inner-sphere mechanism with H_2_ metathesis as the rate-determining step and this reasonably explains the need for the high H_2_ pressure and long reaction time on the basis of the computed apparent barriers.

## Conflicts of interest

The authors declare no competing financial interest.

## Supplementary Material

SC-011-D0SC01084G-s001
