# Supplementary material for: General and selective synthesis of primary amines using Ni-based homogeneous catalysts
Source: Chem Sci. 2020 Mar 25;11(17):4332–9. doi: 10.1039/d0sc01084g (PMC8152594; doi:10.1039/d0sc01084g)

Electronic Supplementary Material (ESI) for Chemical Science.
This journal is © The Royal Society of Chemistry 2020

Supporting Information

General and selective synthesis of primary amines using Ni-based homogeneous catalysts

Dr. Kathiravan Murugesan, Dr. Zhihong Wei, Vishwas G. Chandrashekhar, Dr. Haijun Jiao,* Prof. Matthias Beller,* and Dr. Rajenahally V. Jagadeesh*

Leibniz-Institut für Katalyse e.V. an der Universität Rostock, Albert-Einstein Str. 29a, Rostock, D-18059, Germany.
*Corresponding authors:

Haijun Jiao (E-mail: haijun.jiao@catalysis.de)

Matthias Beller (E-mail: matthias.beller@catalysis.de)

Rajenahally V. Jagadeesh (E-mail: jagadeesh.rajenahally@catalysis.de)

Table of Content

S1 Materials and methods

S2 Synthesis and characterization of $[(Ph_2PCH_2CH_2)_2PPh]NiCl_2$ complex

S3 General procedure for the synthesis of primary amines

S4 General procedure for the hydrogenation of nitroarenes to aromatic primary amines

S5 Solvent screening

S6 Catalytic poison test

S7 Computational details

S8 NMR data

S9 NMR and HRMS spectra

S1. Materials and methods

Unless specified, all substrates were obtained commercially from various chemical companies and their purity has been checked before use. Unless otherwise stated, all commercial reagents were used as received without purification. All catalytic reactions were carried out in 300 mL and 100 mL autoclaves (PARR Instrument Company). In order to avoid unspecific reactions, catalytic reactions were carried out either in glass vials, which were placed inside the autoclave, or glass/Teflon vessel fitted autoclaves. GC conversion and yields were determined by GC-FID, HP6890 with FID detector, column HP530 m x 250 mm x 0.25 µm. ^{1}H, ^{13}C NMR data were recorded on a Bruker ARX 300 and Bruker ARX 400 spectrometers using DMSO-d_6, CD_3OD and $CDCl_3$ solvents. HRMS data were recorded on EI-HRMS: Mass Spectrometer MAT 95XP (Thermo Electron), 70eV.

*X-ray crystal structure analysis of complex **A**:* Data were collected on a Bruker Kappa APEX II Duo diffractometer. The structure was solved by direct methods (SHELXS-97: Sheldrick, G. M. Acta Cryst. **2008**, *A64*, 112.) and refined by full-matrix least-squares procedures on F^2 (SHELXL-2014: Sheldrick, G. M. Acta Cryst. **2015**, *C71*, 3.). XP (Bruker AXS) was used for graphical representations.

S2. Synthesis and characterization of $[(Ph_2PCH_2CH_2)_2PPh]NiCl_2$ complex [6a]

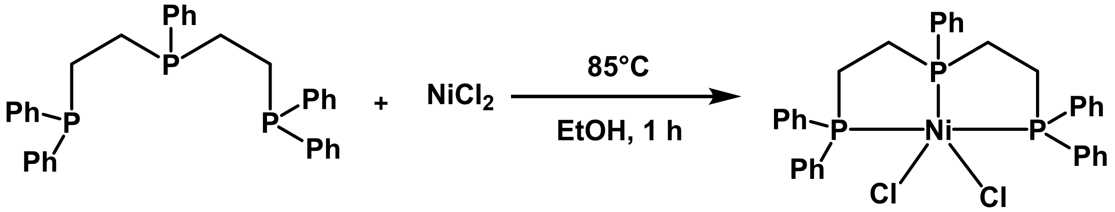

The anhydrous $NiCl_2$ (64.8 mg, 1.0 mmol) in ethanol (6 mL) was stirred at 85 °C under argon to obtain pale yellow solution. To this, triphos ((phenylphosphanediyl) bis(ethane-2,1-diyl)) bis (diphenylphosphane) (535.55 mg, 1. mmol) was added and stirring was continued at 85 °C for 1 h. Upon adding, the ligand pale yellow solution was turned into brown color. After the completion of reaction, the heating and stirring was switched off and allowed to cool for overnight. Dark brown crystals were observed along with a brown solid. The crystals were separated carefully and recrystallized again with ethanol for measuring X-ray diffraction analysis. The remaining reaction mixture was concentrated under reduced pressure to remove ethanol. The brown colored solid was washed with diethyl ether (3 x 10 mL) and dried under high vacuum for 2h to get the [(Ph2PCH2CH2)2PPh]$NiCl_2$ complex as pale brown color solid (600 mg , 90% yield).

^{1}H NMR (400 MHz, Chloroform-d) δ 8.85 – 8.50 (m, 2H), 8.03 – 7.76 (m, 4H), 7.68 – 7.26 (m, 19H), 4.41 – 3.98 (m, 2H), 3.44 – 2.87 (m, 4H), 2.34 – 2.02 (m, 2H).

^{13}C NMR (101 MHz, Chloroform-*d*) δ 134.75 (Vd, J = 10.2 Hz), 134.10 (Vt, J = 5.7 Hz), 133.62 , 132.88 (Vt, J = 5.7 Hz), 132.00 (Vd, J = 15.2 Hz), 130.16 (Vd, J = 10.6 Hz), 129.59 (Vt, J = 5.1 Hz), 129.17 (Vt, J = 5.4 Hz), 30.90 , 28.47 .

^{31}P NMR (162 MHz, Chloroform-d) δ 111.06 (t, J = 48.3 Hz), 48.55 (d, J = 48.3 Hz).

ESI-HRMS (m/z pos): Calculated for [C34H33ClNiP3]: 627.0842; found: 627.0852.

Elemental analysis: Calculated for [C34H33Cl2NiP3]: C, 61.49; H, 5.01; Cl, 10.68; Ni, 8.84. Found: C, 61.81; H, 4.58; Cl, 10.36; Ni, 7.83.

Crystal data for complex A: $C_{34}H_{33}Cl_2NiP_3$, M = 664.12, monoclinic, space group $P2_1/m$, a = 8.1732(6), b = 20.6167(15), c = 9.3736(7) Å, β = 103.6446(11)°, V = 1534.9(2) Å^3, T = 150(2) K, Z = 2, 26537 reflections measured, 3812 independent reflections (R_{int} = 0.0219), final R values ($I > 2\sigma(I)$): R_1 = 0.0232, wR_2 = 0.0593, final R values (all data): R_1 = 0.0246, wR_2 = 0.0604, GOF on F^2: 1.047, 196 parameters.

CCDC 1869414 contains the supplementary crystallographic data for this paper. These data are provided free of charge by The Cambridge Crystallographic Data Centre.

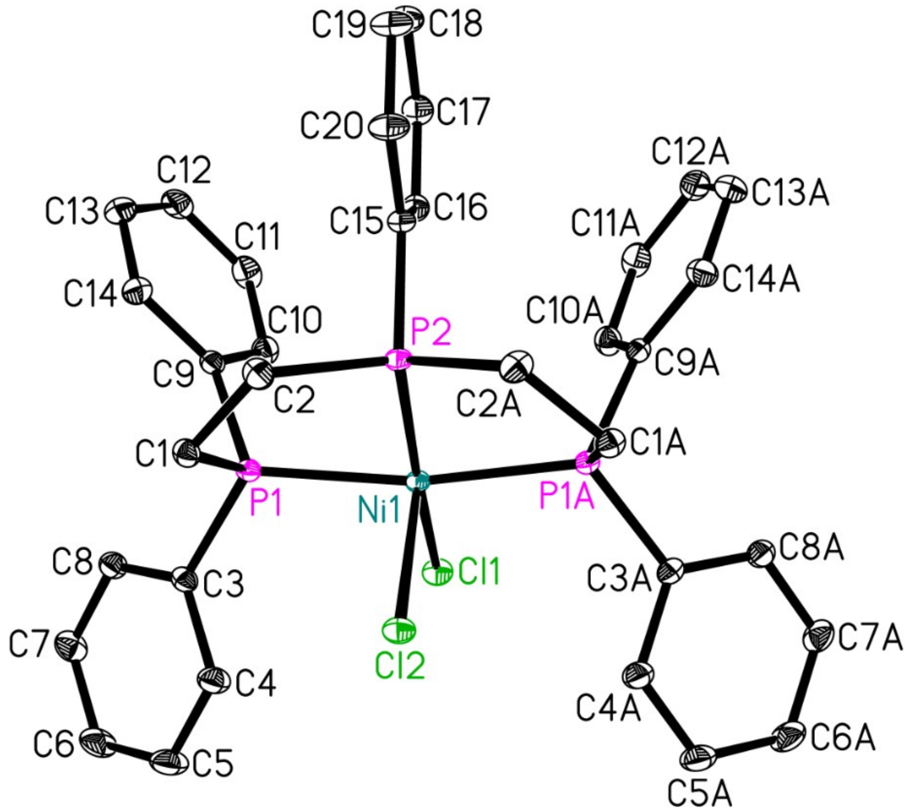

Figure S1. Molecular structure of complex **A** in the crystal. Displacement ellipsoids are drawn at the 30% probability level. Hydrogen atoms are omitted for clarity. Symmetry transformation used to generate equivalent atoms: x, -y+1/2, z.

S3. General procedure for the synthesis of primary amines

The magnetic stirring bar and $Ni(BF_4)_2{\cdot}6H_2O$ (4 mol%) and linear triphos ((**L1**; phenylphosphanediyl)bis(ethane-2,1-diyl))bis(diphenylphosphane) (4 mol%) were transferred to 8 mL glass vial and then 2 mL degassed (degassed under argon for 15 minutes before adding) trifluoroethanol (TFE) solvent was added. The colorless solution turned in to brown color, which was stirred under argon for 15 minutes. Then, 0.5 mmol of corresponding carbonyl compound was added and the vial was fitted with septum, cap and needle. The reaction vials (8 vials with different substrates at a time) were placed into a 300 mL autoclave. The autoclave was flushed with hydrogen twice at 30 bar pressure and then it was pressurized with 5-7 bar ammonia gas and 40 bar hydrogen. The autoclave was placed into an aluminum block preheated at 120 °C-130 (placed 30 minutes before counting the reaction time in ordered to attain reaction temperature) and the reactions were stirred for required time. During the reaction, the inside temperature of the autoclave was measured to be 100-120 °C (10 ^{0}C less then temperature set at aluminum block) and this temperature was used as the reaction temperature. After the completion of the reactions, the autoclave was cooled to room temperature. The remaining ammonia and hydrogen were discharged and the vials containing reaction products were removed from the autoclave. The reaction mixture was filtered off and washed thoroughly with ethyl acetate. The reaction products were analyzed by GC-MS. The corresponding primary amines were converted to their respective hydrochloride salt and characterized by NMR and GC-MS analysis. For converting into hydrochloride salt of amine, 1-2 mL methanolic HCl (0.5M HCl in methanol) was added to the ether solution of respective amine and stirred at room temperature for 4-5 h. Then, solvent was removed, and the resulted hydrochloride salt of amine is dried under high vacuum. The yields were determined by GC for the selected amines: After completion of the reaction, n-hexadecane (100μL) as standard was added to the reaction vials and the reaction products were diluted with ethyl acetate followed by filtration using plug of silica and then analyzed by GC.

S4. General procedure for the hydrogenation of nitroarenes to aromatic primary amines

The magnetic stirring bar and $Ni(BF_4)_2 \cdot 6H_2O$ (4 mol%) and linear triphos ((phenylphosphanediyl)bis(ethane-2,1-diyl))bis(diphenylphosphane) (4 mol%) were transferred to 8 mL glass vial and then 2 mL degassed (degassed under argon for 15 minutes before adding) trifluoroethanol (TFE) solvent was added. The colorless solution turned in to brown color, which was stirred under argon for 15 minutes. Then, 0.5 mmol corresponding nitro compounds was added and the vial was fitted with septum, cap and needle. The reaction vials (8 vials with different substrates at a time) were placed into a 300 mL autoclave. The autoclave was flushed with hydrogen twice at 30 bar pressure and then it was pressurized 40 bar hydrogen. The autoclave was placed into an aluminum block preheated at 130 °C (placed 30 minutes before counting the reaction time in ordered to attain reaction temperature) and the reactions were stirred for required time. During the reaction the inside temperature of the autoclave was measured to be 120 °C and this temperature was used as the reaction temperature. After the completion of the reactions, the autoclave was cooled to room temperature. The remaining hydrogen was discharged and the vials containing reaction products were removed from the autoclave. The reaction mixture was filtered off and washed thoroughly with ethyl acetate. The reaction products were analyzed by GC-MS. The corresponding anilines were isolated by column chromatography to their respective amines, which were characterized by NMR and GC-MS analysis. The yields were determined by GC for the selected amines: After completion of the reaction, n-hexadecane (100μL) as standard was added to the reaction vials and the reaction products were diluted with ethyl acetate followed by filtration using plug of silica and then analyzed by GC.

S5. Solvent screening

Table S1. Influence of solvents on Ni-tripos catalyzed reductive amination of veratraldehyde

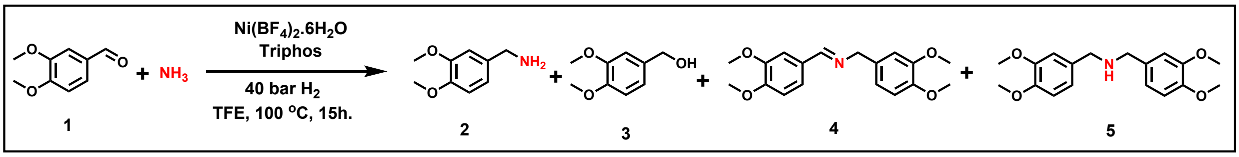

Entry	Solvent	Conv. (%)	Yield of 2 (%)	Yield of 3 (%)	Yield of 4 (%)	Yield of 5 (%)
1	Toluene	4	-	-	2	-
2	THF	20	-	-	18	-
3	*t*-Amyl alcohol	22	-	-	20	-
4	*t*-buOH	25	-	-	23	-
5	EtOH	50	-	-	48	-
6	MeOH	70	-	-	68	-
7	Trifluoroethanol	>99	96	-	2	-

Reaction conditions: 0.5 mmol veratraldehyde, 4 mol% $Ni(BF_4)_2.6H_2O$, 4 mol% triphos (**L1**), 5-7 bar NH_3, 40 bar H_2, 2 mL solvent, 100 °C, 15 h, GC yields using n-hexadecane as standard.

Table S2. Influence of solvents on Ni-triphos catalyzed hydrogenation of nitrobenzene

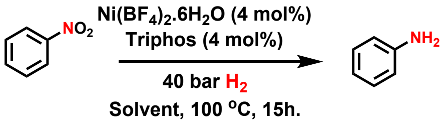

Entry	Solvent	Conv. (%)	Yield of aniline (%)
1[a]	Toluene	4	2
2[a]	THF	6	4
3[a]	IPA	32	30
4[a]	EtOH	23	21
5[a]	MeOH	13	11
6[a]	Trifluoroethanol	72	70
7[b]	Trifluoroethanol	75	72
8[c]	Trifluoroethanol	>99	97

Reaction conditions: 0.5 mmol nitrobenezene, 4 mol% $Ni(BF_4)_2.6H_2O$, 4 mol% triphos (L1), 40 bar H_2, 2 mL solvent, 100 °C, 15 h, GC yields using n-hexadecane as standard. [b]same as for 24h. [c]Same as 'a' at 120 °C for 24h.

S6. Catalytic poison test

Table S3.

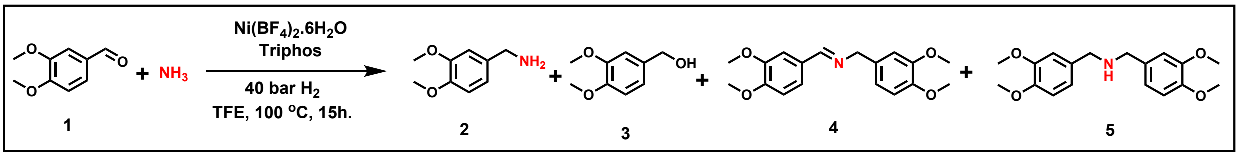

Entry	Catalytic poison	Conv. (%)	Yield of 2 (%)	Yield of 3 (%)	Yield of 4 (%)	Yield of 5 (%)
1a	Hg (2 eq)	>99	93	-	5	-
2 a	PPh3 (50 mol%)	>99	92	-	6	-

Reaction conditions: 0.5 mmol veratraldehyde, 4 mol% $Ni(BF_4)_2.6H_2O$, 4 mol% triphos (**L1**), 5-7 bar NH_3, 40 bar H_2, 2 mL trifluoroethanol (TFE), 100 °C, 15 h, GC yields using n-hexadecane as standard.

S7. Computational details

DFT calculation

All calculations were carried out with Gaussian 16 program.[S1] Geometry optimization was carried out in gas phase at the B3PW91[S2] level with the TZVP [S3] basis set. All optimized structures were further characterized either as energy minimums without imaginary frequencies or transition states with only one imaginary frequency by frequency calculations, which provided zero-point vibrational energies and thermodynamic corrections to enthalpy and Gibbs free energy at 298.15 K under 1 atmosphere. On the basis of B3PW91/TZVP geometries in gas phase, single-point energies including solvation effect of 2,2,2-trifluoroethanol (TFE) as solvent (dielectric constant ε = 26.69) based on solute electron density (SMD [S4]) and van der Waals dispersion (D3[5]) with the gas phase optimized geometries were computed (B3PW91-SMD-D3). The Gibbs free energies at 298.15 K were further corrected to standard state in solution with a standard concentration of 1 mol/L (p = 24.5 atm) from standard state in gas phase (p = 1 atm).

S1. Frisch, M. J. *et al.* Gaussian software, version 16 revision D01. *Gaussian Inc.: Wallingford, CT, USA* (2016)

S2. Becke, A. D. Density-functional thermochemistry. III. The role of exact exchange. *J. Chem. Phys.* 1993, **98**, 5648-5652.

S3. Schäfer, A., Huber, C. & Ahlrichs, R. Fully optimized contracted Gaussian basis sets of triple zeta valence quality for atoms Li to Kr. *J. Chem. Phys.* 1994, **100**, 5829-5835

S4. Marenich, A. V., Cramer, C. J. & Truhlar, D. G. Universal Solvation Model Based on Solute Electron Density and on a Continuum Model of the Solvent Defined by the Bulk Dielectric Constant and Atomic Surface Tensions. *J. Phys. Chem. B* 2009, **113**, 6378-6396

S5. Grimme, S., Ehrlich, S. & Goerigk, L. Effect of the damping function in dispersion corrected density functional theory. *J. Comput. Chem.* 2011, **32**, 1456-1465

Table S4. Energetic data from B3PW91 full optimization (B3PW91/FOpt) as well as from single-point calculations with the B3PW91 optimized geometries including solvation effect and van der Waals dispersion correction (B3PW91-SCRF-D3//SP)

	B3PW91		B3PW91-SCRF-D3//SP
H2	HF=-1,178636<br>ZPE= 0,010064<br>NImag=0<br>Htot= -1,165268<br>Gtot= -1,180066	DH= 0,013368<br>DG = -0,001430	HF=-1,1784042
Ph-CH=NH	HF=-325,6720957<br>ZPE= 0,122294<br>NImag=0<br>Htot= -325,542365<br>Gtot= -325,580510	DH = 0,129730<br>DG = 0,091586	HF=-325,7093359
PhCH2NH2	HF=-326,8931026<br>ZPE= 0,145783<br>NImag=0<br>Htot= -326,739071<br>Gtot= -326,779504	DH = 0,154032<br>DG = 0,113599	HF=-326,9349566
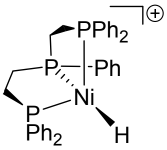	HF=-3848,1898173<br>ZPE= 0,581578<br>NImag=0<br>Htot= -3847,571055<br>Gtot= -3847,681819	DH = 0,618763<br>DG = 0,507999	HF=-3848,496691
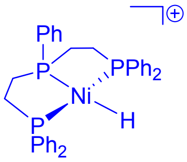	HF=-3848,1958745<br>ZPE=0,581223<br>NImag=0<br>Htot= -3847,577519<br>Gtot= -3847,689688	DH = 0,618355<br>DG = 0,506187	HF=-3848,5022471
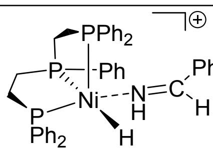	HF=-4173,8847115<br>ZPE= 0,706071<br>NImag=0<br>Htot= -4173,133471<br>Gtot= -4173,263221	DH = 0,751241<br>DG = 0,621491	HF=-4174,2400989
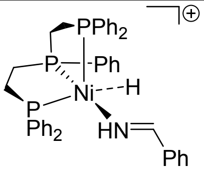	HF=-4173,8809159<br>ZPE= 0,705803<br>NImag=0<br>Htot= -4173,129924<br>Gtot= -4173,259768	DH = 0,750992<br>DG = 0,621148	HF=-4174,2359305
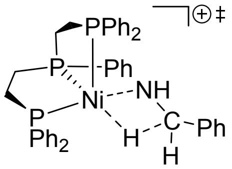	HF=-4173,8560405<br>ZPE= 0,704914<br>NImag=1 (-515)<br>Htot= -4173,106739<br>Gtot= -4173,233294	DH = 0,749301<br>DG = 0,622746	HF= -4174,2238259
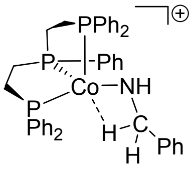	HF=-4173,8604368<br>ZPE=0,707619<br>NImag=0<br>Htot=-4173,108237<br>Gtot= -4173,235999	DH = 0,752200<br>DG = 0,624438	HF=-4174,2261163
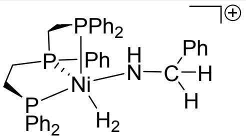	HF=-4175,0315883<br>ZPE=0,724763<br>NImag=0<br>Htot=-4174,260570<br>Gtot= -4174,392754	DH = 0,771018<br>DG = 0,638834	HF=-4175,4030651

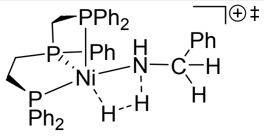	HF=-4175,0233613<br>ZPE= 0,724067<br>NImag=1 (-783)<br>Htot= -4174,253726<br>Gtot= -4174,384676	DH = 0,769635<br>DG = 0,638685	HF=-4175,3958716
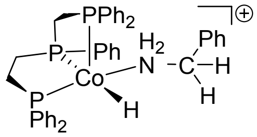	HF=-4175,0995345<br>ZPE=0,730489<br>NImag=0<br>Htot=-4174,323380<br>Gtot= -4174,454313	DH = 0,776155<br>DG = 0,645222	HF=-4175,4642953
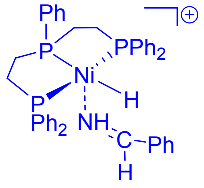	HF=-4173,8702341<br>ZPE= 0,704795<br>NImag=0<br>Htot= -4173,120042<br>Gtot= -4173,251463	DH = 0,750192<br>DG= 0,618771	HF=-4174,2322667
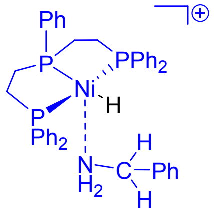	HF=-4175,090463<br>ZPE=0,728990<br>NImag=0<br>Htot=-4174,315506<br>Gtot= -4174,448468	DH = 0,774957<br>DG = 0,641995	HF=-4175,4572105

Table S5. B3PW91 optimized Cartesian Coordinates

Ph-CH=NH<br><br>H,0,-0.0474876739,-0.1281681285,-0.0555576751<br>C,0,-0.0291740412,-0.0465083908,1.024871828<br>C,0,0.0170699424,0.1037518339,3.8049483417<br>C,0,-0.0344085352,-1.2206416834,1.7831357401<br>C,0,-0.0011081343,1.1867085949,1.6531808906<br>C,0,0.0220773038,1.2654403182,3.0438849325<br>C,0,-0.0110733866,-1.133456896,3.1754529458<br>H,0,0.0028349699,2.0946595995,1.0602095654<br>H,0,0.0440300003,2.2335197941,3.5321138422<br>H,0,-0.0150617479,-2.0437409298,3.7677304751<br>H,0,0.0350663721,0.1615948253,4.8874368333<br>C,0,-0.063989135,-2.5427658915,1.1416429596<br>H,0,-0.0653337876,-3.3896145294,1.8419984143<br>H,0,-0.1046136035,-3.6784383921,-0.3699429567<br>N,0,-0.0861474737,-2.6913803885,-0.1194206798	Ph-CH2NH2<br><br>C,0,-0.0399531934,0.0791716512,1.0764204017<br>C,0,0.004558161,0.0047736352,3.8782686864<br>C,0,0.124608206,-1.1272910153,1.7571888373<br>C,0,-0.1930423763,1.2461695307,1.8252581236<br>C,0,-0.1689470691,1.2127644565,3.2136284597<br>C,0,0.1495410681,-1.1669542349,3.1453796938<br>H,0,0.2296015641,-2.0489136307,1.1917058955<br>H,0,-0.3381552174,2.1933210624,1.3133703945<br>H,0,-0.293253318,2.1303066407,3.7785975208<br>H,0,0.2749441556,-2.1152208696,3.6568385859<br>H,0,0.0201729907,-0.0242187706,4.9620525035<br>C,0,-0.0024413612,0.1275199553,-0.4343510864<br>H,0,-0.6475889996,0.9351711923,-0.791056968<br>H,0,-0.415383172,-0.7998527648,-0.8408163132<br>N,0,1.3219779275,0.3217715196,-1.0270687075<br>H,0,1.7312414314,1.1867942597,-0.6920225844<br>H,0,1.946656201,-0.4227682125,-0.7381837767
H2<br><br>H,0,0.,0.,0.372594<br>H,0,0.,0.,-0.372594	
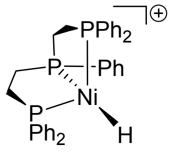<br><br><br>C,0,2.1622727786,-1.6367521574,-2.4069399926<br>H,0,2.3876221995,-0.768749638,-3.0323055003<br>H,0,2.9558348418,-2.3646462377,-2.5932858061<br>C,0,0.7823865625,-2.2047906209,-2.7416070782<br>H,0,0.6861053884,-3.2305107231,-2.3814359645<br>H,0,0.6120745988,-2.2245470162,-3.8204651854<br>C,0,-1.5751144609,-0.2614164702,-3.106948833<br>H,0,-1.48177076,-0.6682213399,-4.1161992335<br>H,0,-2.6168167674,-0.3797721897,-2.8002702163<br>C,0,-1.1876686881,1.2173143021,-3.0718960765<br>H,0,-1.9536941653,1.8203163766,-3.5647835646<br>H,0,-0.2415582089,1.3858280747,-3.593528149<br>C,0,3.9168330039,-0.1865538784,-0.6093626438<br>C,0,5.065351611,-0.9643685731,-0.4512474681<br>H,0,4.9864734338,-2.0336897856,-0.2903674996<br>C,0,6.3175863787,-0.3667477764,-0.4784495558<br>H,0,7.2058252552,-0.9740433393,-0.3475281161<br>C,0,6.4319976086,1.0058229499,-0.6651080146<br>H,0,7.4113375151,1.4700878231,-0.6829676268<br>C,0,5.2913152946,1.7829333382,-0.8194722293<br>H,0,5.377052902,2.8549382629,-0.9549746253<br>C,0,4.0354302784,1.1910693611,-0.7857929806<br>H,0,3.1433468208,1.7999063574,-0.8812616074<br>C,0,2.3547852593,-2.4018714934,0.4694424771<br>C,0,2.1754576183,-2.1546950168,1.8336978565<br>H,0,1.9849238332,-1.1444150648,2.1813044501<br>C,0,2.2538796657,-3.1939690047,2.7489713541<br>H,0,2.1234197321,-2.9913817372,3.8058159347<br>C,0,2.4993088407,-4.4909789902,2.3118655218<br>H,0,2.5585406875,-5.3028792021,3.0274739728<br>C,0,2.6710350008,-4.7447158326,0.957788763<br>H,0,2.86445779,-5.7541649549,0.6134187103<br>C,0,2.6015783859,-3.7047877962,0.0381311115<br>H,0,2.7485083484,-3.9230033559,-1.0133423099<br>C,0,-1.7128050976,-2.5327752368,-1.247563266<br>C,0,-2.6626588869,-3.139610758,-2.0721709475<br>H,0,-2.7672791994,-2.8422547814,-3.1098268462<br>C,0,-3.4854173642,-4.1379674216,-1.5694445088	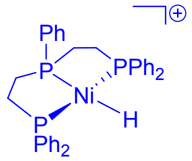<br><br><br>C,0,-0.7407516334,-1.5300679364,2.6700067412<br>H,0,-0.3796305593,-1.9513180863,3.6100992758<br>H,0,-1.7543289223,-1.1652064028,2.8585772081<br>C,0,-0.7551130275,-2.5685803752,1.5436360842<br>H,0,-1.4549199418,-3.3789576034,1.7571998654<br>H,0,0.2361742561,-3.0036794676,1.3916039379<br>C,0,-0.7551130275,-2.5685803752,-1.5436360842<br>H,0,0.2361742561,-3.0036794676,-1.3916039379<br>H,0,-1.4549199418,-3.3789576034,-1.7571998654<br>C,0,-0.7407516334,-1.5300679364,-2.6700067412<br>H,0,-1.7543289223,-1.1652064028,-2.8585772081<br>H,0,-0.3796305593,-1.9513180863,-3.6100992758<br>C,0,1.9869647933,-0.3790407685,2.5661689991<br>C,0,2.3970704242,-1.5601530772,3.1832101373<br>H,0,1.6833752853,-2.3334362093,3.4404220129<br>C,0,3.7387831996,-1.7628407789,3.4859581408<br>H,0,4.0469985283,-2.6846640162,3.9657954452<br>C,0,4.6771776117,-0.7873190093,3.1813254865<br>H,0,5.7219357484,-0.9447437564,3.423106831<br>C,0,4.2739202776,0.3924969568,2.5654652289<br>H,0,5.0035904164,1.1573769107,2.3258270568<br>C,0,2.9395094217,0.5927388363,2.2491523912<br>H,0,2.6322226869,1.5090049916,1.7576403124<br>C,0,-3.0335159878,-1.5280770723,0.<br>C,0,-3.8287003828,-2.677366918,0.<br>H,0,-3.3753452207,-3.663503537,0.<br>C,0,-5.211280246,-2.5675577807,0.<br>H,0,-5.8231933697,-3.4621866608,0.<br>C,0,-5.8106995362,-1.3122519675,0.<br>H,0,-6.8914172287,-1.2297074534,0.<br>C,0,-5.0269434476,-0.1666030818,0.<br>H,0,-5.4930479502,0.8118409393,0.<br>C,0,-3.6415486558,-0.2727891645,0.<br>H,0,-3.0289806706,0.6222047556,0.<br>C,0,1.9869647933,-0.3790407685,-2.5661689991<br>C,0,2.3970704242,-1.5601530772,-3.1832101373<br>H,0,1.6833752853,-2.3334362093,-3.4404220129<br>C,0,3.7387831996,-1.7628407789,-3.4859581408

H,0,-4.2210016591,-4.6040649738,-2.2148519822
C,0,-3.3663174184,-4.5390123833,-0.243891109
H,0,-4.0111652167,-5.3181858915,0.1457921841
C,0,-2.4211197426,-3.9418337975,0.5796998487
H,0,-2.3256768597,-4.2523196916,1.613742732
C,0,-1.5976041185,-2.9402356241,0.0818822989
H,0,-0.8595592134,-2.4795993896,0.7297472055
C,0,-2.5991019481,1.8886892633,-0.5803500418
C,0,-3.5016080168,2.8767520292,-0.9800503344
H,0,-3.2083930679,3.6257857739,-1.7074991548
C,0,-4.7738697016,2.9189297925,-0.4294785684
H,0,-5.4695749836,3.6897247018,-0.740637479
C,0,-5.1527735982,1.9824336991,0.5268067617
H,0,-6.1461283136,2.0220677935,0.9587843327
C,0,-4.2566666407,1.004022515,0.9347545744
H,0,-4.5462034899,0.2790701389,1.6866162231
C,0,-2.9812640212,0.9584557523,0.3853434856
H,0,-2.2784237793,0.2002298572,0.7132772857
C,0,-0.3407611605,3.516178458,-1.4455706355
C,0,-0.4566659203,4.2593582599,-2.6207831361
H,0,-0.8730032883,3.8208019395,-3.5196083519
C,0,-0.0312618854,5.5822428265,-2.6562188841
H,0,-0.1197623347,6.1495034049,-3.5756458493
C,0,0.5009069459,6.1745132638,-1.5194848048
H,0,0.8286773061,7.2072380787,-1.5484567981
C,0,0.6157425361,5.4397064996,-0.3447441358
H,0,1.0333472305,5.897199052,0.5447335604
C,0,0.2048788036,4.1158018071,-0.3088347535
H,0,0.3150133058,3.5422039898,0.6050060419
Ni,0,0.4484040915,0.2457481177,-0.5883114493
P,0,2.2587668369,-0.966189493,-0.6659225826
P,0,-0.5739427028,-1.2424760679,-1.872739028
P,0,-0.919270086,1.782855517,-1.310268812
H,0,1.2023830757,1.2952873275,0.0935750496

H,0,4.0469985283,-2.6846640162,-3.9657954452
C,0,4.6771776117,-0.7873190093,-3.1813254865
H,0,5.7219357484,-0.9447437564,-3.423106831
C,0,4.2739202776,0.3924969568,-2.5654652289
H,0,5.0035904164,1.1573769107,-2.3258270568
C,0,2.9395094217,0.5927388363,-2.2491523912
H,0,2.6322226869,1.5090049916,-1.7576403124
Ni,0,-0.0366951401,0.1975116267,0.
P,0,0.2338881652,-0.0162765411,2.1560733375
P,0,-1.2079200897,-1.643310378,0.
P,0,0.2338881652,-0.0162765411,-2.1560733375
H,0,0.8027604258,1.4166847455,0.
C,0,-0.2876520606,1.2971451842,-3.315952235
C,0,-0.0843191825,1.168442452,-4.6914369121
C,0,-0.9150629128,2.4351812899,-2.8129087384
C,0,-0.5180245782,2.1662976558,-5.5515272675
H,0,0.4261857648,0.2998532151,-5.0932225786
C,0,-1.3466322442,3.4327704205,-3.6783075019
H,0,-1.0477354668,2.5448016491,-1.7421515344
C,0,-1.1509618739,3.2971479861,-5.046048786
H,0,-0.3564395341,2.0652891076,-6.6185514191
H,0,-1.8296288602,4.3189649893,-3.2829786362
H,0,-1.4849819275,4.0766225888,-5.7211629222
C,0,-0.2876520606,1.2971451842,3.315952235
C,0,-0.0843191825,1.168442452,4.6914369121
C,0,-0.9150629128,2.4351812899,2.8129087384
C,0,-0.5180245782,2.1662976558,5.5515272675
H,0,0.4261857648,0.2998532151,5.0932225786
C,0,-1.3466322442,3.4327704205,3.6783075019
H,0,-1.0477354668,2.5448016491,1.7421515344
C,0,-1.1509618739,3.2971479861,5.046048786
H,0,-0.3564395341,2.0652891076,6.6185514191
H,0,-1.8296288602,4.3189649893,3.2829786362
H,0,-1.4849819275,4.0766225888,5.7211629222

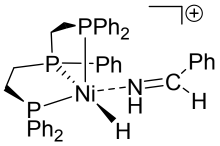

C,0,2.1951866702,1.8651533487,1.6335440586
H,0,2.7562503808,1.0484698848,2.0946637004
H,0,2.7965681026,2.7731204234,1.717900469
C,0,0.8457229584,2.047052393,2.3164344122
H,0,0.375906496,2.9765062289,1.9857009542
H,0,0.9415067203,2.1015523519,3.4035187652
C,0,-0.2220613763,-0.6475623366,3.1338348767
H,0,0.0104290778,-0.2178727862,4.1113274775
H,0,-1.2180322901,-1.0913097359,3.1969882199
C,0,0.7927369592,-1.7108228763,2.7278969677
H,0,0.7053642483,-2.6065889853,3.3475318443
H,0,1.8159173286,-1.3467965108,2.8493988191
C,0,3.6143080322,1.0431498881,-0.7975007388
C,0,4.7676313787,1.1491910133,-0.0211695638
H,0,4.7120750569,1.4321029162,1.0228535499
C,0,6.0161220625,0.8948608233,-0.5792112311
H,0,6.9048933707,0.9802645614,0.0356539465
C,0,6.1249293472,0.5392595494,-1.9157608136
H,0,7.099278557,0.3469877037,-2.3500041797
C,0,4.9789787086,0.4322729672,-2.6967956862
H,0,5.0569631826,0.1558019758,-3.7421111269
C,0,3.7328536084,0.6770673471,-2.1405837441
H,0,2.8442881171,0.5855633326,-2.7564275984
C,0,1.5312062763,3.0008850005,-0.9666425993
C,0,2.3858859087,4.1006836728,-0.8620888118
H,0,3.3148434436,4.0238920774,-0.3070618513
C,0,2.0650470383,5.296663954,-1.4868736074
H,0,2.7329837818,6.146126381,-1.3999288342
C,0,0.8951547274,5.4036305043,-2.2324184203

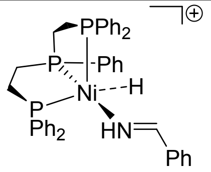

C,0,0.1344728033,2.4110748696,-2.3054003449
H,0,0.3009452502,1.7212902091,-3.136979159
H,0,-0.3017833955,3.3246049562,-2.7155572903
C,0,1.456460488,2.7154383444,-1.6089148342
H,0,1.3280251921,3.4901608366,-0.8485072114
H,0,2.2063271066,3.0758947968,-2.3179849381
C,0,2.7975917008,0.0866713533,-2.0351655394
H,0,3.1365399038,0.6649420385,-2.8981911663
H,0,3.6760426432,-0.3887815238,-1.5932345643
C,0,1.77635845,-0.9721491945,-2.4411144963
H,0,2.2534328776,-1.8001546064,-2.9692960752
H,0,1.0131923574,-0.5532328907,-3.0997386493
C,0,-2.4049636683,0.9739292852,-2.2281453435
C,0,-2.4351840725,1.159911066,-3.6109618069
H,0,-1.6522177213,1.7146103493,-4.1139326987
C,0,-3.4874832617,0.6557533681,-4.3687703933
H,0,-3.4992095592,0.8143231204,-5.4413852391
C,0,-4.5260688649,-0.0299293732,-3.7547318694
H,0,-5.3502282087,-0.4128615402,-4.3455913906
C,0,-4.5070743037,-0.2174132316,-2.3768927855
H,0,-5.3159266015,-0.7498701565,-1.8895271089
C,0,-3.4514487223,0.2714884863,-1.6229500563
H,0,-3.446057939,0.1171859293,-0.5486951793
C,0,-1.8677494318,3.0508945192,-0.3251651776
C,0,-2.8989014794,3.7536876016,-0.9535695632
H,0,-3.2783106559,3.4222131832,-1.9132627041
C,0,-3.4541456054,4.8739815289,-0.3513498032
H,0,-4.2552483966,5.4090369921,-0.8488345242

H,0,0.650367289,6.3380599904,-2.724346045<br>C,0,0.0487142867,4.310564334,-2.3530935964<br>H,0,-0.8570444751,4.3868099782,-2.944249913<br>C,0,0.3658428521,3.1127302574,-1.72134296<br>H,0,-0.2857190857,2.2514675363,-1.8170220478<br>C,0,-1.9863097769,1.4547042563,2.0638649561<br>C,0,-2.8279083137,1.1705507675,3.1400709452<br>H,0,-2.5320035168,0.4646052106,3.9063060056<br>C,0,-4.0589896528,1.8047303487,3.2553458155<br>H,0,-4.700765577,1.5784209448,4.0991095751<br>C,0,-4.4636358524,2.7277660891,2.3005227055<br>H,0,-5.4232580176,3.2225803713,2.395470171<br>C,0,-3.6311323368,3.0198762607,1.2267921584<br>H,0,-3.9376466613,3.7445633069,0.4811124312<br>C,0,-2.4029329774,2.384758078,1.1060448467<br>H,0,-1.7576218145,2.6260802533,0.2673164232<br>C,0,-0.7588416637,-3.296795157,0.7817596248<br>C,0,-1.0947578014,-3.7539473667,-0.4957948095<br>H,0,-0.5223692905,-3.4205051499,-1.3548296919<br>C,0,-2.1422770226,-4.6446415193,-0.6734428542<br>H,0,-2.3843017398,-4.9996928108,-1.6687327227<br>C,0,-2.8814295355,-5.0807061201,0.4208660582<br>H,0,-3.702218263,-5.7747709223,0.2817308873<br>C,0,-2.5601101781,-4.6280326653,1.6924927663<br>H,0,-3.1272974156,-4.969286582,2.5511723444<br>C,0,-1.5027482501,-3.7427974392,1.8734700114<br>H,0,-1.2622165727,-3.4190206228,2.8793422317<br>C,0,2.113511396,-3.1899312857,0.6260663391<br>C,0,2.1677342863,-4.47898048,1.1620254943<br>H,0,1.3192022728,-4.8824272536,1.7035016319<br>C,0,3.2998799935,-5.2608513925,0.9851348472<br>H,0,3.3327761729,-6.2618476535,1.4000852235<br>C,0,4.3855859887,-4.76511193,0.2713704928<br>H,0,5.2676482343,-5.3795220294,0.1309838405<br>C,0,4.3347550502,-3.4869181219,-0.2674983302<br>H,0,5.1748629934,-3.0986221229,-0.8315381857<br>C,0,3.201300542,-2.7012757831,-0.0934007288<br>H,0,3.1592549064,-1.7086441446,-0.5259336293<br>Ni,0,0.3633429818,-0.161362424,-0.1039905323<br>P,0,1.9266230226,1.3914115864,-0.1516742741<br>P,0,-0.3352232463,0.6823406015,1.8331142918<br>P,0,0.6485968732,-2.1162562549,0.9111282839<br>N,0,-1.3199802234,-0.1221346065,-1.2069924866<br>H,0,1.1027029671,-0.7425483085,-1.2106926375<br>C,0,-1.5441632701,-0.4349295865,-2.4284605771<br>C,0,-2.8275999958,-0.4841064852,-3.1243920699<br>C,0,-4.0550976429,-0.2578358612,-2.4895141985<br>C,0,-2.817201584,-0.7675669336,-4.4943801338<br>C,0,-5.2345087097,-0.3103595313,-3.2107040672<br>H,0,-4.0989224028,-0.0481695175,-1.4257387952<br>C,0,-3.999972924,-0.8181173239,-5.2162508127<br>H,0,-1.8708026482,-0.9471872543,-4.9942526964<br>C,0,-5.210274565,-0.5887741618,-4.575277676<br>H,0,-6.1804634265,-0.1375362758,-2.7108604597<br>H,0,-3.9780413461,-1.0369783296,-6.2773030864<br>H,0,-6.1374046319,-0.6291690844,-5.1352930253<br>H,0,-2.1740520481,0.1109959027,-0.7025732805<br>H,0,-0.6719879269,-0.69059045,-3.0295701333	C,0,-2.9887700485,5.3079921235,0.8845785631<br>H,0,-3.4246770657,6.1830892354,1.3528719897<br>C,0,-1.9668015959,4.6141615844,1.517613462<br>H,0,-1.601604572,4.9455422717,2.4833234638<br>C,0,-1.4139282481,3.4881555416,0.9191312714<br>H,0,-0.621392395,2.9424798229,1.4184456699<br>C,0,3.5957533798,1.8457747884,0.1576141355<br>C,0,4.6989702038,2.3012376986,-0.569809325<br>H,0,4.6934249823,2.2827494924,-1.6544050585<br>C,0,5.8190079416,2.7865706001,0.0895967142<br>H,0,6.669510432,3.139498278,-0.4822740421<br>C,0,5.8491882807,2.8214645728,1.4790333653<br>H,0,6.7252860792,3.200780152,1.9923129778<br>C,0,4.7571250788,2.3692808691,2.2070597874<br>H,0,4.7787826586,2.3933476144,3.2905981246<br>C,0,3.6347655377,1.8814618289,1.5494136509<br>H,0,2.7811644957,1.5245933732,2.1144405704<br>C,0,2.202042271,-2.6309966306,-0.1088731659<br>C,0,2.6726974908,-2.2983077361,1.1596686382<br>H,0,2.2596657617,-1.4335416198,1.6676255034<br>C,0,3.6636708775,-3.0642847009,1.7637265575<br>H,0,4.0234238257,-2.799569991,2.7514372233<br>C,0,4.1909899436,-4.1640123103,1.1019468841<br>H,0,4.9645896748,-4.760976538,1.5713213498<br>C,0,3.7253157394,-4.5028559843,-0.1649876114<br>H,0,4.1343009763,-5.3634320849,-0.6820466689<br>C,0,2.7342127325,-3.7432098241,-0.7678900308<br>H,0,2.3666163235,-4.0275470693,-1.7481539898<br>C,0,-0.3491577901,-2.7866898101,-1.4513703645<br>C,0,-0.6401852116,-3.8891758803,-0.6426577786<br>H,0,-0.0630953388,-4.0712894212,0.257237309<br>C,0,-1.6430422698,-4.7797403252,-1.0022285864<br>H,0,-1.8504538899,-5.6373095354,-0.372126019<br>C,0,-2.3658748625,-4.5819413472,-2.1718902998<br>H,0,-3.1413252376,-5.2832487852,-2.4577192411<br>C,0,-2.0872980672,-3.4846197684,-2.9764233158<br>H,0,-2.6474062456,-3.3228967852,-3.8899336113<br>C,0,-1.0927668455,-2.5855999911,-2.6152721053<br>H,0,-0.9071778495,-1.729007895,-3.2519508891<br>Ni,0,0.4415382171,0.287629499,0.2539948981<br>P,0,-1.0629579251,1.5874262989,-1.1242331915<br>P,0,2.1077353415,1.2234612103,-0.7105396286<br>P,0,0.9202758837,-1.575961613,-0.9038433165<br>H,0,0.7847201838,1.4558232068,1.0838876385<br>H,0,-1.1761664196,-1.3105710643,1.3852478339<br>C,0,-0.888652114,-0.1249888894,2.867749586<br>H,0,-0.3488959877,0.7471512151,3.2305151989<br>C,0,-1.8024909434,-0.7502858135,3.8166685998<br>C,0,-1.8710048823,-0.2125186777,5.1066996532<br>C,0,-2.6058954528,-1.8533016025,3.4999757647<br>C,0,-2.7145784877,-0.7627212393,6.0592862824<br>H,0,-1.2549725773,0.6439503517,5.3605626785<br>C,0,-3.4476735588,-2.4003364346,4.4513835128<br>H,0,-2.5836274239,-2.291513266,2.5073616661<br>C,0,-3.5038332883,-1.8575586161,5.7329899231<br>H,0,-2.7575540834,-0.3372965491,7.0548913139<br>H,0,-4.066720686,-3.2529043387,4.1977656036<br>H,0,-4.1656412104,-2.2899730104,6.4744226892<br>N,0,-0.6453376243,-0.4866397983,1.6616140986
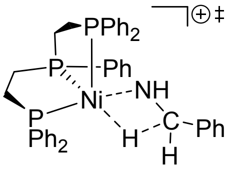<br><br>C,0,1.8324122626,-1.9970068644,-2.0894923206<br>H,0,1.8949738147,-1.2646471571,-2.8984803995<br>H,0,2.6280185046,-2.7251804104,-2.2647608287	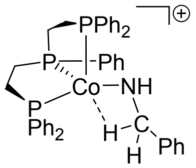<br><br>C,0,1.8405840261,-1.7586397732,-2.0959303188<br>H,0,1.9249870384,-0.9751256621,-2.8535726904<br>H,0,2.6238942194,-2.4862871128,-2.3227966563

C,0,0.4664582462,-2.6739988758,-2.0799070949	C,0,0.4667944868,-2.4209852481,-2.1593208591
H,0,0.4623928116,-3.5543383166,-1.43308105	H,0,0.4262182545,-3.3129589018,-1.5295905515
H,0,0.1819737532,-3.0156935729,-3.0789765223	H,0,0.2308060407,-2.7420653309,-3.1781182044
C,0,-1.390524874,-0.4438072727,-2.7904435829	C,0,-1.2697717179,-0.1031135841,-2.8821055284
H,0,-1.1762458628,-0.9011628249,-3.7590725912	H,0,-1.0332274984,-0.5184122188,-3.8644830934
H,0,-2.4746767176,-0.3388593714,-2.7083637979	H,0,-2.3471778725,0.0746905399,-2.8492417011
C,0,-0.7236902077,0.9227455971,-2.6638065728	C,0,-0.5199321362,1.2046825442,-2.6407219048
H,0,-1.2253566423,1.657438231,-3.2976154705	H,0,-0.9348349128,2.0068114566,-3.2548985834
H,0,0.3279377084,0.8782695812,-2.9574863462	H,0,0.5404593163,1.1049336031,-2.8844076081
C,0,3.581677557,0.0395763954,-1.0034689259	C,0,3.73230493,0.034778465,-0.8841507704
C,0,4.763924613,-0.5166729867,-1.5012351403	C,0,4.882248635,-0.612080483,-1.3457571742
H,0,4.8669183853,-1.5923369668,-1.593737923	H,0,4.8982020243,-1.6914649252,-1.4505136781
C,0,5.8224156442,0.3023808183,-1.863098069	C,0,6.0197684134,0.1186605026,-1.6535168463
H,0,6.7358090505,-0.1362948651,-2.2484426994	H,0,6.90766649,-0.391133466,-2.0099897817
C,0,5.7162425494,1.6828404392,-1.7231934509	C,0,6.0253477397,1.5011541259,-1.4956043996
H,0,6.5475773888,2.3203571933,-2.0017497198	H,0,6.9179579226,2.069317649,-1.7313545729
C,0,4.5497101316,2.2408722223,-1.2206341858	C,0,4.8913419167,2.1492384236,-1.0278650388
H,0,4.4651473013,3.3149074997,-1.1028582995	H,0,4.8942538819,3.2247576961,-0.8936357359
C,0,3.4840951315,1.421957928,-0.8616677444	C,0,3.7477661308,1.4187435969,-0.7219186005
H,0,2.5754063603,1.8617806645,-0.4670162461	H,0,2.8670567107,1.9299490031,-0.3502744836
C,0,2.9352633008,-2.2190886144,0.6371413852	C,0,2.8531019774,-2.2311653822,0.623878189
C,0,3.6282815306,-1.7041023281,1.7384641627	C,0,3.4201412647,-1.8353674357,1.8407881156
H,0,3.7642988998,-0.6321235196,1.8399576786	H,0,3.5111960344,-0.7796277169,2.077202311
C,0,4.1822605657,-2.5539475682,2.6845281467	C,0,3.9038928377,-2.7778679972,2.7357030668
H,0,4.7287653003,-2.1399584247,3.524287172	H,0,4.353481124,-2.454026902,3.6675074119
C,0,4.0436126204,-3.9312809453,2.5538629206	C,0,3.8157304823,-4.1335241561,2.4381005754
H,0,4.4768906426,-4.594767293,3.293087931	H,0,4.191463778,-4.8702589453,3.1384472594
C,0,3.3545655362,-4.4524129442,1.4679812149	C,0,3.249875826,-4.537753525,1.2377098445
H,0,3.2491643098,-5.5253577249,1.3543927329	H,0,3.1833295371,-5.5923547861,0.9954461547
C,0,2.8001906,-3.602909764,0.5165369602	C,0,2.7700738588,-3.5935146255,0.3355679324
H,0,2.2789633992,-4.0380474243,-0.3277648363	H,0,2.3473206404,-3.9381520639,-0.6006409881
C,0,-2.2785090035,-2.7278044782,-1.1708467037	C,0,-2.3550054466,-2.4668903104,-1.5123880383
C,0,-3.0034511228,-3.2009232993,-2.2678796363	C,0,-3.0549298737,-2.7654417351,-2.6843223849
H,0,-2.7702638861,-2.8694089508,-3.2739400668	H,0,-2.7794588064,-2.3118030376,-3.6299529729
C,0,-4.0380406802,-4.1060891105,-2.0788082887	C,0,-4.1197704559,-3.6546621249,-2.6485885663
H,0,-4.5984198508,-4.4687441327,-2.9328751073	H,0,-4.6613050177,-3.8817961481,-3.5596704875
C,0,-4.3531803313,-4.546715041,-0.798373574	C,0,-4.4886702228,-4.2523968283,-1.4490995973
H,0,-5.1613101121,-5.2546260166,-0.6539614084	H,0,-5.3203477186,-4.9472111419,-1.4248274053
C,0,-3.6333034478,-4.0796545339,0.293282053	C,0,-3.792327411,-3.9589499831,-0.2838937977
H,0,-3.8788285213,-4.4218303331,1.2922770227	H,0,-4.0804890509,-4.423985934,0.6520488812
C,0,-2.5979571657,-3.1699730175,0.1133425934	C,0,-2.7280610536,-3.0662583038,-0.3089048463
H,0,-2.0342525462,-2.7942279127,0.9611413889	H,0,-2.1795458147,-2.8216520592,0.5950294361
C,0,-2.5524083284,1.862211124,-0.6330080717	C,0,-2.3824557114,2.1570354093,-0.6218278575
C,0,-3.0821112998,3.0597516197,-1.1205914921	C,0,-2.8416192215,3.3427260813,-1.2020029606
H,0,-2.4361493652,3.7943083061,-1.5883464528	H,0,-2.1601981131,3.9877180021,-1.7463524824
C,0,-4.4384559051,3.3218343431,-0.9961328007	C,0,-4.1711879134,3.7118819412,-1.0674735487
H,0,-4.8426818923,4.2541938811,-1.3735709447	H,0,-4.5225316259,4.633077787,-1.5180388707
C,0,-5.2763465079,2.3945984743,-0.3854322265	C,0,-5.0496662225,2.9071423986,-0.3483751628
H,0,-6.3356539097,2.6034352378,-0.2887214676	H,0,-6.0874262466,3.2013898016,-0.2409566014
C,0,-4.7539445792,1.2065888497,0.1061766491	C,0,-4.5958900509,1.7343158705,0.238013873
H,0,-5.4022615814,0.4852311882,0.5900412306	H,0,-5.2745372575,1.1111399277,0.8084606888
C,0,-3.3946237614,0.9424972659,-0.011617253	C,0,-3.2644215071,1.3582370863,0.1033128305
H,0,-2.9834997653,0.0234404471,0.3900111105	H,0,-2.9080880672,0.4475270231,0.5721440187
C,0,0.0594265765,3.0759391532,-0.812275883	C,0,0.3177792216,3.1746178778,-0.6083856632
C,0,0.5872301611,3.7045037752,-1.9397026085	C,0,0.966746143,3.8273744875,-1.6560102678
H,0,0.5363203479,3.2342814113,-2.9140924753	H,0,0.9460433301,3.4241680521,-2.6611607467
C,0,1.1892062113,4.9531720936,-1.8275680787	C,0,1.6526873095,5.0141371181,-1.4222645705
H,0,1.5942033974,5.4337204212,-2.7108421747	H,0,2.1500846765,5.5159959038,-2.2443253335
C,0,1.2640144581,5.5840843889,-0.5938146759	C,0,1.6947013127,5.5569228664,-0.145681732
H,0,1.7281714575,6.5599961869,-0.5097557393	H,0,2.2261272893,6.48450574,0.0330997614
C,0,0.7378437998,4.9627996608,0.5339226969	C,0,1.0485932236,4.9110652252,0.9030633699
H,0,0.7898722794,5.4536726414,1.4989878505	H,0,1.0743507489,5.3341917848,1.9006396742
C,0,0.1421032745,3.7151427893,0.4275041545	C,0,0.3658330045,3.7261908957,0.6745973533
H,0,-0.2712388506,3.2409782189,1.3118415267	H,0,-0.1468822332,3.2370581663,1.4965358493
Ni,0,0.0138165525,-0.3202662599,0.1932036329	Ni,0,-0.1465821372,-0.2730293639,0.2108757544
P,0,2.1570512957,-1.0212783476,-0.5281410155	P,0,2.1971879983,-0.8999231868,-0.4727959496
P,0,-0.8752140867,-1.5757528717,-1.3965924427	P,0,-0.9142458936,-1.3436857171,-1.5352266906
P,0,-0.77641426,1.4448132442,-0.8783632041	P,0,-0.6364395031,1.6260925973,-0.8363078198
H,0,0.5946353752,0.6155944923,1.2044037408	H,0,0.1806031862,0.4139361304,1.6601762054

H,0,0.5560397886,-2.1682834052,1.7262838642<br>C,0,0.1417855291,-0.3964561962,2.3863526502<br>H,0,1.1628127655,-0.3249138804,2.7757031804<br>C,0,-0.8292021731,0.4486813984,3.1313857419<br>C,0,-0.3865901496,1.6241950751,3.7401037038<br>C,0,-2.1492788375,0.0424143105,3.3138909198<br>C,0,-1.2553830881,2.3940368206,4.4995278673<br>H,0,0.6495782601,1.93098431,3.629057194<br>C,0,-3.0170972361,0.8126656894,4.0741886485<br>H,0,-2.4748739074,-0.8935772517,2.8772925921<br>C,0,-2.5758589654,1.9915893793,4.6636622419<br>H,0,-0.9006860935,3.3016821496,4.9744167556<br>H,0,-4.0402245089,0.4854981226,4.2203709342<br>H,0,-3.254473276,2.5869978622,5.2634948527<br>N,0,-0.2377381487,-1.5328948274,1.7875335082	H,0,0.4440515109,-2.2764020221,1.4804378638<br>C,0,-0.0932167601,-0.5757922347,2.3497130724<br>H,0,0.8603093727,-0.6358638428,2.8888179606<br>C,0,-1.1992247437,-0.0770309566,3.2478796931<br>C,0,-1.0072380065,1.0818283257,3.9995175868<br>C,0,-2.3893904412,-0.7835083601,3.3794326272<br>C,0,-1.9953621474,1.5340660861,4.8616058974<br>H,0,-0.0717140943,1.629385594,3.9221370668<br>C,0,-3.3794314201,-0.3303998869,4.2432527195<br>H,0,-2.5198566905,-1.6970539386,2.8133439129<br>C,0,-3.1877323896,0.8290574063,4.9829136099<br>H,0,-1.8339273876,2.4332062557,5.4453313384<br>H,0,-4.300280655,-0.8933666583,4.3477233449<br>H,0,-3.9585251199,1.1774791893,5.6606487657<br>N,0,-0.3672902957,-1.6621756207,1.508859108
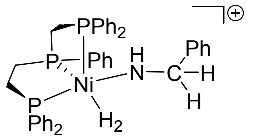<br>C,0,1.6330044057,-1.9386496723,-2.0981447989<br>H,0,2.2672785074,-1.0933767528,-2.3726745366<br>H,0,2.1236749698,-2.8506319563,-2.4443173409<br>C,0,0.2421257963,-1.7981759651,-2.7025417672<br>H,0,-0.3080376304,-2.7374889504,-2.6109231004<br>H,0,0.279596989,-1.5460004015,-3.7651705408<br>C,0,-0.4205427826,1.1051002908,-2.5870160697<br>H,0,-0.4146841437,0.9788771313,-3.6735325747<br>H,0,-1.2719216208,1.7409213546,-2.3330483143<br>C,0,0.8816632582,1.7471077605,-2.1124400964<br>H,0,0.9934437907,2.7345358272,-2.5663341276<br>H,0,1.7456372362,1.1629690859,-2.4370327538<br>C,0,3.1769615734,-2.0124068923,0.4138757291<br>C,0,4.3061875933,-1.8046213012,-0.3771568965<br>H,0,4.2148463729,-1.5902898665,-1.4347995994<br>C,0,5.5759770289,-1.8801620429,0.1831535674<br>H,0,6.4470895581,-1.7255500131,-0.442985381<br>C,0,5.7286904729,-2.1543820516,1.5348844508<br>H,0,6.7202253172,-2.2169307746,1.96816643<br>C,0,4.6063462288,-2.3559578,2.3310887001<br>H,0,4.7194607932,-2.5764062219,3.3863240628<br>C,0,3.3380134787,-2.2860650515,1.7752538674<br>H,0,2.471560028,-2.4670618145,2.4028480053<br>C,0,0.8100959788,-3.618367548,0.1393611211<br>C,0,1.5367601088,-4.7624242898,-0.2014108097<br>H,0,2.5070816414,-4.6751757801,-0.6787515125<br>C,0,1.0318104525,-6.0204387395,0.0900667745<br>H,0,1.6000872886,-6.9039344066,-0.1773201757<br>C,0,-0.1953932353,-6.1483538956,0.7333353264<br>H,0,-0.5840283585,-7.1331545261,0.9659548062<br>C,0,-0.9163125681,-5.015671049,1.0840037343<br>H,0,-1.8677948079,-5.1119674352,1.5944991993<br>C,0,-0.4176348158,-3.7517887968,0.786725508<br>H,0,-0.98403197,-2.8657397621,1.0594545216<br>C,0,-2.4941062422,-0.941800864,-2.2359519468<br>C,0,-3.2379377402,-0.1893559342,-3.1475602319<br>H,0,-2.8189675517,0.6909022948,-3.6189132061<br>C,0,-4.533879762,-0.5688260932,-3.4705021768<br>H,0,-5.102662837,0.0197535544,-4.1809453635<br>C,0,-5.0984141748,-1.6961416951,-2.8885777761<br>H,0,-6.1112207402,-1.9873186567,-3.1414831253<br>C,0,-4.3646823715,-2.4476984229,-1.9792828064<br>H,0,-4.8035989673,-3.3244749369,-1.5177693998<br>C,0,-3.070400456,-2.0730001079,-1.6503200641<br>H,0,-2.5110466436,-2.657203311,-0.9283685392<br>C,0,0.170486583,3.4501987855,0.1544011833<br>C,0,0.3232944679,3.9425563742,1.4543779285<br>H,0,0.942039226,3.4077303117,2.1678186523	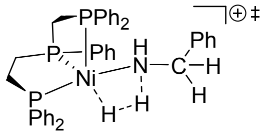<br>C,0,-1.7283131268,-1.8706700583,2.002247623<br>H,0,-2.3480422732,-1.0092947573,2.2600590051<br>H,0,-2.2537072744,-2.7688411596,2.3328889747<br>C,0,-0.3532146185,-1.771679837,2.6510171541<br>H,0,0.1630836326,-2.7321457482,2.5916147917<br>H,0,-0.4171197407,-1.5043043141,3.7085412146<br>C,0,0.5261444528,1.0773038732,2.6170588895<br>H,0,0.5940100105,0.9265355271,3.6980935767<br>H,0,1.3763424138,1.6933247675,2.3138040814<br>C,0,-0.78208111,1.7632191458,2.2306108984<br>H,0,-0.8374861063,2.750869276,2.6927240315<br>H,0,-1.6429064784,1.2010172568,2.5998379255<br>C,0,-3.2147470867,-1.8847132795,-0.5435930673<br>C,0,-4.3613659691,-1.7455299553,0.2367873989<br>H,0,-4.2936832645,-1.6001313876,1.3078528198<br>C,0,-5.6187299268,-1.7993001884,-0.3534483291<br>H,0,-6.5041252087,-1.6961794712,0.2631959476<br>C,0,-5.7407934878,-1.9862744913,-1.7229033122<br>H,0,-6.7226794279,-2.0325112267,-2.1796000938<br>C,0,-4.6002074052,-2.1196492724,-2.5076447096<br>H,0,-4.6895610538,-2.2702432748,-3.5773693157<br>C,0,-3.344246526,-2.0681555098,-1.9229395146<br>H,0,-2.4620590927,-2.1879087298,-2.5434821997<br>C,0,-0.89435181,-3.5444799762,-0.2453197054<br>C,0,-1.5940113515,-4.682269453,0.1649884412<br>H,0,-2.522472568,-4.5879390106,0.7180634409<br>C,0,-1.1182888245,-5.9453630589,-0.1533057054<br>H,0,-1.6651860247,-6.8237529068,0.169728035<br>C,0,0.0503119073,-6.0851479488,-0.89550104<br>H,0,0.4150845065,-7.073863947,-1.1491299563<br>C,0,0.7416412508,-4.9589545998,-1.3193568961<br>H,0,1.6450436808,-5.0633277531,-1.9093704968<br>C,0,0.2728418953,-3.6908953212,-0.9931218674<br>H,0,0.8123888023,-2.8113192441,-1.3282101505<br>C,0,2.4352154029,-1.1077697364,2.1166364194<br>C,0,3.3384194762,-0.3449490299,2.8578108237<br>H,0,3.058117115,0.6222501084,3.2554838175<br>C,0,4.6172578832,-0.82665077,3.1085820114<br>H,0,5.3095409784,-0.228709645,3.6898815268<br>C,0,5.0068747737,-2.0659133695,2.6204102244<br>H,0,6.0059396786,-2.4370652768,2.8167042536<br>C,0,4.1136378638,-2.8295010629,1.8780696876<br>H,0,4.4135374273,-3.79665507,1.4918689894<br>C,0,2.8363041556,-2.3537907241,1.6238011451<br>H,0,2.1512607032,-2.9570843469,1.0365603192<br>C,0,-0.1734196068,3.4592108862,-0.0802328927<br>C,0,-0.3310248005,3.9014590091,-1.3966307419<br>H,0,-0.9297032514,3.3259721418,-2.0954835288

C,0,-0.2958041759,5.1202072292,1.8417892518<br>H,0,-0.1602782174,5.4919797224,2.8509714938<br>C,0,-1.090108152,5.8215736451,0.9406302887<br>H,0,-1.5772666007,6.7407111896,1.2445650807<br>C,0,-1.2510102279,5.3407688267,-0.3503767831<br>H,0,-1.8619848513,5.8852240313,-1.0616360981<br>C,0,-0.6234091799,4.1625669595,-0.7428977352<br>H,0,-0.7569633012,3.8214569022,-1.7625846779<br>C,0,2.7892820512,2.3703519543,-0.0520238204<br>C,0,3.3101538667,3.4819921118,-0.7208641186<br>H,0,2.6794498101,4.070583853,-1.3780868211<br>C,0,4.6303030575,3.8597888502,-0.530095639<br>H,0,5.024747659,4.7235099277,-1.0533332481<br>C,0,5.4421932353,3.1422800734,0.3430967978<br>H,0,6.4711601298,3.4458256378,0.4988985122<br>C,0,4.9299529653,2.0457872095,1.0207475769<br>H,0,5.5553194562,1.4885915575,1.7083871841<br>C,0,3.6084962336,1.6592271915,0.8221158283<br>H,0,3.2173737928,0.7992129012,1.3535266996<br>Ni,0,-0.0998356901,-0.4063448587,0.2809424628<br>P,0,1.4685315643,-1.9433383128,-0.2505367639<br>P,0,-0.7719076704,-0.5285729766,-1.7878254016<br>P,0,1.0252842439,1.8638601538,-0.252133937<br>N,0,-1.850839943,-0.0162670823,0.8245695498<br>H,0,-2.5767575565,-0.0225054253,0.1149047632<br>H,0,0.1005650305,-0.7637658905,1.9935199902<br>H,0,0.5135014801,-0.0994775852,1.9100332891<br>C,0,-2.114075087,1.1081611483,1.7160839628<br>H,0,-1.4752104483,1.0058766028,2.6027428126<br>H,0,-1.8714425309,2.0833125568,1.2762646654<br>C,0,-3.5647296576,1.1175977191,2.134656651<br>C,0,-4.0805995633,0.0812781589,2.9147316579<br>C,0,-4.4177905853,2.1439481067,1.7349819931<br>C,0,-5.4155020252,0.077039539,3.2925081865<br>H,0,-3.4249592265,-0.7242229905,3.2281818202<br>C,0,-5.7561863257,2.1423175841,2.1110339304<br>H,0,-4.0271473217,2.9587179029,1.1331724247<br>C,0,-6.2576264804,1.1085794085,2.8905091081<br>H,0,-5.8013212863,-0.7283969494,3.9077042549<br>H,0,-6.4064361162,2.9520122113,1.7988974143<br>H,0,-7.2998720814,1.1077712135,3.1889308567	C,0,0.25633551,5.0840666152,-1.8191162693<br>H,0,0.1174457056,5.4179610024,-2.8410227895<br>C,0,1.0227912108,5.8375917615,-0.9370677575<br>H,0,1.4855417842,6.7598713378,-1.2682372273<br>C,0,1.1898786708,5.4037465499,0.3698905353<br>H,0,1.7824431003,5.9874912454,1.0652774535<br>C,0,0.5954853353,4.2211409905,0.7971095856<br>H,0,0.7382539796,3.9139438124,1.8266419721<br>C,0,-2.7633331529,2.3160879427,0.1851746061<br>C,0,-3.3745965524,3.2703166893,1.003225104<br>H,0,-2.8161607941,3.7549198072,1.796306292<br>C,0,-4.6988064919,3.6256571674,0.7954508763<br>H,0,-5.164745994,4.3657534399,1.4361678743<br>C,0,-5.4219820032,3.0476797779,-0.2430838949<br>H,0,-6.4536548215,3.3355000286,-0.4102942319<br>C,0,-4.8186409244,2.1093944477,-1.067791569<br>H,0,-5.3758621627,1.6593802315,-1.8810443777<br>C,0,-3.4954697887,1.7406738182,-0.8513958851<br>H,0,-3.0330632434,0.999692127,-1.4933721999<br>Ni,0,0.0358084217,-0.2995913635,-0.2921055223<br>P,0,-1.520292285,-1.8596721087,0.1581341892<br>P,0,0.7339368294,-0.5486163351,1.7525534152<br>P,0,-0.9867493695,1.8647403359,0.3766292727<br>N,0,1.8327830826,-0.0132768458,-1.01334926<br>H,0,2.4639928491,-0.668811334,-0.5670185916<br>H,0,0.4789719269,-0.3111096269,-1.8473408646<br>H,0,-0.4014416169,-0.3293323231,-1.8866434239<br>C,0,2.4351493841,1.3076575568,-1.0841138462<br>H,0,1.7656122925,1.9629212264,-1.6485586891<br>H,0,2.5742345004,1.7872648725,-0.1029278653<br>C,0,3.779062775,1.2392844976,-1.7728554308<br>C,0,3.8686520531,0.8599046608,-3.1131813469<br>C,0,4.9506769466,1.5401782641,-1.0832925205<br>C,0,5.0994720042,0.7928754336,-3.7487398614<br>H,0,2.96311033,0.6163632632,-3.6586747596<br>C,0,6.1867727247,1.4749906547,-1.7171199353<br>H,0,4.8953754349,1.8375066442,-0.0405155698<br>C,0,6.2634577642,1.1011267672,-3.0514940605<br>H,0,5.1538825229,0.5053833203,-4.7929443324<br>H,0,7.089620647,1.7188450862,-1.1682205944<br>H,0,7.2252558187,1.0518574822,-3.5492644439
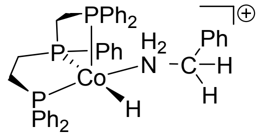<br><br>C,0,2.2976617516,1.721769575,1.6536948567<br>H,0,2.8403012736,0.8317353044,1.9818181156<br>H,0,2.9658740595,2.5796570285,1.7589472452<br>C,0,1.0346243245,1.9269741,2.4809081147<br>H,0,0.6420370438,2.9335823341,2.3240784576<br>H,0,1.2306570743,1.8256613338,3.5510374667<br>C,0,-0.3525087873,-0.6358004685,3.2092223549<br>H,0,-0.1430200093,-0.2608783311,4.2141877735<br>H,0,-1.3736157713,-1.0222536402,3.2102743324<br>C,0,0.6133155902,-1.7411273444,2.8026102147<br>H,0,0.4417973186,-2.6554578521,3.3760472585<br>H,0,1.6493095028,-1.4462071597,2.9870993509<br>C,0,3.4453253964,1.0608432964,-0.9601198754<br>C,0,4.6777414371,1.1547569463,-0.3142078233<br>H,0,4.7381743164,1.4253015453,0.7328858728<br>C,0,5.8557680624,0.8983788812,-1.0078253003<br>H,0,6.807144341,0.9724083413,-0.4933283559<br>C,0,5.8149098363,0.554588397,-2.351448881<br>H,0,6.7344455683,0.3605517691,-2.8915369958<br>C,0,4.5897388544,0.4597844594,-3.0027460177<br>H,0,4.5507330214,0.1905752115,-4.0520887896	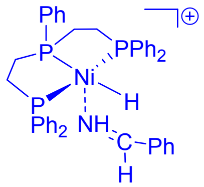<br><br>C,0,1.0341357918,-2.6713372931,1.7599582358<br>H,0,0.6042154717,-3.6262660562,2.0669034702<br>H,0,2.1115114933,-2.8213494535,1.6583399471<br>C,0,0.7640180245,-1.5715214661,2.7969659792<br>H,0,1.3293370816,-1.7480377951,3.7142980005<br>H,0,-0.2969238053,-1.5144395724,3.0527072452<br>C,0,0.764787641,1.5820981882,2.7902530989<br>H,0,-0.2961355903,1.526578582,3.0464196695<br>H,0,1.330339249,1.7622859993,3.7067280967<br>C,0,1.0352277143,2.6773293194,1.7484957435<br>H,0,2.1126573356,2.8263460018,1.6459748342<br>H,0,0.6058873686,3.633792567,2.0514524067<br>C,0,-1.1058726249,-3.1581287375,-0.2176271876<br>C,0,-1.8977984292,-3.5826849385,0.8480697267<br>H,0,-1.6048206209,-3.3790976799,1.8718481654<br>C,0,-3.0746130104,-4.2841289309,0.6148373081<br>H,0,-3.6764986165,-4.617985168,1.4524322821<br>C,0,-3.4743297576,-4.5620666657,-0.6851228656<br>H,0,-4.3892260008,-5.1141675993,-0.866421011<br>C,0,-2.6943555221,-4.1332404863,-1.7527434516

C,0,3.4136065507,0.7056355742,-2.3107680332
H,0,2.4622217606,0.6195274259,-2.825160426
C,0,1.4628629223,3.1155491898,-0.7717227891
C,0,2.3952529156,4.1527103862,-0.6957140443
H,0,3.3749076686,3.9788994617,-0.2636655431
C,0,2.0859072023,5.4099590384,-1.1932086491
H,0,2.8155970448,6.2091227902,-1.1291571311
C,0,0.8478632345,5.6431862331,-1.783530732
H,0,0.6119795651,6.6250000779,-2.1776796112
C,0,-0.07984334,4.6151220536,-1.8747964368
H,0,-1.0415724774,4.7910952701,-2.3434658421
C,0,0.2283123717,3.356533496,-1.3697744675
H,0,-0.4919242694,2.5486296793,-1.437757834
C,0,-1.8461593271,1.7382379855,2.361126523
C,0,-2.8200618666,1.3227417523,3.2694879826
H,0,-2.7175366278,0.3922581909,3.8129633817
C,0,-3.9412073698,2.1087370434,3.5107496866
H,0,-4.6848440927,1.7736759336,4.2247283121
C,0,-4.1059250161,3.3165907108,2.8487430649
H,0,-4.9798091388,3.9282403225,3.0403379
C,0,-3.1419304569,3.7394203081,1.9411077307
H,0,-3.2603586253,4.6831460573,1.4210592391
C,0,-2.0243771074,2.9556196641,1.6961972119
H,0,-1.2861666167,3.3006101127,0.9795844235
C,0,-0.8492107699,-3.2519519203,0.7139948186
C,0,-1.0150807411,-3.7826444822,-0.5701031292
H,0,-0.3045470153,-3.53563738,-1.353388273
C,0,-2.0574350808,-4.6561648682,-0.8440154662
H,0,-2.164261689,-5.0708648955,-1.839951044
C,0,-2.9578546628,-5.0042082782,0.1572361343
H,0,-3.7707394164,-5.6887879743,-0.0554504859
C,0,-2.8037049747,-4.4803620229,1.4331001664
H,0,-3.4954102666,-4.7560847402,2.221152147
C,0,-1.7563166905,-3.60828015,1.710967356
H,0,-1.6490814151,-3.227602379,2.7199397987
C,0,2.0185279469,-3.1187174934,0.6906569345
C,0,2.0960904708,-4.3925736815,1.2603217739
H,0,1.2590106899,-4.7935077505,1.8215560461
C,0,3.2369780536,-5.163151826,1.0930248362
H,0,3.2885131993,-6.1519005222,1.534506354
C,0,4.3085949821,-4.6714611866,0.3549184568
H,0,5.197712804,-5.2772455946,0.2220050911
C,0,4.2347225962,-3.409227474,-0.2171647458
H,0,5.0635992528,-3.0247389519,-0.8000522588
C,0,3.0925531655,-2.6339102294,-0.0510919316
H,0,3.0329554081,-1.6532855461,-0.5082654741
Ni,0,0.2627233046,-0.0979513235,-0.0069834236
P,0,1.8429651869,1.4242150049,-0.1303611121
P,0,-0.3363356665,0.7565554084,1.9710530817
P,0,0.5376771023,-2.0655238734,0.9658075713
N,0,-1.4559200518,-0.2181057926,-1.2514536587
H,0,-1.1926683693,0.1402176589,-2.1646840419
H,0,-1.5772598259,-1.2180724796,-1.3928796664
H,0,0.9884716653,-0.71237284,-1.1043829978
C,0,-2.7788251898,0.3498644542,-0.8899851482
H,0,-3.0733581602,-0.0926891392,0.0625564101
H,0,-2.6472871428,1.4182248737,-0.7151632233
C,0,-3.836988768,0.1117717849,-1.9343176489
C,0,-4.6144233657,-1.0460794358,-1.907738678
C,0,-4.0307484967,1.0259642959,-2.9698588798
C,0,-5.5570382418,-1.2888840079,-2.8981018801
H,0,-4.4873895944,-1.7606393797,-1.0999736972
C,0,-4.9717427091,0.7856391276,-3.9624041706
H,0,-3.4481206432,1.9423909643,-2.9953060843
C,0,-5.7359367047,-0.374161288,-3.9286406645
H,0,-6.1595997743,-2.1894189675,-2.861582987
H,0,-5.1156366938,1.5078648278,-4.7579956664
H,0,-6.4749268167,-0.5607600107,-4.6992958311
H,0,-2.9982435253,-4.3514119086,-2.7702894717
C,0,-1.5195922084,-3.4315167497,-1.5224206029
H,0,-0.9143093607,-3.1094689062,-2.3625457616
C,0,3.0699035044,0.0030576719,2.0019117229
C,0,3.7385644962,0.0053179505,3.2298420425
H,0,3.1824969228,0.0072389377,4.1621321697
C,0,5.1248777791,0.0051349732,3.270152507
H,0,5.6370339058,0.0068921256,4.225532026
C,0,5.8555915326,0.0027037623,2.0862511697
H,0,6.9390402504,0.002567968,2.1199294943
C,0,5.1979902471,0.0004537908,0.8642211963
H,0,5.7652866462,-0.0014454918,-0.0592600757
C,0,3.8086117644,0.0006233574,0.8206117579
H,0,3.2914812953,-0.0011475019,-0.1319494781
C,0,-1.1053766816,3.156127862,-0.2304115115
C,0,-1.8966260511,3.5857558823,0.8337543286
H,0,-1.6030809518,3.3869172698,1.8583032314
C,0,-3.0734724478,4.286284984,0.5979337559
H,0,-3.6748291434,4.624128397,1.4343096175
C,0,-3.4738861611,4.5582430871,-0.7030749899
H,0,-4.3888065774,5.1096288583,-0.8864150645
C,0,-2.6945761498,4.1243562832,-1.7691354588
H,0,-2.9990095857,4.3378487676,-2.7875102646
C,0,-1.5197926995,3.4235253553,-1.5362233968
H,0,-0.9150427752,3.0974701545,-2.3751868067
Ni,0,0.1934647313,-0.0004928764,0.0410051819
P,0,0.4319116006,-2.179859814,0.0368440392
P,0,1.2359759479,0.0033522854,1.9410871594
P,0,0.4323777012,2.178765467,0.0276508615
H,0,-0.1423886368,-0.0034406617,-1.4116339746
C,0,1.6310198244,2.9808085874,-1.1069705585
C,0,2.0045901022,4.3162723211,-0.9366963191
C,0,2.1630642765,2.2498671421,-2.165881082
C,0,2.9093156116,4.9024911429,-1.8092581369
H,0,1.5830424344,4.9076267352,-0.1310580678
C,0,3.063366904,2.8424189518,-3.0438331023
H,0,1.8565965368,1.2184337115,-2.2988727849
C,0,3.4398982428,4.1660968754,-2.8635830868
H,0,3.1965873845,5.9386158372,-1.6713121165
H,0,3.4695399855,2.2692667989,-3.8695873189
H,0,4.1432947791,4.6287439196,-3.5464478529
C,0,1.6307977261,-2.9867823687,-1.094056445
C,0,2.0044255112,-4.3214666201,-0.9179063089
C,0,2.1629311965,-2.2604431852,-2.1560871103
C,0,2.9092940248,-4.9114387846,-1.787787184
H,0,1.5828167109,-4.9093224717,-0.1097437731
C,0,3.0633718157,-2.8567733525,-3.0313345698
H,0,1.856434462,-1.2296129425,-2.2936136056
C,0,3.4399598092,-4.179628811,-2.8452566516
H,0,3.1966124241,-5.9469326774,-1.645277063
H,0,3.4696144078,-2.2872095769,-3.859533895
H,0,4.1434686218,-4.6452128938,-3.5260062955
N,0,-1.9122549919,0.0012104486,0.6940088513
C,0,-4.3275029049,-0.000154215,0.1705807918
C,0,-4.8446624109,0.0034007656,1.4705541161
C,0,-5.215441124,-0.0029442008,-0.9087009115
C,0,-6.2120850635,0.0041616982,1.6812192266
H,0,-4.1811643816,0.0055817146,2.3295976121
C,0,-6.5863526649,-0.0021693943,-0.6984146805
H,0,-4.8221244455,-0.0057294298,-1.9198747465
C,0,-7.0862545743,0.0013857023,0.5968869322
H,0,-6.6038617674,0.0069144387,2.6917800528
H,0,-7.2644077601,-0.0043436302,-1.5437537648
H,0,-8.1569520432,0.0019927911,0.7659420808
C,0,-2.8928744389,-0.0011358189,-0.1234925073
H,0,-2.2265752188,0.0041566898,1.663915667
H,0,-2.6295805645,-0.0042739942,-1.1815714897

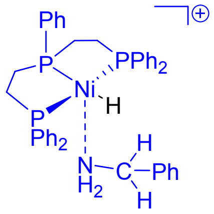

C,0,0.9819345617,-2.6185465117,1.7153925831
H,0,0.5874674584,-3.5944816613,2.001365996
H,0,2.0712486672,-2.7052189902,1.7273697019
C,0,0.5409164139,-1.5207171648,2.6940298714
H,0,0.9888310265,-1.6685771786,3.6786657161
H,0,-0.5448674923,-1.4947753535,2.8061700111
C,0,0.4740065803,1.6391426319,2.6366382925
H,0,-0.6097977683,1.5702810348,2.7526421584
H,0,0.9156770988,1.8416424089,3.6144026491
C,0,0.8650047261,2.7183832801,1.6170709702
H,0,1.9493641256,2.8539192901,1.6231734038
H,0,0.4253624347,3.6859182333,1.8641560633
C,0,-0.9598911763,-3.1437046382,-0.4845426801
C,0,-1.6687106188,-3.9132537315,0.4364371674
H,0,-1.3598182877,-3.9677362277,1.4734038832
C,0,-2.7857356856,-4.6391404518,0.0349449239
H,0,-3.32348826,-5.2394940733,0.7598934592
C,0,-3.2062408192,-4.59945159,-1.2867634408
H,0,-4.0712574295,-5.1727398518,-1.5996358144
C,0,-2.5114579164,-3.82381434,-2.2091333616
H,0,-2.8346777521,-3.7880230507,-3.2432207703
C,0,-1.4005707488,-3.0951427166,-1.8107983024
H,0,-0.8623158028,-2.4951216188,-2.5369869994
C,0,2.888928659,0.1009323974,2.1675417407
C,0,3.401437196,0.1291291848,3.4680699527
H,0,2.7350923028,0.1247028289,4.3250091177
C,0,4.7719723784,0.1630209123,3.6781604657
H,0,5.1624897442,0.1846803535,4.6891158856
C,0,5.6426244667,0.1691636934,2.593132228
H,0,6.7133944331,0.1955932503,2.7595537205
C,0,5.1406497905,0.1414797458,1.2996030792
H,0,5.8172661813,0.1461800237,0.4529521612
C,0,3.7676027986,0.1074084453,1.0858255409
H,0,3.3728638242,0.0860317065,0.0764187639
C,0,-1.1177410534,3.0639021852,-0.5671047535
C,0,-1.9146223518,3.7106079571,0.3766739178
H,0,-1.6278576193,3.7398330487,1.4212274368
C,0,-3.0931155786,4.3394578342,-0.0113391098
H,0,-3.7005691163,4.84517729,0.7304586273
C,0,-3.4869776821,4.3245326281,-1.3414213505
H,0,-4.4037568254,4.8178089753,-1.6424935485
C,0,-2.702044174,3.6732014759,-2.2868344283
H,0,-3.0050049607,3.657534212,-3.3274688702
C,0,-1.5290838381,3.0409425808,-1.9032371696
H,0,-0.9235876446,2.5340100617,-2.6468263433
Ni,0,0.3221548014,0.0030245735,-0.1468163523
P,0,0.5509247535,-2.1740809828,-0.0642221018
P,0,1.0767711723,0.056769998,1.8834291213
P,0,0.4477067657,2.1907008572,-0.1433258339
H,0,0.1060688827,-0.025605147,-1.6218475286
C,0,1.6993986252,3.0481249547,-1.1732655961
C,0,1.9415735178,4.4157215614,-1.0237456304
C,0,2.411532098,2.323746928,-2.1256398976
C,0,2.8951486828,5.0425382346,-1.8118041057
H,0,1.3796934794,4.999286943,-0.3024159751
C,0,3.3619028359,2.9564620787,-2.9183913348
H,0,2.204573536,1.2663389784,-2.2463816236
C,0,3.6066266598,4.313390799,-2.7592354777
H,0,3.0797231241,6.1039668185,-1.6921427909
H,0,3.9092952533,2.3889075379,-3.6623686623

H,0,4.3479216042,4.8075359926,-3.376766131<br>C,0,1.8559321262,-3.0005786238,-1.0545667566<br>C,0,2.2037879341,-4.333384693,-0.8229650247<br>C,0,2.4964267347,-2.288925092,-2.065682602<br>C,0,3.1899201597,-4.9374433333,-1.588528156<br>H,0,1.7004650577,-4.9095115665,-0.05400543<br>C,0,3.4791167461,-2.899585416,-2.8359941642<br>H,0,2.2089869321,-1.259698182,-2.2491905745<br>C,0,3.8288638365,-4.2210572761,-2.5953853196<br>H,0,3.4566290867,-5.9718582903,-1.4044390908<br>H,0,3.9703949064,-2.3423072337,-3.6255328671<br>H,0,4.5958681068,-4.697731041,-3.1949776136<br>N,0,-2.0795039819,-0.0384400273,0.164724153<br>H,0,-2.3411749629,0.8639307192,-0.2219956063<br>H,0,-2.2764785973,-0.7149196222,-0.5660419527<br>C,0,-2.9341782301,-0.3254894652,1.3319269078<br>H,0,-2.7108485582,-1.3447538484,1.6575124234<br>H,0,-2.6389172927,0.3434631605,2.1444665228<br>C,0,-4.416775416,-0.1808904428,1.088317015<br>C,0,-5.0999620572,-1.110380445,0.302546564<br>C,0,-5.1271007708,0.8889274477,1.6280611332<br>C,0,-6.4577737759,-0.9666290342,0.0558266931<br>H,0,-4.5672785522,-1.960463167,-0.1134046499<br>C,0,-6.4880316839,1.0341213147,1.3859775174<br>H,0,-4.6127686439,1.6146064518,2.250803757<br>C,0,-7.1557119616,0.1073724867,0.5972117538<br>H,0,-6.9769565843,-1.6987010755,-0.5525404023<br>H,0,-7.0279951274,1.8691874043,1.8180118401<br>H,0,-8.2175865884,0.2166313451,0.4090403043	

S8. NMR Data

(4-methoxyphenyl)methanamine hydrochloride

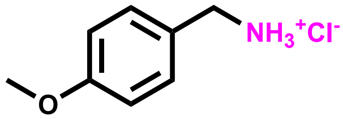

^{1}H NMR (400 MHz, DMSO-d_6) δ 8.56 (br s, 3H), 7.45 (d, J = 9.0 Hz, 2H), 6.95 (d, J = 8.7 Hz, 2H), 3.92 (s, 2H), 3.75 (s, 3H). **^{13}C NMR (101 MHz, DMSO-d_6)** δ 159.74 , 131.03 , 126.47 , 114.31 , 55.65 , 42.04 . **White solid.**

(3,4,5-trimethoxyphenyl)methanamine hydrochloride

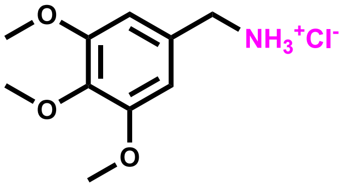

^{1}H NMR (400 MHz, DMSO-d_6) δ 8.67 (br s, 3H), 6.97 (s, 2H), 3.94 (s, 2H), 3.78 (s, 6H), 3.64 (s, 3H). **^{13}C NMR (101 MHz, DMSO-d_6)** δ 153.22 , 137.70 , 130.08 , 107.03 , 60.46 , 56.47 , 42.83 . **Off white solid.**

p-tolylmethanamine hydrochloride

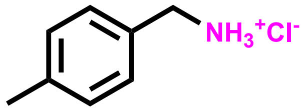

^{1}H NMR (400 MHz, DMSO-d_6) δ 8.63 (br s, 3H), 7.40 (d, J = 8.0 Hz, 2H), 7.20 (d, J = 7.8 Hz, 2H), 3.94 (s, 2H), 2.30 (s, 3H). **^{13}C NMR (101 MHz, DMSO-d_6)** δ 138.10 , 131.54 , 129.46 , 129.44 , 42.30 , 21.22 . **White solid.**

(4-(tert-butyl)phenyl)methanamine hydrochloride

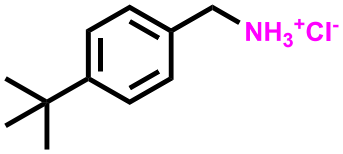

^{1}H NMR (400 MHz, DMSO-d_6) δ 8.60 (br s, 3H), 7.54 – 7.14 (m, 4H), 3.95 (s, 2H), 1.27 (s, 9H). **^{13}C NMR (101 MHz, DMSO-d_6)** δ 151.33 , 131.63 , 129.25 , 125.72 , 42.23 , 34.79 , 31.54 . **White solid.**

(4-chlorophenyl)methanamine hydrochloride

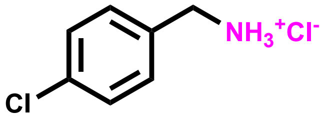

^{1}H NMR (400 MHz, DMSO-d_6) δ 8.70 (br s, 3H), 7.57 (d, J = 8.5 Hz, 2H), 7.47 (d, J = 8.4 Hz, 2H), 4.00 (s, 2H). **^{13}C NMR (101 MHz, DMSO-d_6)** δ 133.59 , 133.51 , 131.48 , 128.90 , 41.81 . **White solid.**

(4-(trifluoromethyl)phenyl)methanamine hydrochloride

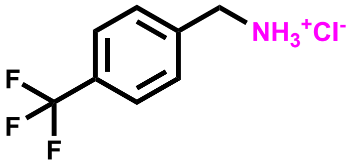

^{1}H NMR (400 MHz, DMSO-d_6) δ 8.81 (br s, 3H), 7.95 – 7.53 (m, 4H), 4.12 (s, 2H). **^{13}C NMR (101 MHz, DMSO-d_6)** δ 139.30 , 130.27 , 129.26 (q, J = 31.8 Hz), 125.74 (q, J = 3.7 Hz), 124.58 (q, J = 272.2 Hz), 41.99. **White solid.**

(4-(trifluoromethoxy)phenyl)methanamine hydrochloride

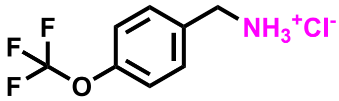

^{1}H NMR (400 MHz, DMSO-d_6) δ 8.70 (br s, 3H), 7.69 (d, J = 8.7 Hz, 2H), 7.41 (d, J = 7.8 Hz, 2H), 4.07 (s, 2H). **^{13}C NMR (101 MHz, DMSO-d_6)** δ 148.70 , 134.06 , 131.67 , 121.52 , 120.48 (q, J = 256.3 Hz), 41.77 . **Off white solid.**

(3-hydroxy-4-methoxyphenyl)methanamine hydrochloride

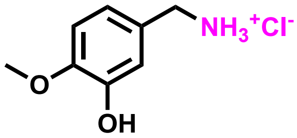

^{1}H NMR (400 MHz, DMSO-d_6) δ 8.49 (br s, 3H), 7.22 – 6.82 (m, 3H), 3.90 (s, 2H), 3.81 (s, 3H). **^{13}C NMR (101 MHz, DMSO-d_6)** δ 148.27 , 146.87 , 126.82 , 120.45 , 116.84 , 112.51 , 56.15 , 42.32 . **Brown solid.**

(4-(methylthio)phenyl)methanamine hydrochloride

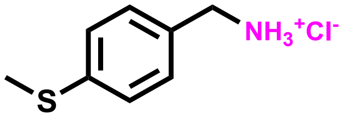

^{1}H NMR (400 MHz, DMSO-d_6) δ 7.99 (br s, 3H), 7.46 (d, J = 8.3 Hz, 2H), 7.26 (d, J = 8.3 Hz, 2H), 3.95 (s, 2H), 2.46 (s, 3H). **^{13}C NMR (101 MHz, DMSO-d_6)** δ 138.95 , 131.05 , 130.18 , 126.22 , 42.10 , 15.14 . **Brown solid.**

benzo[d][1,3]dioxol-5-ylmethanamine hydrochloride

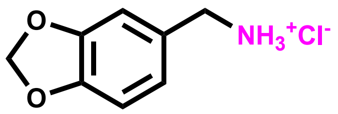

^{1}H NMR (400 MHz, DMSO-d_6) δ 8.58 (br s, 3H), 7.16 (d, J = 1.7 Hz, 1H), 6.98 (dd, J = 8.0, 1.7 Hz, 1H), 6.93 (dd, J = 7.9, 0.4 Hz, 1H), 6.03 (s, 2H), 3.90 (s, 2H). **^{13}C NMR (101 MHz, DMSO-d_6)** δ 147.69 , 147.67 , 128.17 , 123.35 , 109.99 , 108.65 , 101.63 , 42.37 . **Off white solid.**

(2,3-dihydrobenzo[b][1,4]dioxin-6-yl)methanamine hydrochloride

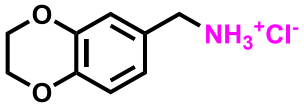

^{1}H NMR (400 MHz, DMSO-d_6) δ 8.29 (br s, 3H), 7.07 (d, J = 2.1 Hz, 1H), 6.96 (dd, J = 8.3, 2.1 Hz, 1H), 6.85 (d, J = 8.3 Hz, 1H), 4.23 (s, 4H), 3.86 (s, 2H). **^{13}C NMR (101 MHz, DMSO-d_6)** δ 143.91 , 143.57 , 127.44 , 122.54 , 118.40 , 117.45 , 64.56 , 64.52 , 42.05 . **Off white solid.**

(benzo[d][1,3]dioxol-5-yl)-2-methylpropan-1-amine hydrochloride

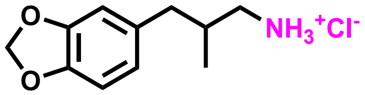

^{1}H NMR (400 MHz, DMSO-d_6) δ 8.30 (br s, 3H), 6.81 – 6.78 (m, 2H), 6.63 (dd, J = 8.0, 1.6 Hz, 1H), 5.95 (s, 2H), 2.73 – 2.63 (m, 2H), 2.63 – 2.54 (m, 1H), 2.28 (dd, J = 13.5, 8.3 Hz, 1H), 2.08 – 1.96 (m, 1H), 0.85 (d, J = 6.6 Hz, 3H). **^{13}C NMR (101 MHz, DMSO-d_6)** δ 147.58 , 145.86 , 133.85 , 122.38 , 109.74 , 108.43 , 101.13 , 44.25 , 39.72 , 33.72 , 17.30 . **Pale brown solid**.

(4-(4,4,5,5-tetramethyl-1,3,2-dioxaborolan-2-yl)phenyl)methanamine hydrochloride

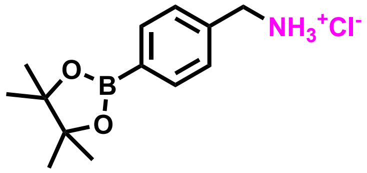

^{1}H NMR (400 MHz, DMSO-d_6) δ 8.61 (br s, 3H), 7.69 (d, J = 8.0 Hz, 2H), 7.52 (d, J = 8.1 Hz, 2H), 4.02 (s, 2H), 1.30 (s, 12H). **^{13}C NMR (101 MHz, DMSO-d_6)** δ 137.85 , 134.96 , 129.42 , 128.77 , 84.22 , 42.50 , 25.13 . **Off white solid.**

(E)-(4-styrylphenyl)methanamine hydrochloride

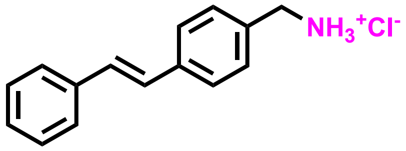

^{1}H NMR (300 MHz, DMSO-d_6) δ 8.61 (br s, 3H), 7.69 – 7.56 (m, 4H), 7.52 (d, J = 8.0 Hz, 2H), 7.45 – 7.32 (m, 3H), 7.32 – 7.20 (m, 2H), 4.02 (s, 2H). **^{13}C NMR (75 MHz, DMSO-d_6)** δ 137.59 , 137.32 , 133.76 , 129.86 , 129.46 , 129.40 , 129.20 , 128.26 , 128.22 , 127.00 , 42.37 . **Yellow solid.**

(4-(benzyloxy)phenyl)methanamine hydrochloride

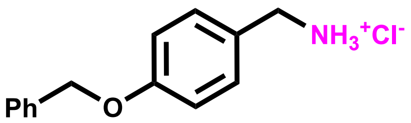

^{1}H NMR (400 MHz, DMSO-d_6) δ 8.59 (br s, 3H), 7.49 – 7.25 (m, 7H), 7.02 (d, J = 8.7 Hz, 2H), 5.12 (s, 2H), 3.91 (s, 2H). **^{13}C NMR (101 MHz, DMSO-d_6)** δ 158.74 , 137.41 , 131.06 , 128.90 , 128.28 , 128.08 , 126.70 , 115.23 , 69.61 , 42.03 . **Off white solid.**

(4-((2-chloro-6-fluorobenzyl)oxy)-3-methoxyphenyl)methanamine hydrochloride

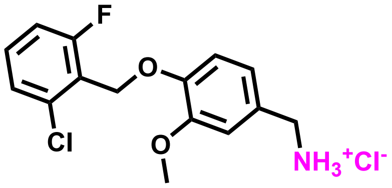

^{1}H NMR (400 MHz, DMSO-d_6) δ 8.55 (br s, 3H), 7.56 – 7.46 (m, 1H), 7.45 – 7.38 (m, 1H), 7.36 – 7.26 (m, 2H), 7.13 (d, J = 8.2 Hz, 1H), 7.03 (dd, J = 8.2, 2.0 Hz, 1H), 5.12 (s, 2H), 3.95 (s, 2H), 3.75 (s, 3H). **^{13}C NMR (101 MHz, DMSO-d_6)** δ 161.94 (d, J = 249.9 Hz), 149.52 , 148.10 , 136.00 (d, J = 5.1 Hz), 132.29 (d, J = 9.9 Hz), 127.91 , 126.18 (d, J = 3.2 Hz), 122.49 (d, J = 17.9 Hz), 121.85 , 115.23 (d, J = 22.4 Hz), 114.14 , 113.80 , 62.12 , 56.07 , 42.47 . **Off white solid.**

[1,1'-biphenyl]-4-ylmethanamine hydrochloride

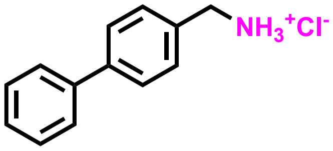

^{1}H NMR (400 MHz, DMSO-d_6) δ 8.75 (br s, 3H), 7.77 – 7.58 (m, 6H), 7.50 – 7.44 (m, 2H), 7.41 – 7.34 (m, 1H), 4.06 (s, 2H). **^{13}C NMR (101 MHz, DMSO-d_6)** δ 140.59 , 139.99 , 133.74 , 130.12 , 129.45 , 128.12 , 127.19 , 127.14 , 42.25 . **Off white solid.**

KM24-108 2,2-diphenylethan-1-amine hydrochloride

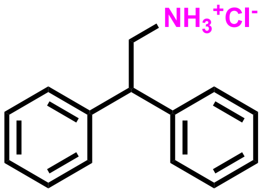

^{1}H NMR (400 MHz, DMSO-d_6) δ 8.20 (br s, 3H), 7.41 – 7.30 (m, 8H), 7.27 – 7.20 (m, 2H), 4.41 (t, J = 7.9 Hz, 1H), 3.53 (d, J = 7.9 Hz, 2H). **^{13}C NMR (101 MHz, DMSO-d_6)** δ 141.56 , 129.22 , 128.29 , 127.45 , 48.97 , 42.82 . **Pale brown solid**.

3-(4-(tert-butyl)phenyl)-2-methylpropan-1-amine hydrochloride

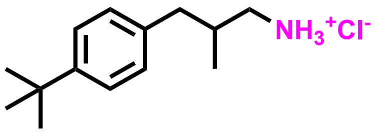

^{1}H NMR (400 MHz, DMSO-d_6) δ 8.36 (br s, 3H), 7.34 (d, J = 8.2 Hz, 2H), 7.17 (d, J = 8.3 Hz, 2H), 2.81 – 2.71 (m, 2H), 2.71 – 2.62 (m, 1H), 2.39 (dd, J = 13.5, 8.3 Hz, 1H), 2.19 – 2.06 (m, 1H), 1.29 (s, 9H), 0.92 (d, J = 6.6 Hz, 3H). **^{13}C NMR (101 MHz, DMSO-d_6)** δ 148.65 , 136.95 , 129.15 , 125.39 , 44.37 , 39.67 , 34.50 , 33.54 , 31.65 , 17.46 . **Pale brown solid.**

1-(3-methoxyphenyl)ethan-1- amine hydrochloride

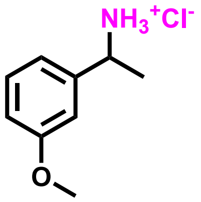

^{1}H NMR (300 MHz, DMSO-d_6) δ 8.57 (br s, 3H), 7.31 (dd, J = 8.2, 7.6 Hz, 1H), 7.27 – 7.19 (m, 1H), 7.14 – 7.04 (m, 1H), 6.91 (ddd, J = 8.3, 2.6, 0.9 Hz, 1H), 4.33 (q, J = 6.8 Hz, 1H), 3.77 (s, 3H), 1.52 (d, J = 6.7 Hz, 3H). **^{13}C NMR (75 MHz, DMSO-d_6)** δ 159.85, 141.54, 130.18 , 119.34 , 114.19 , 113.05 , 55.70 , 50.48 , 21.37 . **White solid.**

1-(4-fluorophenyl)ethan-1- amine hydrochloride

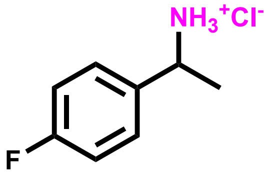

^{1}H NMR (300 MHz, DMSO-d_6) δ 8.76 (br s, 3H), 7.78 – 7.48 (m, 2H), 7.35 – 7.10 (m, 2H), 4.50 – 4.24 (m, 1H), 1.52 (d, J = 6.8 Hz, 3H). **^{13}C NMR (75 MHz, DMSO-d_6)** δ 162.34 (d, J = 244.4 Hz), 136.19 (d, J = 3.1 Hz), 129.71 (d, J = 8.4 Hz), 115.85 (d, J = 21.4 Hz), 49.80 , 21.25 . **Brown solid.**

1-(2-methoxy-4-(trifluoromethoxy)phenyl)ethan-1-amine hydrochloride

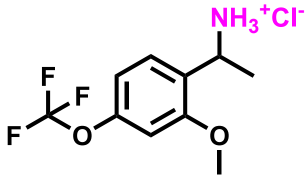

^{1}H NMR (300 MHz, DMSO-d_6) δ 8.58 (br s, 3H), 7.68 (d, J = 8.4 Hz, 1H), 7.26 – 6.84 (m, 2H), 4.79 – 4.40 (m, 1H), 3.94 (s, 3H), 1.55 (d, J = 6.7 Hz, 3H). **^{13}C NMR (75 MHz, DMSO-d_6)** δ 157.82 , 149.65 , 128.81 , 126.76 ,120.49 (q, J = 256.5 Hz), 112.91 , 105.51 , 56.76 , 44.66 , 19.56 . **Off white solid.**

KM24-154 2-methyl-1-phenylpropan-1-amine hydrochloride

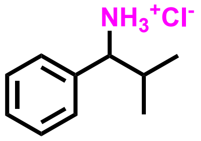

^{1}H NMR (300 MHz, DMSO-d_6) δ 8.08 (br s, 3H), 7.53 – 7.44 (m, 2H), 7.42 – 7.28 (m, 3H), 4.06 – 3.70 (m, 1H), 2.30 – 2.06 (m, 1H), 0.99 (d, *J* = 6.2 Hz, 3H), 0.67 (d, *J* = 6.3 Hz, 3H). **^{13}C NMR (75 MHz, DMSO-d_6)** δ 137.93 , 128.82 , 128.65 , 128.33 , 60.57 , 32.60 , 20.03 , 18.96 . **Off white solid.**

1-(benzo[d][1,3]dioxol-5-yl)ethan-1-amine hydrochloride

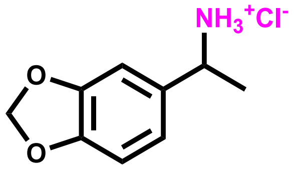

^{1}H NMR (300 MHz, DMSO-d_6) δ 8.66 (br s, 3H), 7.21 (d, *J* = 1.7 Hz, 1H), 6.99 (dd, *J* = 8.1, 1.7 Hz, 1H), 6.91 (d, *J* = 8.0 Hz, 1H), 6.02 (s, 2H), 4.40 – 4.12 (m, 1H), 1.49 (d, *J* = 6.7 Hz, 3H). **^{13}C NMR (75 MHz, DMSO-d_6)** δ 147.81 , 147.52 , 133.69 , 121.10 , 108.65 , 107.85 , 101.64 , 50.34 , 21.30 . **Off white solid.**

4-(1H-indol-3-yl)butan-2-amine hydrochloride

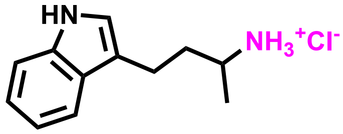

^{1}H NMR (300 MHz, DMSO-d_6) δ 11.01 (s, 1H), 8.32 (br s, 3H), 7.57 (d, *J* = 7.7 Hz, 1H), 7.39 (d, *J* = 8.0 Hz, 1H), 7.18 (d, *J* = 2.1 Hz, 1H), 7.14 – 7.02 (m, 1H), 7.03 – 6.94 (m, 1H), 3.31 – 3.02 (m, 1H), 2.88 – 2.65 (m, 2H), 2.20 – 1.98 (m, 1H), 1.91 – 1.68 (m, 1H), 1.30 (d, *J* = 6.5 Hz, 3H). **^{13}C NMR (75 MHz, DMSO-d_6)** δ 136.81 , 127.40 , 122.81 , 121.40 , 118.85 , 118.64 , 113.59 , 111.95 , 47.13 , 35.17 , 21.26 , 18.54 . **Brown solid.**

1-(4-(methylthio)phenyl)ethan-1-amine hydrochloride

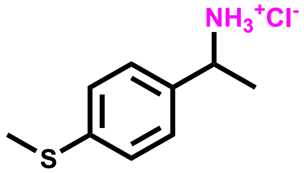

^{1}H NMR (300 MHz, DMSO-d_6) δ 8.31 (br s, 3H), 7.48 (d, *J* = 7.9 Hz, 2H), 7.29 (d, *J* = 8.0 Hz, 2H), 4.53 – 4.08 (m, 1H), 2.47 (s, 3H), 1.51 (d, *J* = 6.4 Hz, 3H). **^{13}C NMR (75 MHz, DMSO-d_6)** δ 138.88 , 136.38 , 128.07 , 126.44 , 50.09 , 21.27 , 15.29 . **Brown solid.**

1-(4-(methylsulfonyl)phenyl)ethan-1-amine hydrochloride

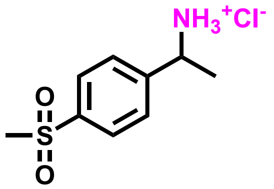

^{1}H NMR (300 MHz, DMSO-d_6) δ 8.87 (br s, 3H), 7.98 (d, J = 8.4 Hz, 2H), 7.84 (d, J = 8.4 Hz, 2H), 4.66 – 4.42 (m, 1H), 3.24 (s, 3H), 1.55 (d, J = 6.8 Hz, 3H). **^{13}C NMR (75 MHz, DMSO-d_6)** δ 145.49 , 141.06 , 128.44 , 127.74 , 50.03 , 43.85 , 21.12 . **Pale brown solid.**

4-(3-aminobutyl)phenol

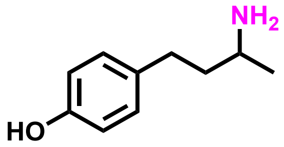

^{1}H NMR (300 MHz, DMSO-d_6) δ 6.98 (d, J = 8.4 Hz, 2H), 6.68 (d, J = 8.4 Hz, 2H), 6.28 (br s, 2H), 3.02 – 2.78 (m, 1H), 2.66 – 2.33 (m, 2H), 1.82 – 1.48 (m, 2H), 1.10 (d, J = 6.4 Hz, 3H). **^{13}C NMR (75 MHz, DMSO-d_6)** δ 155.88 , 132.03 , 129.44 , 115.57 , 46.57 , 39.49 , 31.06 , 21.37 . **Brown solid.**

4-(4-hydroxy-3-methoxyphenyl)butan-2-amine hydrochloride

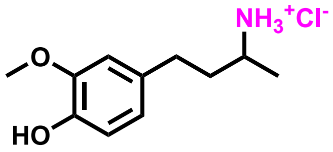

^{1}H NMR (300 MHz, DMSO-d_6) δ 8.27 (br s, 3H), 6.84 (d, J = 1.9 Hz, 1H), 6.78 (d, J = 7.9 Hz, 1H), 6.64 (dd, J = 8.0, 1.9 Hz, 1H), 3.79 (s, 3H), 3.27 – 2.98 (m, 1H), 2.71 – 2.55 (m, 2H), 2.10 – 1.85 (m, 1H), 1.88 – 1.62 (m, 1H), 1.28 (d, J = 6.5 Hz, 3H). **^{13}C NMR (75 MHz, DMSO-d_6)** δ 147.90 , 145.10 , 132.10 , 120.70 , 115.82 , 112.91 , 56.02 , 46.86 , 36.50 , 30.86 , 18.46 . **Pale yellow solid.**

(1-hydroxycyclohexyl)(phenyl)methanamine hydrochloride

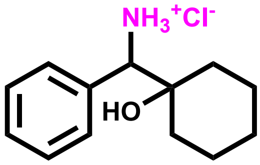

^{1}H NMR (300 MHz, DMSO-d_6) δ 8.45 (br s, 3H), 7.57 – 7.42 (m, 2H), 7.45 – 7.24 (m, 3H), 5.04 (s, 1H), 4.11 (s, 1H), 1.89 – 0.90 (m, 10H). **^{13}C NMR (75 MHz, DMSO-d_6)** δ 135.87 , 129.40 , 128.62 , 128.38 , 71.20 , 63.23 , 34.87 , 33.02 , 25.48 , 21.47 , 21.12 . **Off white solid.**

1-phenylpentan-1- amine hydrochloride

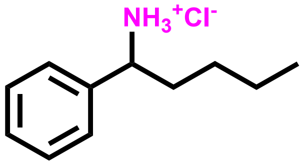

^{1}H NMR (300 MHz, DMSO-d_6) δ 8.59 (br s, 3H), 7.56 – 7.46 (m, 2H), 7.46 – 7.34 (m, 3H), 4.15 (dd, J = 9.5, 5.4 Hz, 1H), 2.05 – 1.91 (m, 1H), 1.89 – 1.70 (m, 1H), 1.30 – 0.93 (m, 4H), 0.80 (t, J = 7.1 Hz, 3H). **^{13}C NMR (75 MHz, DMSO-d_6)** δ 138.41 , 129.15 , 128.94 , 127.91 , 54.94 , 34.36 , 27.65 , 22.10 , 14.18 . **Pale brown solid.**

1,3-diphenylpropan-2- amine hydrochloride

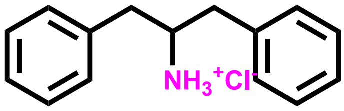

^{1}H NMR (300 MHz, DMSO-d_6) δ 8.26 (br s, 3H), 7.44 – 7.08 (m, 10H), 3.76 – 3.56 (m, 1H), 3.03 (dd, J = 13.9, 6.2 Hz, 2H), 2.80 (dd, J = 13.9, 6.9 Hz, 2H). **^{13}C NMR (75 MHz, DMSO-d_6)** δ 136.96 , 129.84 , 129.07 , 127.25 , 53.61 , 38.17 . **Brown solid.**

1-phenyl-2-(p-tolyl)ethan-1-amine hydrochloride

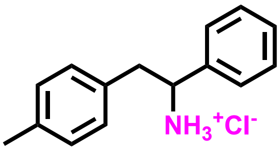

^{1}H NMR (300 MHz, DMSO-d_6) δ 8.87 (br s, 3H), 7.50 – 7.37 (m, 2H), 7.36 – 7.25 (m, 3H), 7.06 – 6.84 (m, 4H), 4.53 – 4.29 (m, 1H), 3.42 (dd, J = 13.4, 5.0 Hz, 1H), 3.08 (dd, J = 13.3, 10.2 Hz, 1H), 2.18 (s, 3H). **^{13}C NMR (75 MHz, DMSO-d_6)** δ 137.56 , 135.97 , 133.58 , 129.56 , 129.29 , 128.86 , 128.86 , 128.38 , 56.43 , 40.25 , 21.07 . **White solid.**

1-(4-hydroxy-3-methoxyphenyl)propan-2- amine hydrochloride

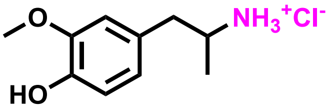

^{1}H NMR (300 MHz, DMSO-d_6) δ 8.19 (br s, 3H), 6.80 (d, J = 1.9 Hz, 1H), 6.74 (d, J = 8.0 Hz, 1H), 6.60 (dd, J = 8.0, 1.9 Hz, 1H), 3.75 (s, 3H), 3.39 – 3.23 (m, 1H), 2.93 (dd, J = 13.4, 5.2 Hz, 1H), 2.58 (dd, J = 9.0, 4.4 Hz, 1H), 1.12 (d, J = 6.7 Hz, 3H). **^{13}C NMR (75 MHz, DMSO-d_6)** δ 147.98 , 145.80 , 127.84 , 121.92 , 115.94 , 113.73 , 56.00 , 48.66 , 31.76 , 18.04 . **Brown solid.**

4-(6-methoxynaphthalen-2-yl)butan-2-amine hydrochloride

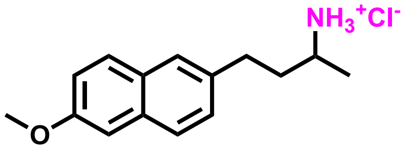

^{1}H NMR (300 MHz, DMSO-d_6) δ 8.33 (br s, 3H), 7.74 (dd, J = 8.7, 2.7 Hz, 2H), 7.65 – 7.60 (m, 1H), 7.34 (dd, J = 8.4, 1.7 Hz, 1H), 7.26 (d, J = 2.5 Hz, 1H), 7.12 (dd, J = 8.9, 2.5 Hz, 1H), 3.83 (s, 3H), 3.32 – 3.09 (m, 1H), 2.93 – 2.66 (m, 2H), 2.18 – 1.93 (m, 1H), 1.93 – 1.70 (m, 1H), 1.28 (d, J = 6.5 Hz, 3H). **^{13}C NMR (75 MHz, DMSO-d_6)** δ 157.26 , 136.54 , 133.26 , 129.25 , 129.02 , 128.02 , 127.31 , 126.40 , 119.00 , 106.23 , 55.60 , 46.97 , 36.21 , 31.28 , 18.51 . **Off white solid.**

6-(3,7-dimethyl-2,6-dioxo-2,3,6,7-tetrahydro-1H-purin-1-yl)hexan-2-amine hydrochloride

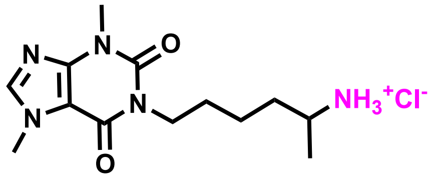

^{1}H NMR (300 MHz, Methanol-d_4) δ 7.91 (s, 1H), 4.83 (br s, 3H), 3.89 (s, 3H), 3.87 – 3.81 (m, 2H), 3.40 (s, 3H), 3.29 – 3.15 (m, 1H), 1.71 – 1.47 (m, 4H), 1.44 – 1.30 (m, 2H), 1.24 (d, J = 6.5 Hz, 3H). **^{13}C NMR (75 MHz, Methanol-d_4)** δ 154.88 , 151.35 , 147.73 , 142.34 , 107.26 , 47.55 , 40.46 , 33.88 , 32.92 , 28.96 , 27.13 , 22.29 , 17.33 . **White solid.**

1-(4-fluorophenyl)-4-(4-(pyridin-2-yl)piperazin-1-yl)butan-1-amine

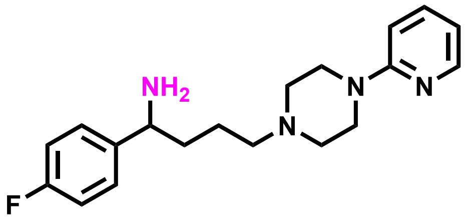

^{1}H NMR (300 MHz, Chloroform-d) δ 8.14 – 8.04 (m, 1H), 7.45 – 7.33 (m, 1H), 7.29 – 7.21 (m, 2H), 6.98 – 6.89 (m, 2H), 6.58 – 6.47 (m, 2H), 3.87 (t, J = 6.9 Hz, 1H), 3.65 (br s, 2H), 3.49 – 3.38 (m, 4H), 2.49 – 2.37 (m, 4H), 2.34 – 2.25 (m, 2H), 1.93 – 1.27 (m, 4H). **^{13}C NMR (75 MHz, Chloroform-d)** δ 161.96 (d, J = 245.2 Hz), 159.46 , 147.91 , 140.36 , 137.44 , 128.11 (d, J = 7.9 Hz), 115.36 (d, J = 21.2 Hz), 113.31 , 107.07 , 58.33 , 55.45 , 52.96 , 45.09 , 36.85 , 23.69 . **Brown solid.**

(8R,9S,13S,14S)-3-hydroxy-13-methyl-7,8,9,11,12,13,14,15,16,17-decahydro-6H-cyclopenta[a]phenanthren-17-amine hydrochloride **(diastereomeric mixture)**

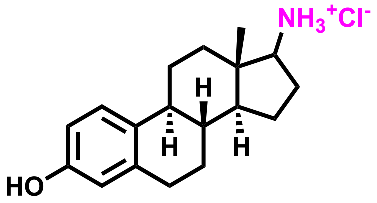

^{1}H NMR (300 MHz, DMSO-*d*$_6$) (diastereomeric mixture) δ 9.09 (br s, 1H), 8.24 (br s, 3H), 7.03 (d, *J* = 8.1 Hz, 1H), 6.53 (dd, *J* = 8.4, 2.5 Hz, 1H), 6.46 (d, *J* = 2.6 Hz, 1H), 3.22 – 2.91 (m, 1H), 2.86 – 2.61 (m, 2H), 2.30 – 2.00 (m, 4H), 1.85 – 1.51 (m, 4H), 1.35 – 1.20 (m, 5H), 0.75 (s, 3H). **^{13}C NMR (75 MHz, DMSO-*d*$_6$)** (diastereomeric mixture) δ 155.52 , 155.41 , 137.44 , 137.41 , 130.82 , 130.36 , 126.53 , 126.42 , 115.43 , 115.40, 113.26 , 113.21 , 59.95, 59.24, 51.27 , 49.98 , 47.93 , 43.99 , 43.86 , 43.56 , 43.27 , 39.16 , 38.66 , 36.23 , 29.64 , 29.53 , 28.38 , 28.26 , 27.50 , 27.44 , 26.71 , 26.18 , 26.09 , 23.55 , 18.33 , 12.15 . (traces of ethylacetate solvent peak was observed in the NMR spectra). **HRMS (EI):** Calcd for C18H25NO [M]+ 271.1930; found 271.1929. **Off white solid.**

4-(tert-butyl)cyclohexan-1- amine hydrochloride **(diastereomeric mixture)**

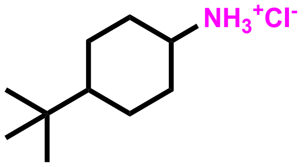

^{1}H NMR (300 MHz, DMSO-*d*$_6$) (diastereomeric mixture) δ 7.43 (br s, 3H), 3.32 – 2.56 (m, 1H), 2.19 – 1.23 (m, 7H), 1.06 – 0.87 (m, 2H), 0.78 (s, 9H). **^{13}C NMR (75 MHz, DMSO-*d*$_6$)** (diastereomeric mixture) δ 49.94 , 47.50 , 46.72 , 46.01 , 32.72 , 32.47 , 30.89 , 28.93 , 27.94 , 27.81 , 25.37 , 20.71 . **Off white solid.**

Nonan-5-amine hydrochloride

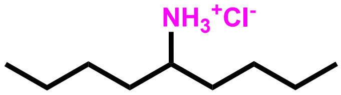

^{1}H NMR (300 MHz, DMSO-*d*$_6$) δ 8.10 (br s, 3H), 3.11 – 2.84 (m, 1H), 1.57 – 1.47 (m, 4H), 1.33 – 1.20 (m, 8H), 0.90 – 0.81 (m, 6H). **^{13}C NMR (75 MHz, DMSO-*d*$_6$)** δ 51.09 , 31.92 , 27.01 , 22.44 , 14.20 . **White solid.**

Octan-2-amine hydrochloride

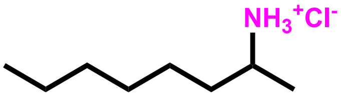

^{1}H NMR (300 MHz, DMSO-d_6) δ 8.09 (br s, 3H), 3.29 – 3.04 (m, 1H), 1.73 – 1.59 (m, 1H), 1.58 – 1.42 (m, 1H), 1.40 – 1.27 (m, 8H), 1.23 (d, J = 6.5 Hz, 3H), 0.96 – 0.89 (m, 3H). **^{13}C NMR (75 MHz, DMSO-d_6)** δ 47.23 , 34.52 , 31.51 , 28.88 , 25.18 , 22.44 , 18.53 , 14.40 . **White solid.**

4-(2,6,6-trimethylcyclohex-1-en-1-yl)butan-2-amine hydrochloride

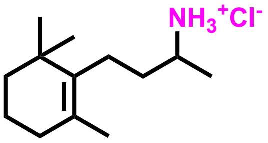

^{1}H NMR (300 MHz, DMSO-d_6) δ 8.14 (br s, 3H), 3.20 – 3.05 (m, 1H), 2.07 – 1.92 (m, 2H), 1.87 (t, J = 6.2 Hz, 2H), 1.75 – 1.59 (m, 1H), 1.56 (s, 3H), 1.54 – 1.44 (m, 3H), 1.42 – 1.33 (m, 2H), 1.22 (d, J = 6.5 Hz, 3H), 0.97 (s, 3H), 0.97 (s, 3H). **^{13}C NMR (75 MHz, DMSO-d_6)** δ 136.49 , 127.31 , 47.71 , 39.78 , 35.18 , 35.05 , 32.69 , 28.84 , 24.60 , 20.07 , 19.48 , 18.43 . **Brown solid.**

(5S,8R,9R,10S,13S,14S,17S)-17-hydroxy-10,13-dimethylhexadecahydro-1H-cyclopenta[a]phenanthren-3-amine hydrochloride **(diastereomeric mixture)**

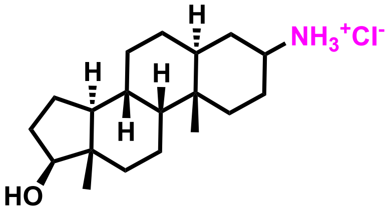

^{1}H NMR (300 MHz, DMSO-d_6) (diastereomeric mixture) δ 8.07 (br s, 3H), 4.44 (s, 1H), 3.57 – 3.33 (m, 1H), 1.99 – 0.81 (m, 23H), 0.75 (s, 3H), 0.63 (s, 3H). **^{13}C NMR (75 MHz, DMSO-d_6)** (diastereomeric mixture) δ 80.52, 80.48 , 54.07 , 53.68 , 52.92, 52.71, 51.21 , 51.01 , 49.90 , 46.68 , 44.57 , 44.02, 42.18, 43.02 , 38.42 , 37.11 , 37.05 , 36.49 , 36.00 , 35.56 , 35.48 , 32.76 , 31.61 , 31.26 , 30.98 , 30.30 , 28.44 , 28.21 , 26.32 , 26.27 , 24.29 , 23.51 , 20.79 , 20.72 , 20.41 , 12.28 , 11.80 , 11.55 . **HRMS (EI):** Calcd for C19H33NO [M]+ 291.2556; found 291.2549. **White solid.**

(3R,8R,9S,10S,13S,14S)-3-hydroxy-10,13-dimethylhexadecahydro-1H-cyclopenta[a]phenanthren-17-amine hydrochloride **(diastereomeric mixture)**

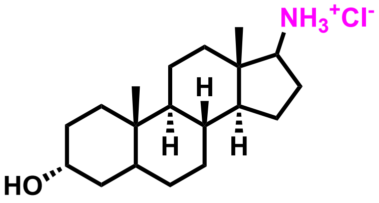

^{1}H NMR (300 MHz, DMSO-*d*$_6$) (diastereomeric mixture) δ 7.98 (br s, 3H), 3.90 – 3.71 (m, 1H), 3.14 – 2.96 (m, 1H), 2.29 – 1.89 (m, 1H), 1.79 – 0.86 (m, 21H), 0.83 – 0.56 (m, 6H). **^{13}C NMR (75 MHz, DMSO-*d*$_6$)** δ 64.62, 64.47 , 59.92 , 59.09 , 54.22 , 53.80 , 53.69, 52.40 , 49.01 , 48.93 , 43.57 , 42.22 , 38.97 , 36.26 , 36.15 , 36.11 , 35.70 , 35.26 , 32.58 , 32.47 , 32.41 , 32.36 , 31.91 , 29.07 , 28.64 , 28.58 , 28.23 , 26.57 , 24.96 , 23.80 , 20.24 , 20.16 , 18.26 , 18.07, 12.12 , 12.08, 11.57 , 11.53. **HRMS (EI):** Calcd for C19H33NO [M]+ 291.2556; found 291.2549. **White solid.**

2,4,6-trimethylaniline

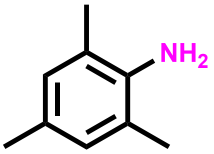

^{1}H NMR (300 MHz, Chloroform-*d*) δ 6.82 – 6.52 (m, 2H), 3.42 (br s, 2H), 2.12 (s, 3H), 2.07 (s, 6H). **^{13}C NMR (75 MHz, Chloroform-*d*)** δ 140.00 , 128.91 , 127.32 , 122.03 , 20.44 , 17.66 . **Brown oil.**

N1,N1-dimethylbenzene-1,3-diamine

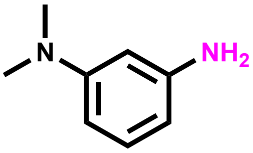

^{1}H NMR (300 MHz, Chloroform-*d*) δ 7.02 – 6.88 (m, 1H), 6.18 – 6.07 (m, 1H), 6.07 – 5.93 (m, 2H), 3.37 (br s, 2H), 2.83 (s, 6H). **^{13}C NMR (75 MHz, Chloroform-*d*)** δ 151.85 , 147.30 , 129.87 , 104.30 , 103.82 , 99.63 , 40.62 . **Brown gum.**

methyl 4-aminobenzoate

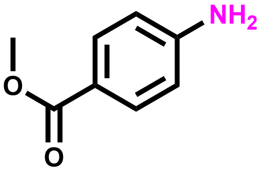

^{1}H NMR (300 MHz, DMSO-*d*$_6$) δ 7.67 (d, *J* = 8.7 Hz, 2H), 6.60 (d, *J* = 8.7 Hz, 2H), 5.95 (br s, 2H), 3.73 (s, 3H). **^{13}C NMR (75 MHz, DMSO-*d*$_6$)** δ 166.90 , 153.89 , 131.55 , 116.31 , 113.17 , 51.58 . **Brown solid.**

4-aminobenzamide

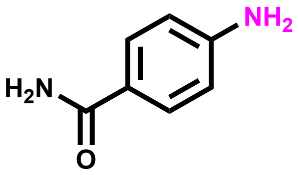

^{1}H NMR (300 MHz, DMSO-d_6) δ 7.63 (d, J = 8.6 Hz, 2H), 6.94 (s, 1H), 6.57 (d, J = 8.6 Hz, 2H), 5.62 (s, 1H), 3.63 (br s, 2H). **^{13}C NMR (75 MHz, DMSO-d_6)** δ 168.85 , 152.17 , 129.65 , 121.29 , 113.04 . **Pale brown solid.**

2,6-dichlorobenzene-1,4-diamine

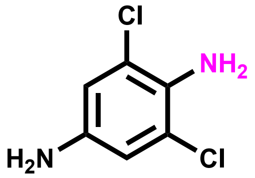

^{1}H NMR (300 MHz, Chloroform-d) δ 6.53 (s, 2H), 3.79 (br s, 2H), 3.41 (br s, 2H). **^{13}C NMR (75 MHz, Chloroform-d)** δ 138.36 , 132.53 , 120.81 , 115.41 . **Brown solid.**

4-phenoxyaniline

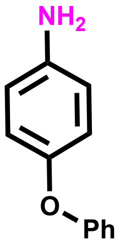

^{1}H NMR (300 MHz, DMSO-d_6) δ 7.34 – 7.24 (m, 2H), 7.04 – 6.95 (m, 1H), 6.89 – 6.83 (m, 2H), 6.77 (d, J = 8.8 Hz, 2H), 6.62 (d, J = 8.8 Hz, 2H), 5.02 (br s, 2H). **^{13}C NMR (75 MHz, DMSO-d_6)** δ 159.44 , 145.98 , 145.88 , 130.11 , 122.17 , 121.39 , 116.87 , 115.39 . **Pale brown solid.**

[1,1'-biphenyl]-2-amine

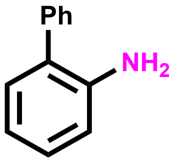

^{1}H NMR (300 MHz, Chloroform-d) δ 7.61 – 7.49 (m, 4H), 7.48 – 7.37 (m, 1H), 7.31 – 7.19 (m, 2H), 6.99 – 6.89 (m, 1H), 6.84 (dd, J = 8.1, 1.3 Hz, 1H), 3.85 (br s, 2H). **^{13}C NMR (75 MHz, Chloroform-d)** δ 143.48 , 139.60 , 130.54 , 129.19 , 128.90 , 128.59 , 127.77 , 127.26 , 118.80 , 115.76 . **Off white Solid.**

Quinolin-6-amine

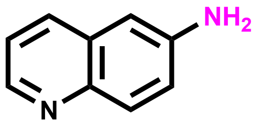

^{1}H NMR (300 MHz, DMSO-d_6) δ 8.47 (dd, J = 4.2, 1.7 Hz, 1H), 7.98 – 7.88 (m, 1H), 7.70 (d, J = 9.0 Hz, 1H), 7.27 (dd, J = 8.3, 4.2 Hz, 1H), 7.17 (dd, J = 9.0, 2.5 Hz, 1H), 6.79 (d, J = 2.5 Hz, 1H), 5.66 (br s, 2H). **^{13}C NMR (75 MHz, DMSO-d_6)** δ 147.52 , 145.39 , 142.33 , 133.43 , 130.30 , 129.91 , 122.14 , 121.66 , 105.19 . **Brown solid.**

N-(4-amino-3-(trifluoromethyl)phenyl)isobutyramide

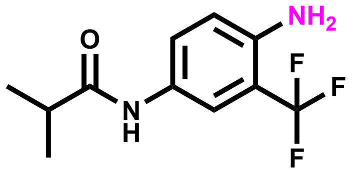

^{1}H NMR (300 MHz, DMSO-d_6) δ 9.63 (s, 1H), 7.73 (d, J = 2.4 Hz, 1H), 7.48 – 7.37 (m, 1H), 6.79 (dd, J = 8.8, 0.9 Hz, 1H), 5.32 (br s, 2H), 2.57 – 2.48 (m, 1H), 1.08 (d, J = 6.8 Hz, 6H). **^{13}C NMR (75 MHz, DMSO-d_6)** δ 175.11 , 142.48 , 128.79 , 126.44 (q, J = 272.0 Hz), 125.44, 117.61 , 117.39 (q, J = 5.6 Hz), 110.72 (q, J = 29.5 Hz), 35.21 , 19.97 . **Yellow Solid.**

S9. NMR and HRMS spectra

190128.404.10.fid
Kathir ILM24-65
Au1H DMSO {C:\Bruker\TopSpin3.5pl6} 1901 4

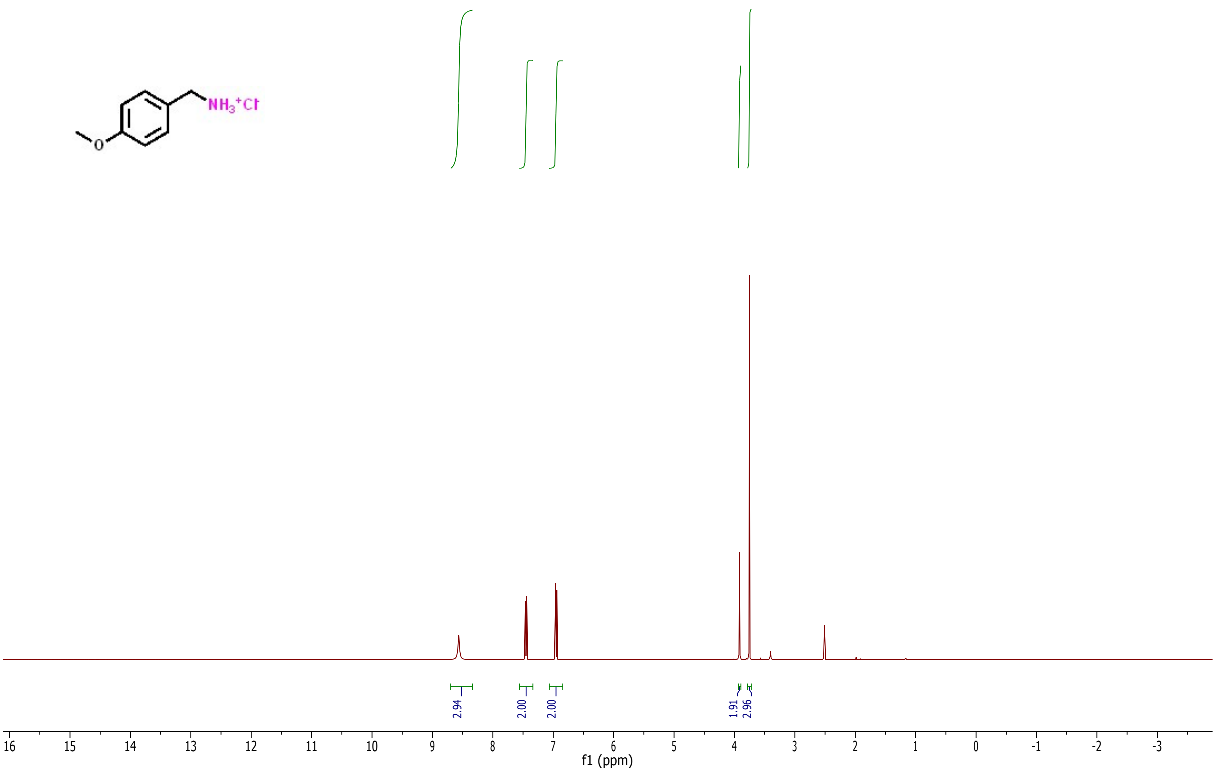

190128.404.11.fid
Kathir ILM24-65
Au13C DMSO {C:\Bruker\TopSpin3.5pl6} 1901 4

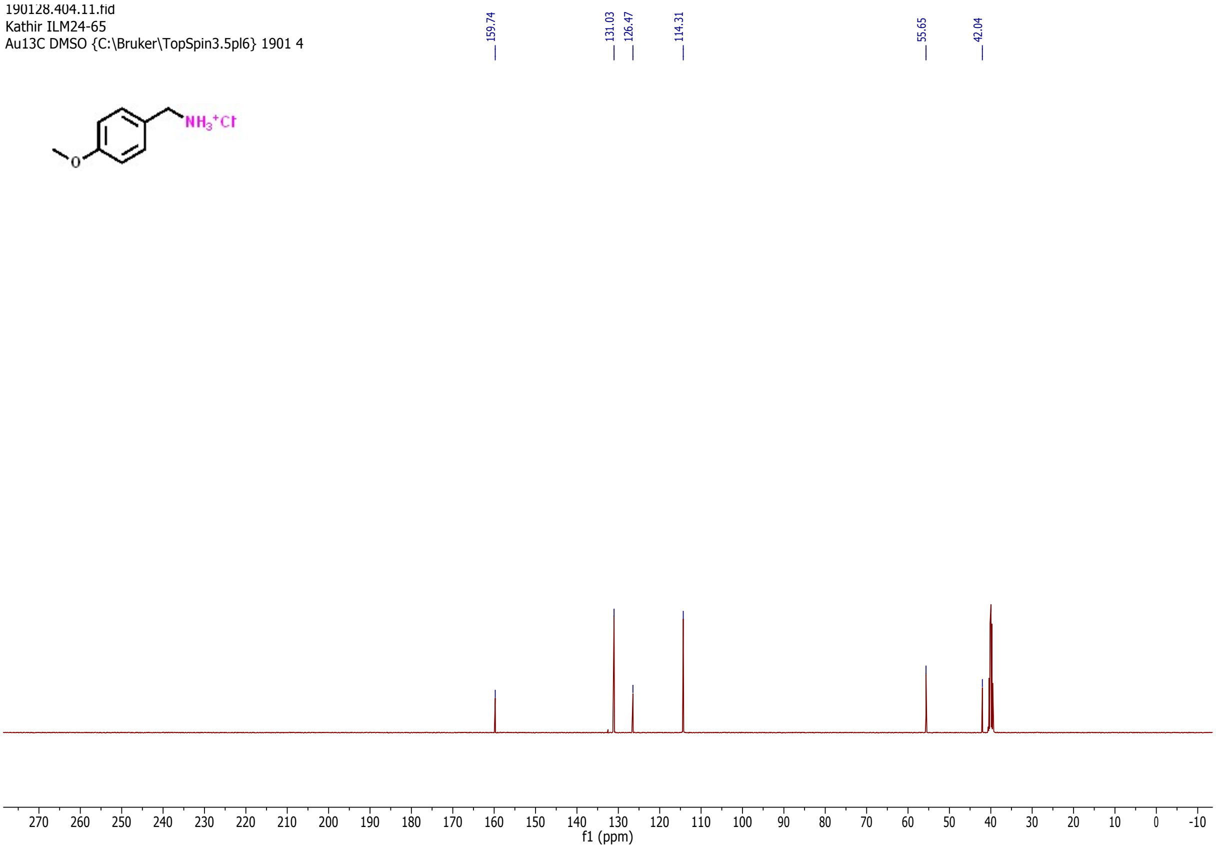

190128.407.10.fid
Kathir ILM24-89
Au1H DMSO {C:\Bruker\TopSpin3.5pl6} 1901 7

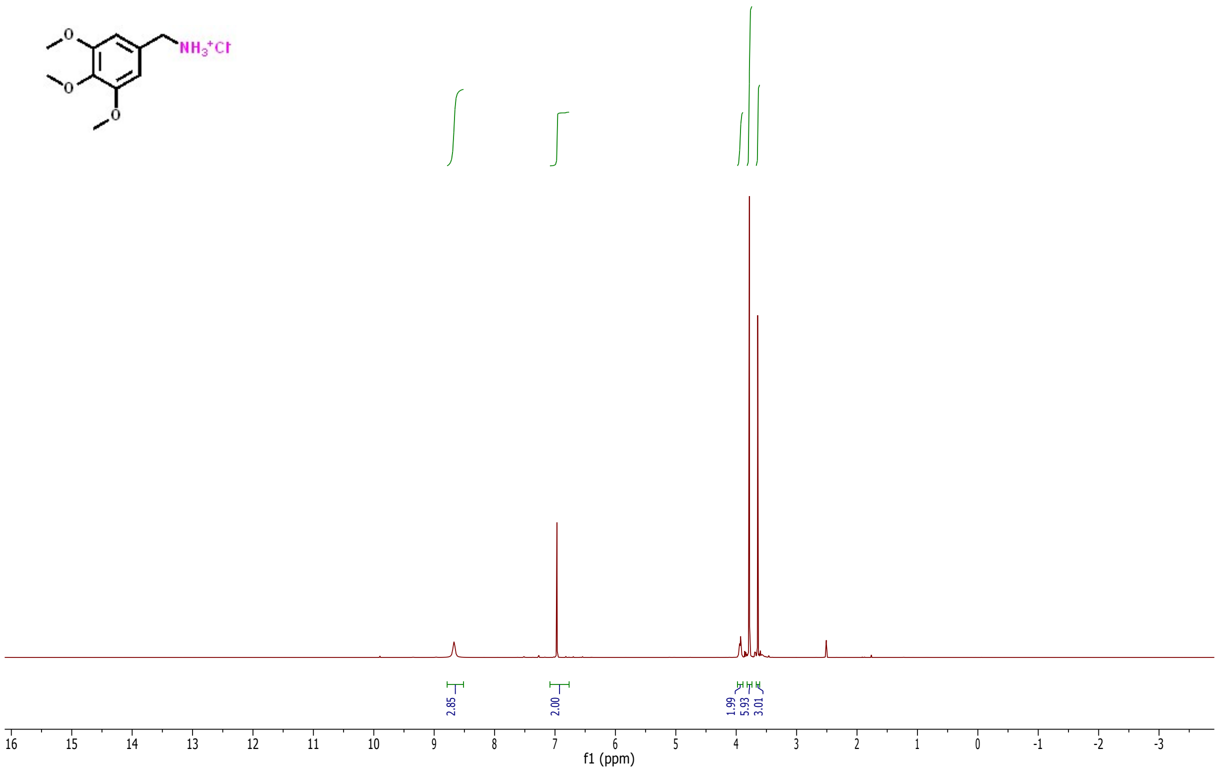

190128.407.11.fid
Kathir ILM24-89
Au13C DMSO {C:\Bruker\TopSpin3.5pl6} 1901 7

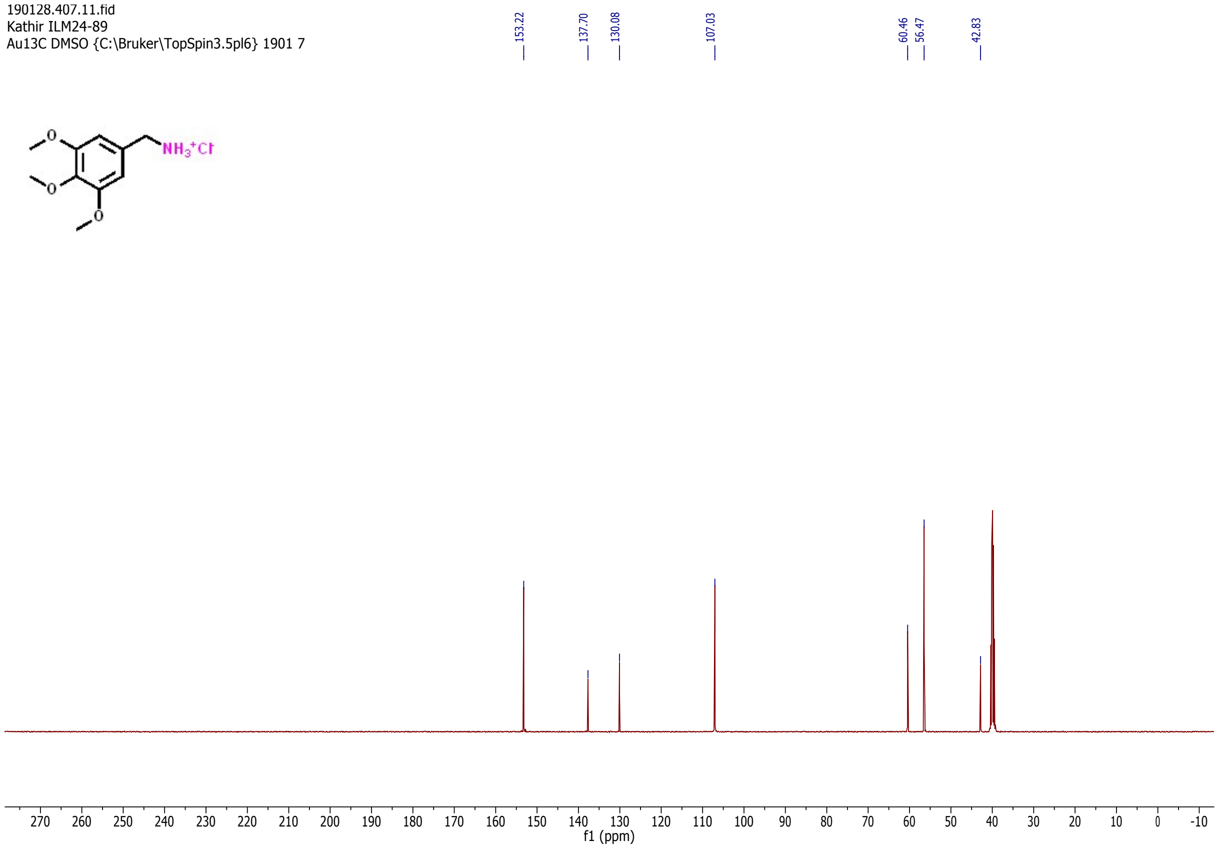

190128.408.10.fid
Kathir ILM24-66
Au1H DMSO {C:\Bruker\TopSpin3.5pl6} 1901 8

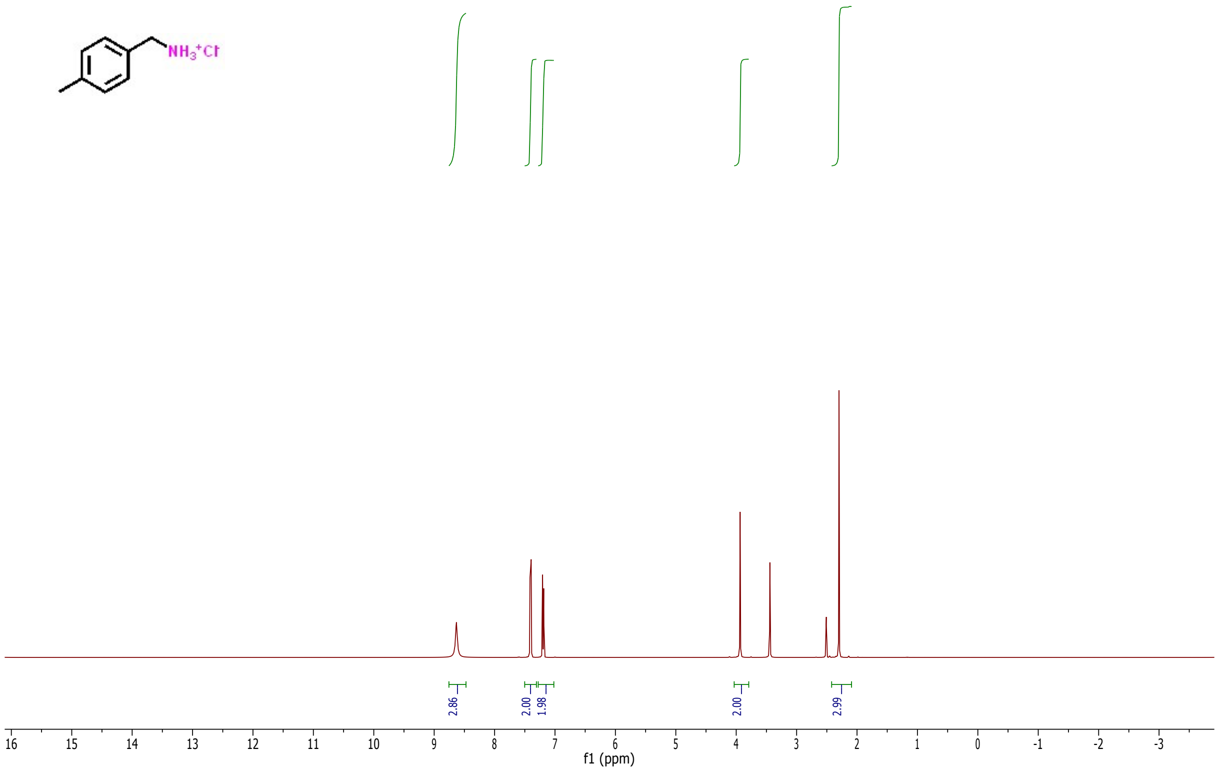

190128.408.11.fid
Kathir ILM24-66
Au13C DMSO {C:\Bruker\TopSpin3.5pl6} 1901 8

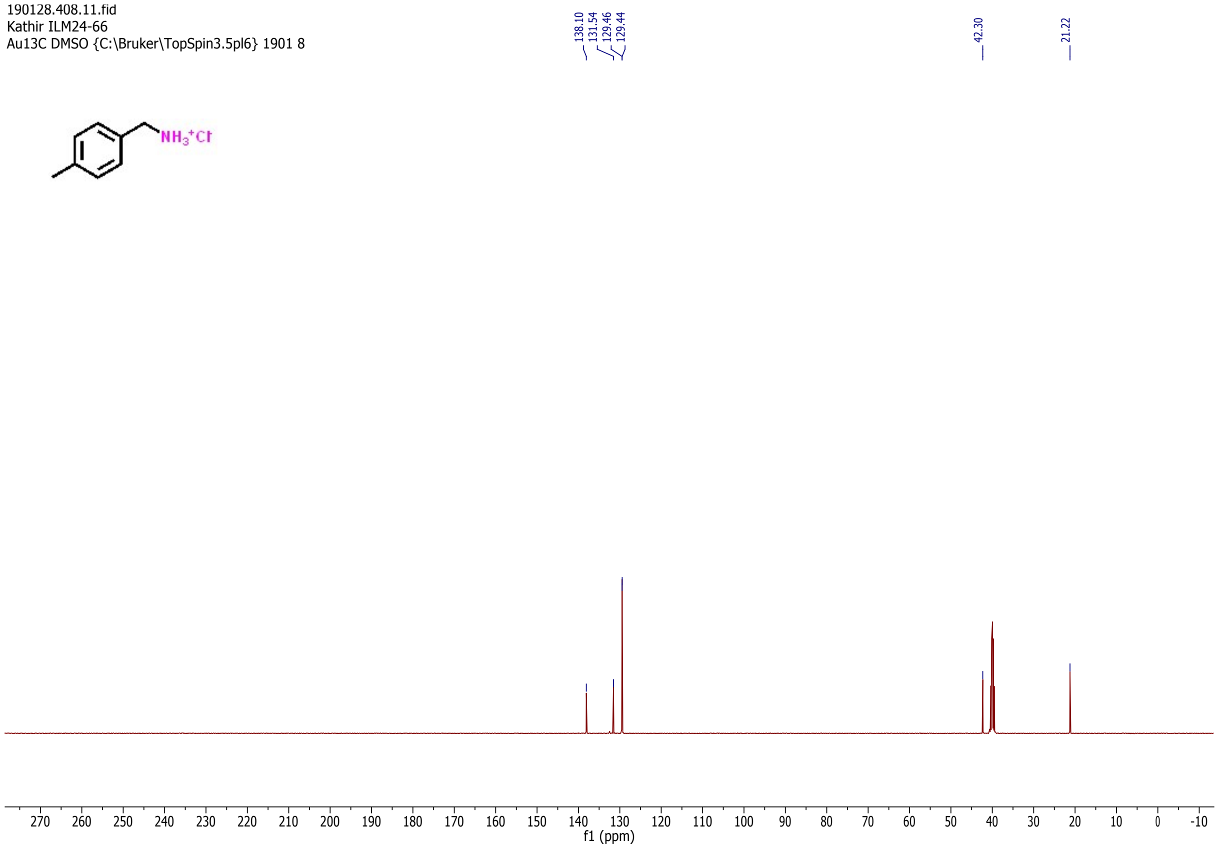

190128.406.10.fid
Kathir ILM24-67
Au1H DMSO {C:\Bruker\TopSpin3.5pl6} 1901 6

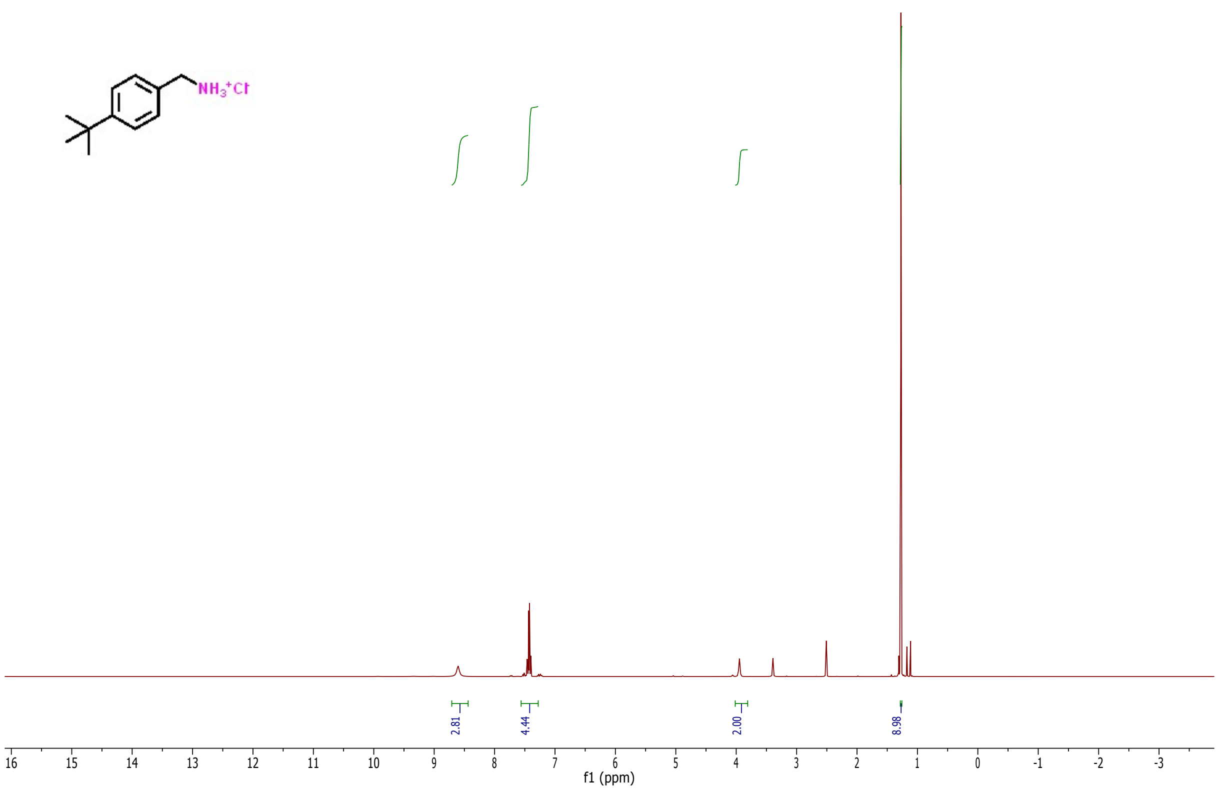

190128.406.11.fid
Kathir ILM24-67
Au13C DMSO {C:\Bruker\TopSpin3.5pl6} 1901 6

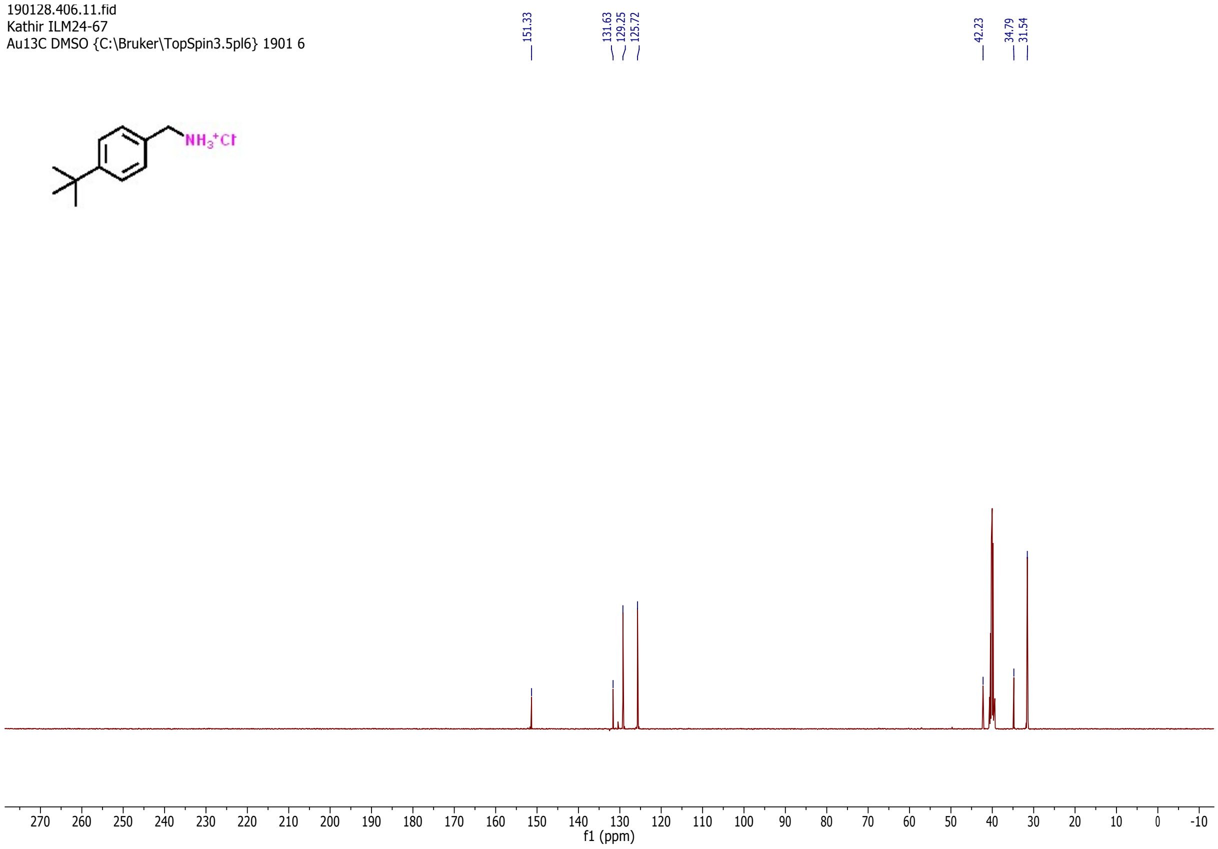

190128.419.10.fid
Kathir ILM24-71
Au1H DMSO {C:\Bruker\TopSpin3.5pl6} 1901 19

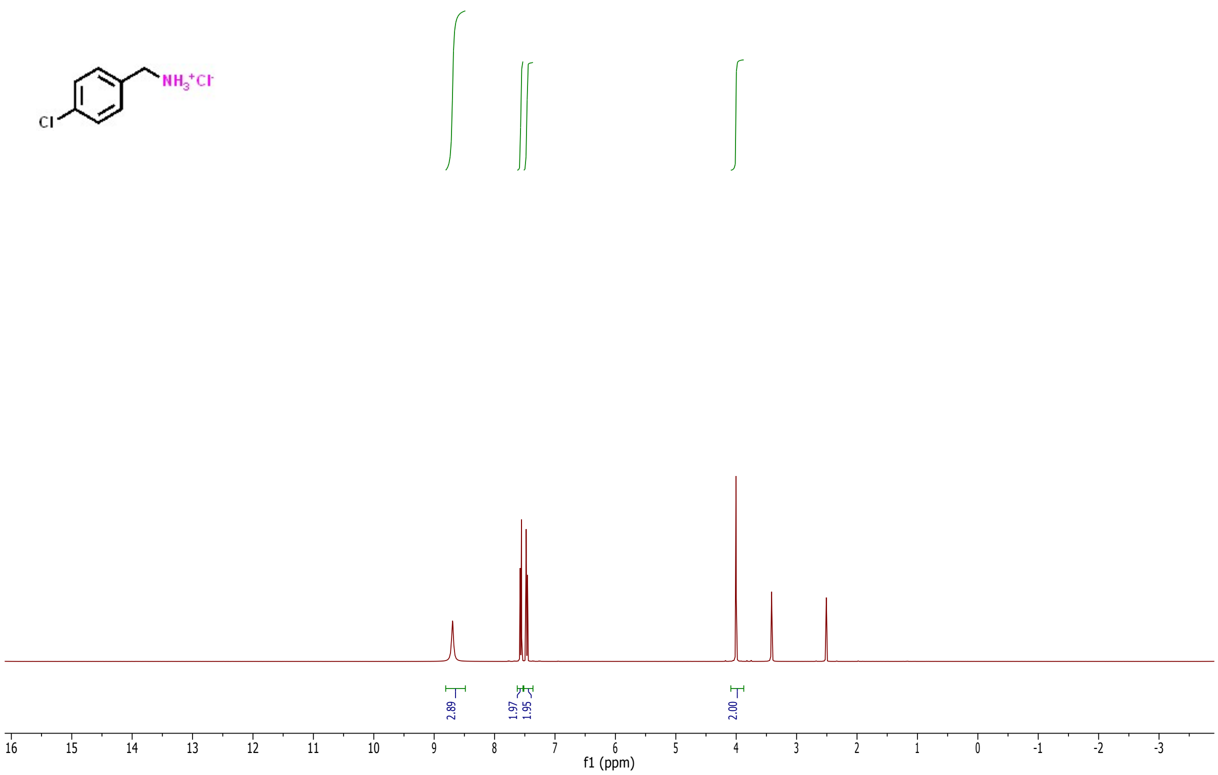

190128.419.11.fid
Kathir ILM24-71
Au13C DMSO {C:\Bruker\TopSpin3.5pl6} 1901 19

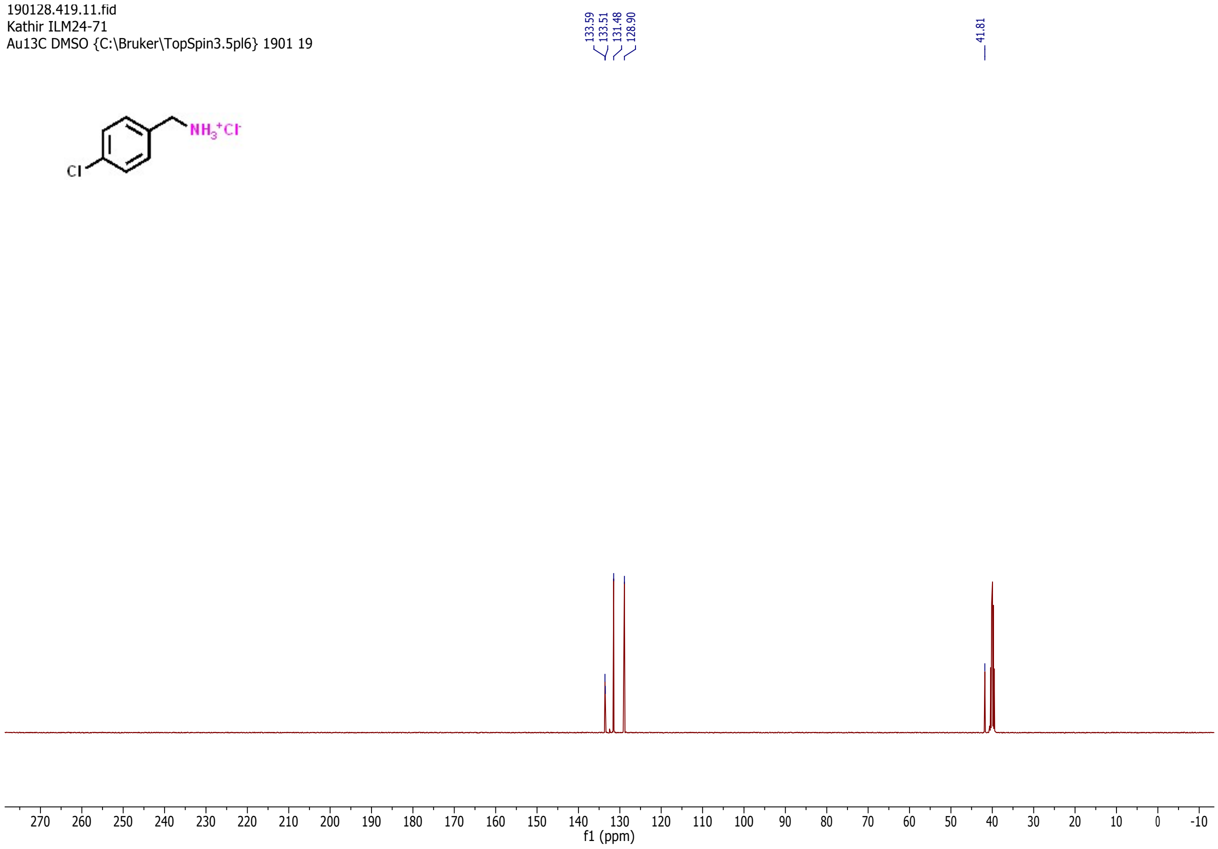

190128.411.10.fid
Kathir ILM24-105
Au1H DMSO {C:\Bruker\TopSpin3.5pl6} 1901 11

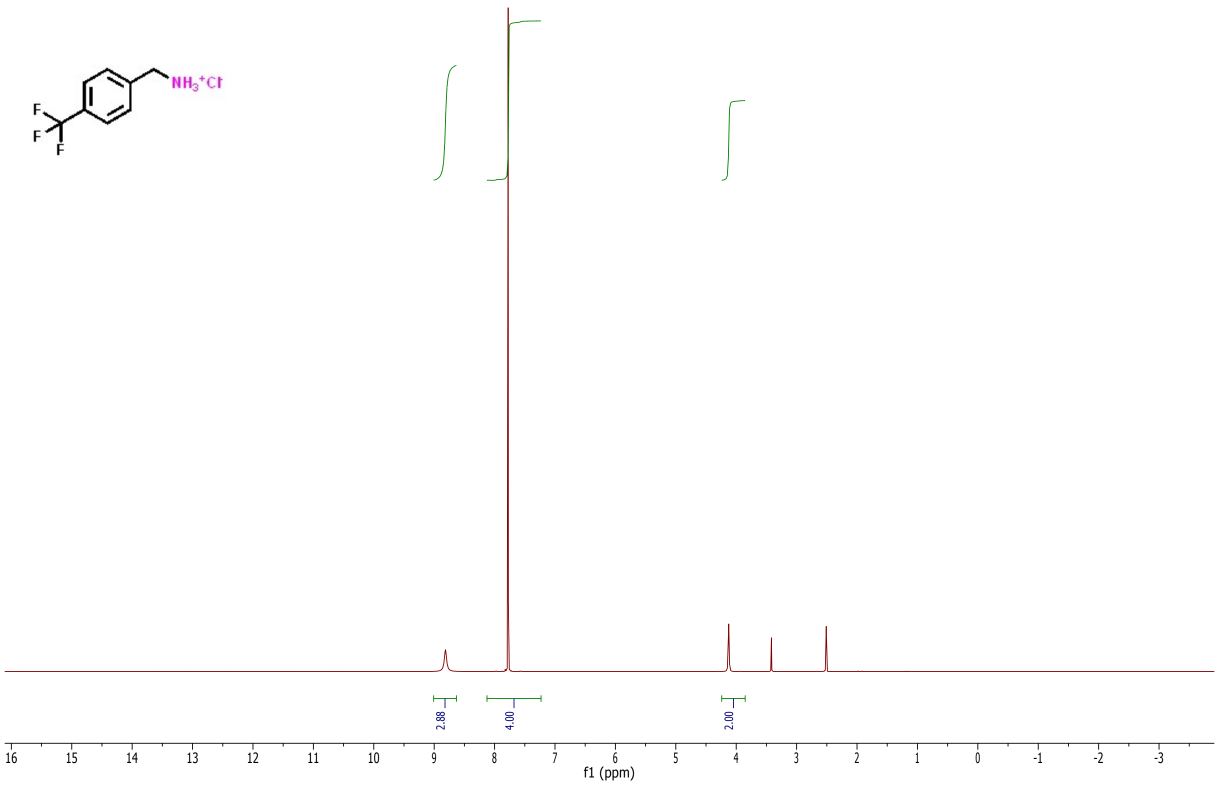

190128.411.11.fid
Kathir ILM24-105
Au13C DMSO {C:\Bruker\TopSpin3.5pl6} 1901 11

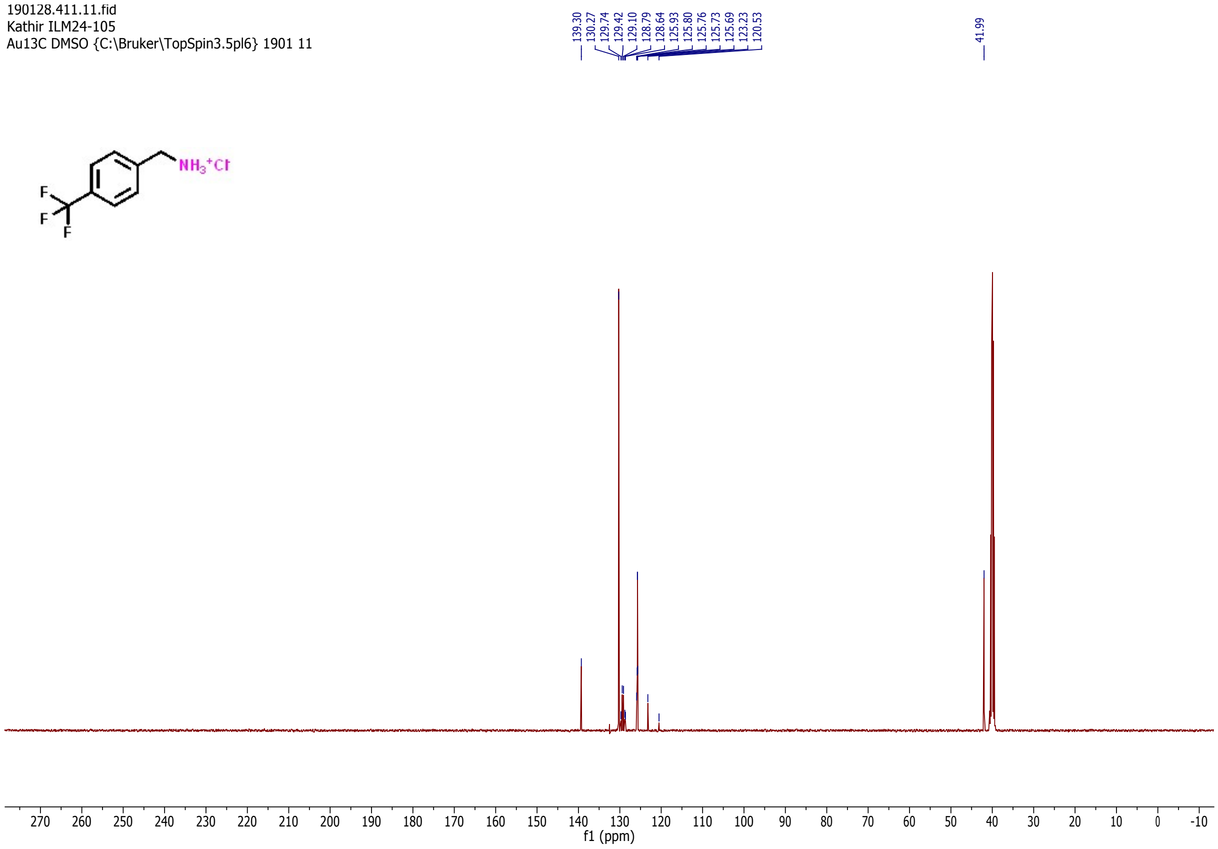

190128.409.10.fid
Kathir ILM24-179
Au1H DMSO {C:\Bruker\TopSpin3.5pl6} 1901 9

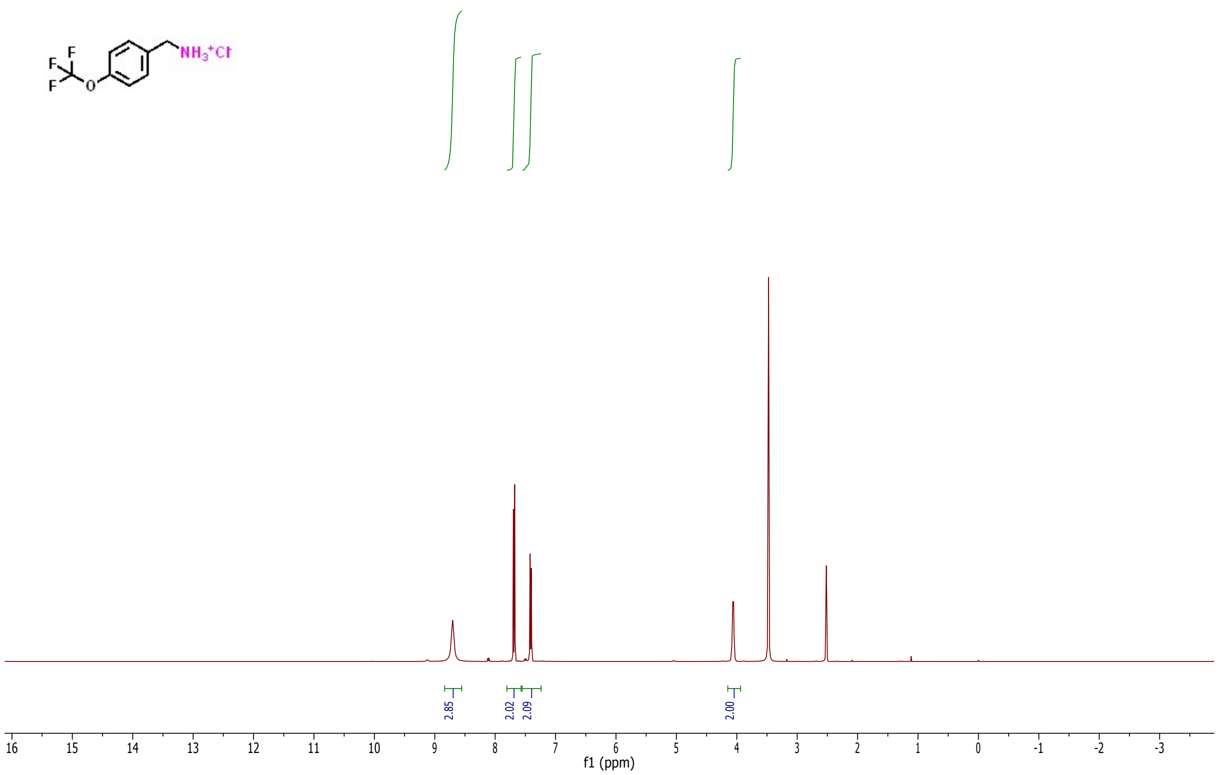

190128.409.11.fid
Kathir ILM24-179
Au13C DMSO {C:\Bruker\TopSpin3.5pl6} 1901 9

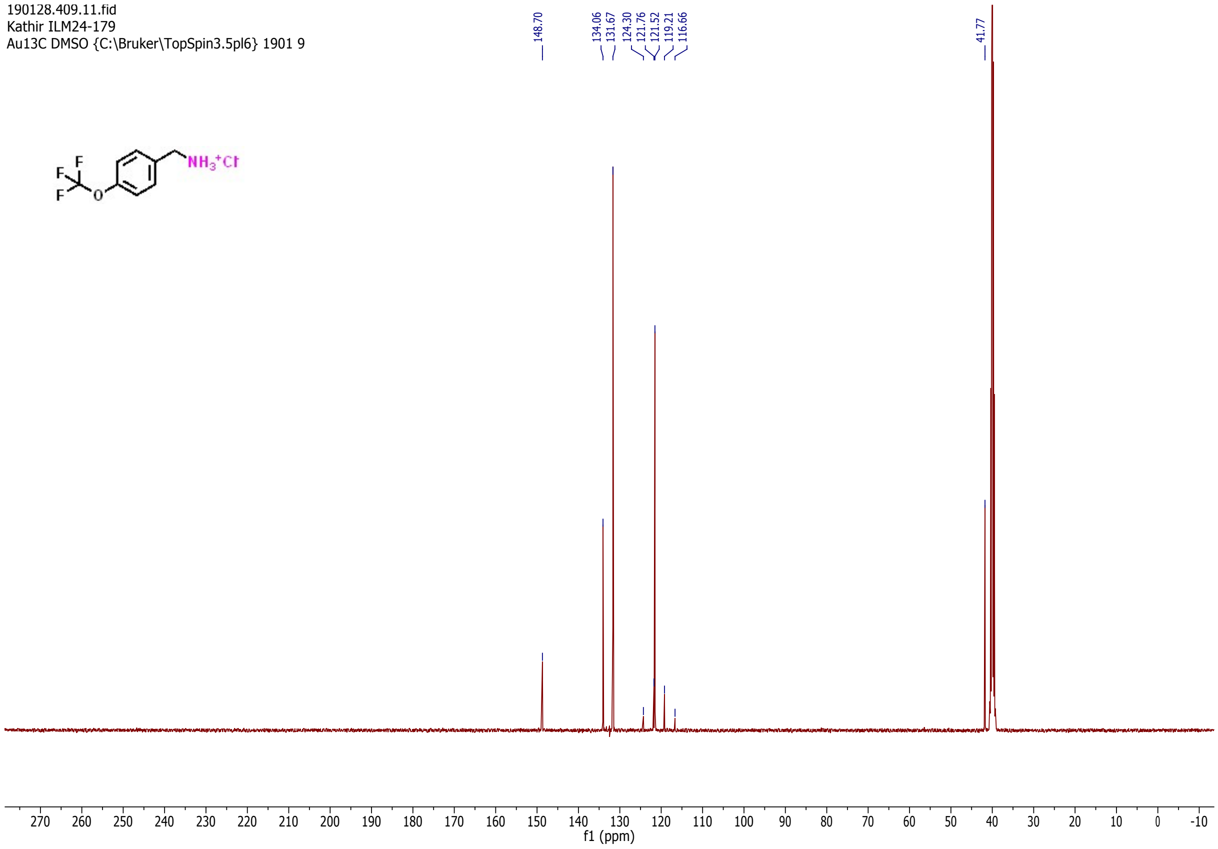

190128.418.10.fid
Kathir ILM24-86
Au1H DMSO {C:\Bruker\TopSpin3.5pl6} 1901 18

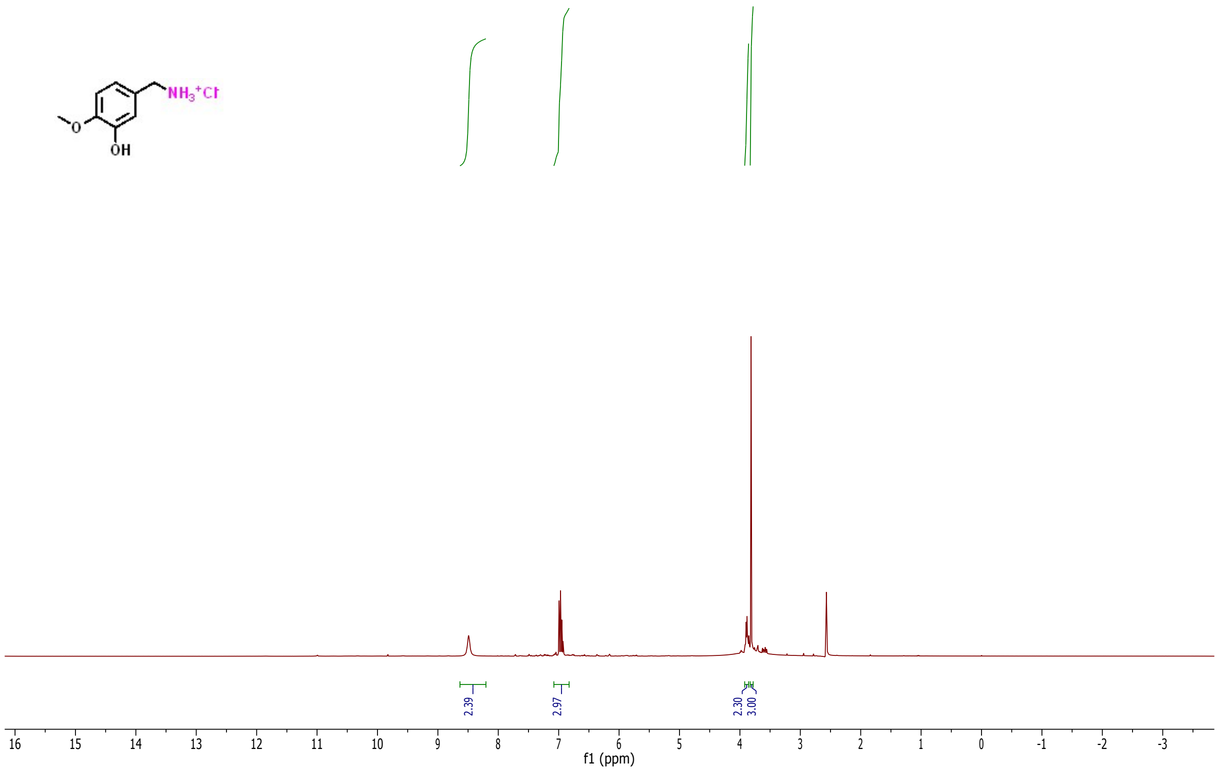

190128.418.11.fid
Kathir ILM24-86
Au13C DMSO {C:\Bruker\TopSpin3.5pl6} 1901 18

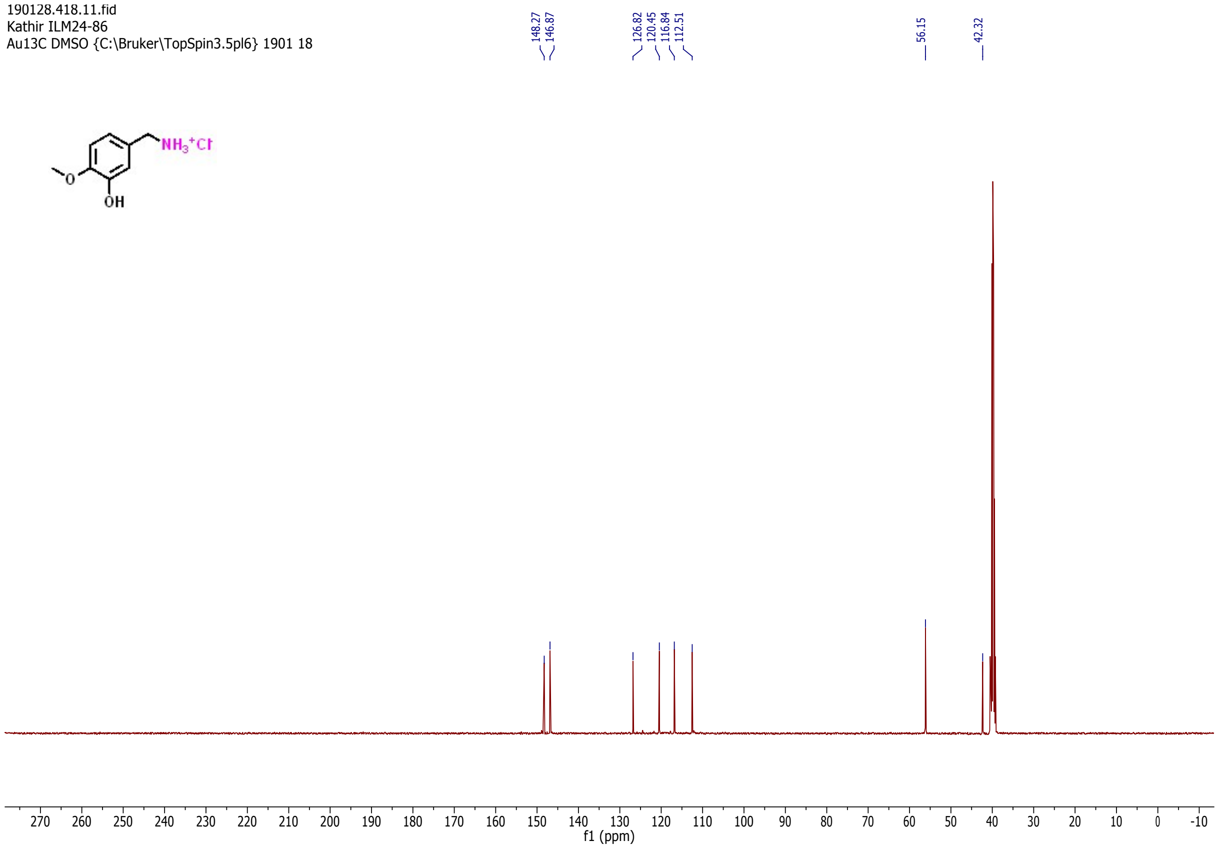

190128.402.10.fid
Kathir ILM24-141
Au1H DMSO {C:\Bruker\TopSpin3.5pl6} 1901 2

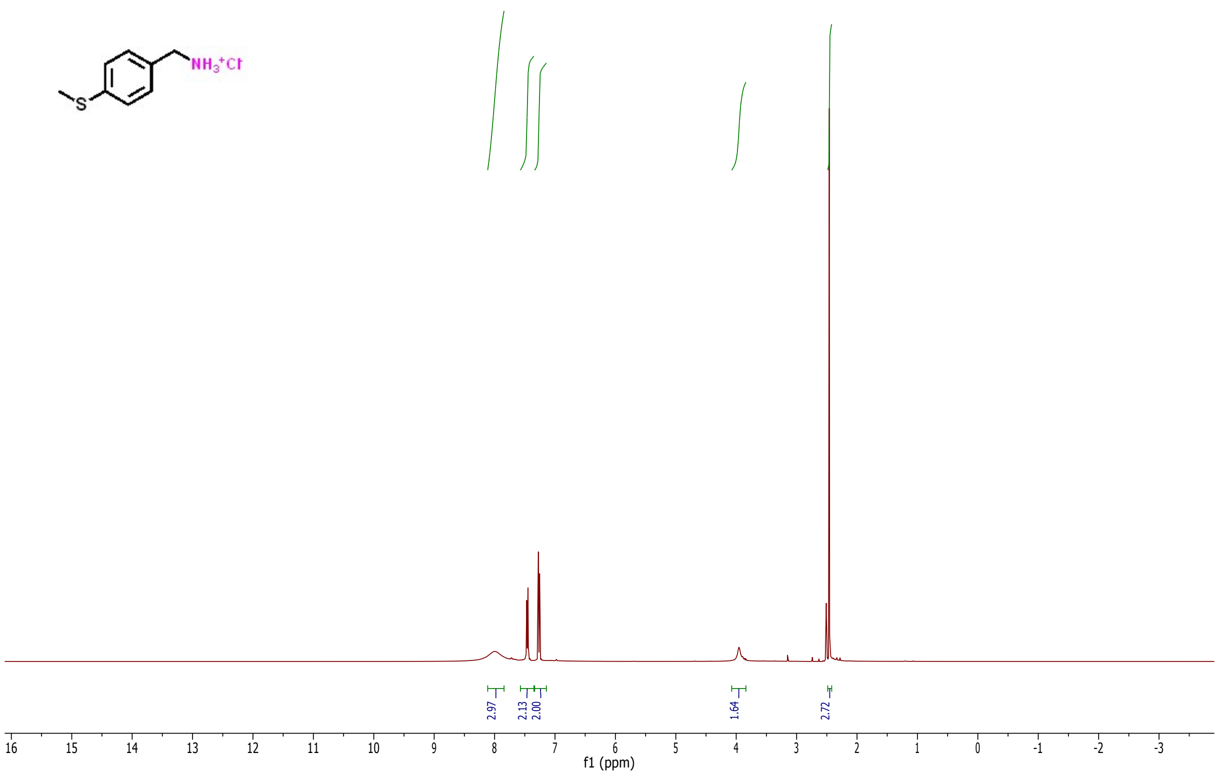

190128.402.11.fid
Kathir ILM24-141
Au13C DMSO {C:\Bruker\TopSpin3.5pl6} 1901 2

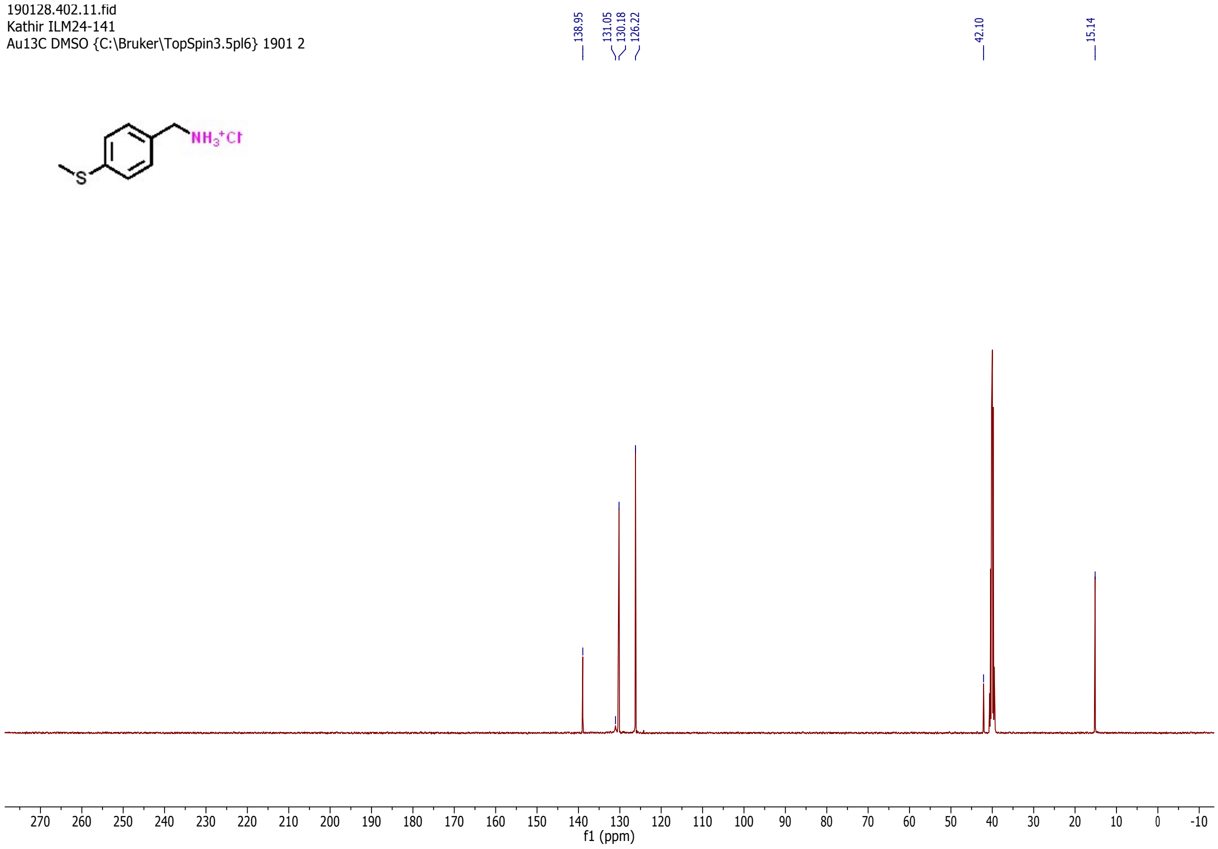

190128.403.10.fid
Kathir ILM24-68
Au1H DMSO {C:\Bruker\TopSpin3.5pl6} 1901 3

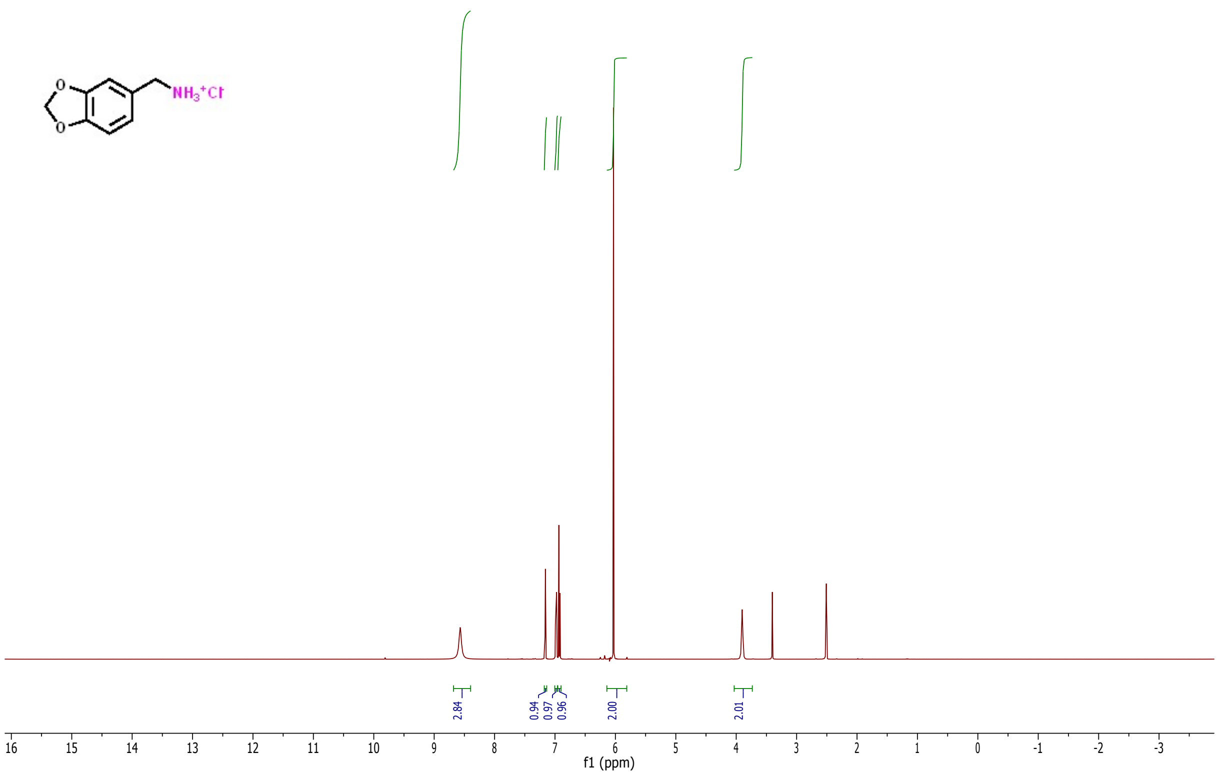

190128.403.11.fid
Kathir ILM24-68
Au13C DMSO {C:\Bruker\TopSpin3.5pl6} 1901 3

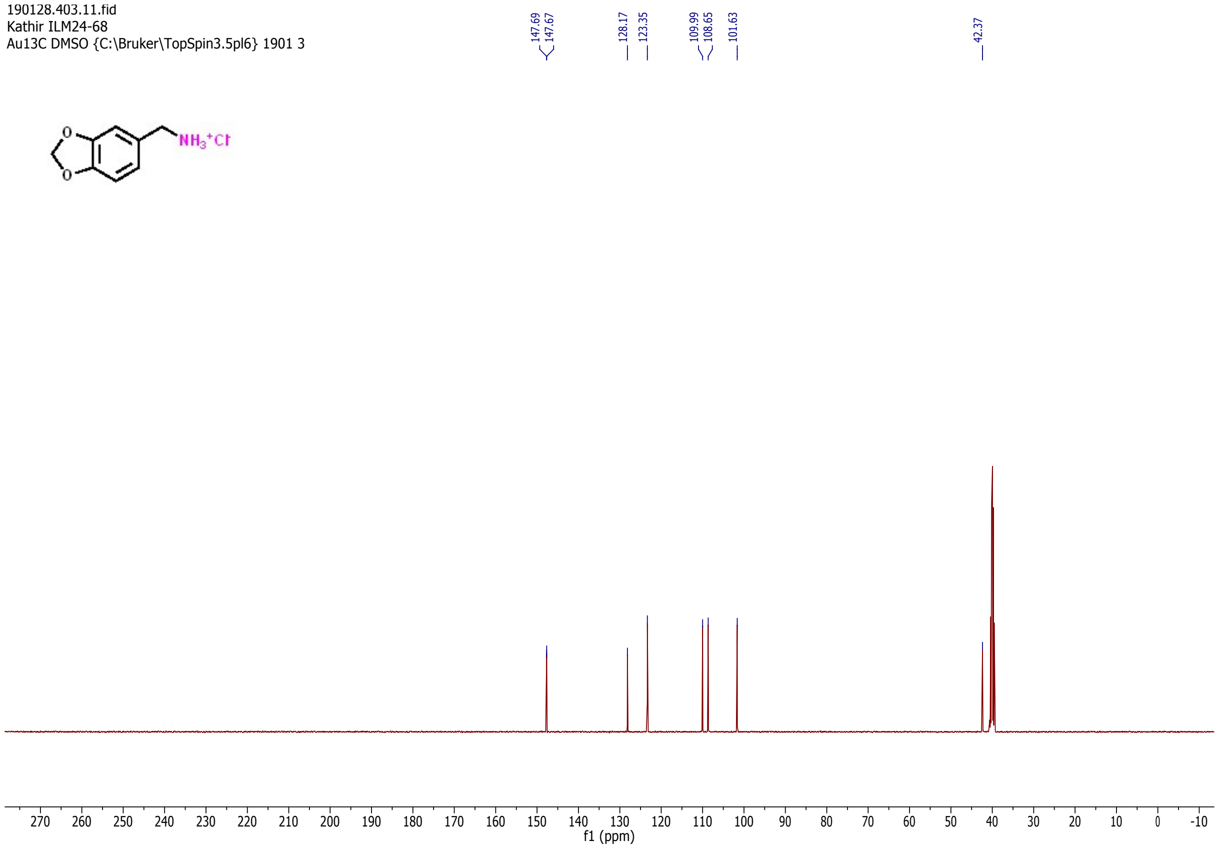

190128.410.10.fid
Kathir ILM24-75
Au1H DMSO {C:\Bruker\TopSpin3.5pl6} 1901 10

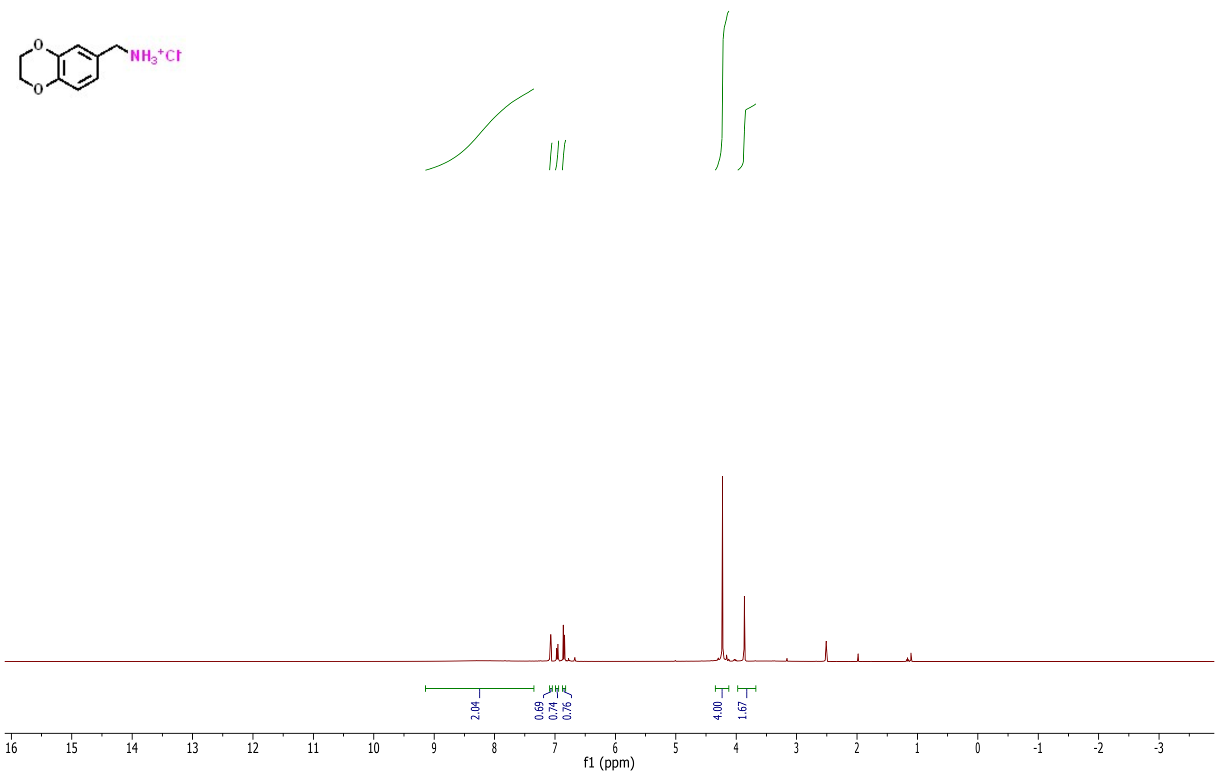

190128.410.11.fid
Kathir ILM24-75
Au13C DMSO {C:\Bruker\TopSpin3.5pl6} 1901 10

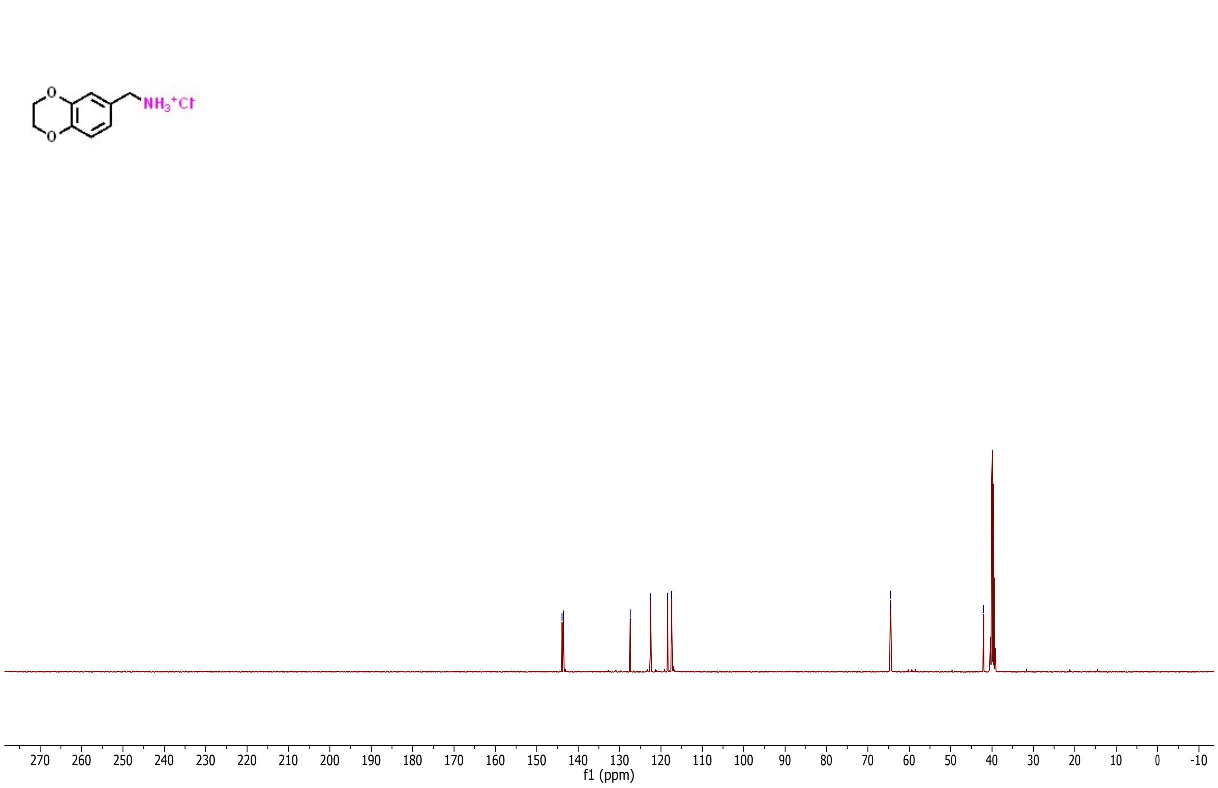

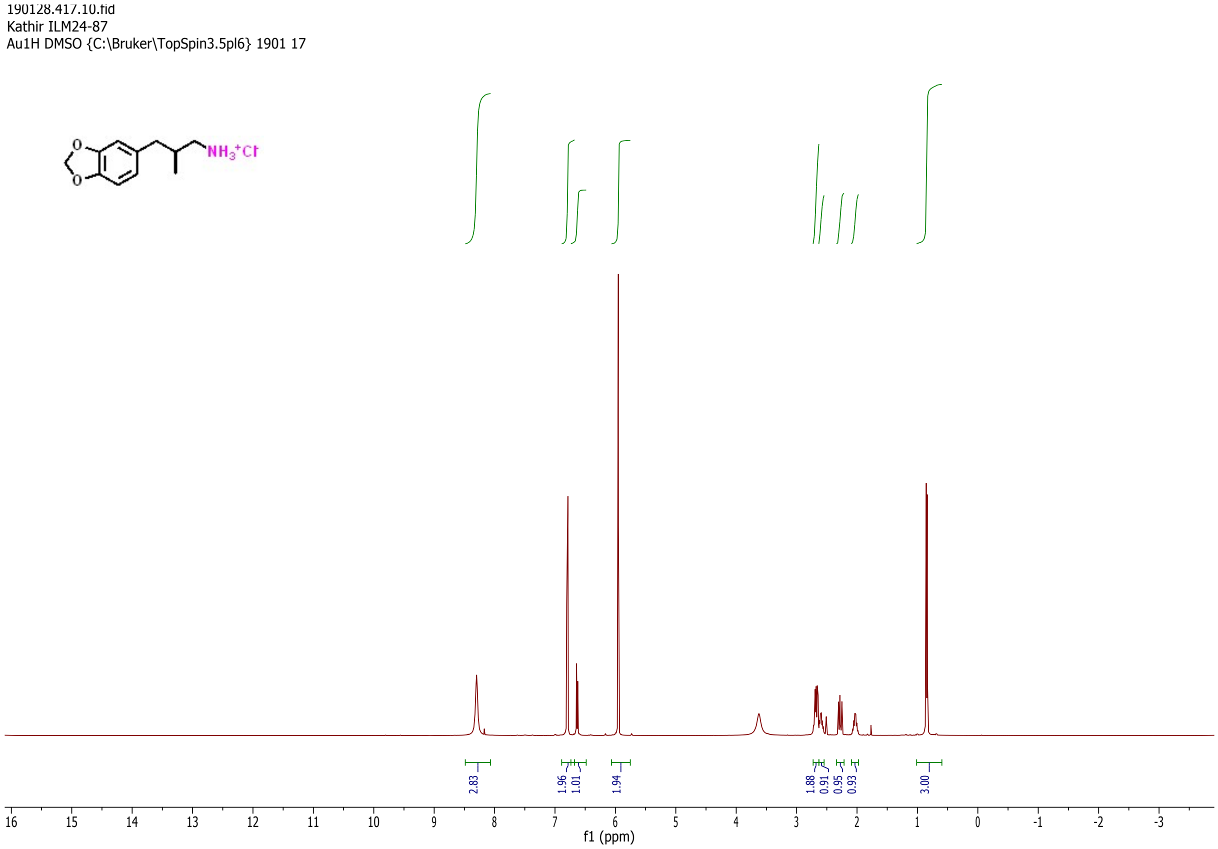
190128.417.10.fid
Kathir ILM24-87
Au1H DMSO {C:\Bruker\TopSpin3.5pl6} 1901 17
NH3+Cl-
2.83
1.96
1.01
1.94
1.88
0.91
0.95
0.93
3.00
f1 (ppm)

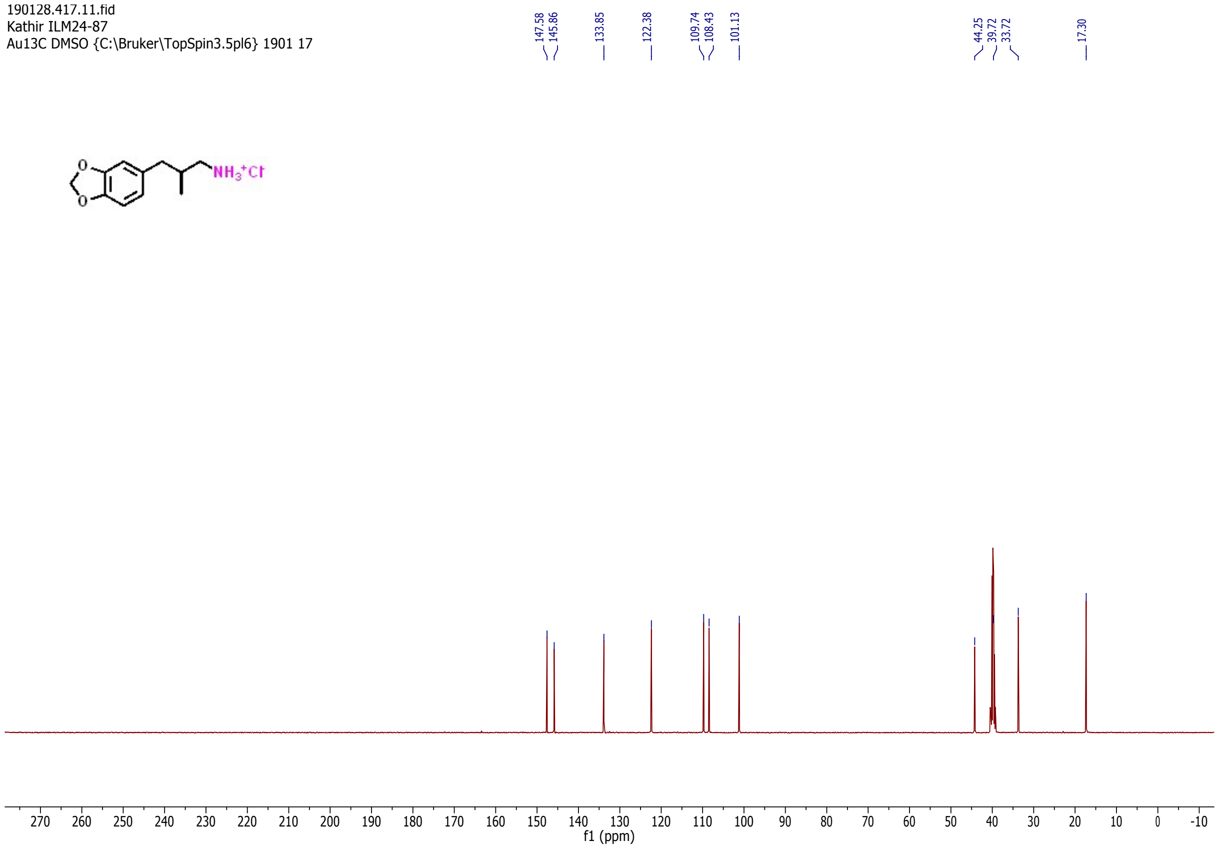
190128.417.11.fid
Kathir ILM24-87
Au13C DMSO {C:\Bruker\TopSpin3.5pl6} 1901 17
147.58
145.86
133.85
122.38
109.74
108.43
101.13
44.25
39.72
33.72
17.30
NH3+Cl-
f1 (ppm)

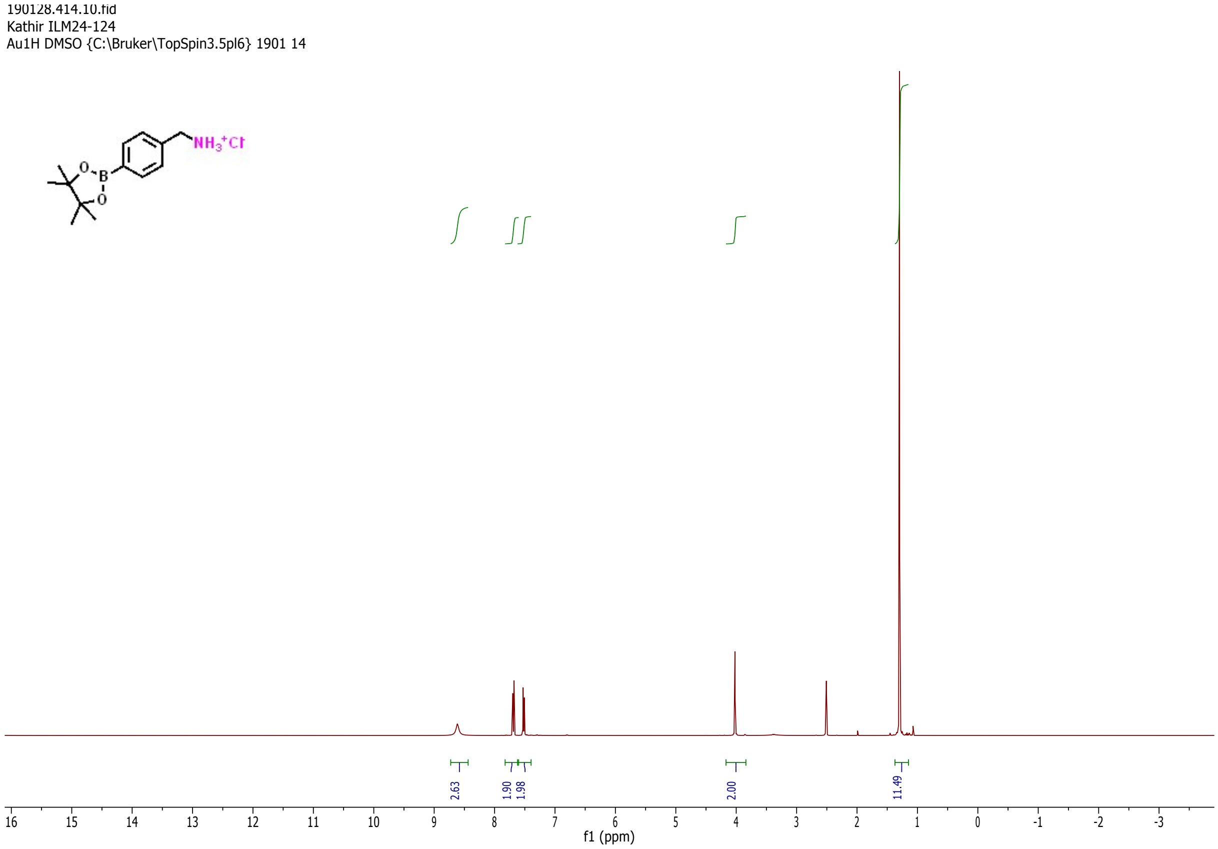
190128.414.10.fid
Kathir ILM24-124
Au1H DMSO {C:\Bruker\TopSpin3.5pl6} 1901 14
NH3+Cl-
2.63
1.90
1.98
2.00
11.49
f1 (ppm)

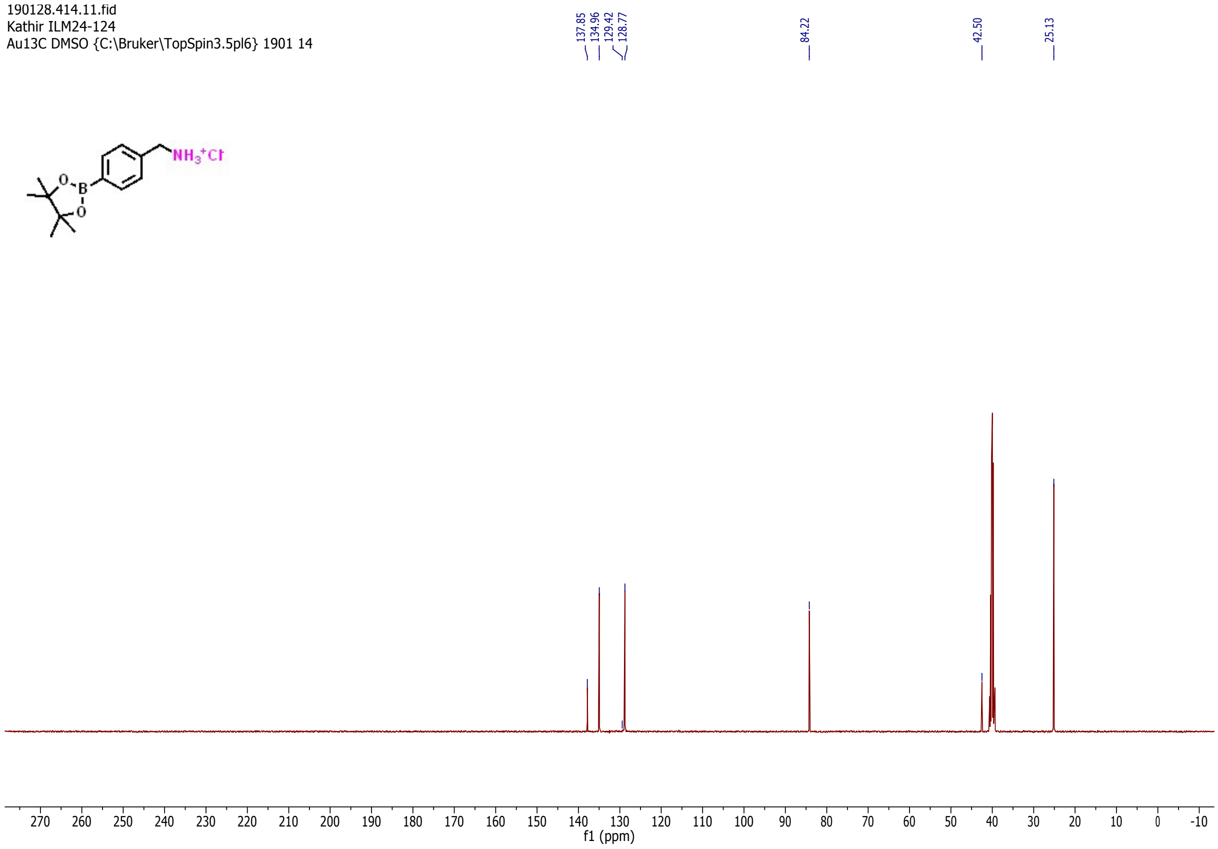
190128.414.11.fid
Kathir ILM24-124
Au13C DMSO {C:\Bruker\TopSpin3.5pl6} 1901 14
137.85
134.96
129.42
128.77
84.22
42.50
25.13
NH3+Cl-
f1 (ppm)

190128.f350.10.fid
Kathir KM24-98
PROTON DMSO {C:\Bruker\TopSpin3.6.0} 1901 50

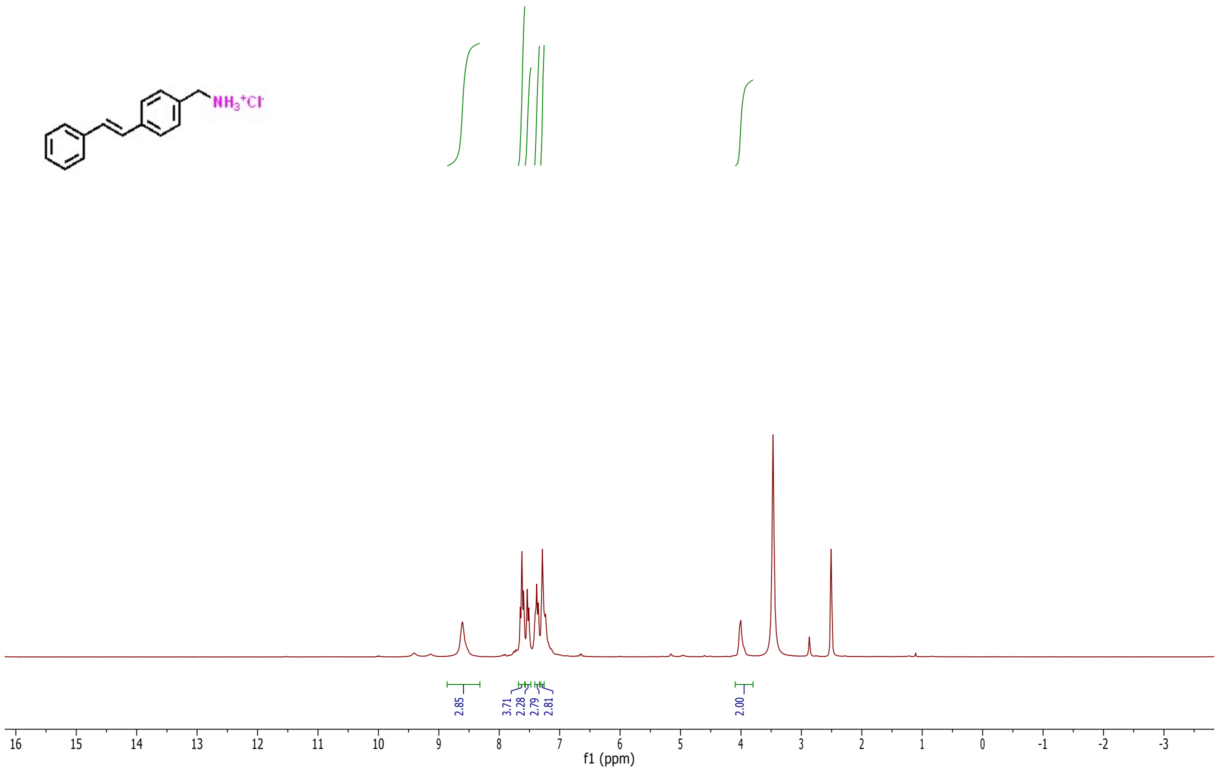

190128.f350.11.fid
Kathir KM24-98
C13CPD DMSO {C:\Bruker\TopSpin3.6.0} 1901 50

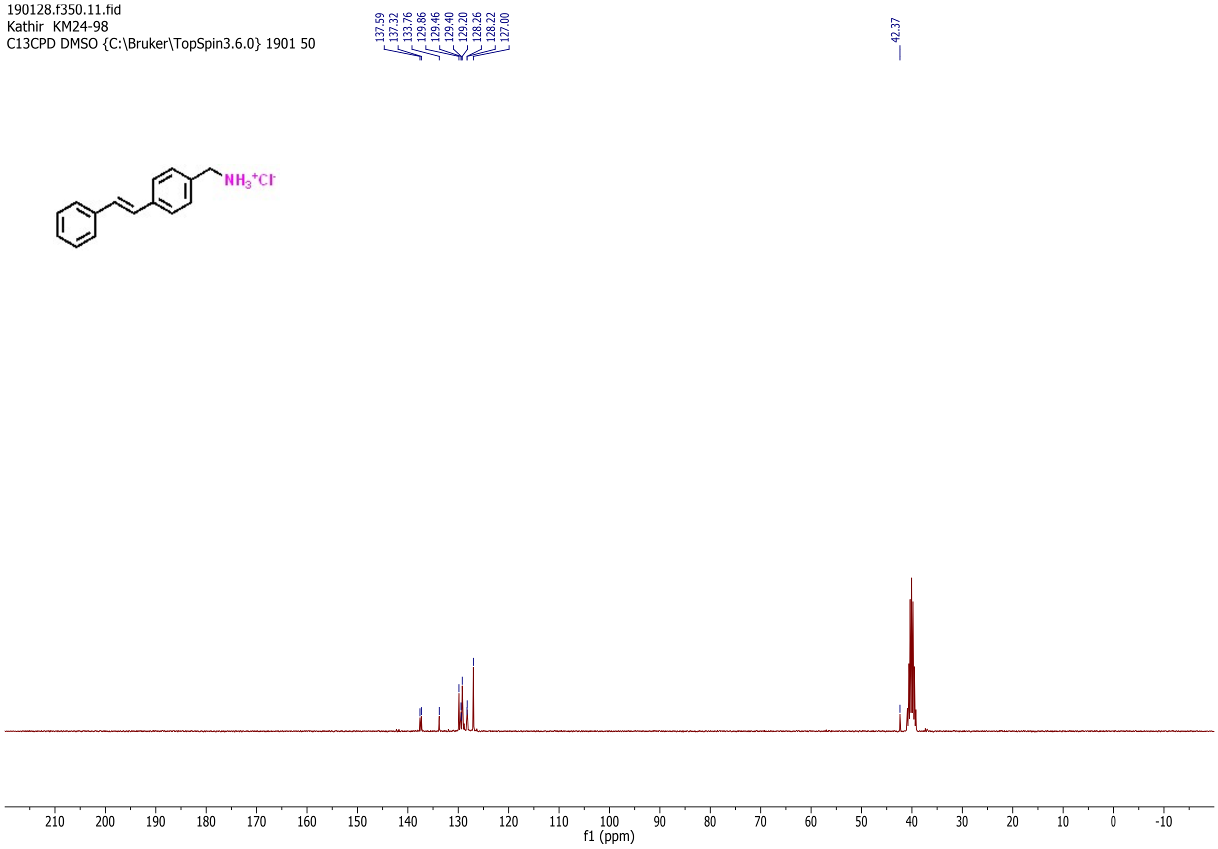

190128.415.10.fid
Kathir ILM24-126
Au1H DMSO {C:\Bruker\TopSpin3.5pl6} 1901 15

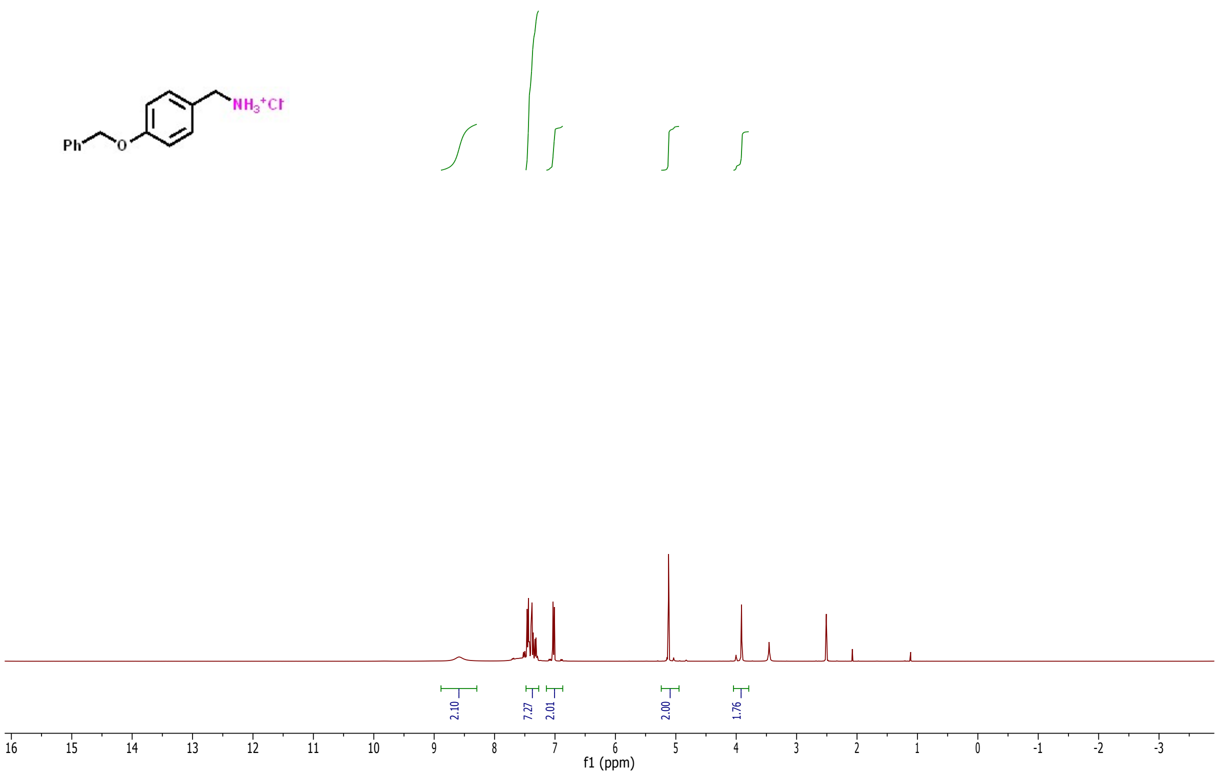

190128.415.11.fid
Kathir ILM24-126
Au13C DMSO {C:\Bruker\TopSpin3.5pl6} 1901 15

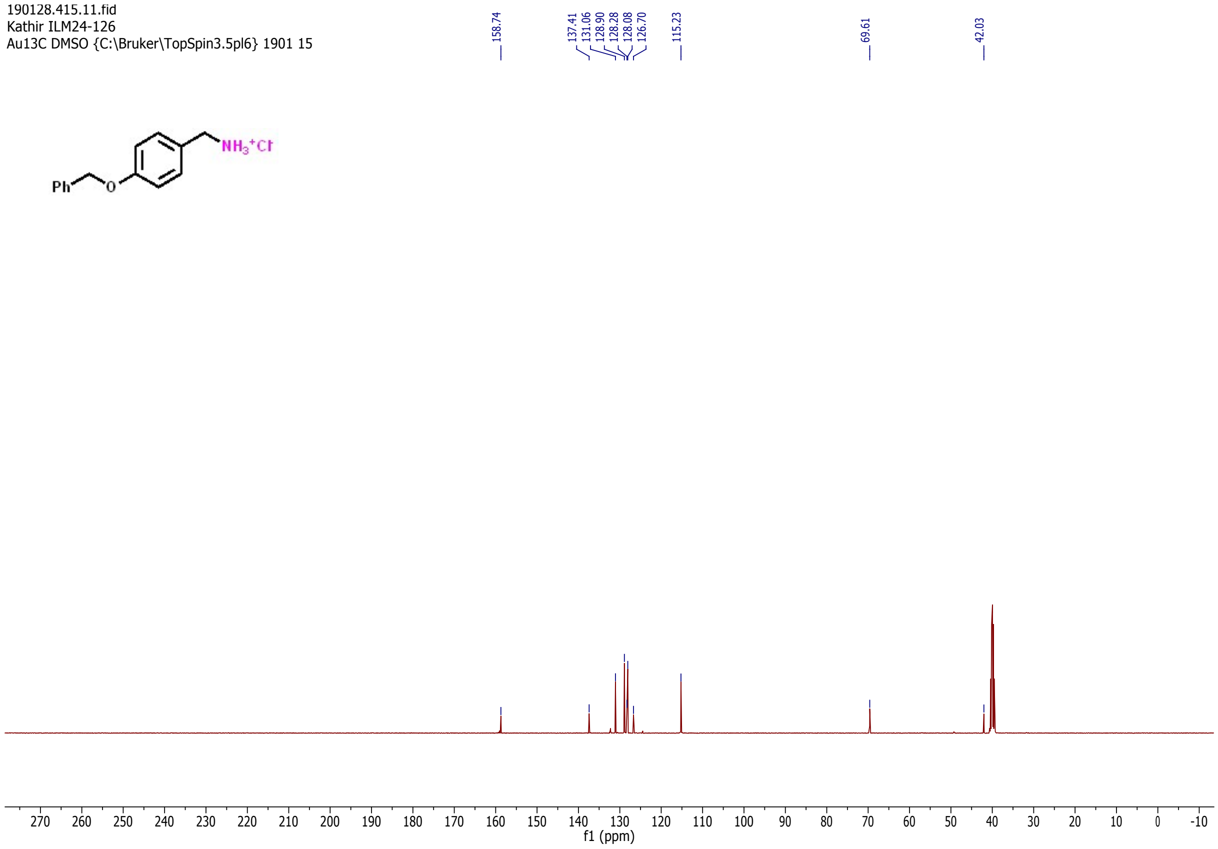

190128.413.10.fid
Kathir ILM24-128
Au1H DMSO {C:\Bruker\TopSpin3.5pl6} 1901 13

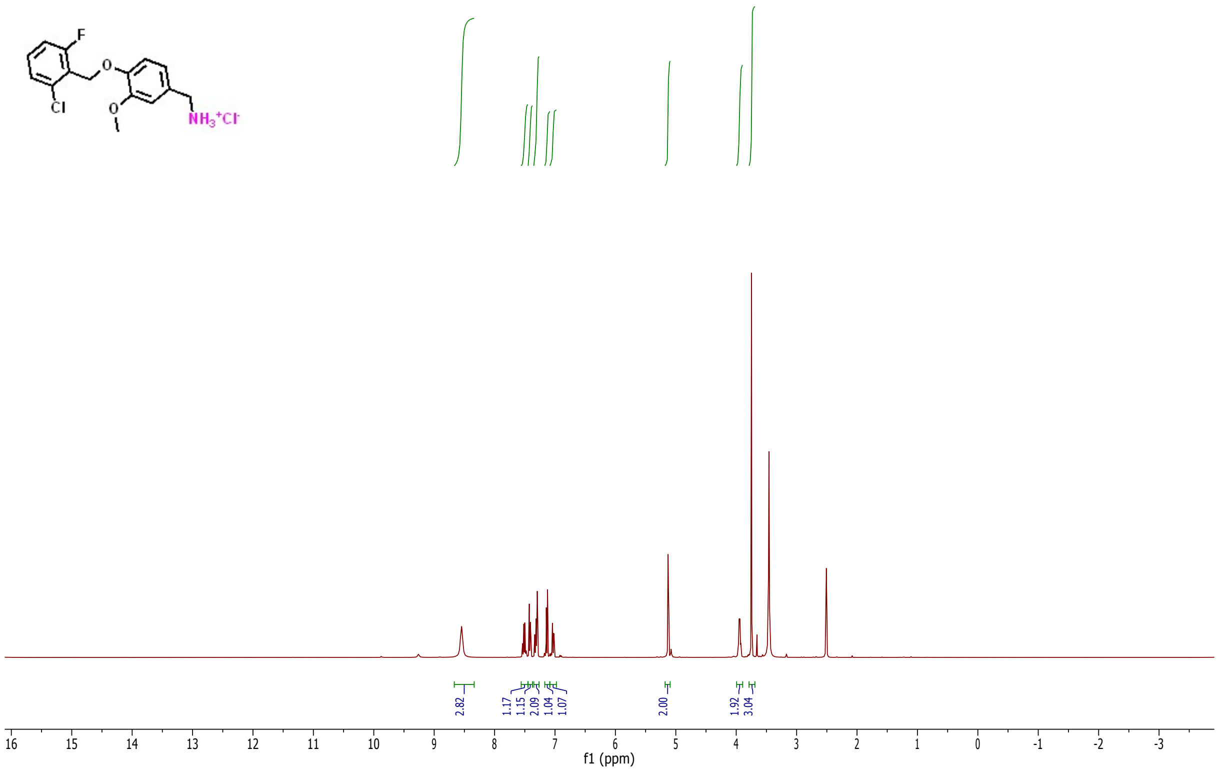

190128.413.11.fid
Kathir ILM24-128
Au13C DMSO {C:\Bruker\TopSpin3.5pl6} 1901 13

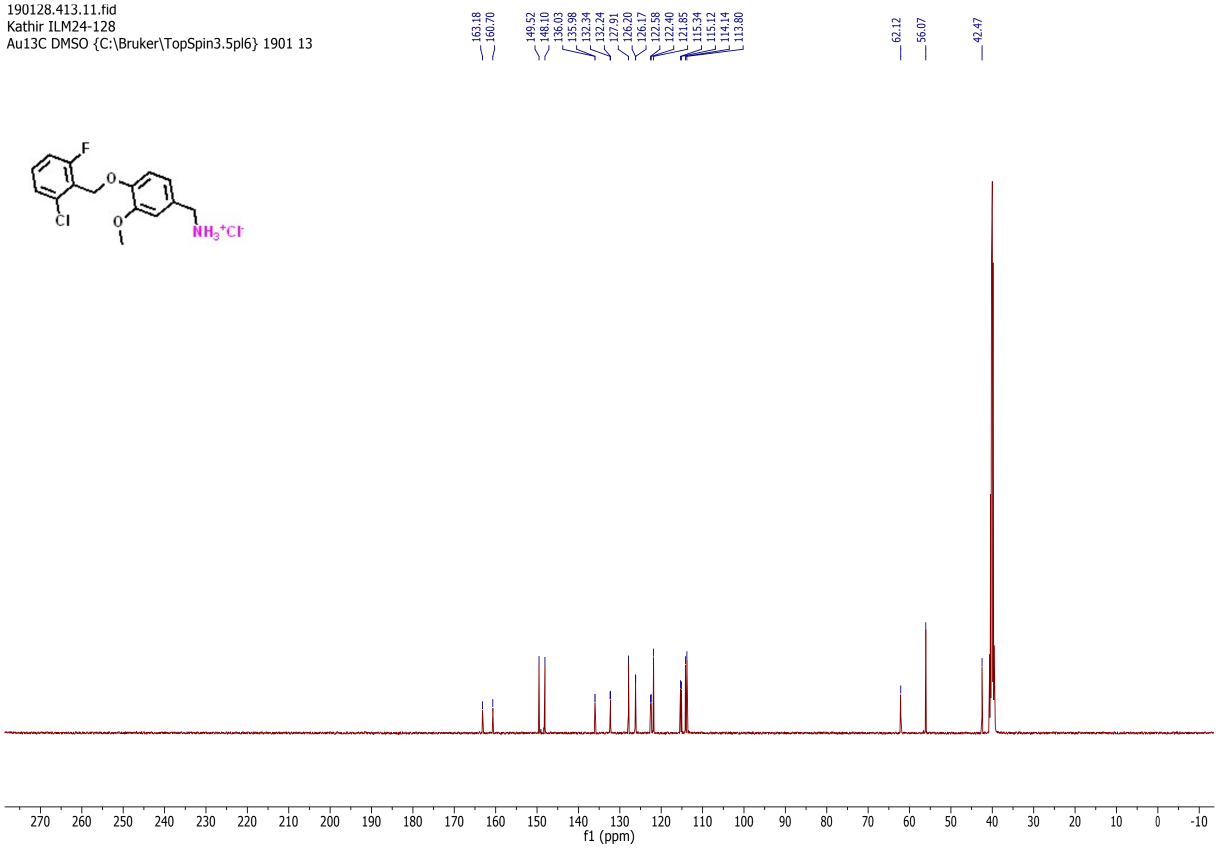

190128.405.10.fid
Kathir ILM24-108
Au1H DMSO {C:\Bruker\TopSpin3.5pl6} 1901 5

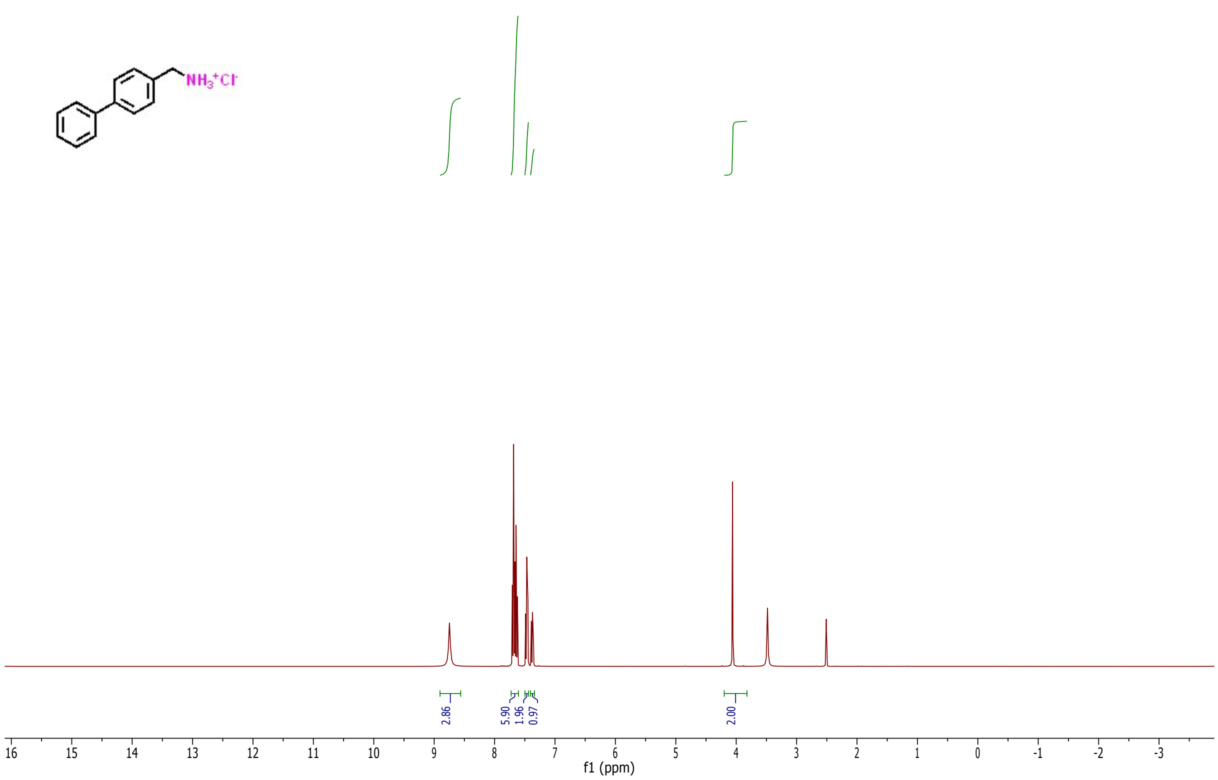

190128.405.11.fid
Kathir ILM24-108
Au13C DMSO {C:\Bruker\TopSpin3.5pl6} 1901 5

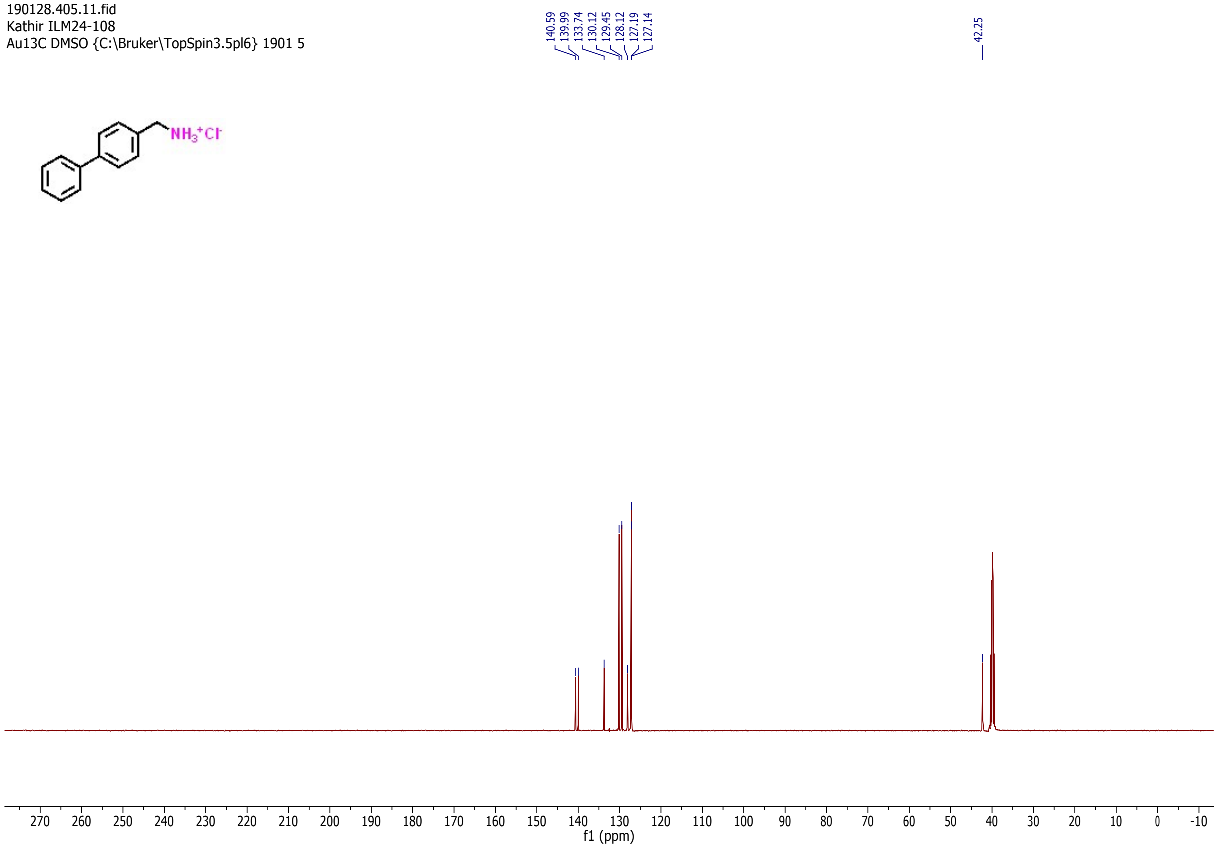

190128.416.10.fid
Kathir ILM24-180
Au1H DMSO {C:\Bruker\TopSpin3.5pl6} 1901 16

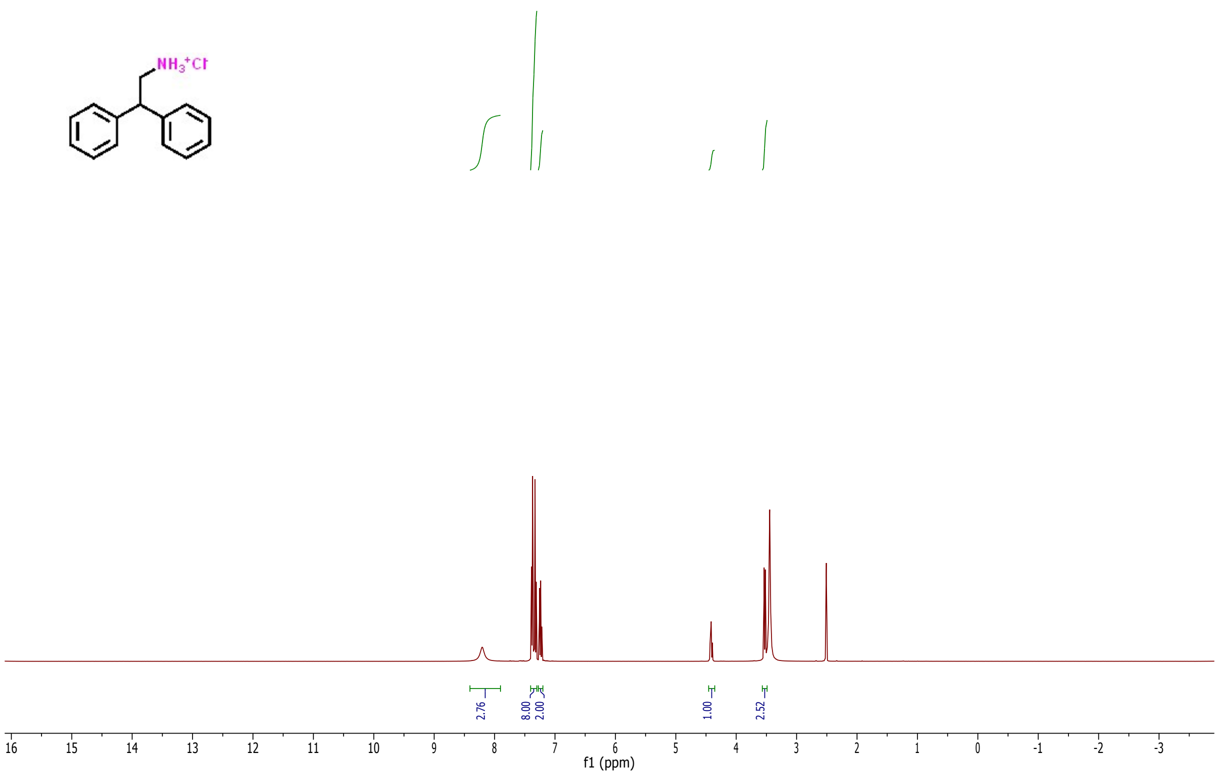

190128.416.11.fid
Kathir ILM24-180
Au13C DMSO {C:\Bruker\TopSpin3.5pl6} 1901 16

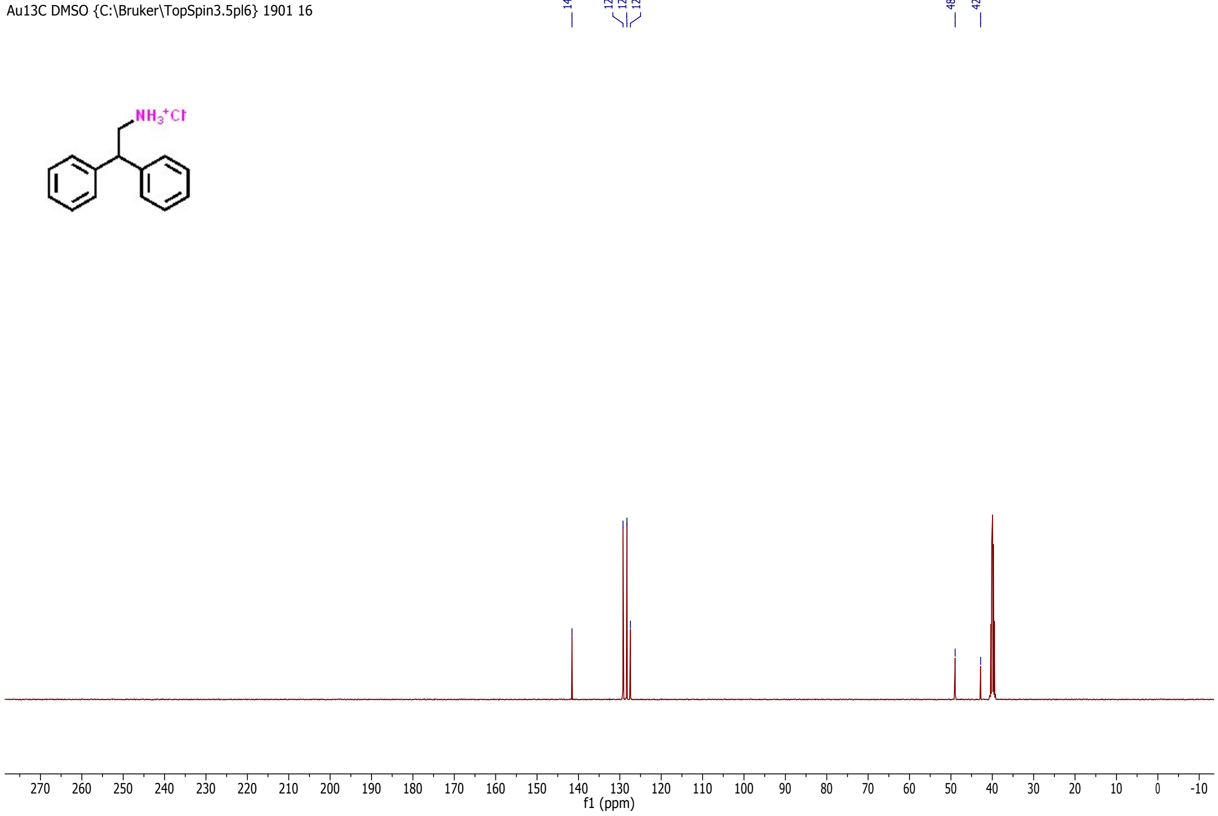

190128.412.10.fid
Kathir ILM24-100
Au1H DMSO {C:\Bruker\TopSpin3.5pl6} 1901 12

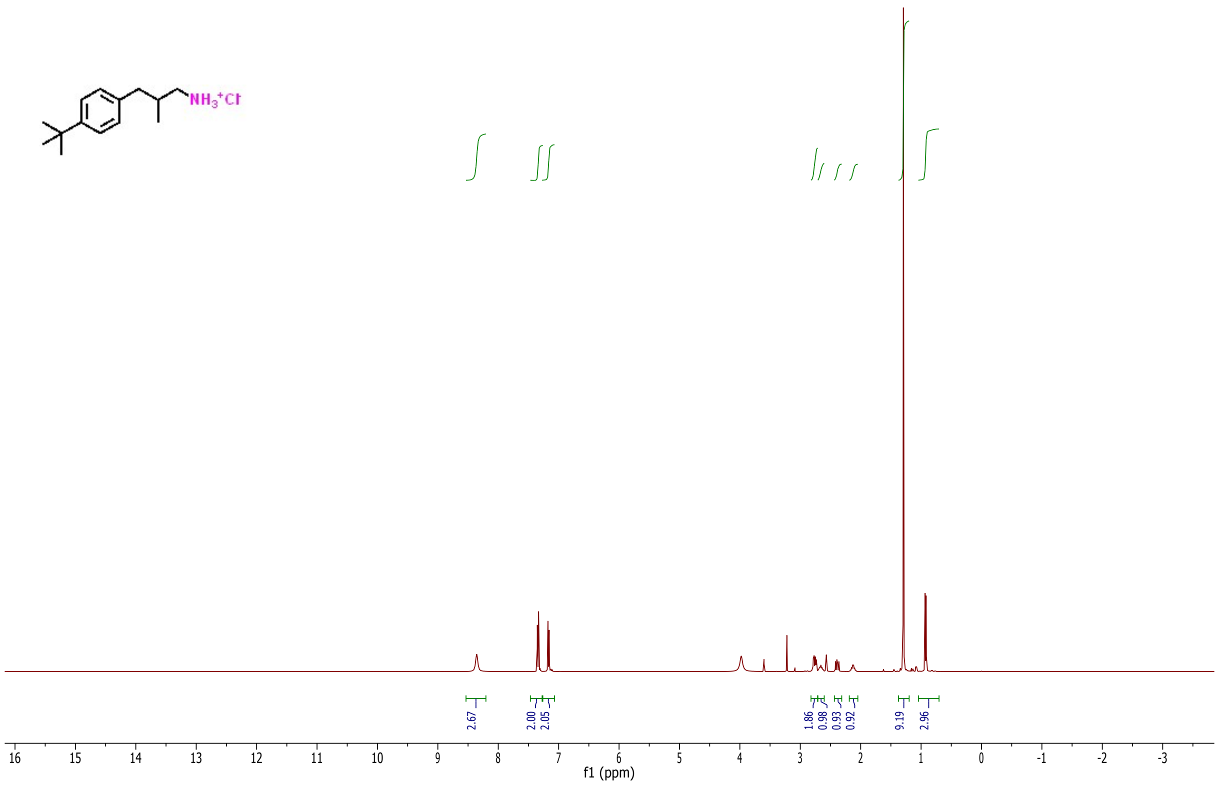

190128.412.11.fid
Kathir ILM24-100
Au13C DMSO {C:\Bruker\TopSpin3.5pl6} 1901 12

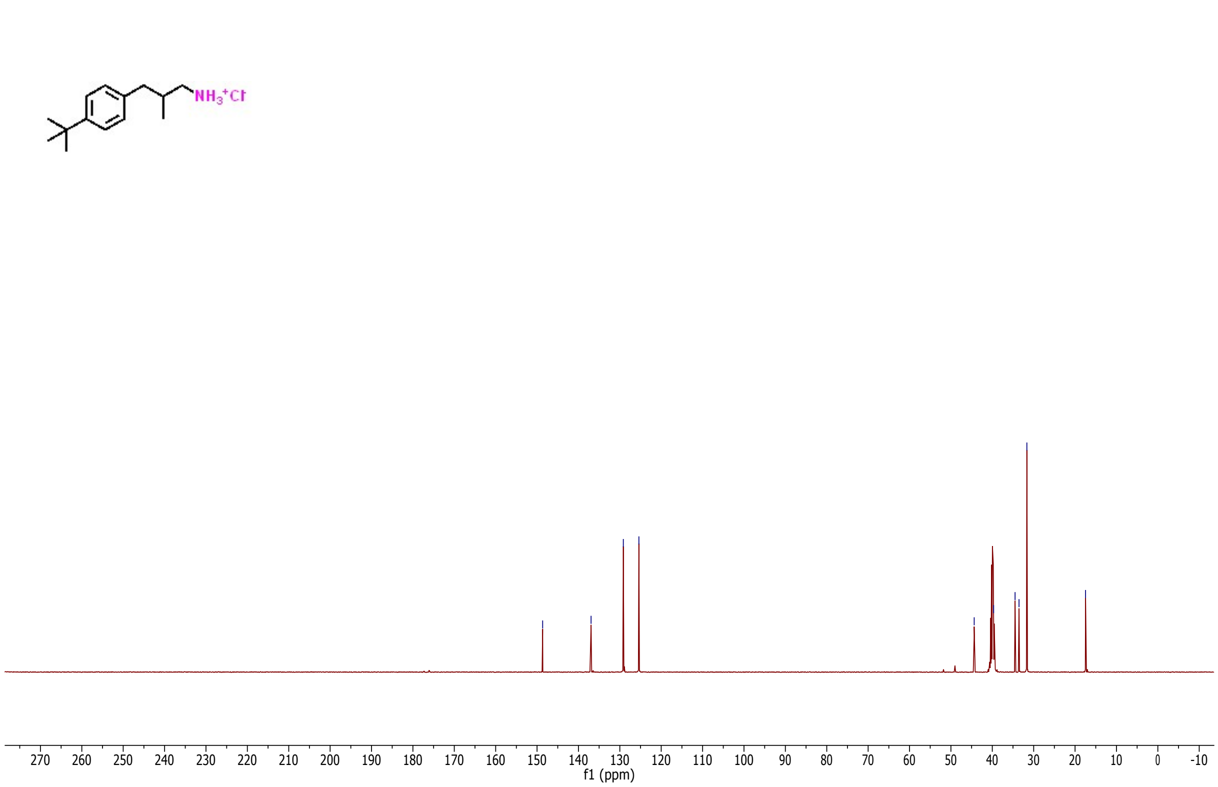

190128.313.10.fid
Kathir KM24-151
Au1H DMSO {C:\Bruker\TopSpin3.6.0} 1901 13

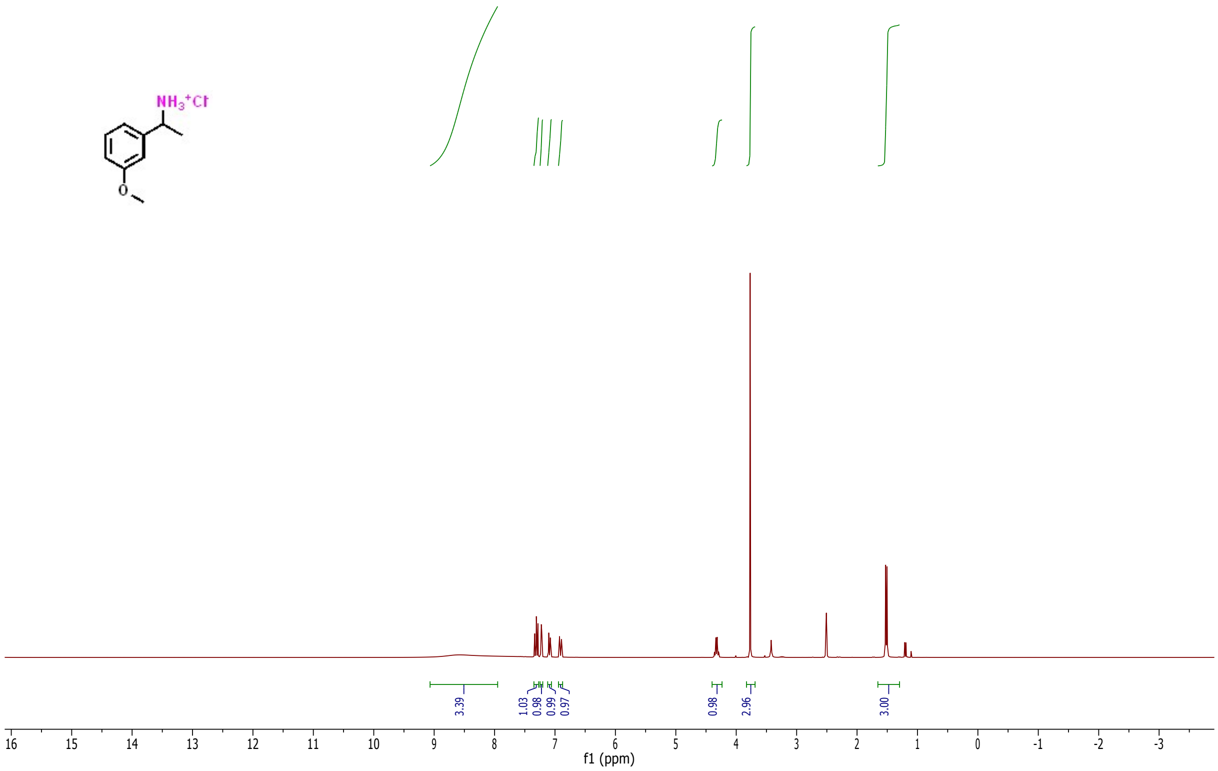

190128.313.11.fid
Kathir KM24-151
Au13C DMSO {C:\Bruker\TopSpin3.6.0} 1901 13

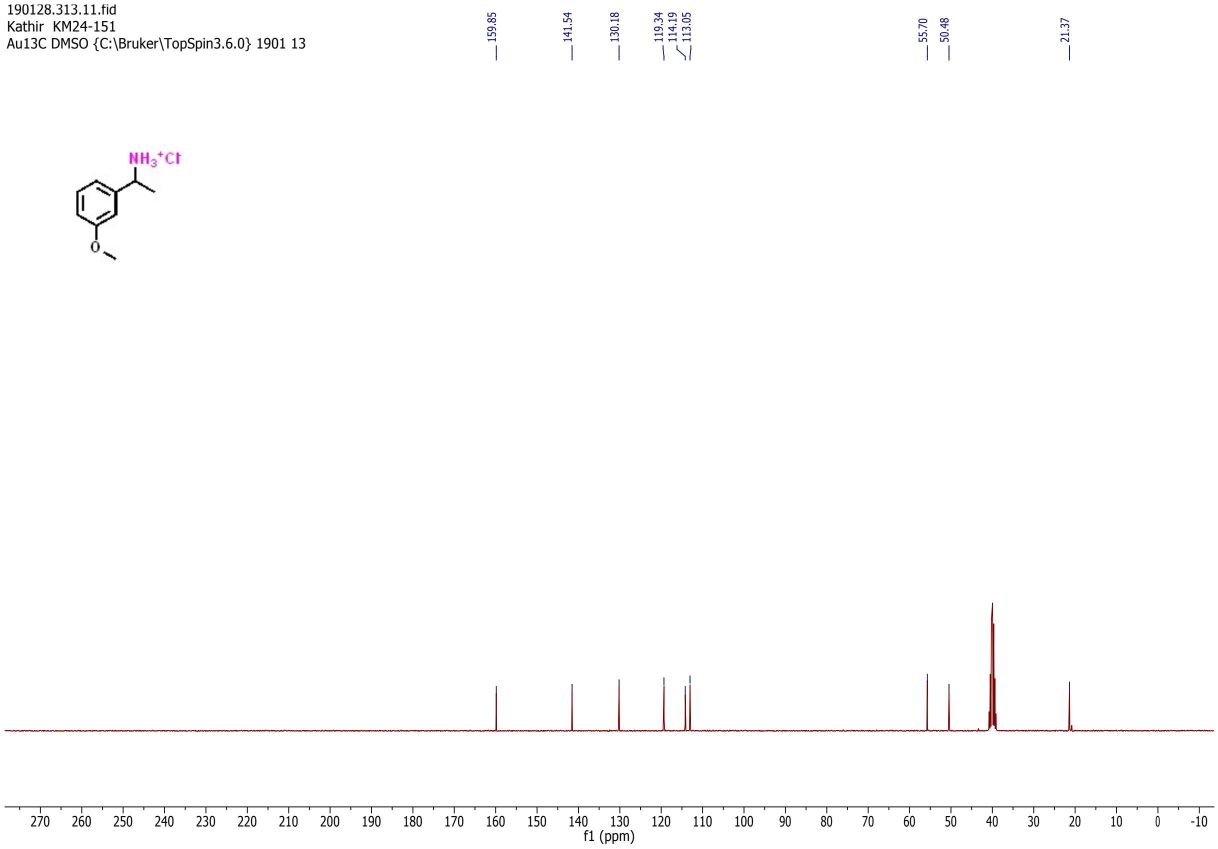

190128.312.10.fid
Kathir KM24-150
Au1H DMSO {C:\Bruker\TopSpin3.6.0} 1901 12

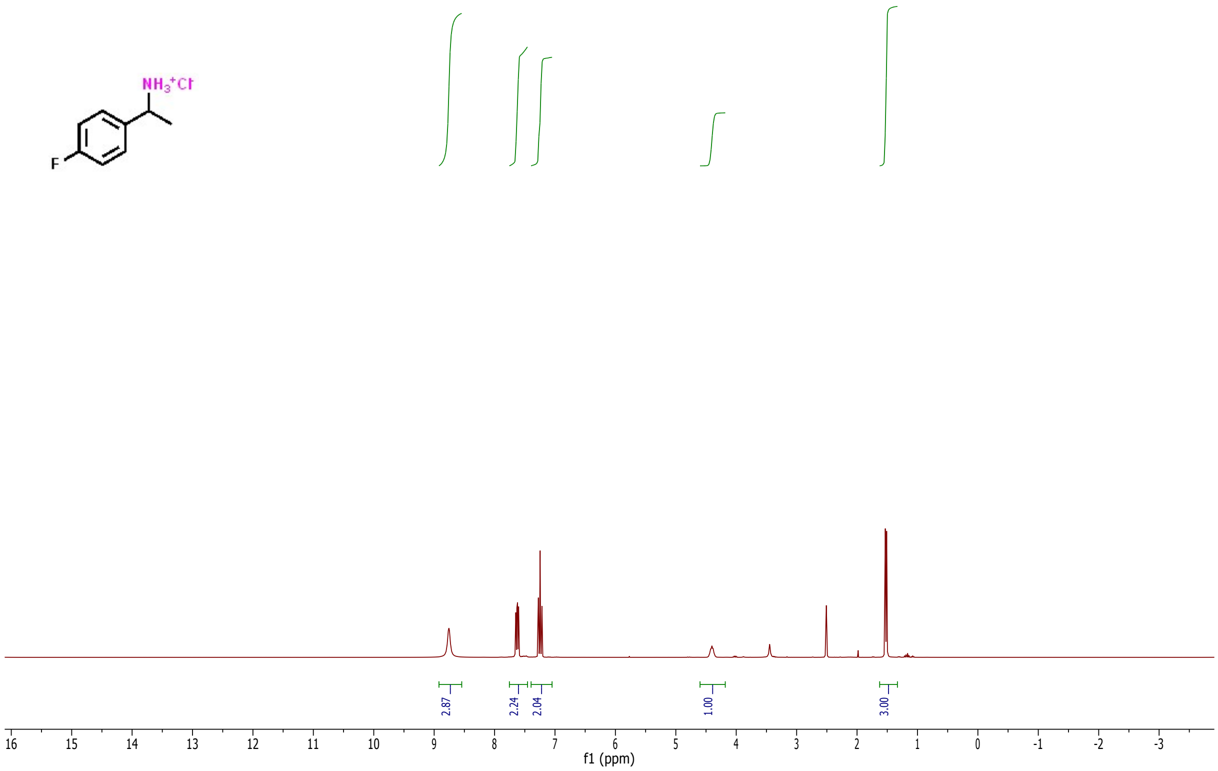

190128.312.11.fid
Kathir KM24-150
Au13C DMSO {C:\Bruker\TopSpin3.6.0} 1901 12

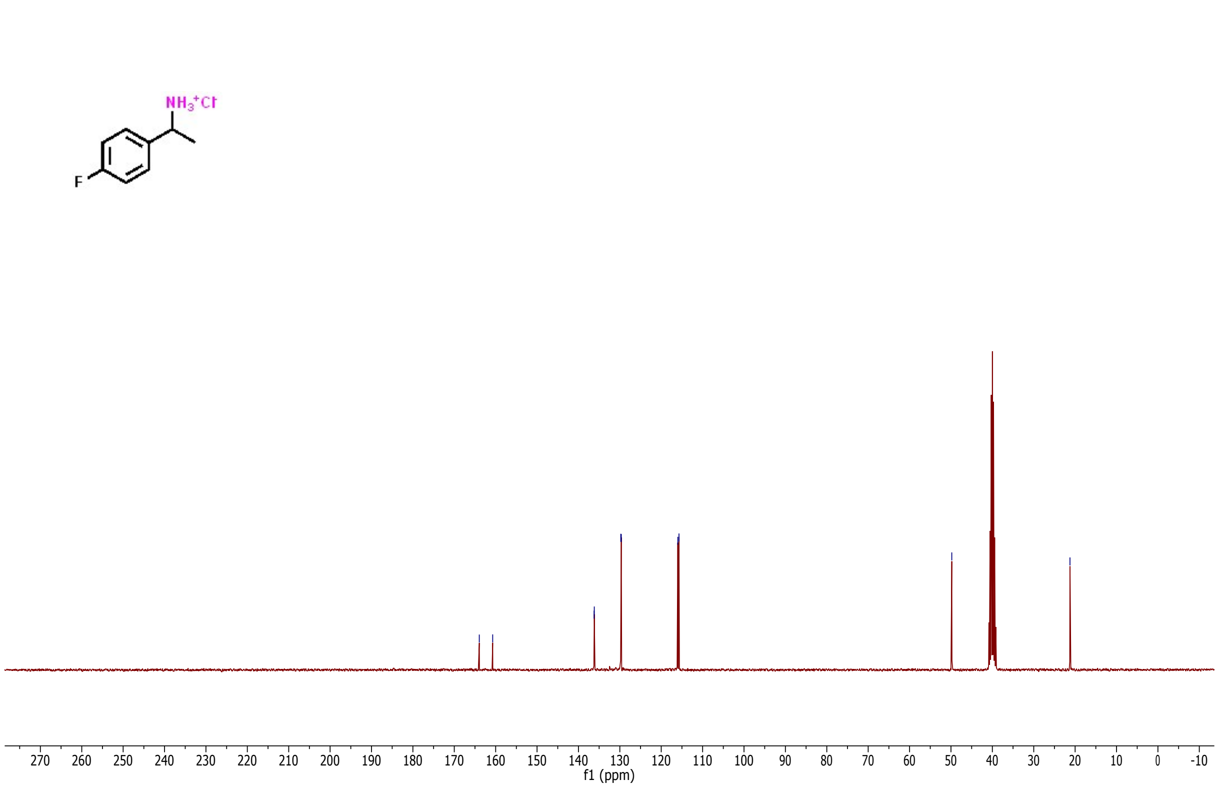

190128.321.10.fid
Kathir KM24-199
Au1H DMSO {C:\Bruker\TopSpin3.6.0} 1901 21

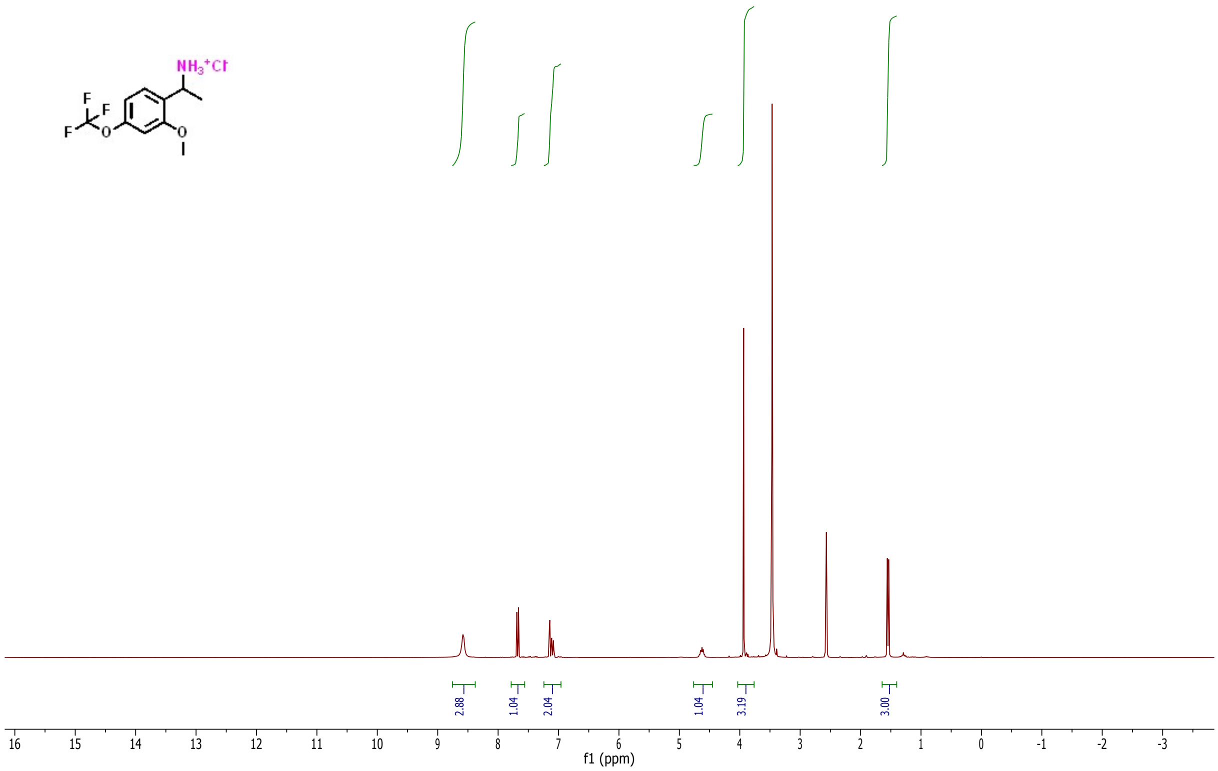

190128.321.11.fid
Kathir KM24-199
Au13C DMSO {C:\Bruker\TopSpin3.6.0} 1901 21

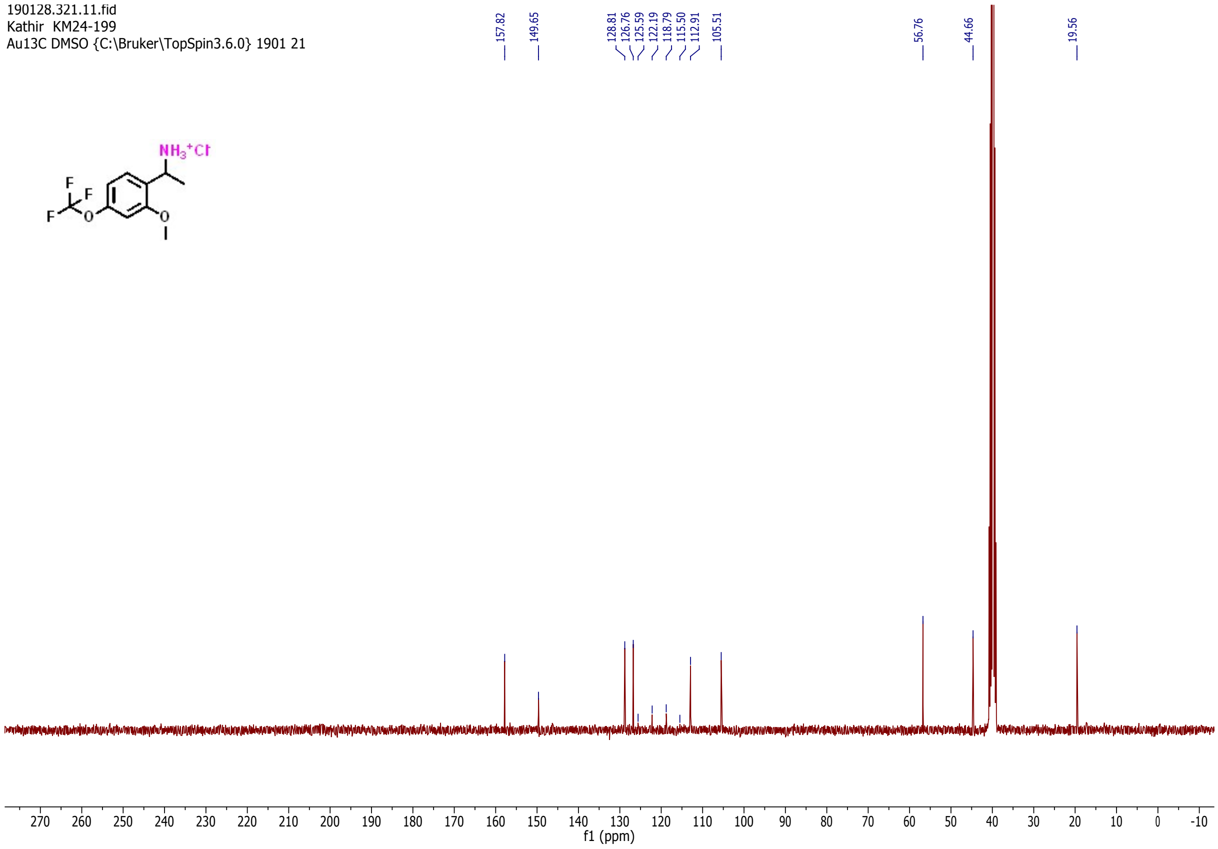

190121.327.10.fid
Kathir KM24-154
Au1H DMSO {C:\Bruker\TopSpin3.6.0} 1901 27

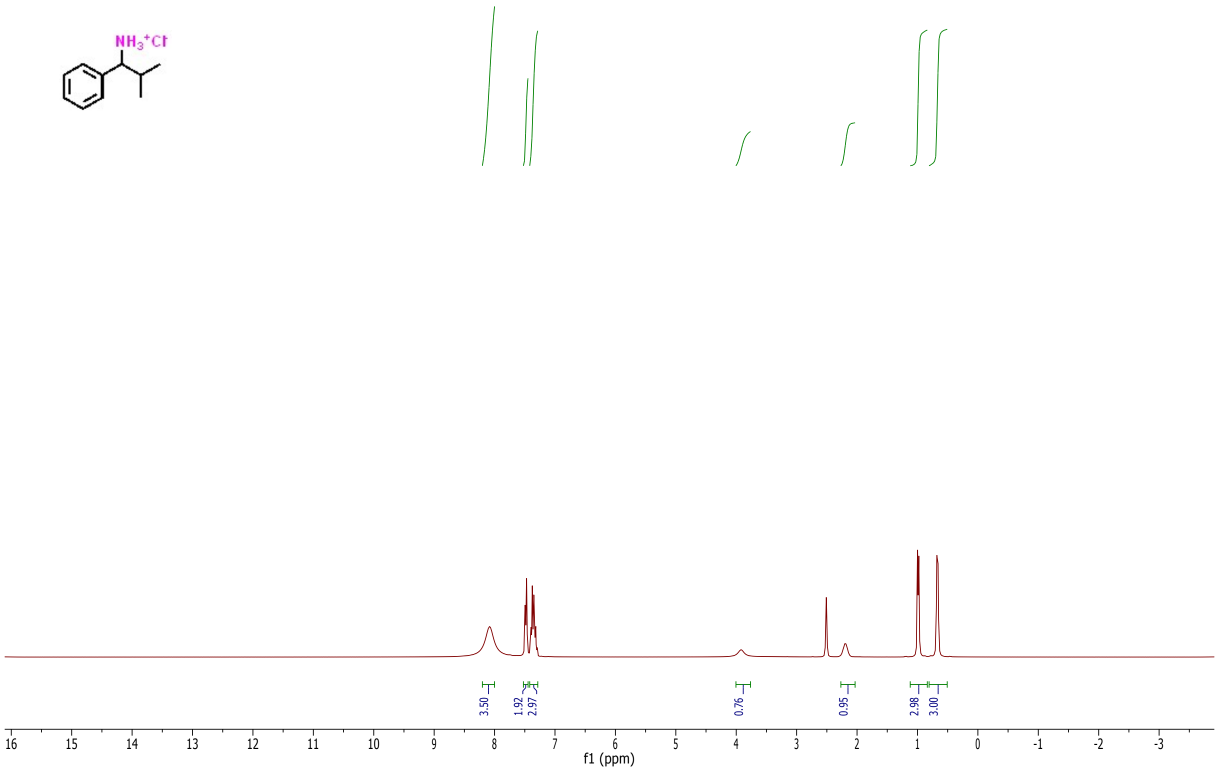

190121.327.11.fid
Kathir KM24-154
Au13C DMSO {C:\Bruker\TopSpin3.6.0} 1901 27

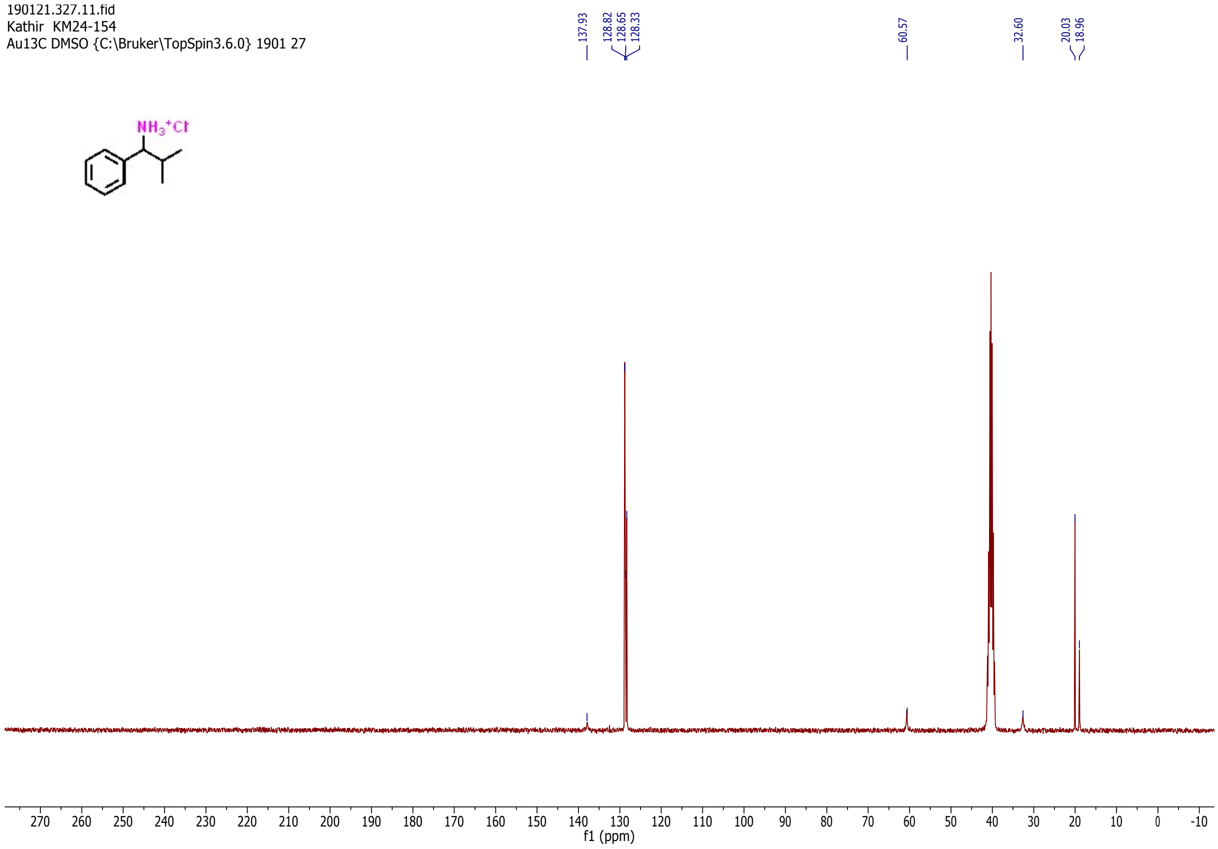

190128.311.10.fid
Kathir KM24-148
Au1H DMSO {C:\Bruker\TopSpin3.6.0} 1901 11

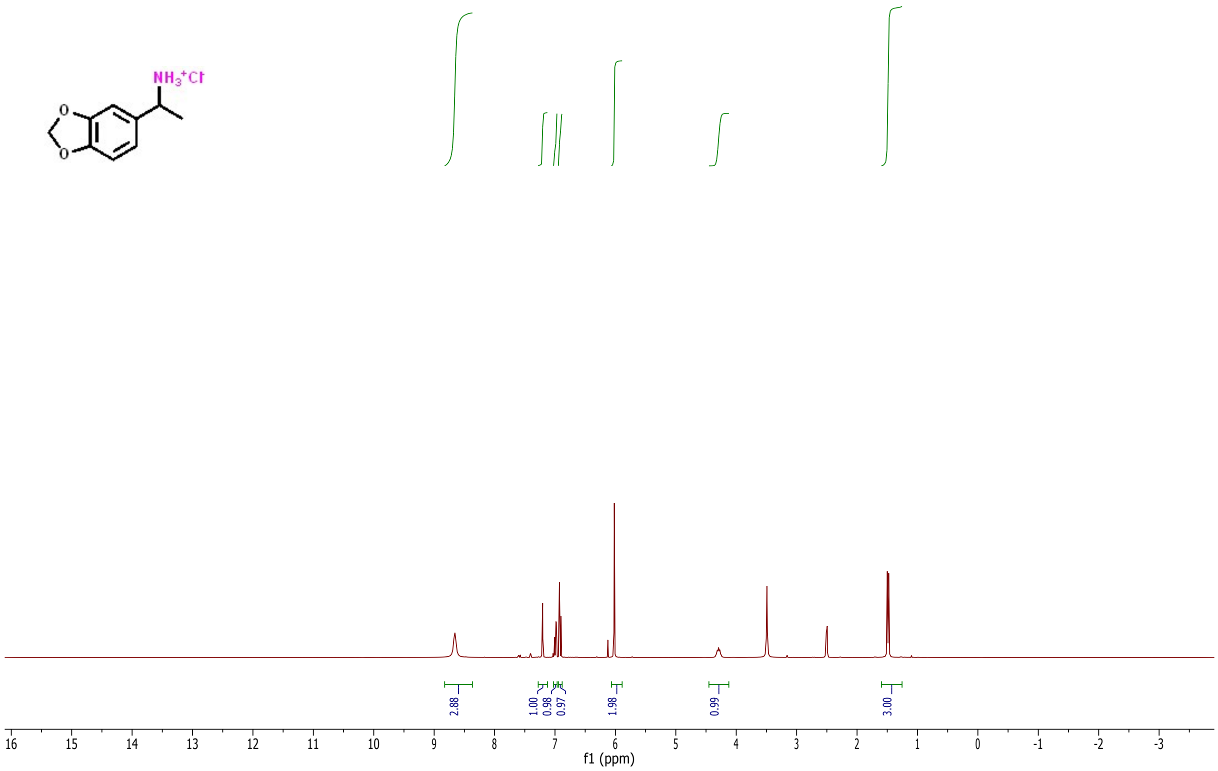

190128.311.11.fid
Kathir KM24-148
Au13C DMSO {C:\Bruker\TopSpin3.6.0} 1901 11

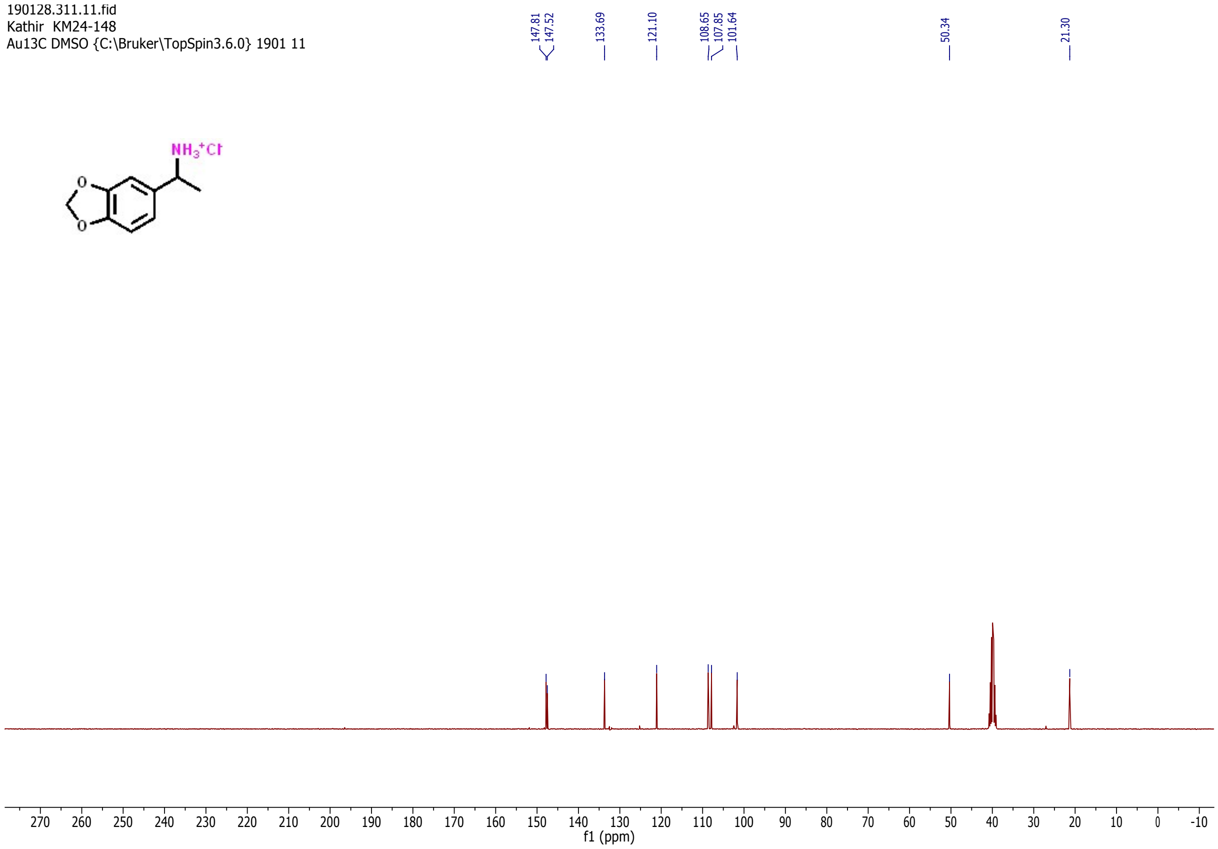

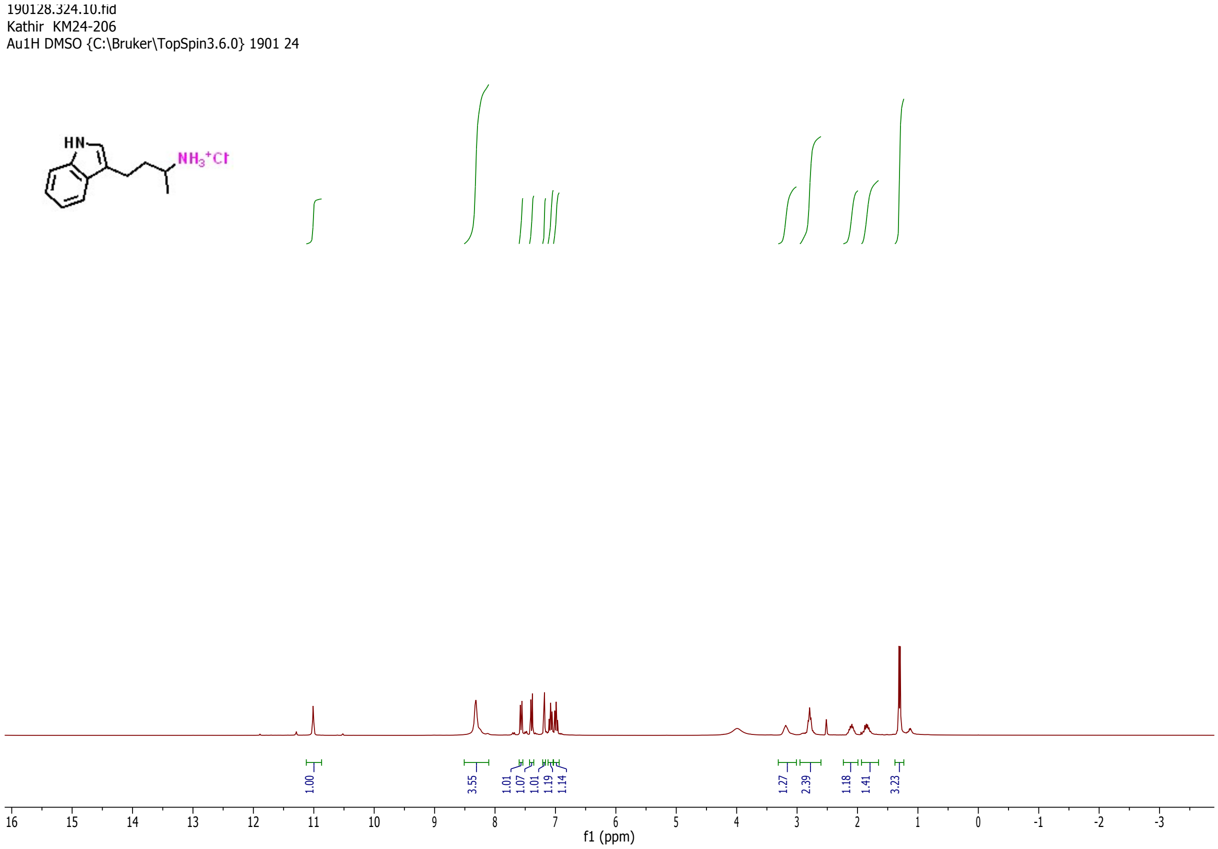
190128.324.10.fid
Kathir KM24-206
Au1H DMSO {C:\Bruker\TopSpin3.6.0} 1901 24
HN
$NH_3^+Cl^-$
1.00
3.55
1.01
1.07
1.01
1.19
1.14
1.27
2.39
1.18
1.41
3.23
16 15 14 13 12 11 10 9 8 7 6 5 4 3 2 1 0 -1 -2 -3
f1 (ppm)

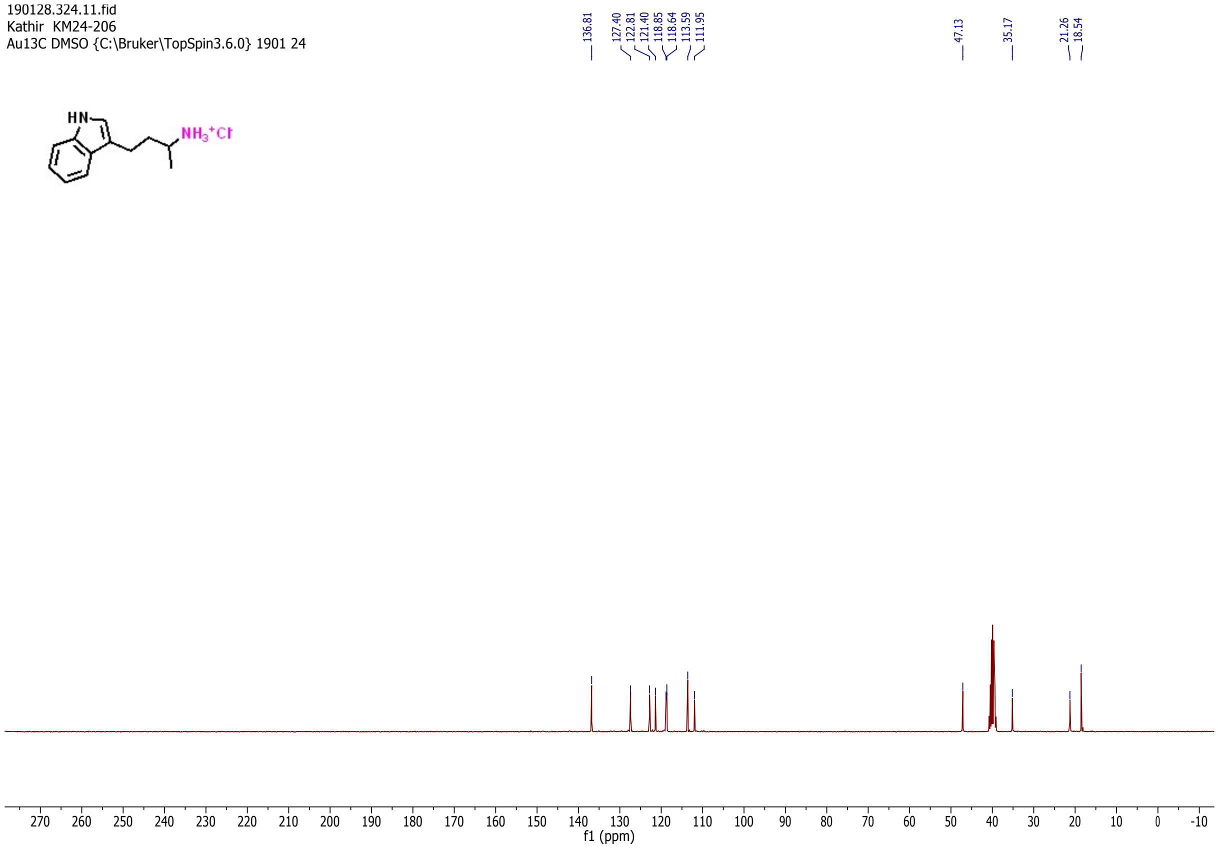
190128.324.11.fid
Kathir KM24-206
Au13C DMSO {C:\Bruker\TopSpin3.6.0} 1901 24
136.81
127.40
122.81
121.40
118.85
118.64
113.59
111.95
47.13
35.17
21.26
18.54
HN
$NH_3^+Cl^-$
270 260 250 240 230 220 210 200 190 180 170 160 150 140 130 120 110 100 90 80 70 60 50 40 30 20 10 0 -10
f1 (ppm)

190128.322.10.fid
Kathir KM24-200
Au1H DMSO {C:\Bruker\TopSpin3.6.0} 1901 22

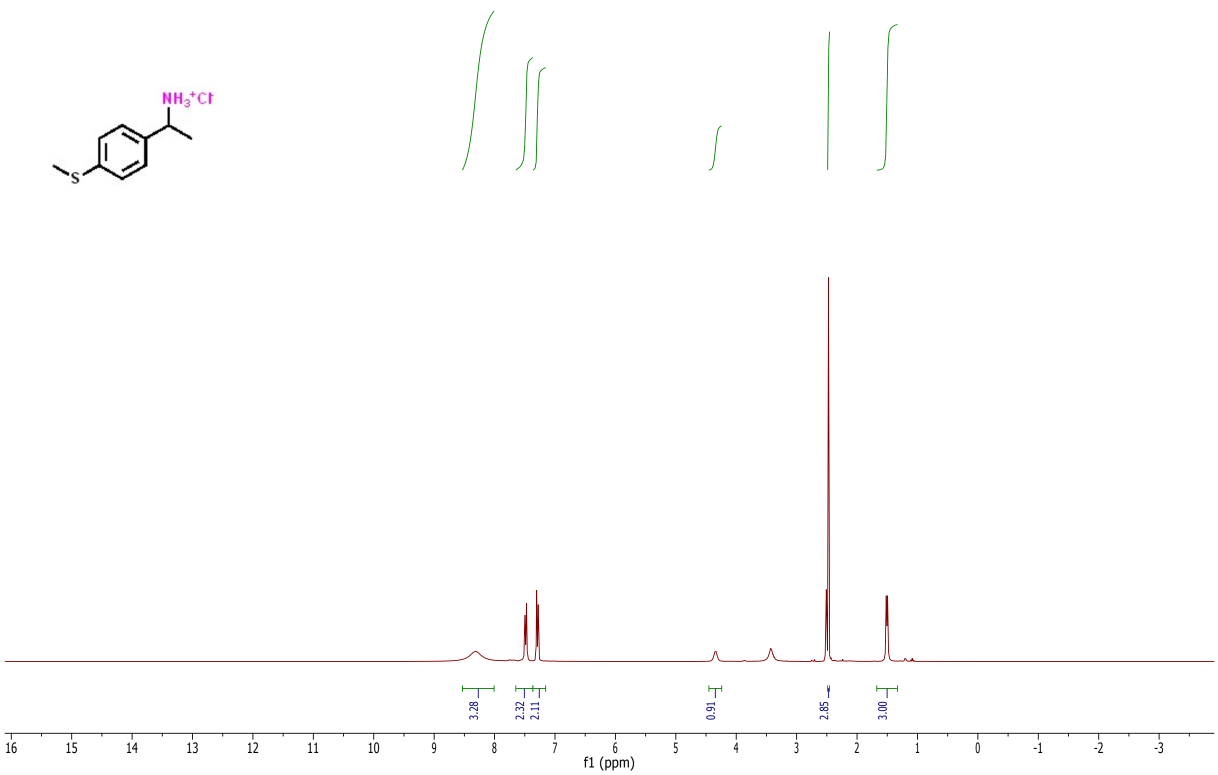

190128.322.11.fid
Kathir KM24-200
Au13C DMSO {C:\Bruker\TopSpin3.6.0} 1901 22

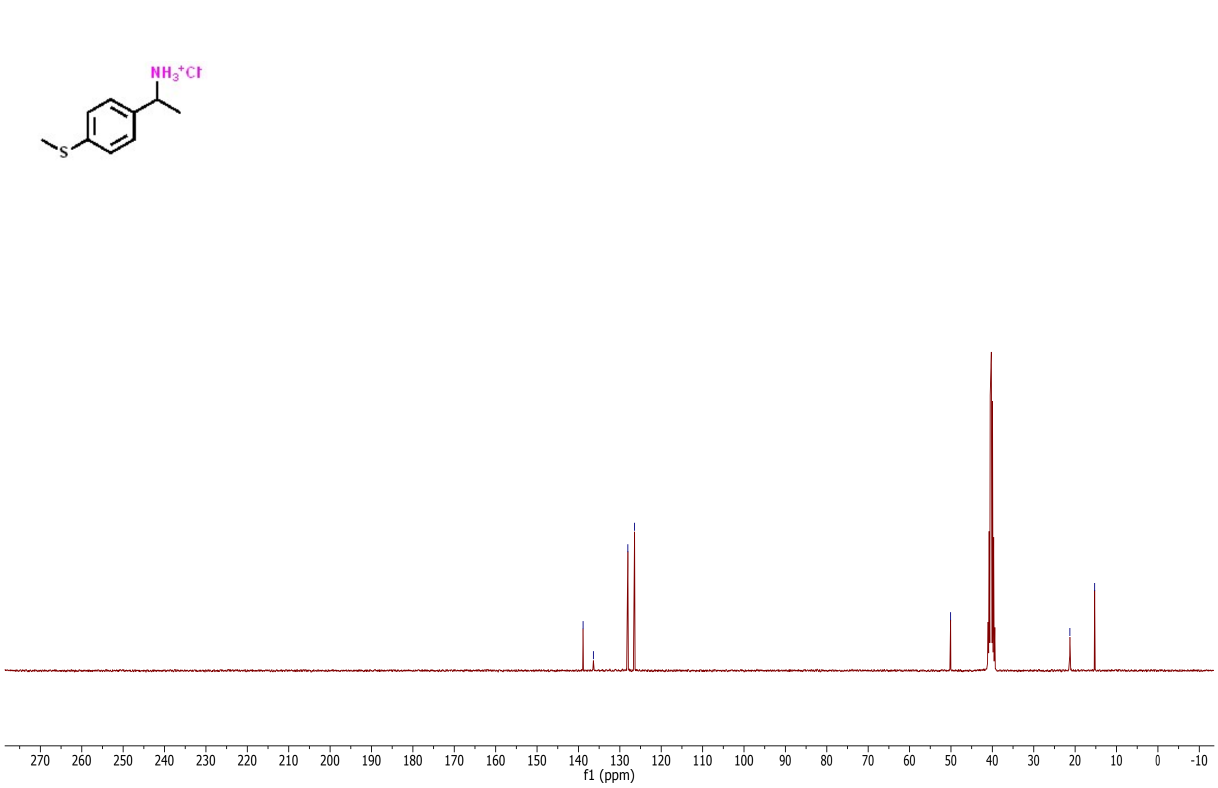

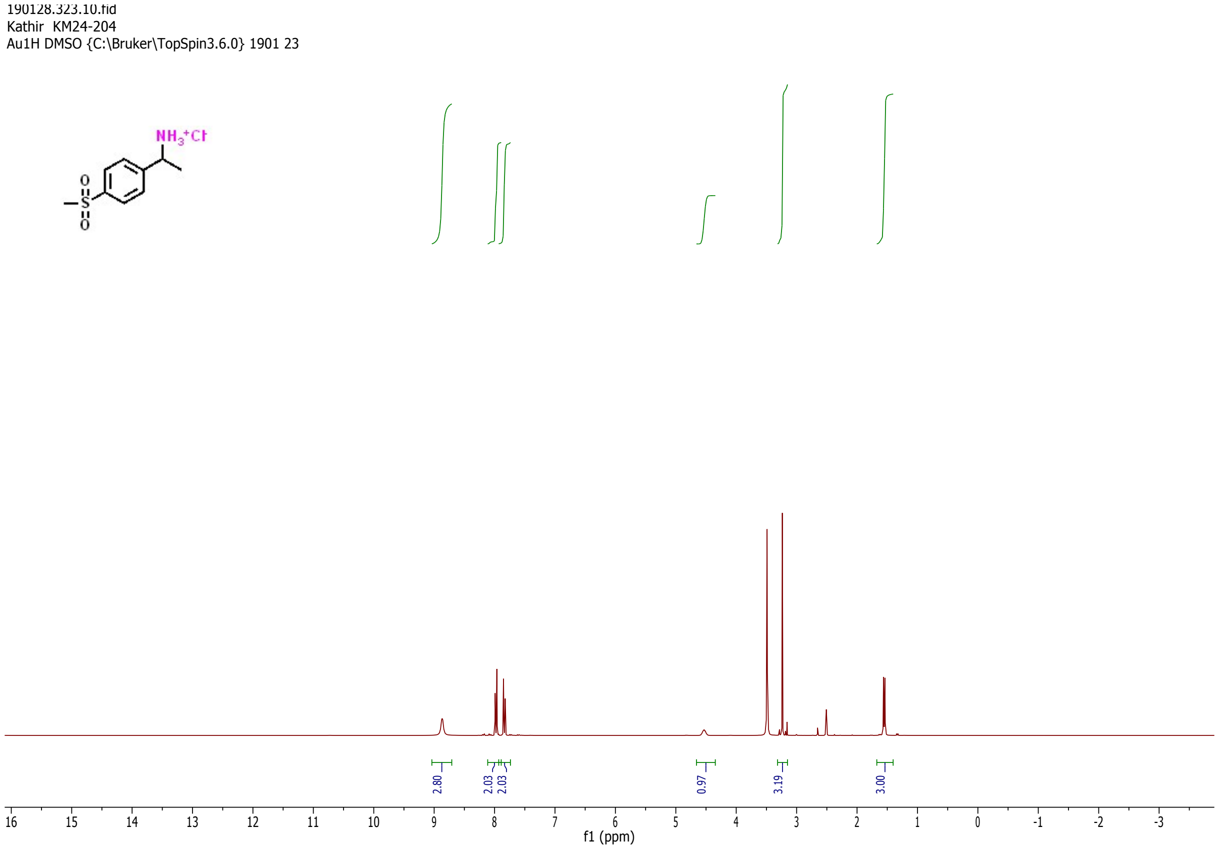
190128.323.10.fid
Kathir KM24-204
Au1H DMSO {C:\Bruker\TopSpin3.6.0} 1901 23
2.80
2.03
2.03
0.97
3.19
3.00
f1 (ppm)

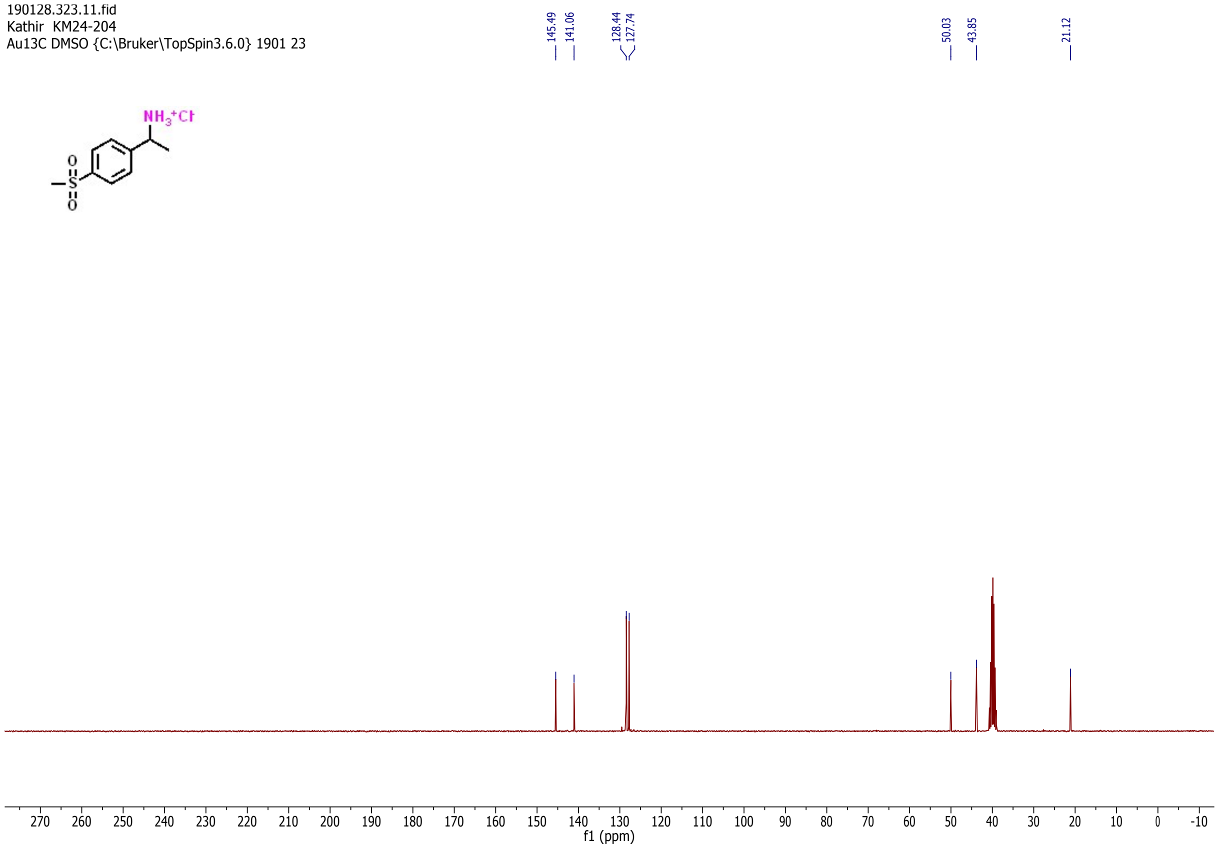
190128.323.11.fid
Kathir KM24-204
Au13C DMSO {C:\Bruker\TopSpin3.6.0} 1901 23
145.49
141.06
128.44
127.74
50.03
43.85
21.12
f1 (ppm)

190128.329.10.fid
Kathir KM24-50
Au1H DMSO {C:\Bruker\TopSpin3.6.0} 1901 29

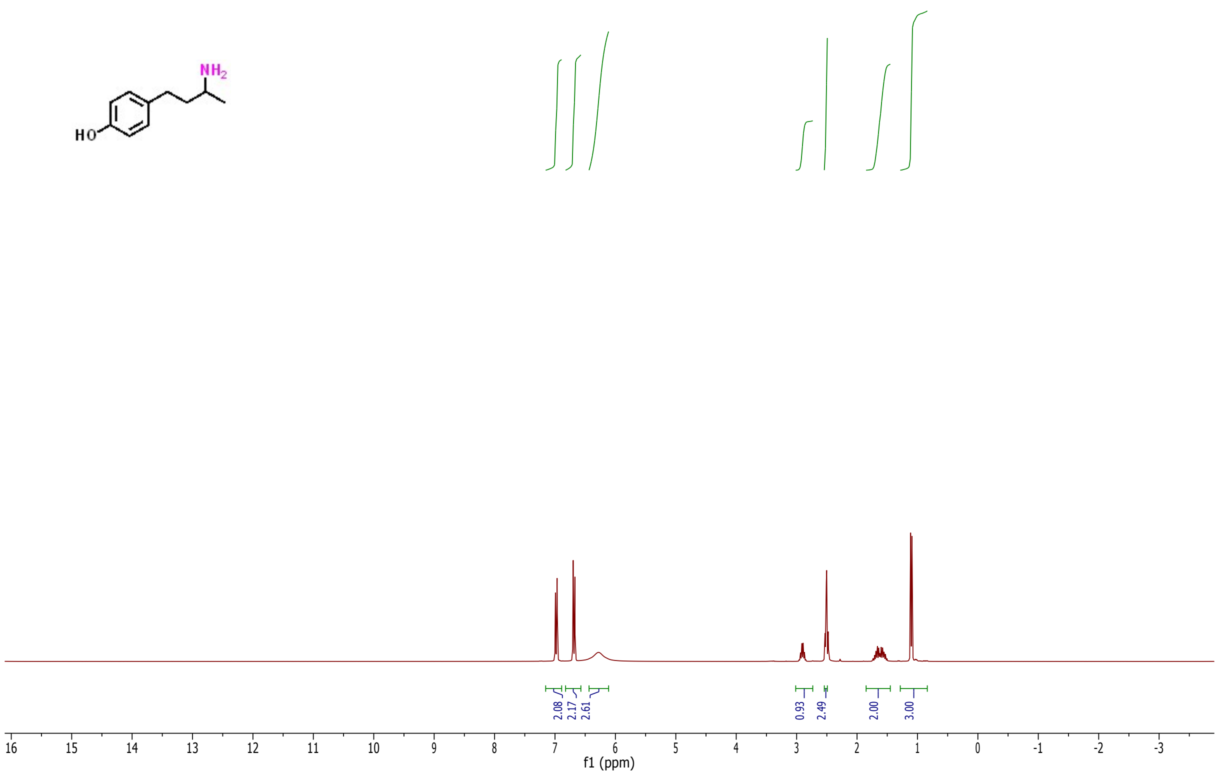

190128.329.11.fid
Kathir KM24-50
Au13C DMSO {C:\Bruker\TopSpin3.6.0} 1901 29

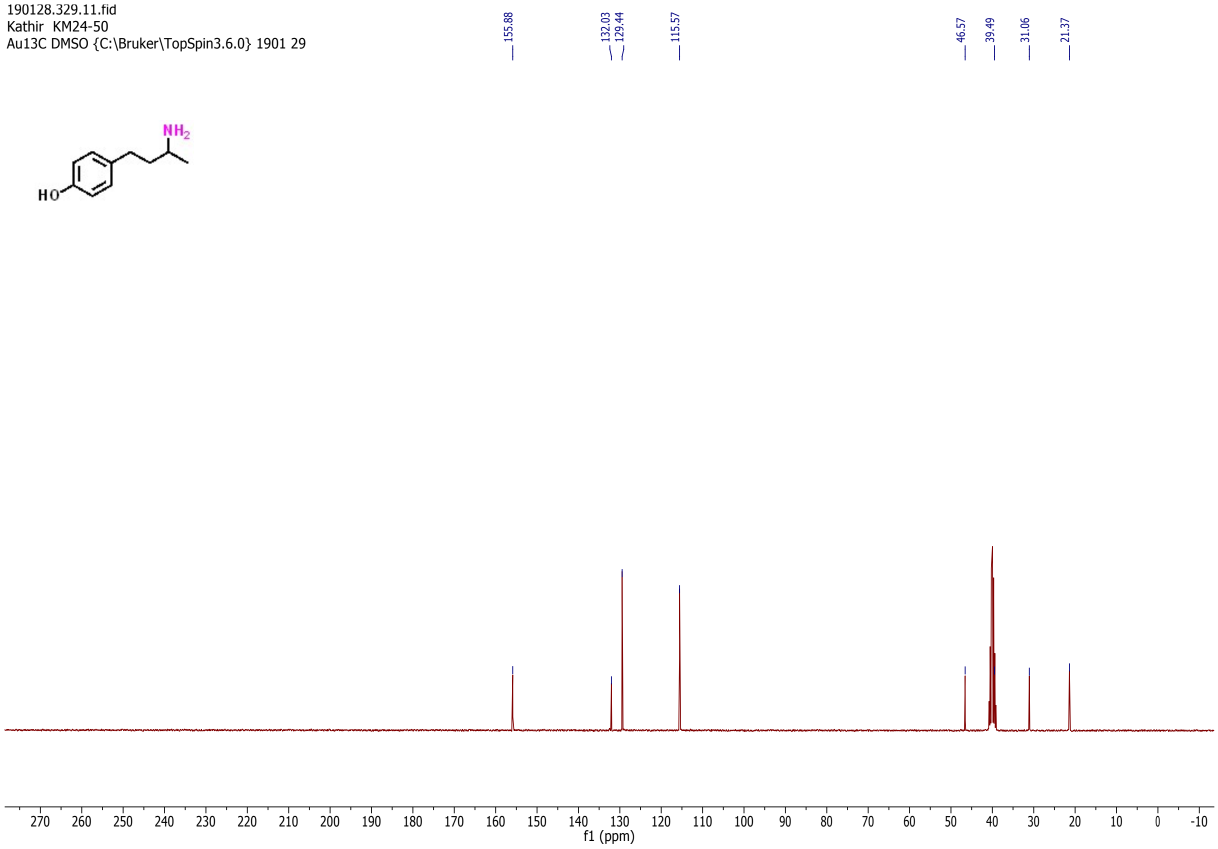

190128.326.10.fid
Kathir KM24-209
Au1H DMSO {C:\Bruker\TopSpin3.6.0} 1901 26

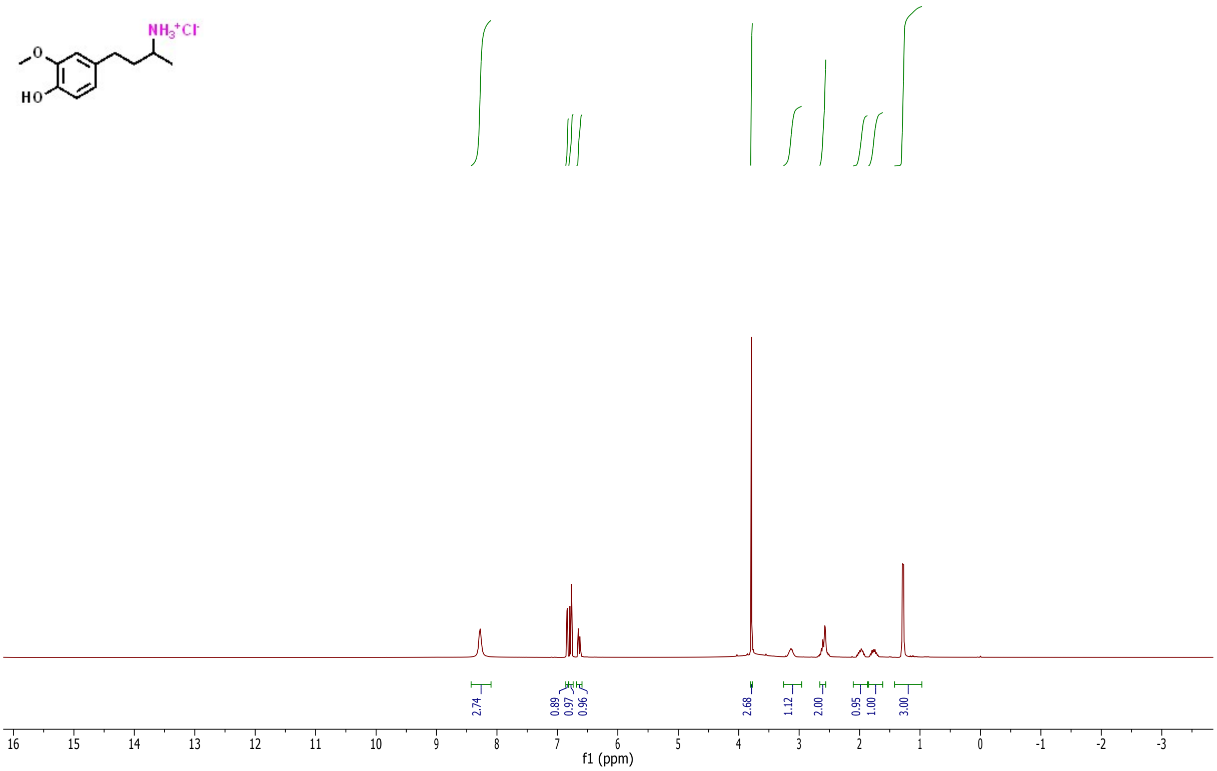

190128.326.11.fid
Kathir KM24-209
Au13C DMSO {C:\Bruker\TopSpin3.6.0} 1901 26

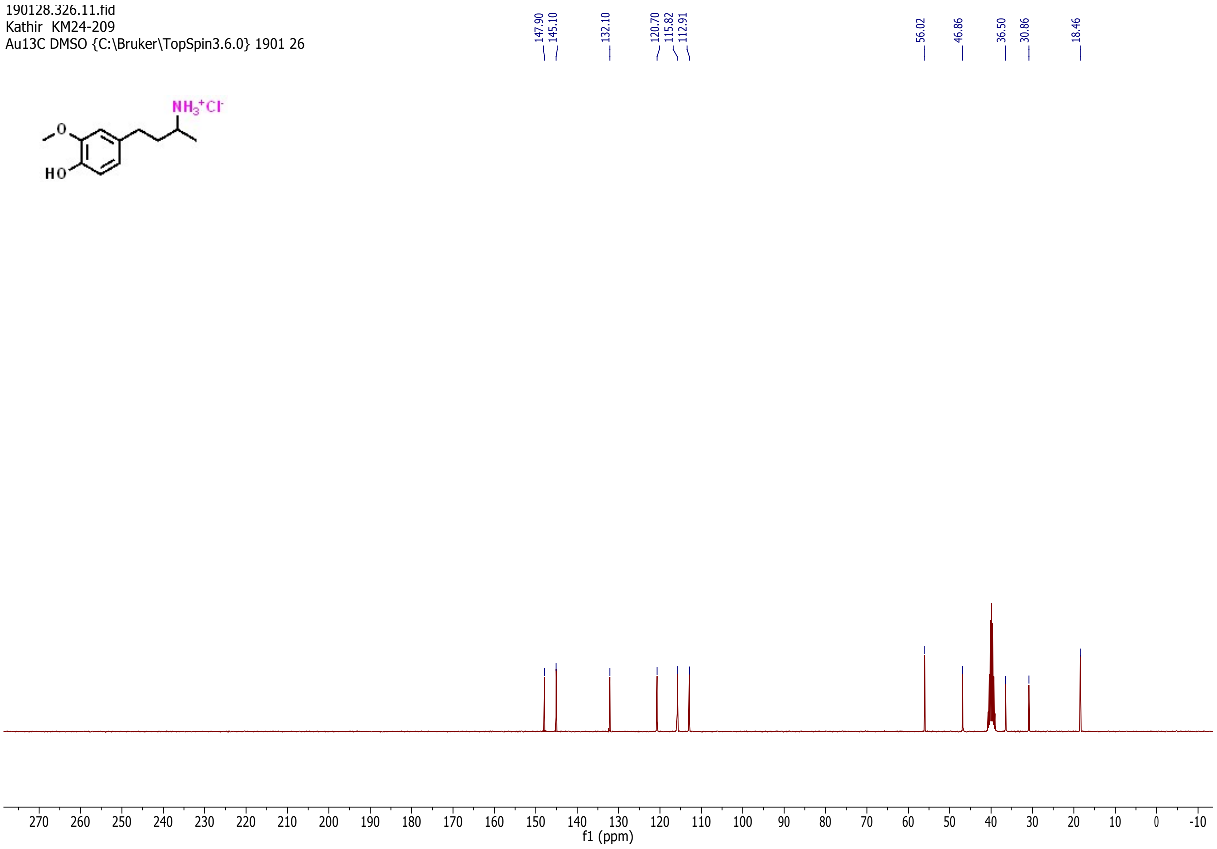

190128.318.10.fid
Kathir KM24-193
Au1H DMSO {C:\Bruker\TopSpin3.6.0} 1901 18

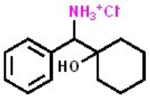

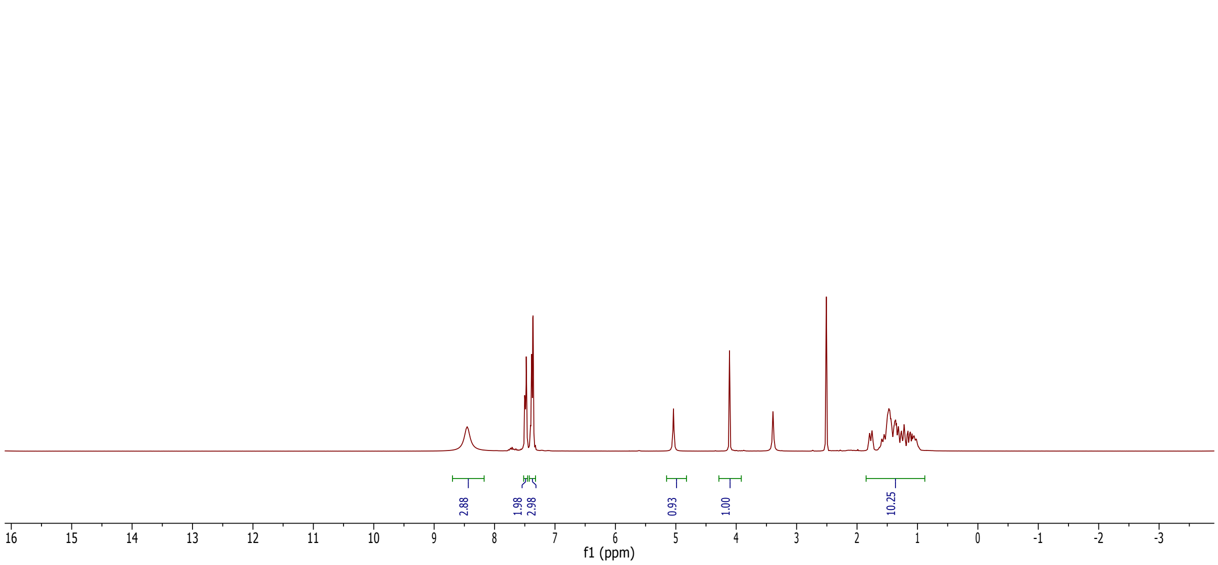

190128.318.11.fid
Kathir KM24-193
Au13C DMSO {C:\Bruker\TopSpin3.6.0} 1901 18

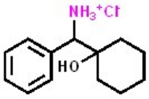

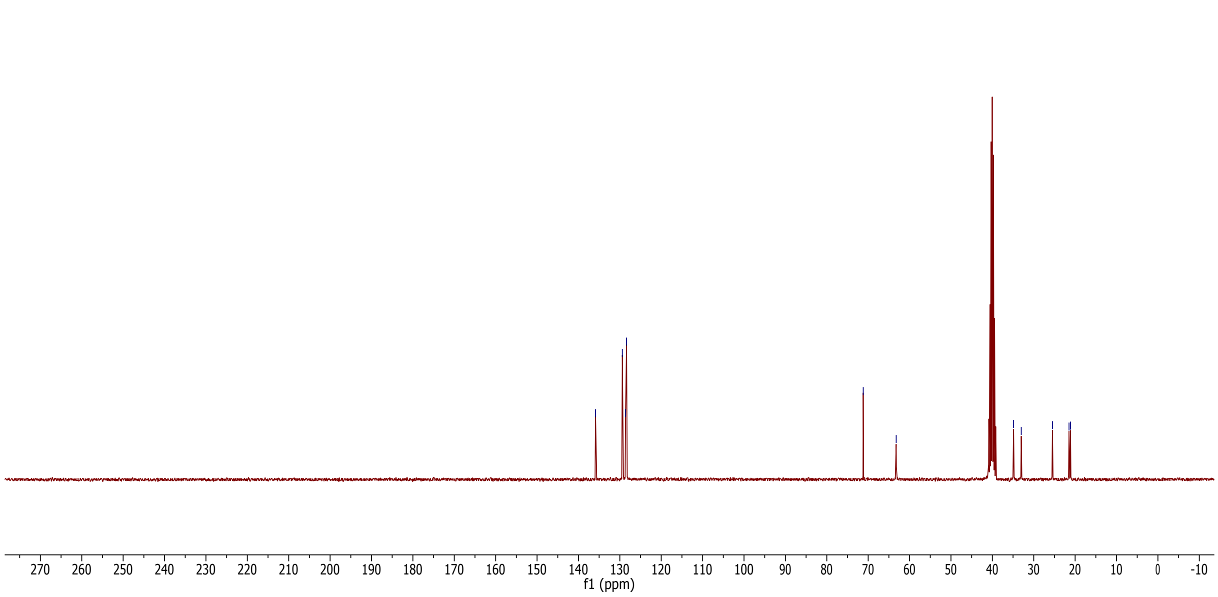

190128.320.10.fid
Kathir KM24-195
Au1H DMSO {C:\Bruker\TopSpin3.6.0} 1901 20

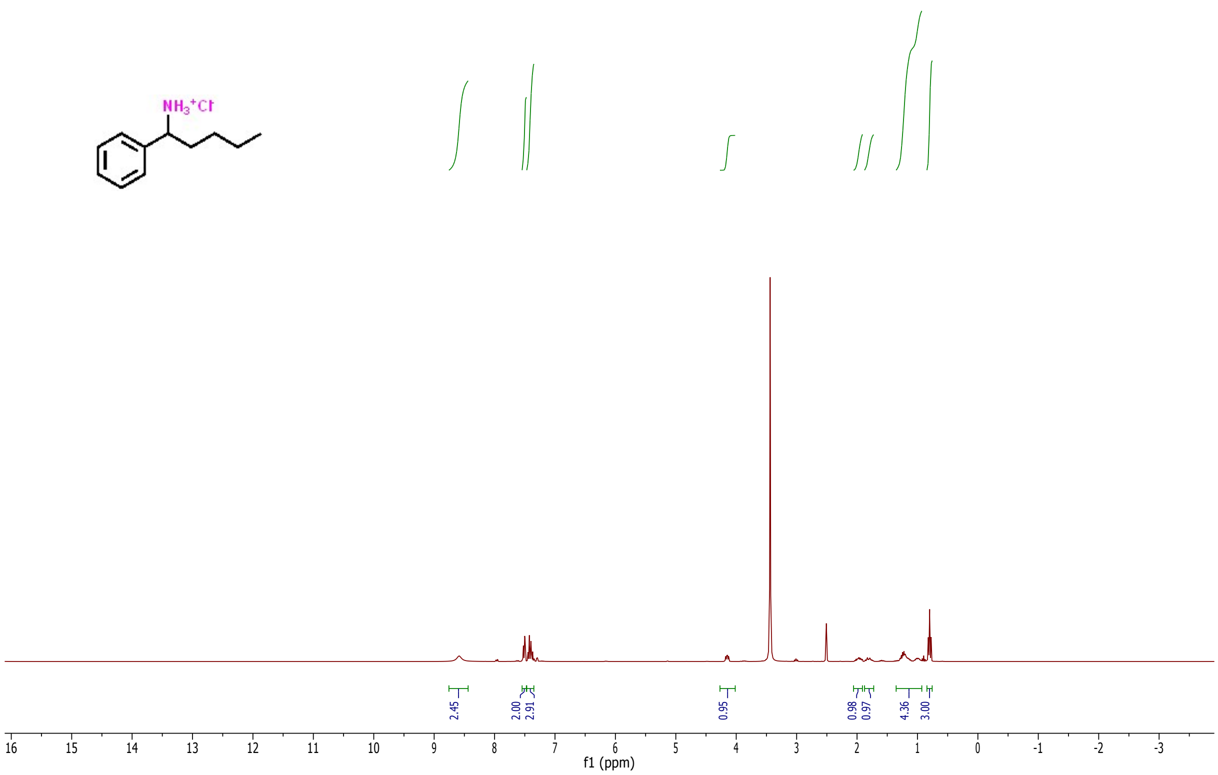

190128.320.11.fid
Kathir KM24-195
Au13C DMSO {C:\Bruker\TopSpin3.6.0} 1901 20

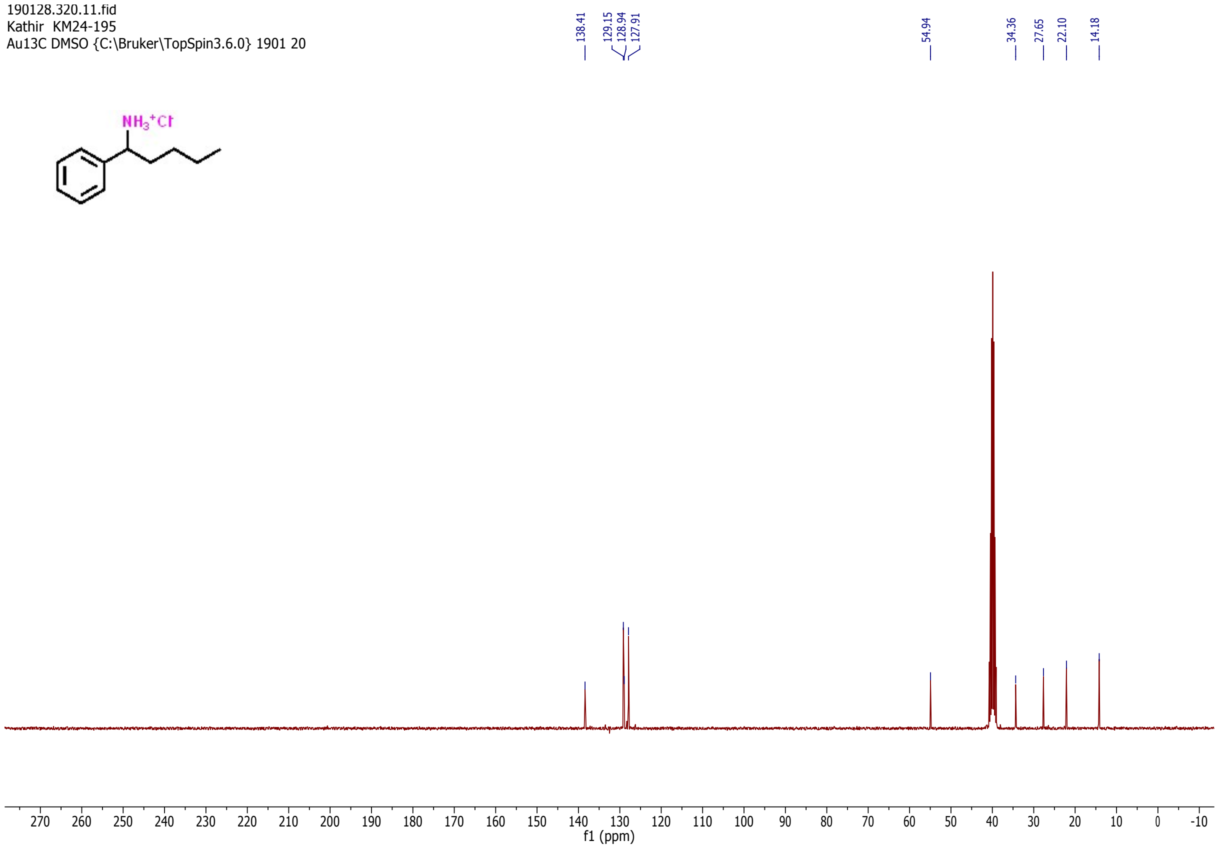

190128.325.10.fid
Kathir KM24-208
Au1H DMSO {C:\Bruker\TopSpin3.6.0} 1901 25

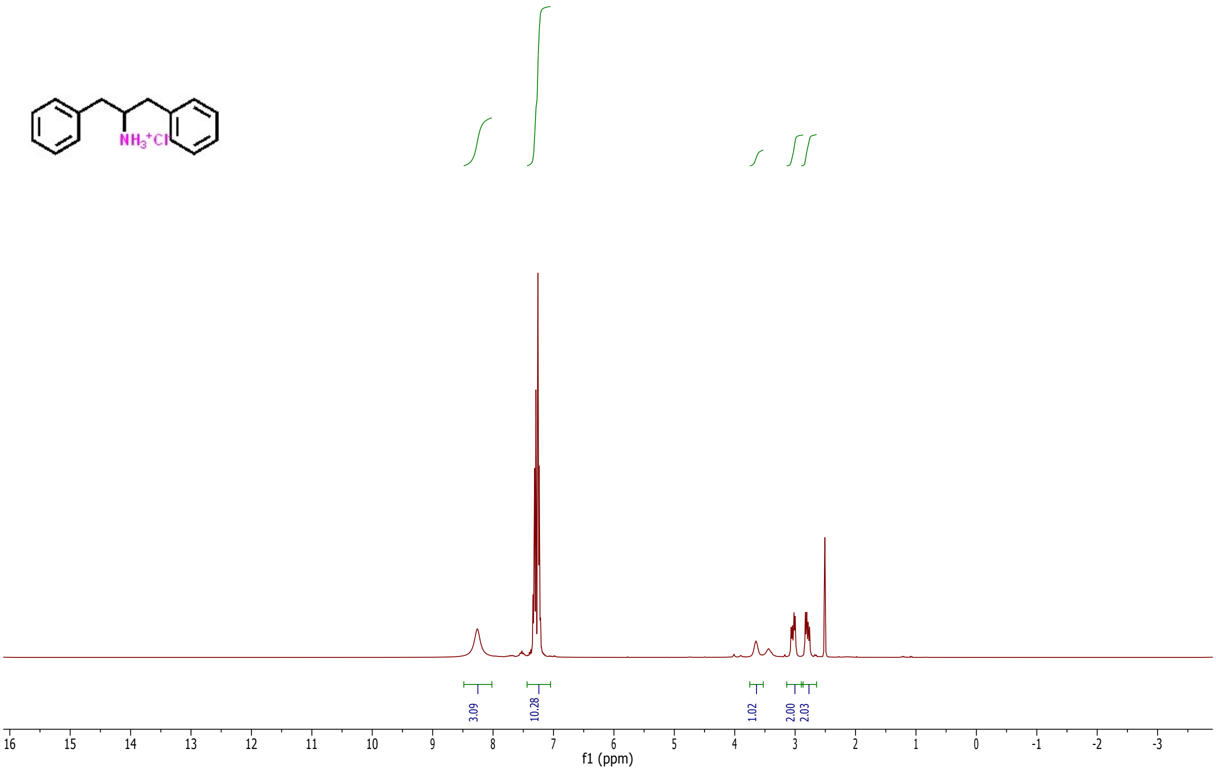

190128.325.11.fid
Kathir KM24-208
Au13C DMSO {C:\Bruker\TopSpin3.6.0} 1901 25

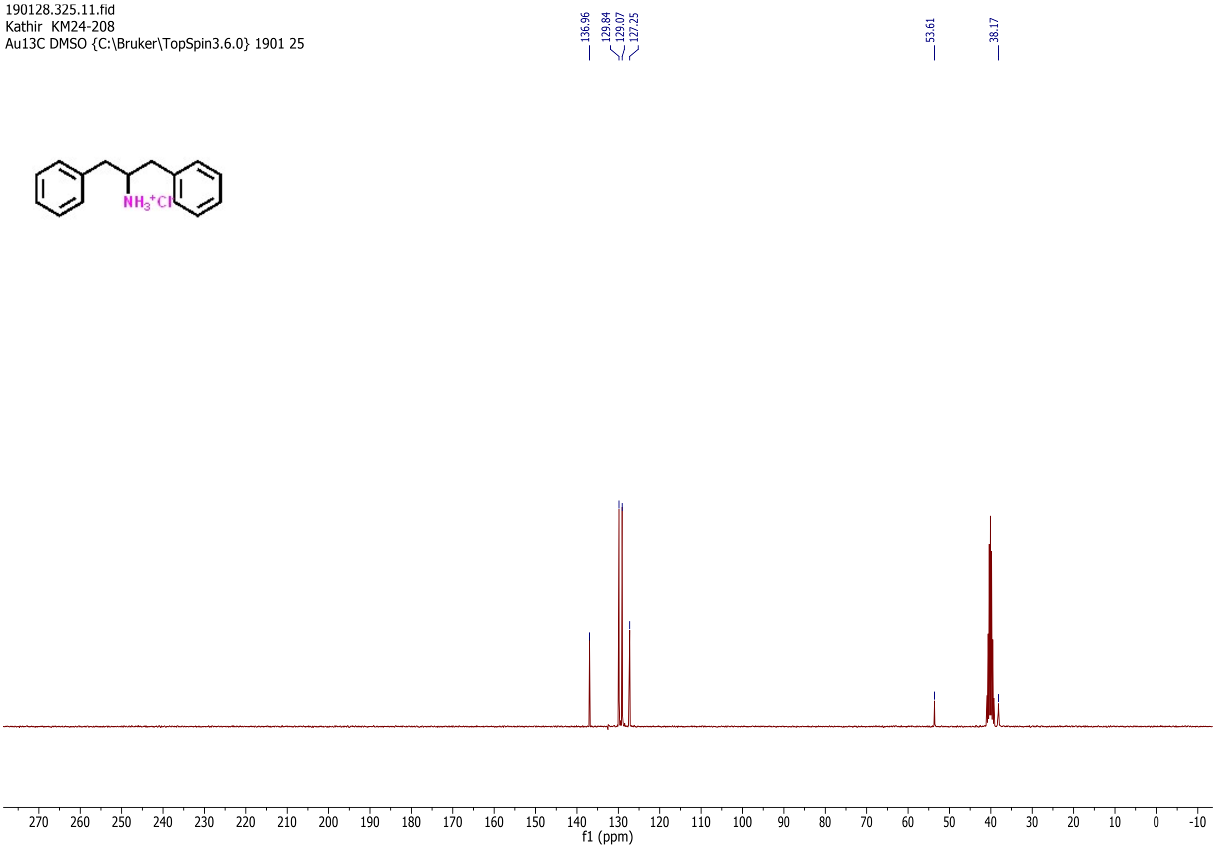

190128.327.10.fid
Kathir KM24-212
Au1H DMSO {C:\Bruker\TopSpin3.6.0} 1901 27

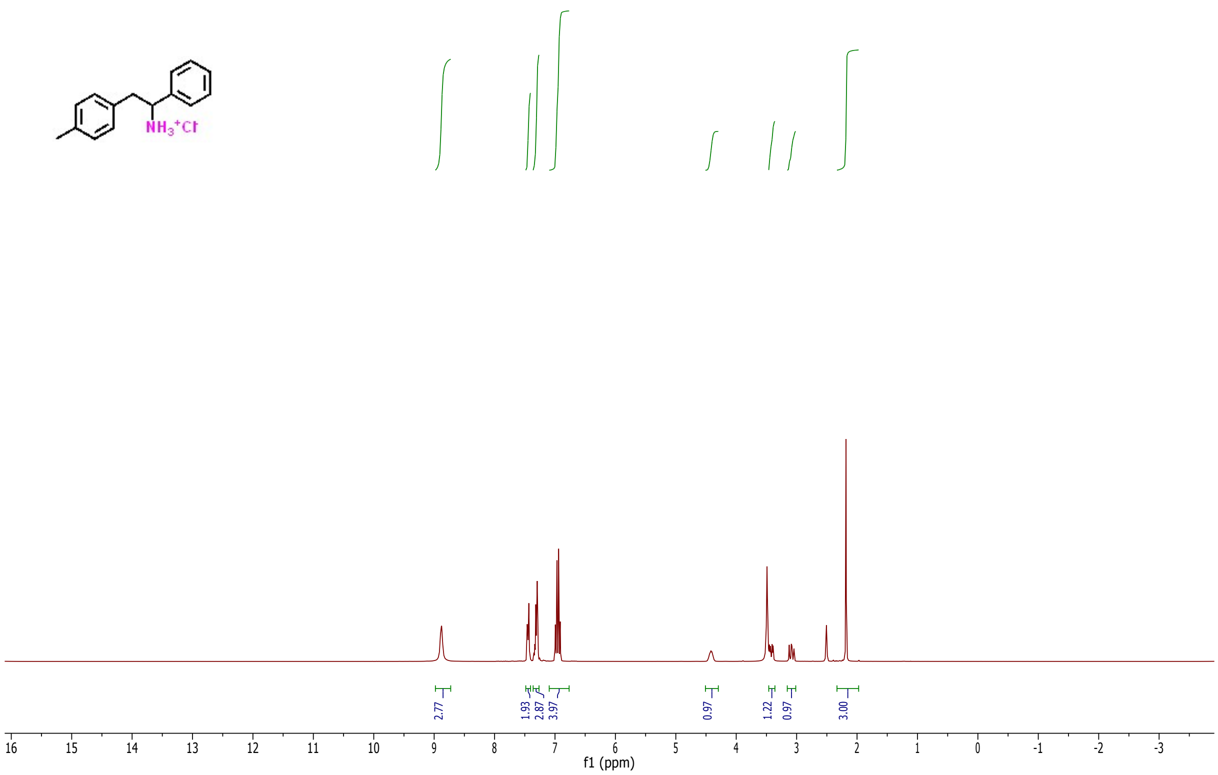

190128.327.11.fid
Kathir KM24-212
Au13C DMSO {C:\Bruker\TopSpin3.6.0} 1901 27

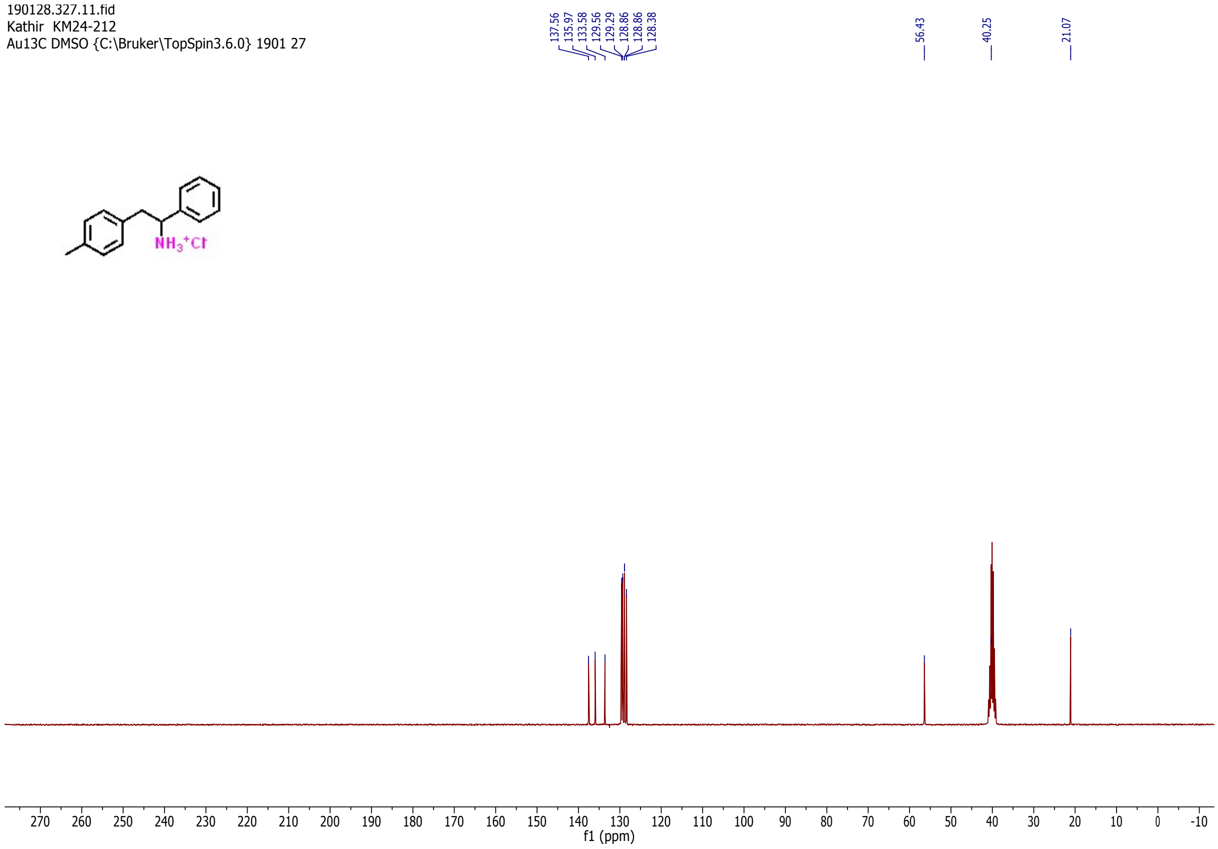

190128.328.10.fid
Kathir KM24-226
Au1H DMSO {C:\Bruker\TopSpin3.6.0} 1901 28

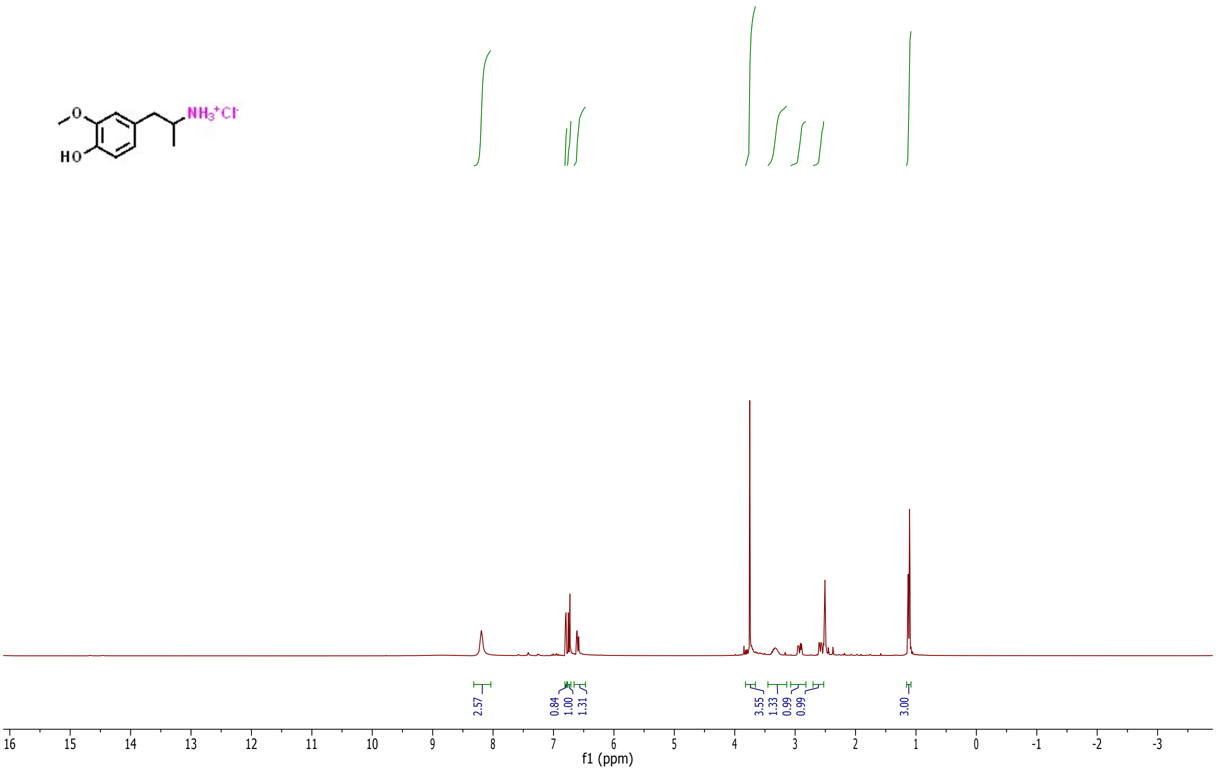

190128.328.11.fid
Kathir KM24-226
Au13C DMSO {C:\Bruker\TopSpin3.6.0} 1901 28

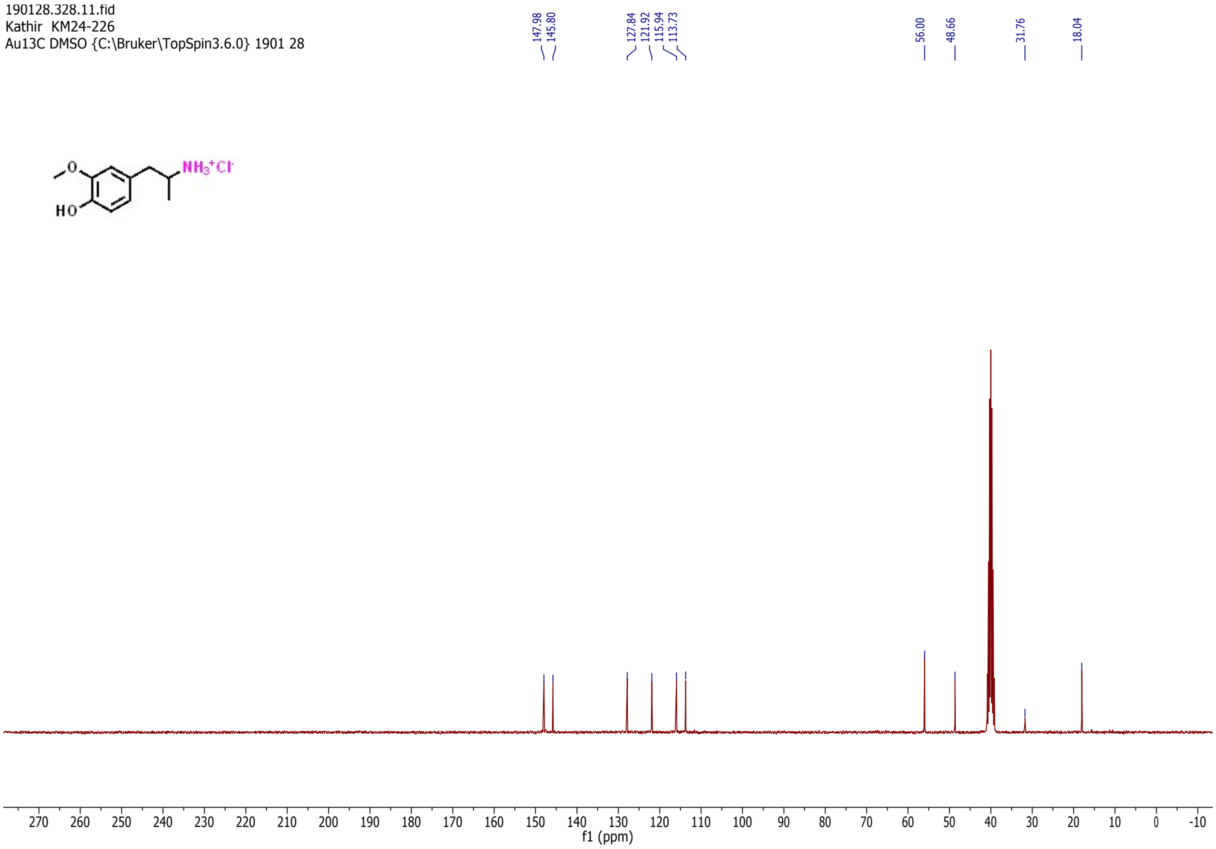

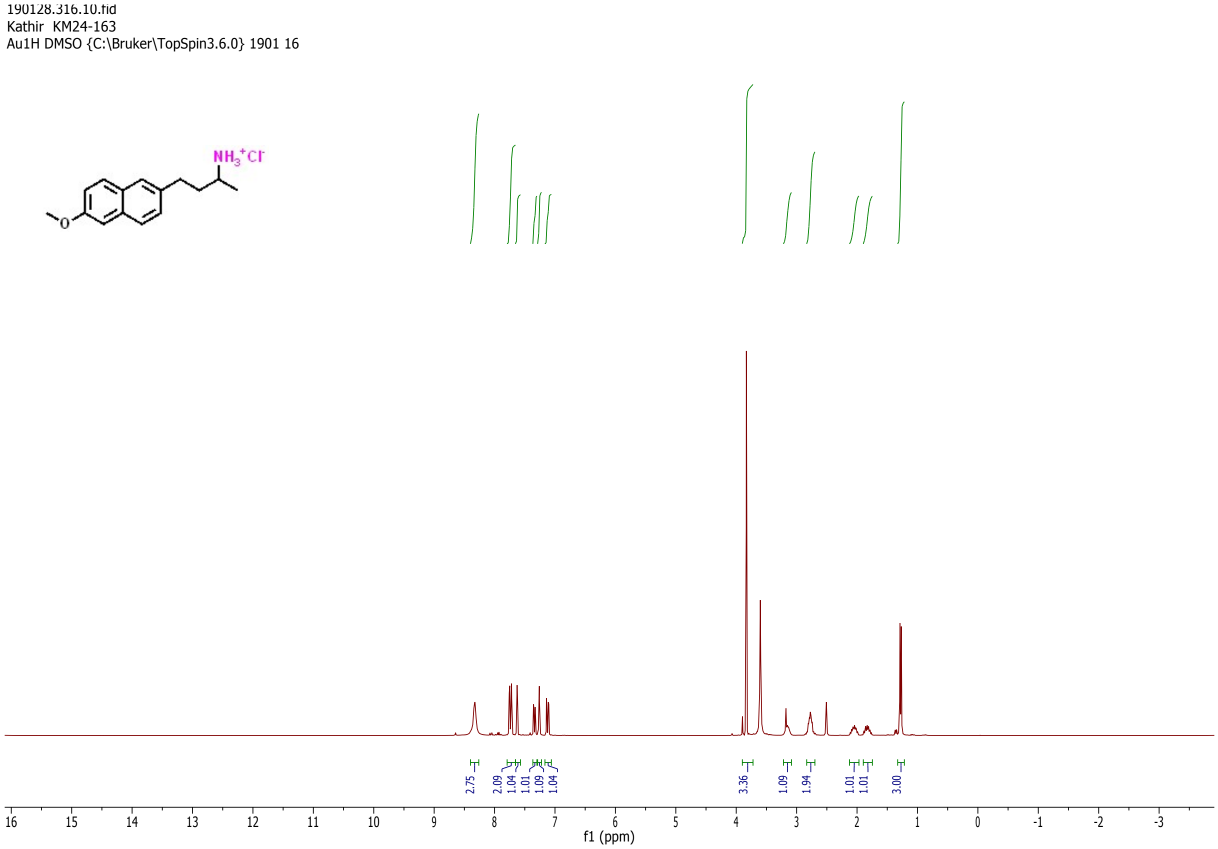
190128.316.10.fid
Kathir KM24-163
Au1H DMSO {C:\Bruker\TopSpin3.6.0} 1901 16
NH3+Cl-
O
2.75
2.09
1.04
1.01
1.09
1.04
3.36
1.09
1.94
1.01
1.01
3.00
f1 (ppm)

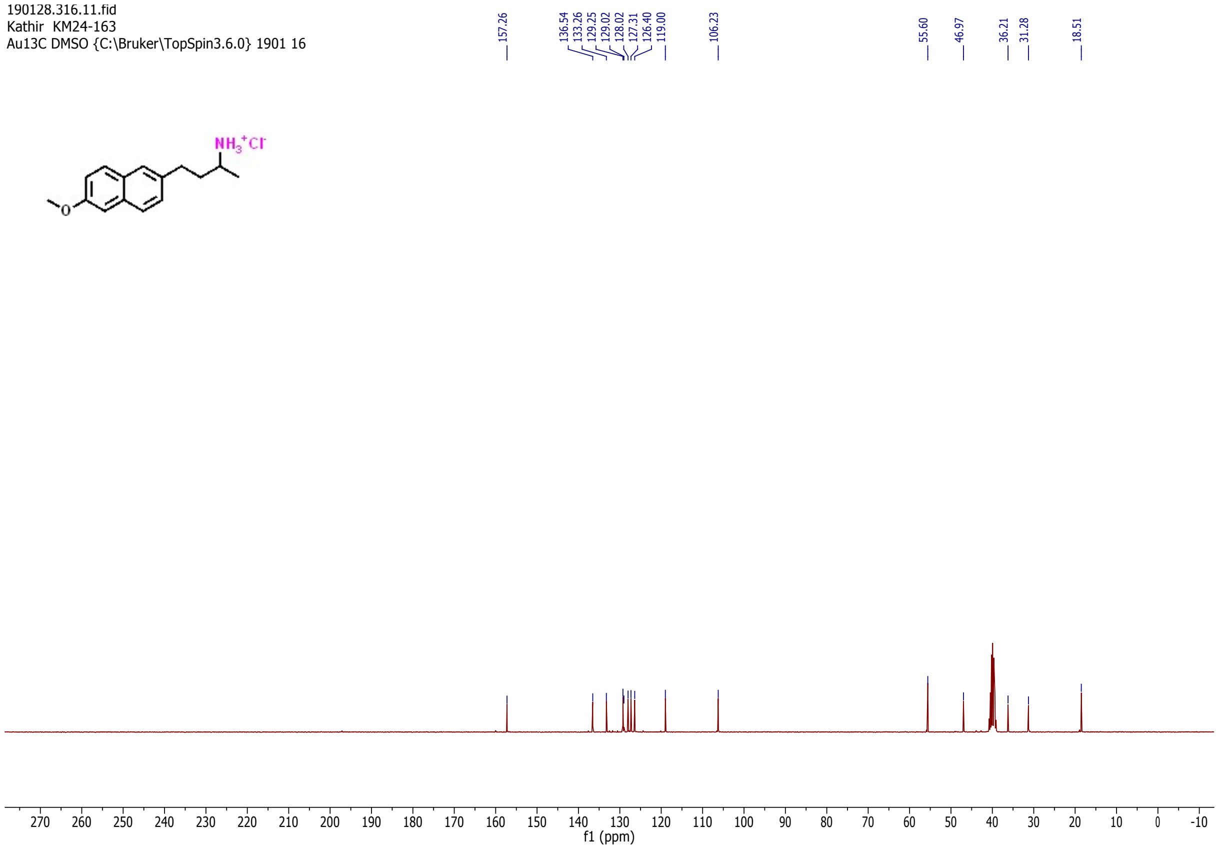
190128.316.11.fid
Kathir KM24-163
Au13C DMSO {C:\Bruker\TopSpin3.6.0} 1901 16
157.26
136.54
133.26
129.25
129.02
128.02
127.31
126.40
119.00
106.23
55.60
46.97
36.21
31.28
18.51
NH3+Cl-
O
f1 (ppm)

190128.f344.10.fid
Kathir KM24-166
PROTON MeOD {C:\Bruker\TopSpin3.6.0} 1901 44

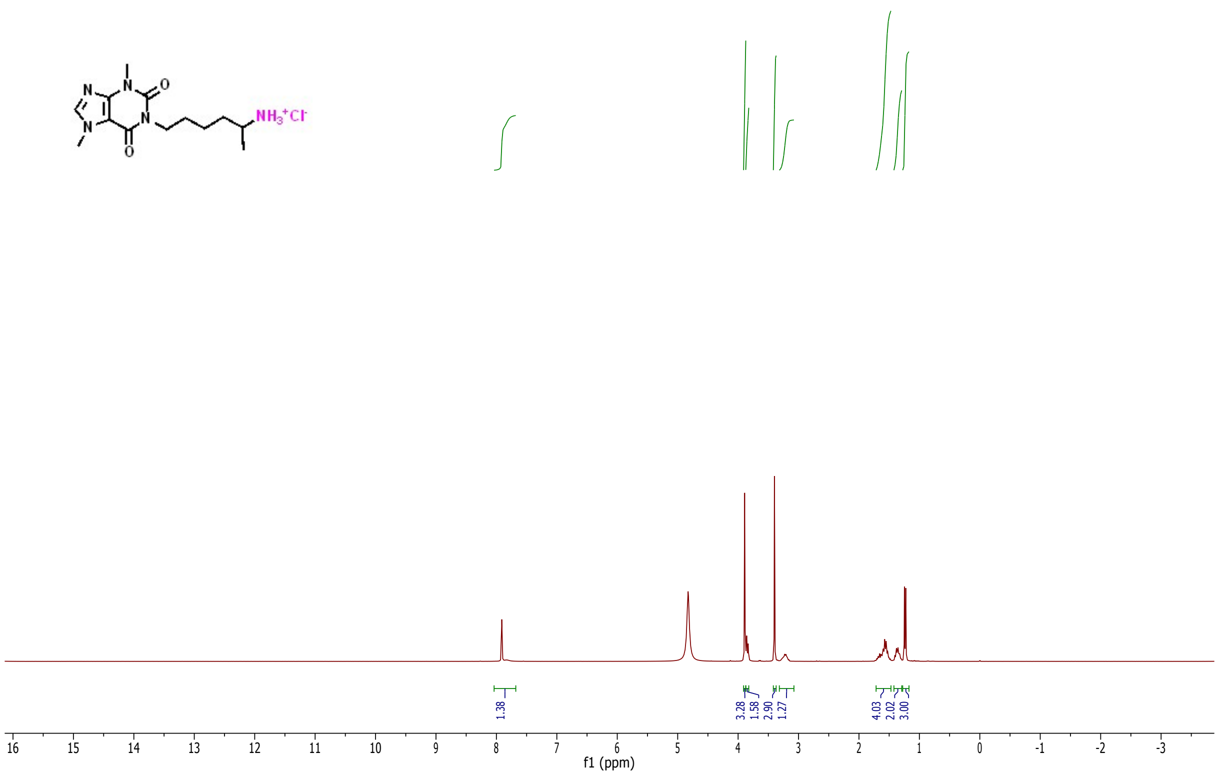

190128.f344.11.fid
Kathir KM24-166
C13CPD MeOD {C:\Bruker\TopSpin3.6.0} 1901 44

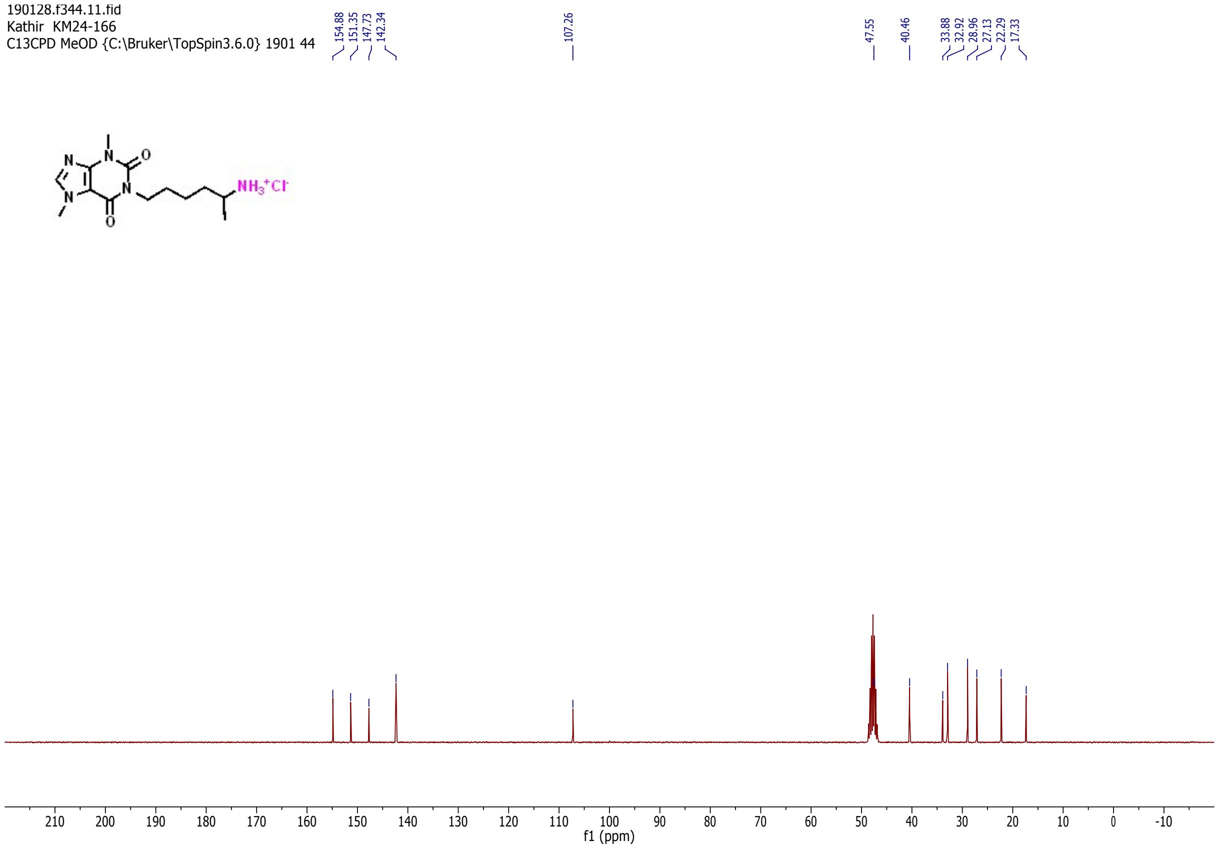

190128.f345.10.fid
Kathir KM24-165
PROTON DMSO {C:\Bruker\TopSpin3.6.0} 1901 45

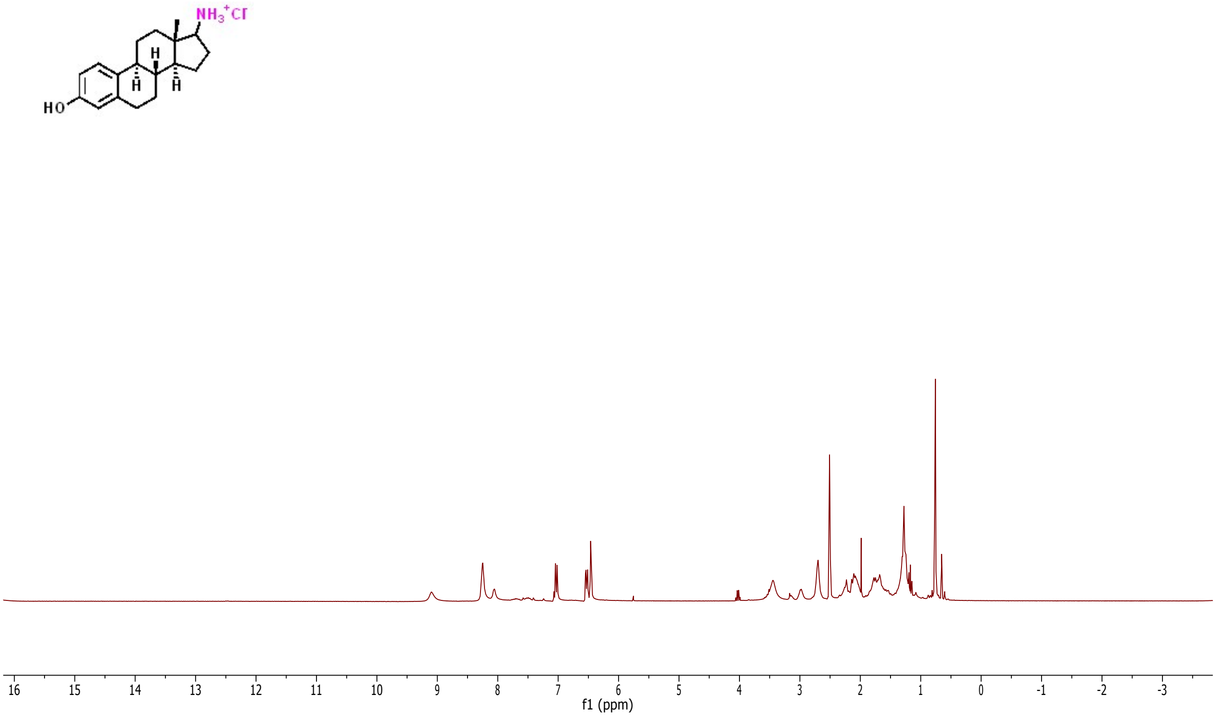

190128.f345.11.fid
Kathir KM24-165
C13CPD DMSO {C:\Bruker\TopSpin3.6.0} 1901 45

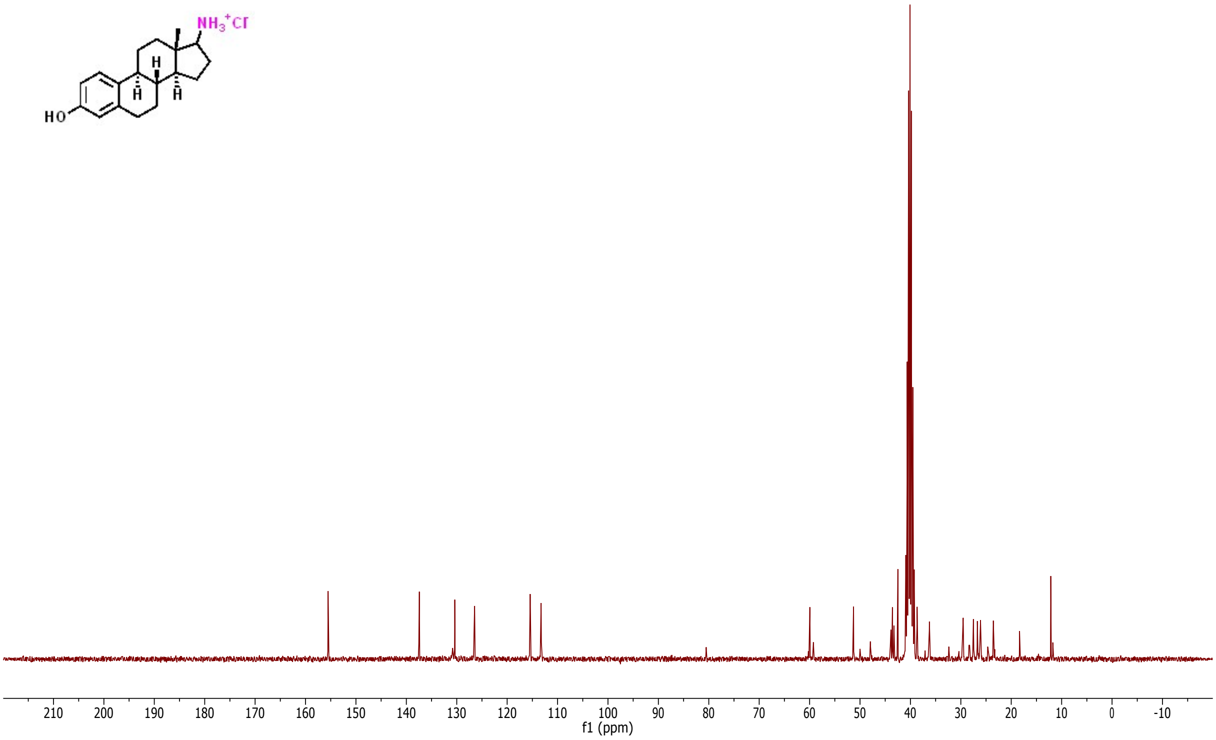

katlin? KM 24 - 165 HK(E1)

File : D:\Xcalibur\data\1903\19030502hrei-av2.RAW
Full ms [251.500 - 285.500] - Range: 271.000 - 271.500
Scan No. 1 of 1

Mass	Absolute Intensity	Relative Intensity	Theoretical Mass	Delta [ppm]	Delta [mmu]	RDB	Composition
271.19292	84857	26.1	271.19307	-0.5	-0.1	7.0	$C_{18} H_{25} O_1 N_1$

MassLib

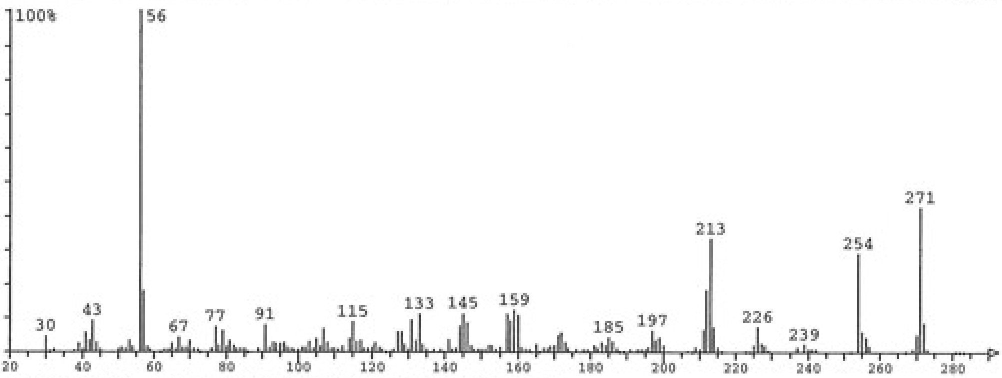

B3164(28:23) 621 16% 19030110 * MS3-Kathir//KM24-165
19030110

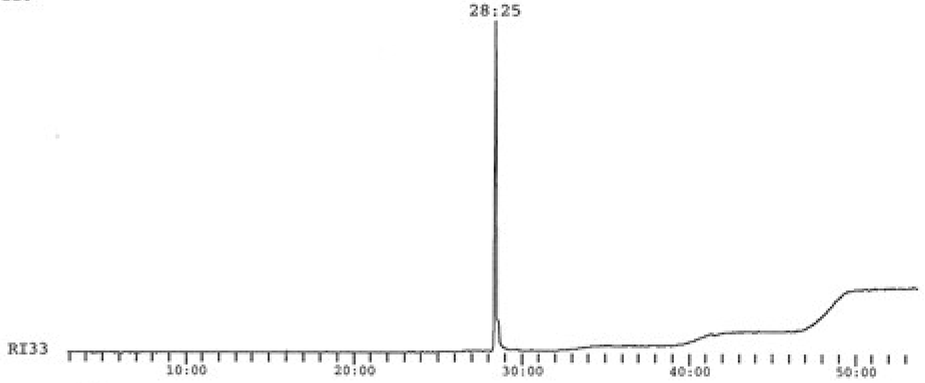

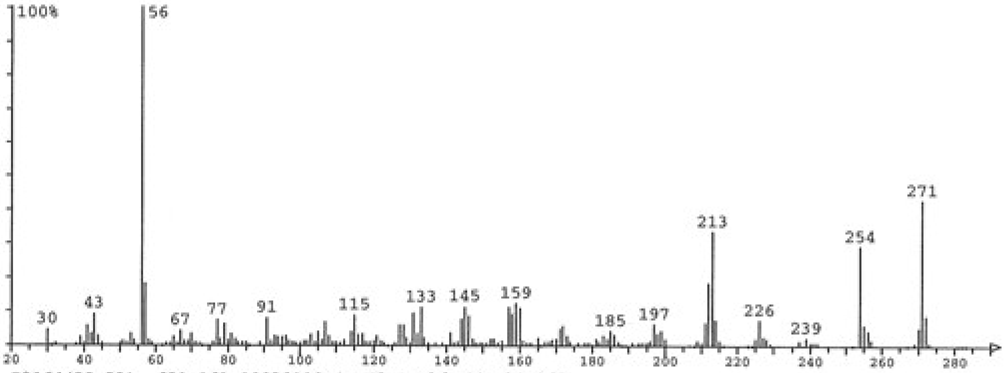

B3164(28:23) 621 16% 19030110 * MS3-Kathir//KM24-165

30	4.38	67	3.97	95	2.28	118	0.75	147	1.67	174	0.81	213	33.42
32	0.62	68	1.12	96	2.68	119	0.82	148	0.63	176	0.57	214	7.19
39	2.26	69	0.99	97	1.03	120	1.07	151	0.54	178	0.54	215	1.13
40	0.57	70	3.16	98	0.70	121	2.61	152	1.72	179	0.51	225	1.62
41	5.51	71	0.66	99	0.56	122	1.15	153	1.62	181	1.66	226	7.19
42	3.19	72	0.64	100	0.58	123	0.52	154	0.59	182	0.83	227	2.25
43	8.88	76	0.71	101	1.17	126	0.47	155	1.05	183	2.55	228	1.67
44	2.40	77	7.05	102	0.97	127	5.78	157	10.85	184	1.71	237	0.95
45	0.61	78	1.68	103	2.92	128	5.79	158	9.04	185	3.89	239	1.97
50	0.61	79	5.94	104	0.67	129	1.94	159	12.16	186	2.94	240	0.65
51	0.95	80	1.29	105	3.84	131	9.27	160	10.75	187	0.70	241	0.48
52	0.75	81	3.00	106	1.33	132	3.06	161	1.14	191	0.50	242	0.53
53	3.03	82	1.56	107	6.62	133	10.83	163	0.63	195	0.48	254	28.82
54	1.39	83	0.83	108	2.42	134	1.95	165	1.83	196	1.05	255	5.70
56	100.00	84	0.81	109	0.88	139	0.52	167	0.80	197	6.13	256	4.02
57	17.98	85	0.71	110	0.93	141	3.53	168	0.68	198	3.07	257	1.25
58	1.33	89	0.77	112	1.25	142	0.64	169	1.49	199	4.11	269	0.64
59	0.61	91	7.84	114	3.65	143	0.95	170	1.64	200	1.66	270	5.01
63	0.61	92	0.98	115	8.69	144	7.52	171	4.43	209	1.04	271	42.53
65	2.18	93	2.60	116	2.69	145	11.08	172	5.44	211	6.32	272	8.47
66	0.54	94	2.31	117	3.27	146	8.48	173	2.66	212	18.22	273	0.54

B3164(28:23)621 16% 19030110 * MS3-Kathir//KM24-165 lim: 0.44%

(Print Identifier)

190128.f343.10.fid
Kathir KM24-164
PROTON CDCl3 {C:\Bruker\TopSpin3.6.0} 1901 43

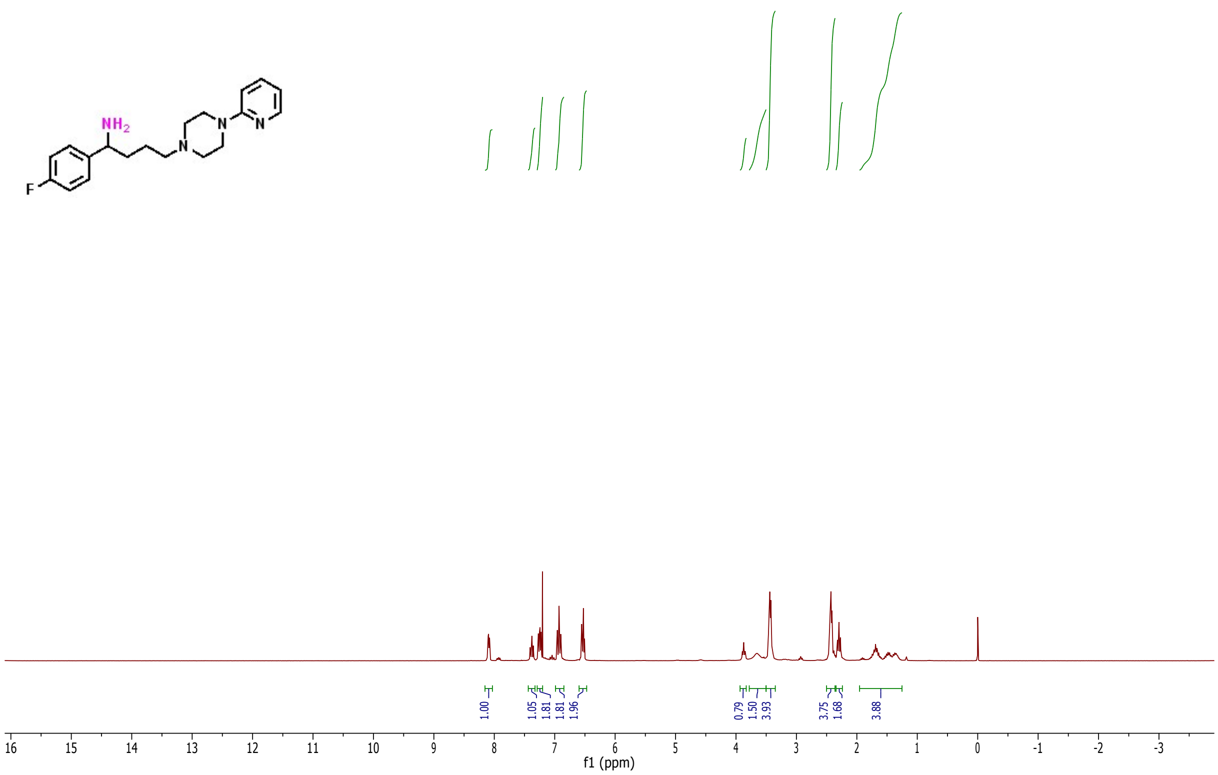

190128.f343.11.fid
Kathir KM24-164
C13CPD CDCl3 {C:\Bruker\TopSpin3.6.0} 1901 43

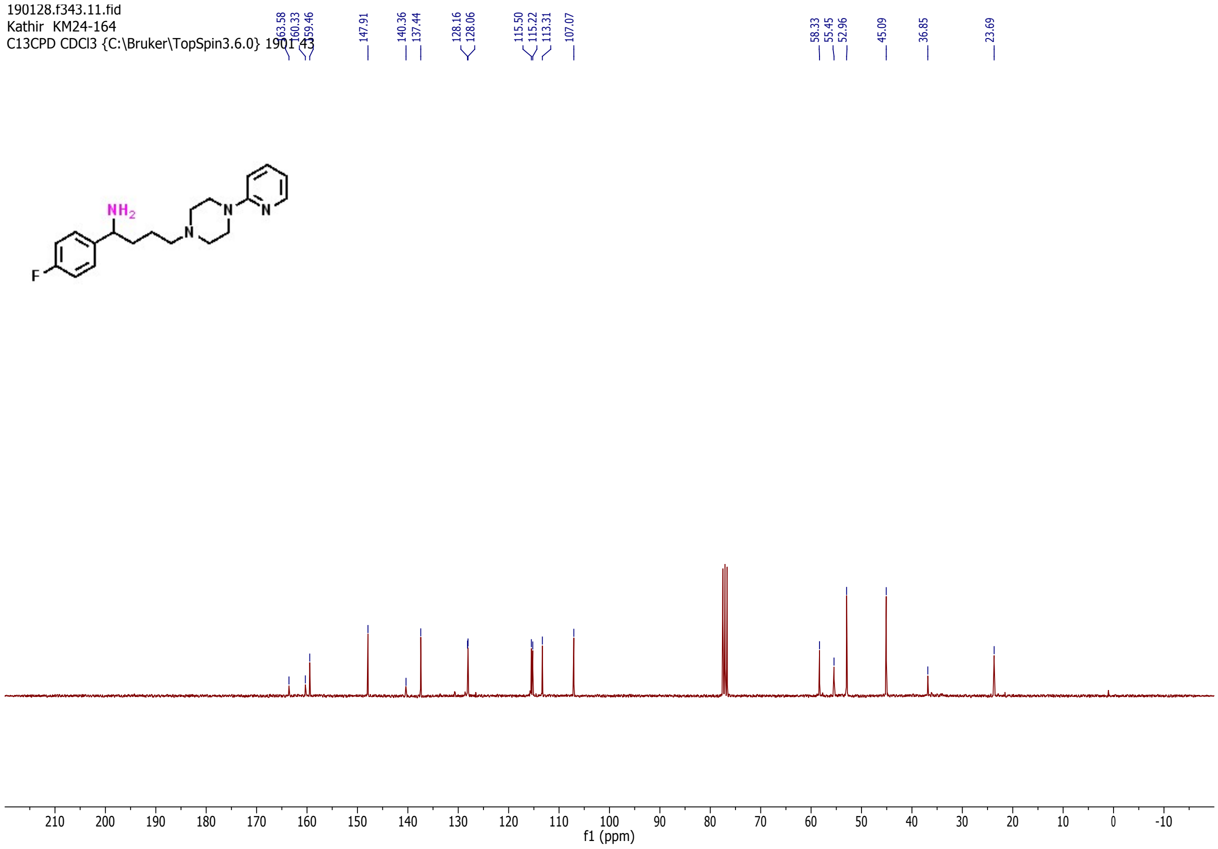

190128.319.10.fid
Kathir KM24-194
Au1H DMSO {C:\Bruker\TopSpin3.6.0} 1901 19

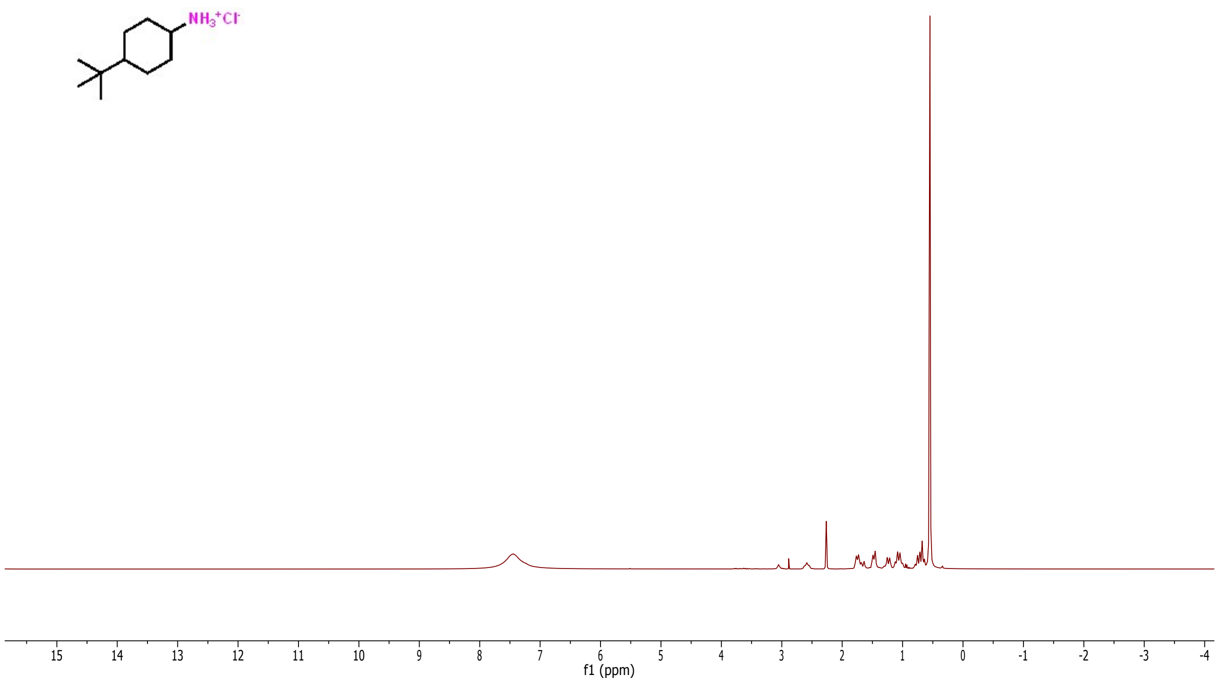

190128.319.11.fid
Kathir KM24-194
Au13C DMSO {C:\Bruker\TopSpin3.6.0} 1901 19

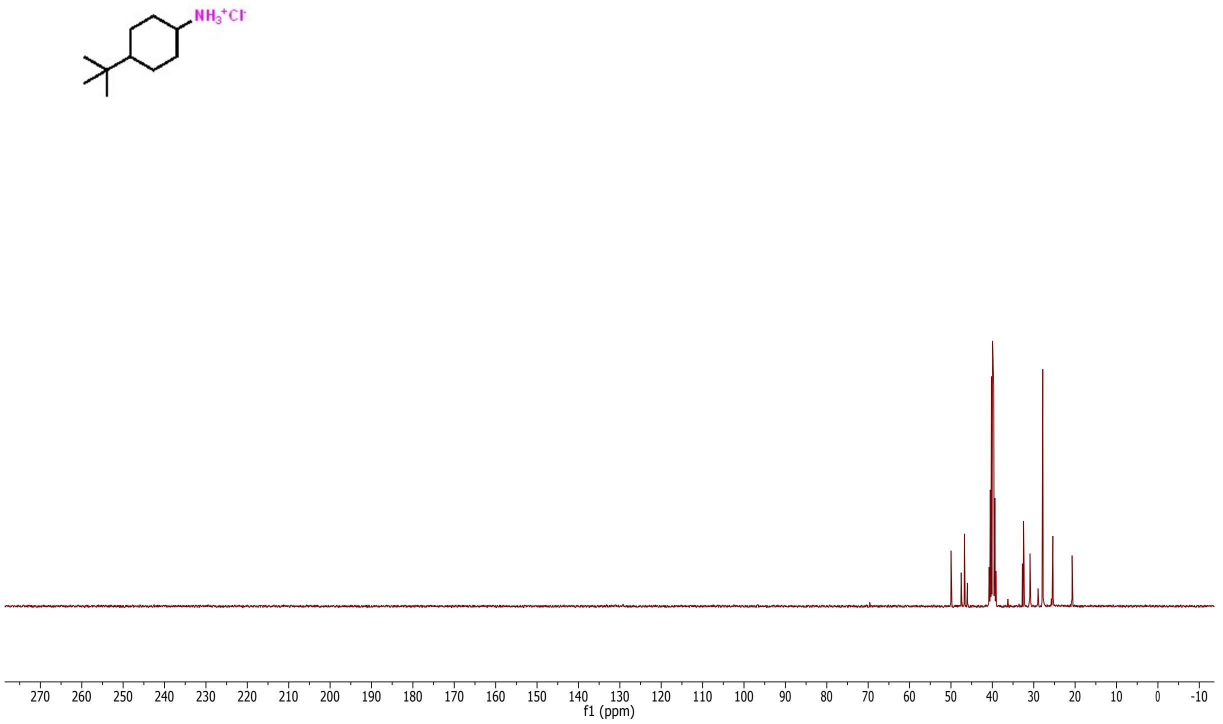

190128.317.10.fid
Kathir KM24-192
Au1H DMSO {C:\Bruker\TopSpin3.6.0} 1901 17

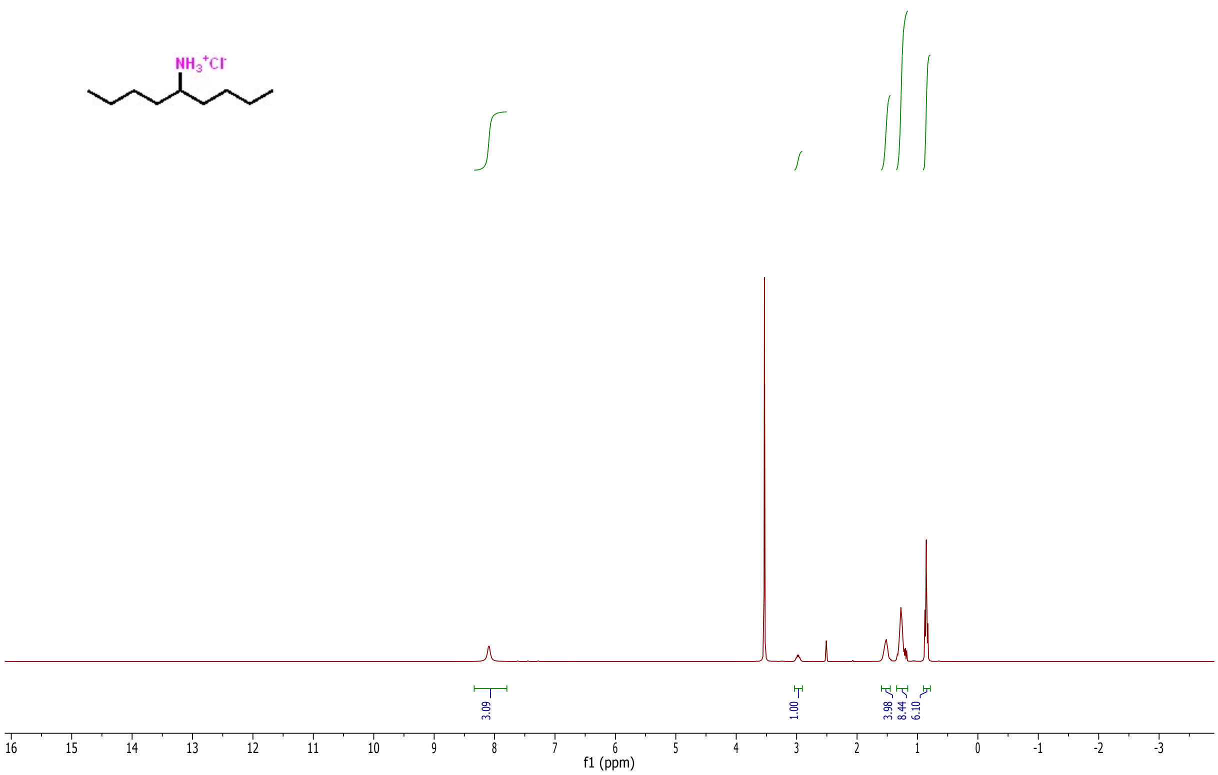

190128.317.11.fid
Kathir KM24-192
Au13C DMSO {C:\Bruker\TopSpin3.6.0} 1901 17

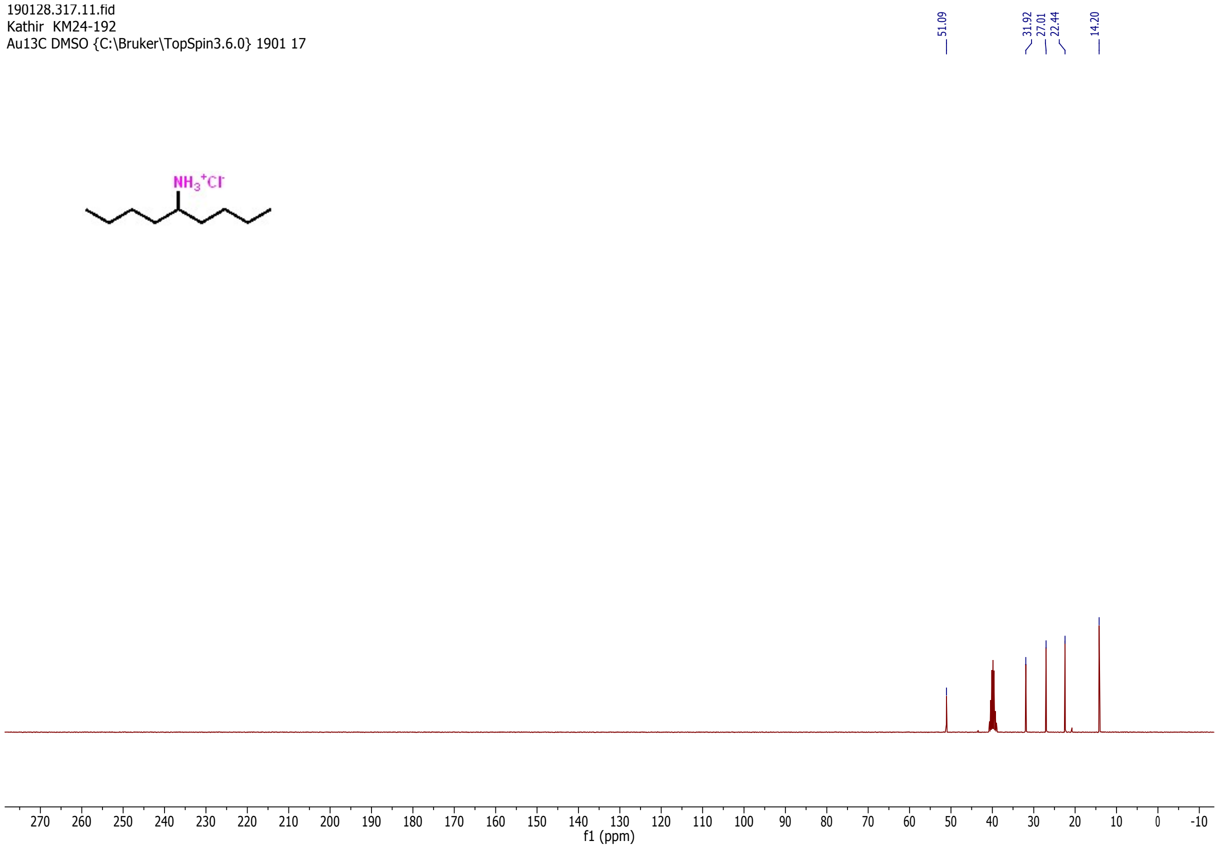

190128.315.10.fid
Kathir KM24-157
Au1H DMSO {C:\Bruker\TopSpin3.6.0} 1901 15

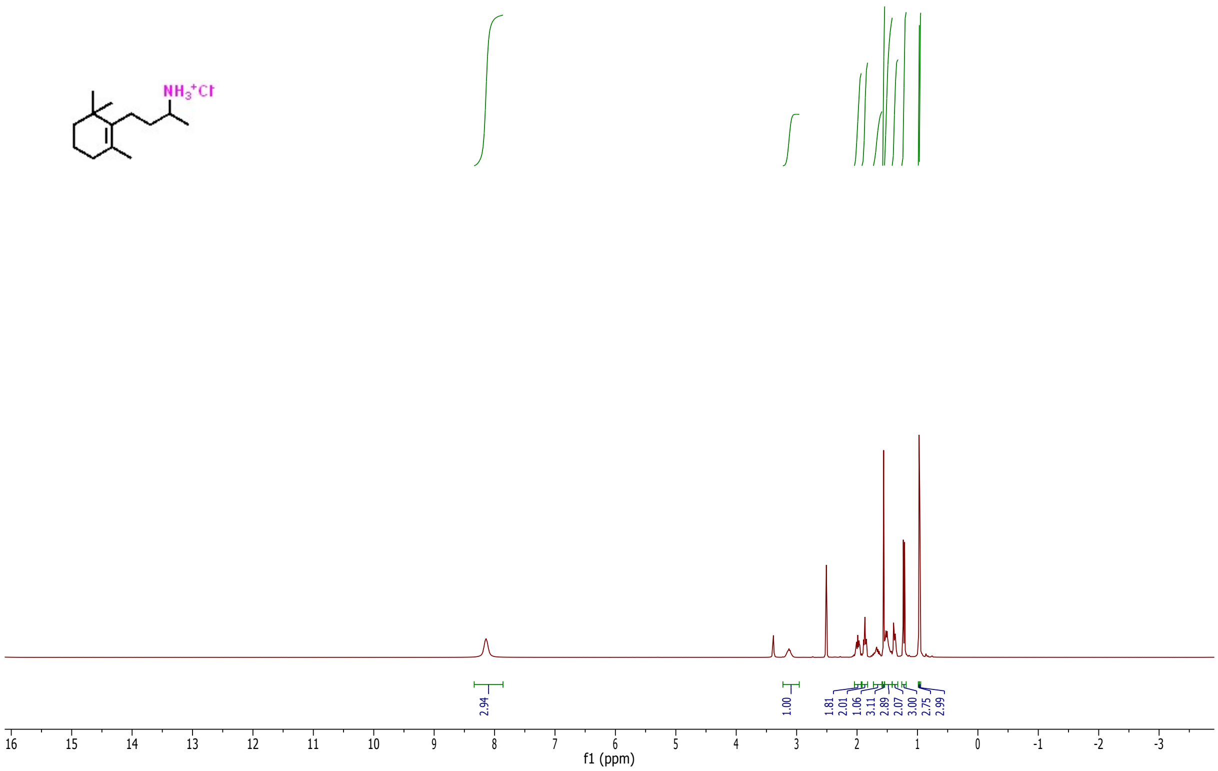

190128.315.11.fid
Kathir KM24-157
Au13C DMSO {C:\Bruker\TopSpin3.6.0} 1901 15

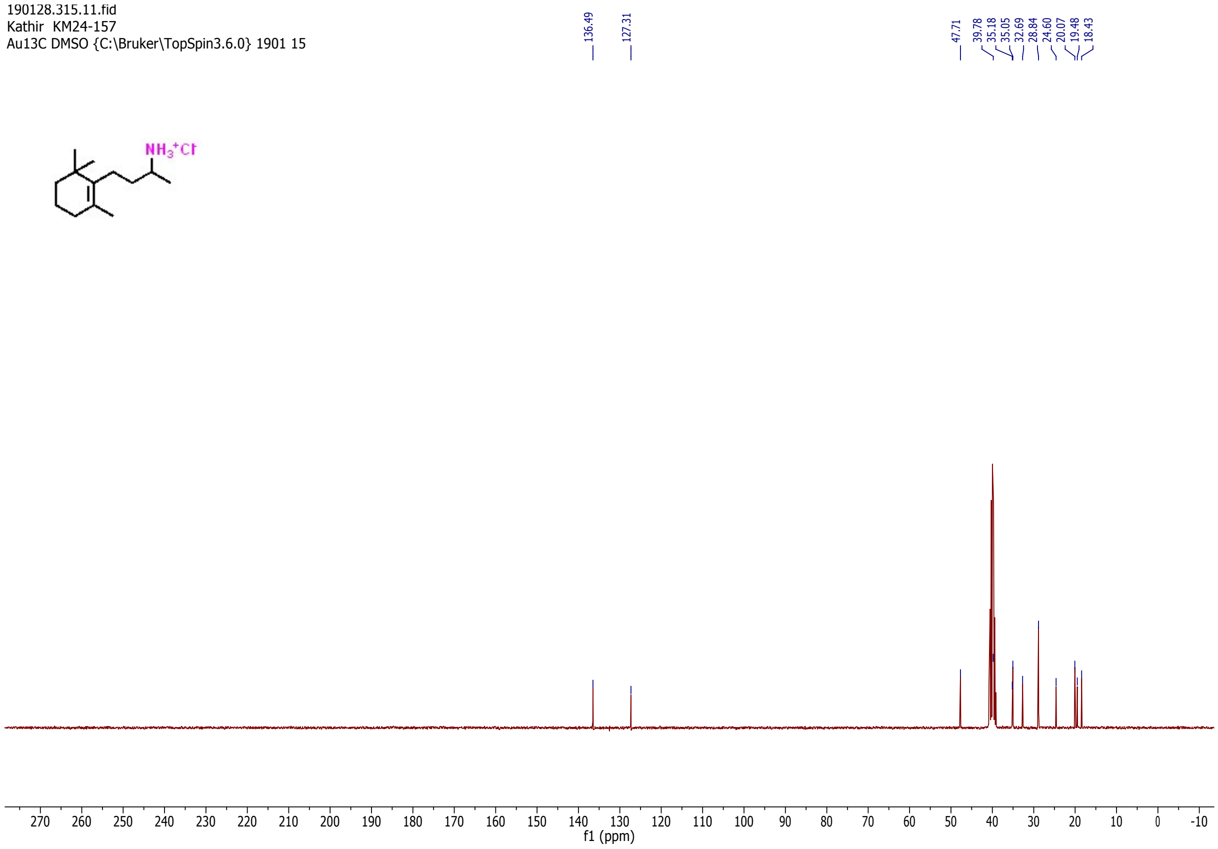

190128.f346.10.fid
Kathir KM24-170
PROTON DMSO {C:\Bruker\TopSpin3.6.0} 1901 46

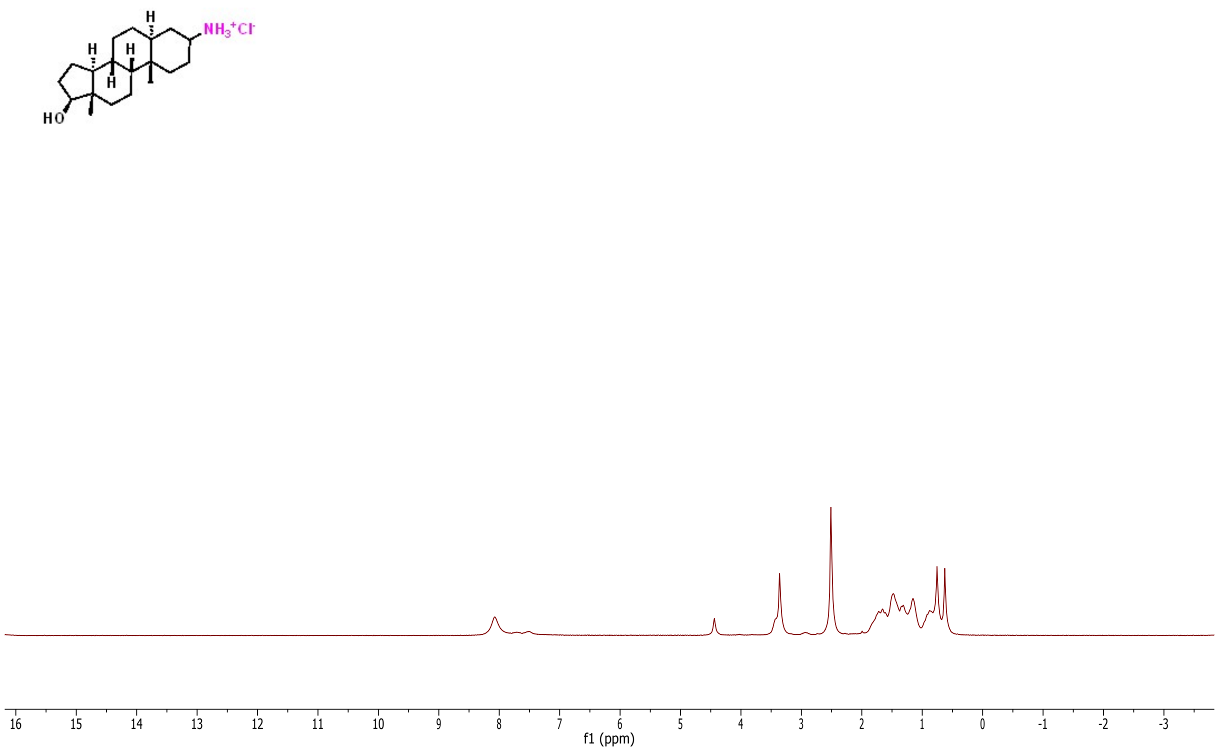

190128.f346.11.fid
Kathir KM24-170
C13CPD DMSO {C:\Bruker\TopSpin3.6.0} 1901 46

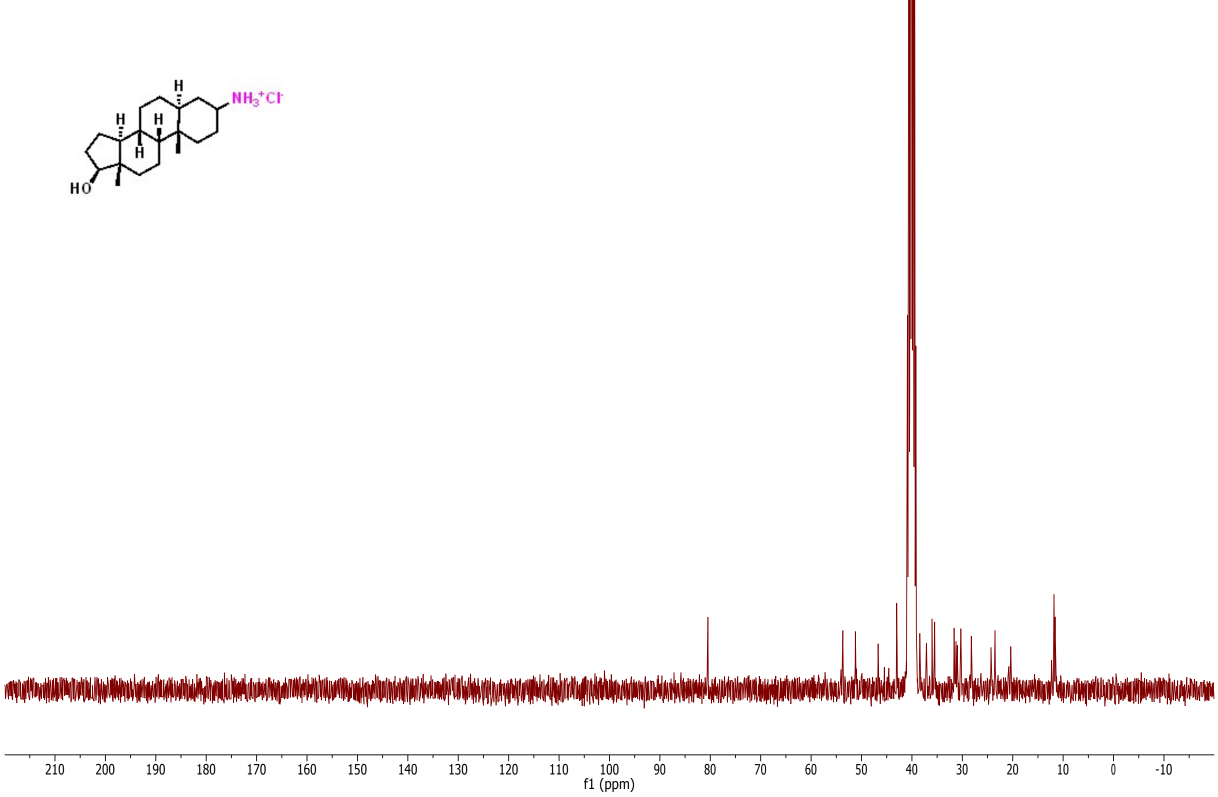

kathrin km 24 - 170 HR(EI)

File : D:\Xcalibur\data\1903\19030503hrei-av2.RAW
Full ms [277.500 - 309.500] - Range: 291.000 - 291.500
Scan No. 1 of 1

Mass	Absolute Intensity	Relative Intensity	Theoretical Mass	Delta [ppm]	Delta [mmu]	RDB	Composition
291.25493	85391	7.6	291.25567	-2.5	-0.7	4.0	$C_{19} H_{33} O_1 N_1$

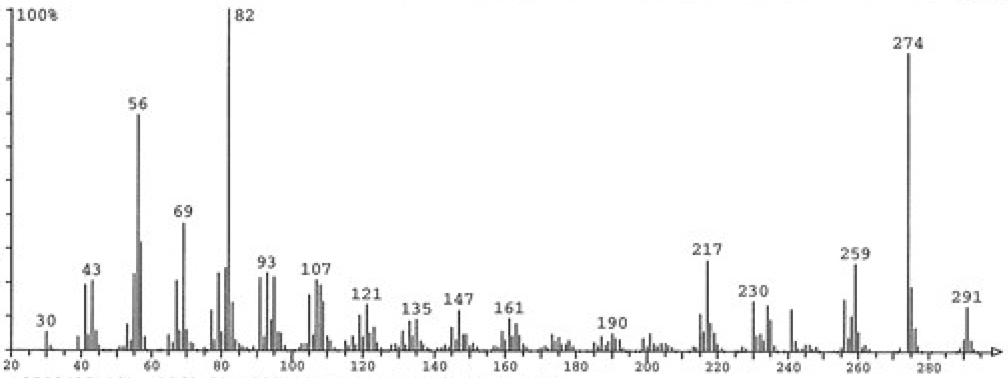

B2590(27:10) 1862 8% 19030102 * MS3-Kathir//KM4-170
19030102

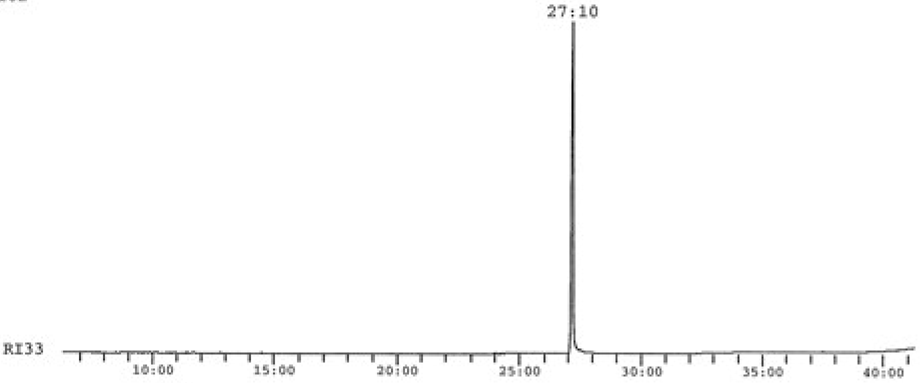

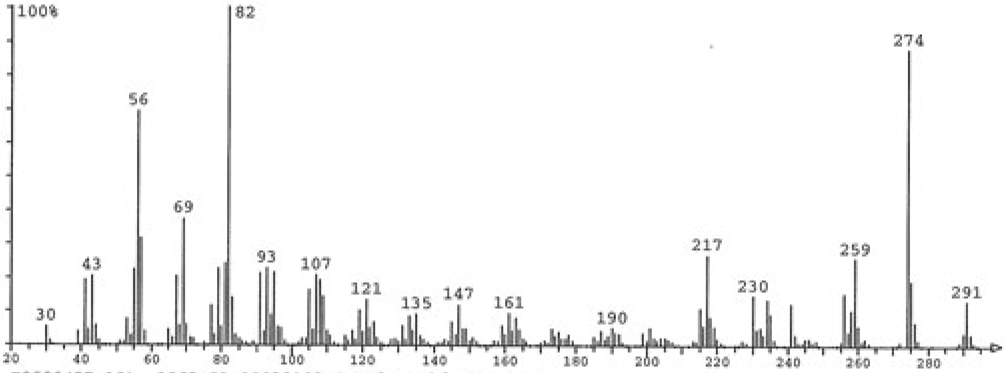

B2590(27:10) 1862 8% 19030102 * MS3-Kathir//KM4-170

30	5.20	72	1.58	105	16.23	132	1.45	163	7.63	201	4.79	235	8.91
31	1.05	77	11.61	106	4.36	133	8.46	164	4.30	202	2.09	236	1.51
39	3.64	78	2.96	107	20.56	134	4.09	165	1.62	203	1.13	241	12.08
41	19.11	79	22.65	108	18.99	135	8.83	171	1.11	204	2.01	242	2.94
42	4.23	80	5.23	109	14.20	136	2.41	173	4.60	205	1.88	245	1.69
43	20.32	81	23.88	110	3.99	137	1.43	174	2.54	206	1.52	246	1.74
44	5.52	82	100.00	111	2.67	143	1.49	175	3.81	207	0.85	248	1.03
45	1.20	83	13.78	115	2.43	144	0.81	176	1.66	213	1.14	256	14.92
53	7.50	84	2.92	116	1.06	145	6.67	177	1.59	214	0.94	257	3.62
54	2.61	85	1.75	117	3.98	146	2.77	178	2.93	215	10.80	258	10.20
55	22.25	89	0.90	118	1.37	147	11.67	179	1.05	216	5.44	259	25.51
56	69.19	91	21.21	119	10.16	148	4.64	185	2.27	217	26.48	260	5.33
57	31.60	92	3.78	120	3.61	149	4.62	186	1.00	218	7.97	261	0.81
58	3.68	93	22.46	121	13.37	150	1.04	187	3.90	219	5.18	262	1.53
65	4.32	94	8.60	122	5.01	151	2.08	188	1.40	220	1.53	274	87.50
66	2.04	95	21.26	123	6.51	157	1.02	189	2.49	227	1.17	275	18.78
67	20.19	96	5.19	124	1.90	158	0.80	190	4.80	230	14.56	276	6.52
68	5.39	97	4.80	128	1.48	159	5.53	191	3.43	231	4.25	277	1.36
69	37.21	98	1.10	129	1.67	160	2.80	192	3.20	232	4.77	290	3.56
70	5.88	103	1.57	130	0.80	161	9.27	199	3.41	233	2.92	291	13.09
71	2.04	104	1.55	131	5.57	162	3.86	200	1.22	234	13.36	292	2.99

B2590(27:10)1862 8% 19030102 * MS3-Kathir//KM4-170 lim: 0.79%

190128.f347.10.fid
Kathir KM24-171
PROTON DMSO {C:\Bruker\TopSpin3.6.0} 1901 47

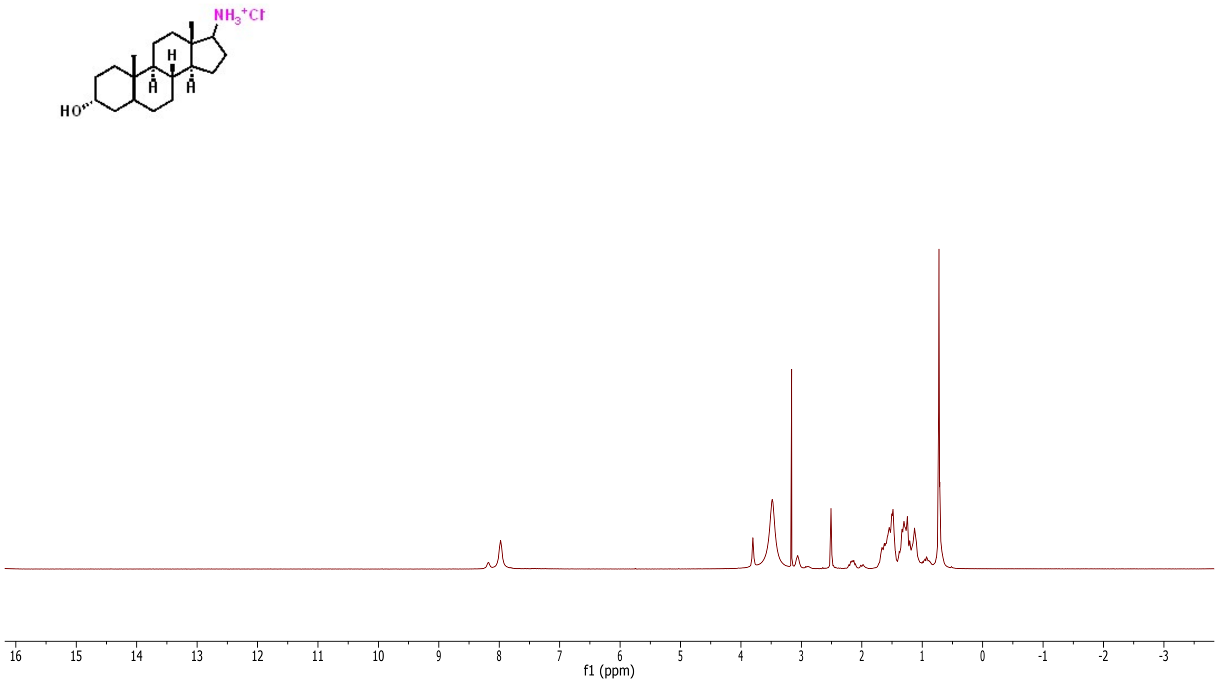

190128.f347.11.fid
Kathir KM24-171
C13CPD DMSO {C:\Bruker\TopSpin3.6.0} 1901 47

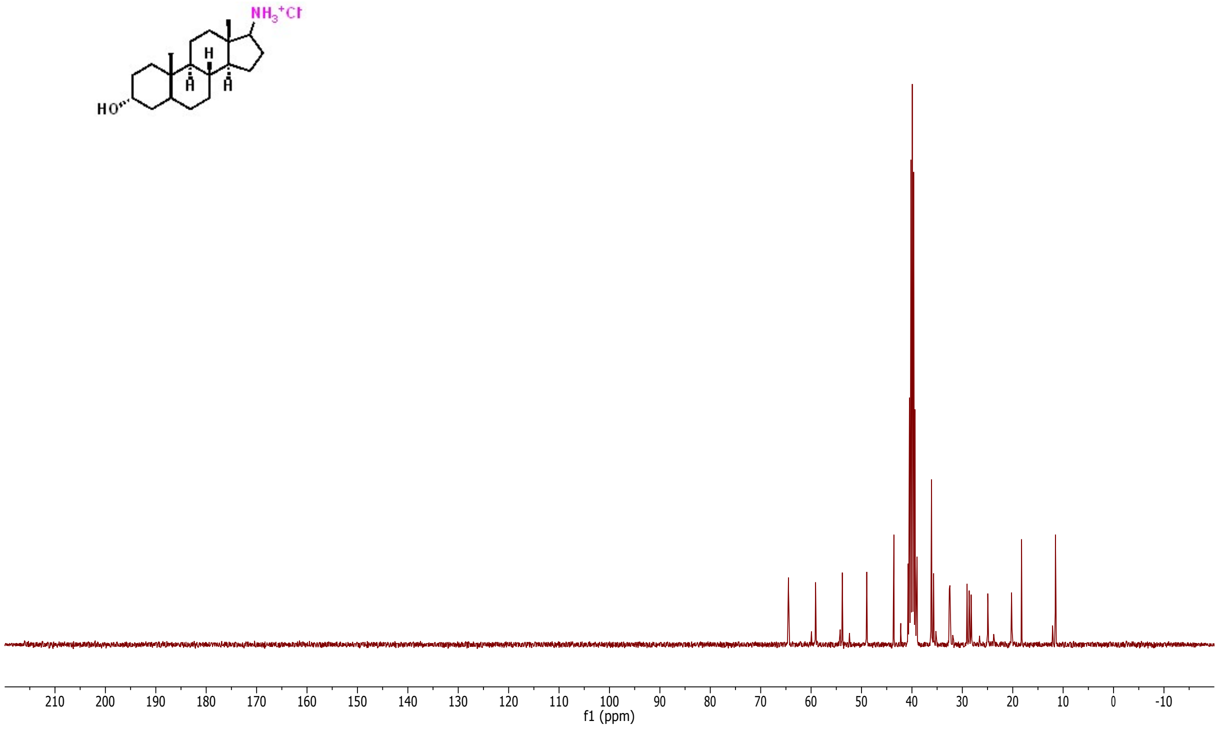

Kathrin KM 24 – 171 HR (EI)

File : D:\Xcalibur\data\1903\19030504hrei-av2.RAW
Full ms [277.500 - 309.500] - Range: 291.000 - 291.500
Scan No. 1 of 1

Mass	Absolute Intensity	Relative Intensity	Theoretical Mass	Delta [ppm]	Delta [mmu]	RDB	Composition
291.25494	670829	47.5	291.25567	-2.5	-0.7	4.0	$C_{19} H_{33} O_{1} N_{1}$

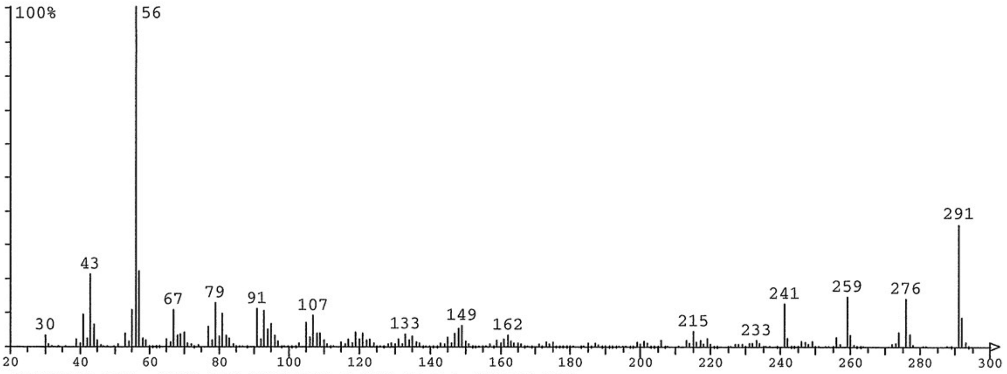

B3016(27:12) 6023 19% 19030113 * MS3-Kathir//KM24-171
19030113

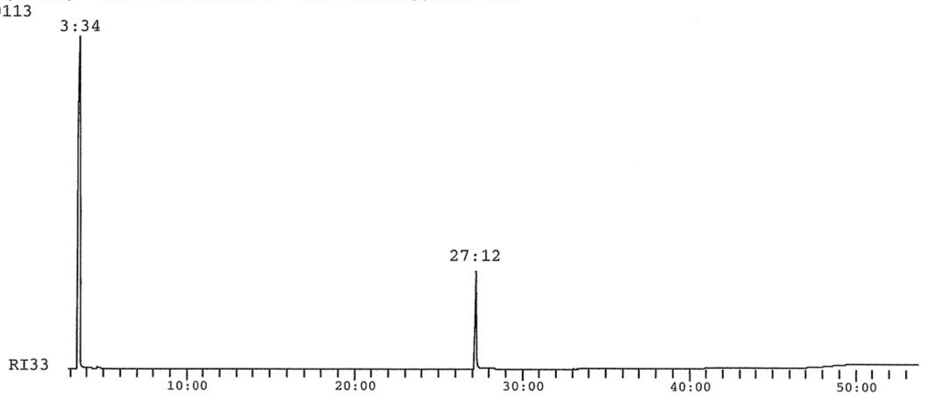

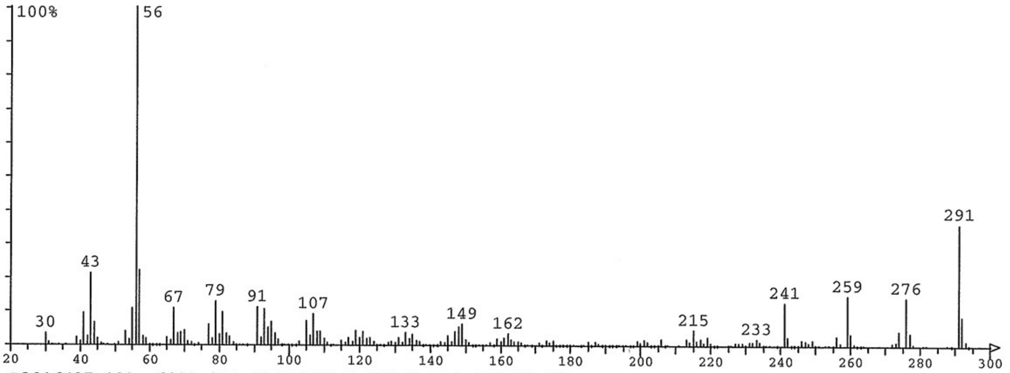

B3016(27:12) 6023 19% 19030113 * MS3-Kathir//KM24-171

30	3.11	68	3.00	97	1.33	129	0.89	160	0.87	202	0.74	243	0.31
31	0.52	69	3.41	98	0.32	130	0.38	161	2.00	203	0.30	246	1.30
39	2.05	70	4.08	103	0.80	131	2.06	162	3.00	206	1.58	247	1.01
40	0.82	71	0.76	105	6.87	132	0.65	163	1.25	207	0.34	248	0.65
41	9.14	72	0.42	106	2.44	133	3.38	164	0.69	213	1.64	249	1.41
42	2.24	74	0.37	107	8.82	134	1.54	165	0.84	214	0.69	250	0.32
43	21.13	77	5.83	108	3.58	135	2.92	166	0.61	215	4.23	256	2.50
44	6.22	78	1.59	109	3.65	136	1.17	171	0.48	216	1.06	257	0.65
45	1.77	79	12.60	110	1.59	137	0.68	173	1.17	217	1.60	259	14.37
46	0.36	80	2.84	111	0.65	143	0.67	174	0.63	218	0.50	260	3.04
51	0.45	81	9.64	115	1.06	144	0.38	175	0.97	219	2.28	261	0.36
53	3.65	82	3.22	116	0.49	145	2.67	176	0.35	220	0.44	272	0.45
54	1.27	83	2.24	117	2.02	146	0.83	185	0.84	227	0.43	273	0.67
55	10.67	84	0.58	118	0.76	147	3.69	186	0.25	228	0.45	274	3.94
56	100.00	88	0.29	119	3.99	148	5.29	187	0.75	229	0.41	276	13.74
57	21.85	91	11.02	120	1.93	149	5.96	188	0.36	231	0.81	277	3.56
58	2.19	92	2.06	121	3.80	150	1.41	189	0.35	232	0.74	278	0.37
59	1.61	93	10.39	122	1.67	151	0.47	192	0.27	233	1.69	289	0.34
65	2.00	94	4.94	123	2.09	157	0.51	199	1.07	234	0.91	291	35.76
66	1.10	95	6.69	124	0.89	158	0.30	200	0.41	241	12.38	292	8.24
67	10.76	96	3.00	128	0.56	159	1.72	201	1.44	242	2.35	293	0.96

B3016(27:12)6023 19% 19030113 * MS3-Kathir//KM24-171 lim: 0.25%

190227.f305.10.fid
Kathir KM24-284
PROTON CDCl3 {C:\Bruker\TopSpin3.6.0} 1902 5

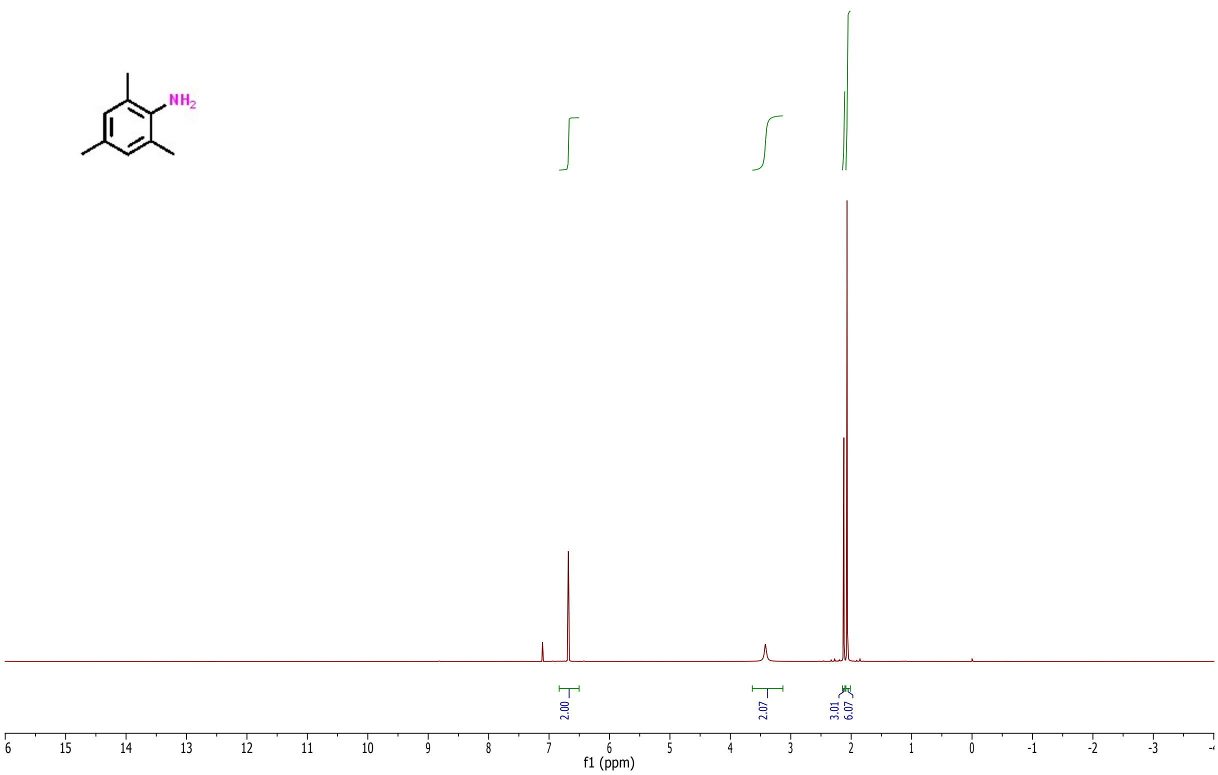

190227.f305.11.fid
Kathir KM24-284
C13CPD CDCl3 {C:\Bruker\TopSpin3.6.0} 1902 5

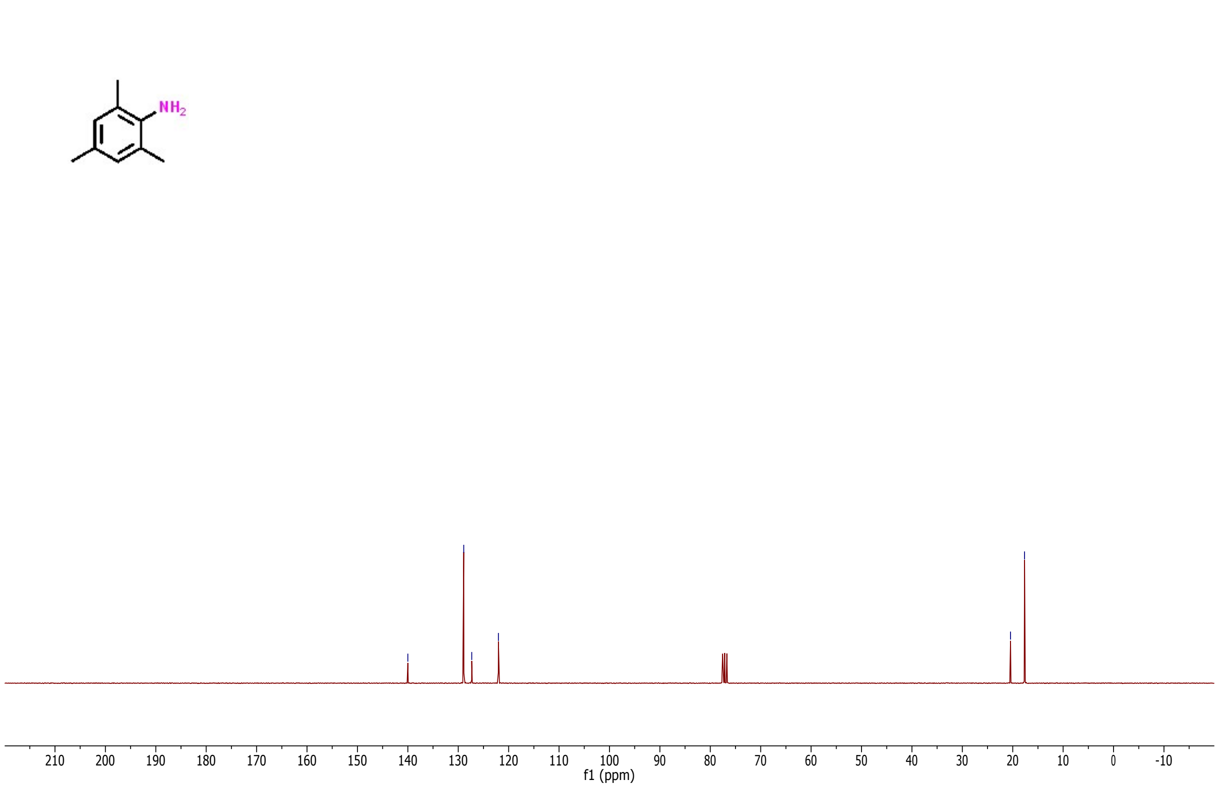

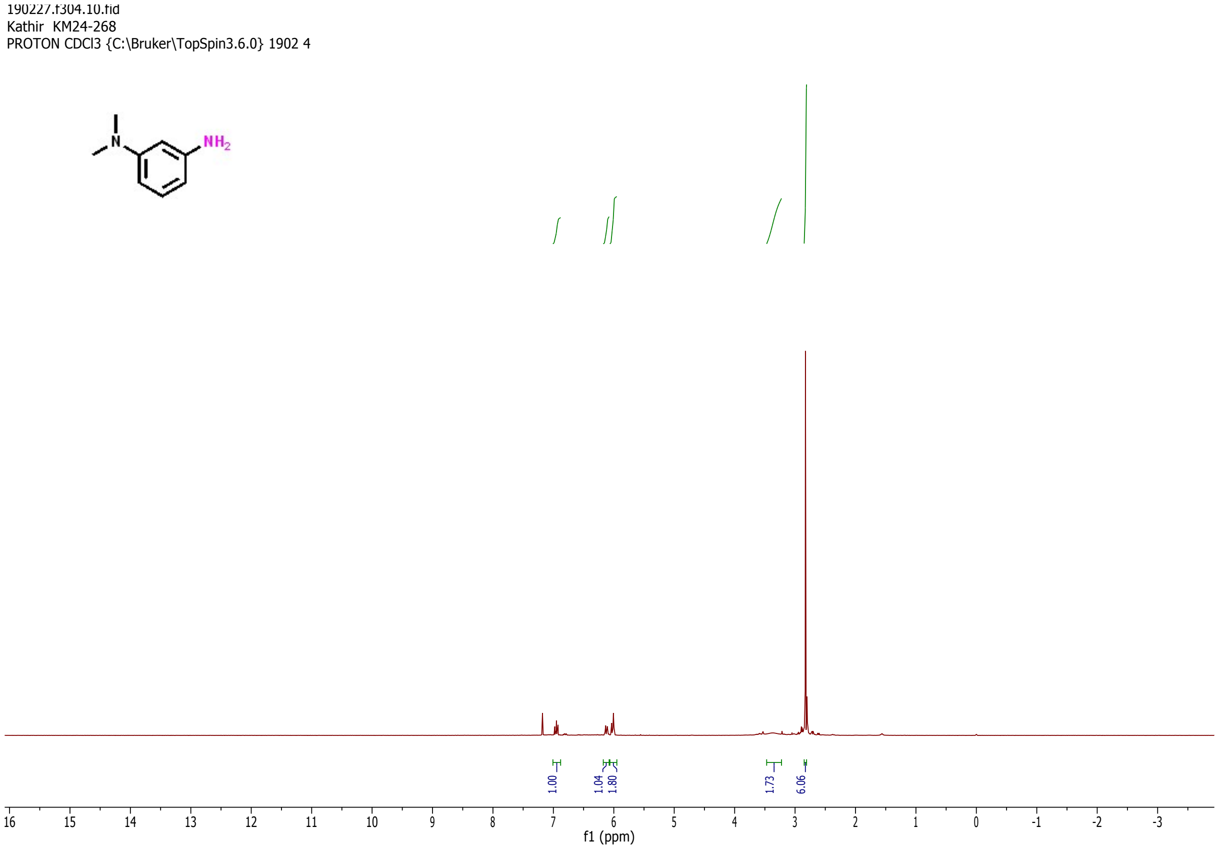
190227.f304.10.fid
Kathir KM24-268
PROTON CDCl3 {C:\Bruker\TopSpin3.6.0} 1902 4
NH2
1.00
1.04
1.80
1.73
6.06
f1 (ppm)

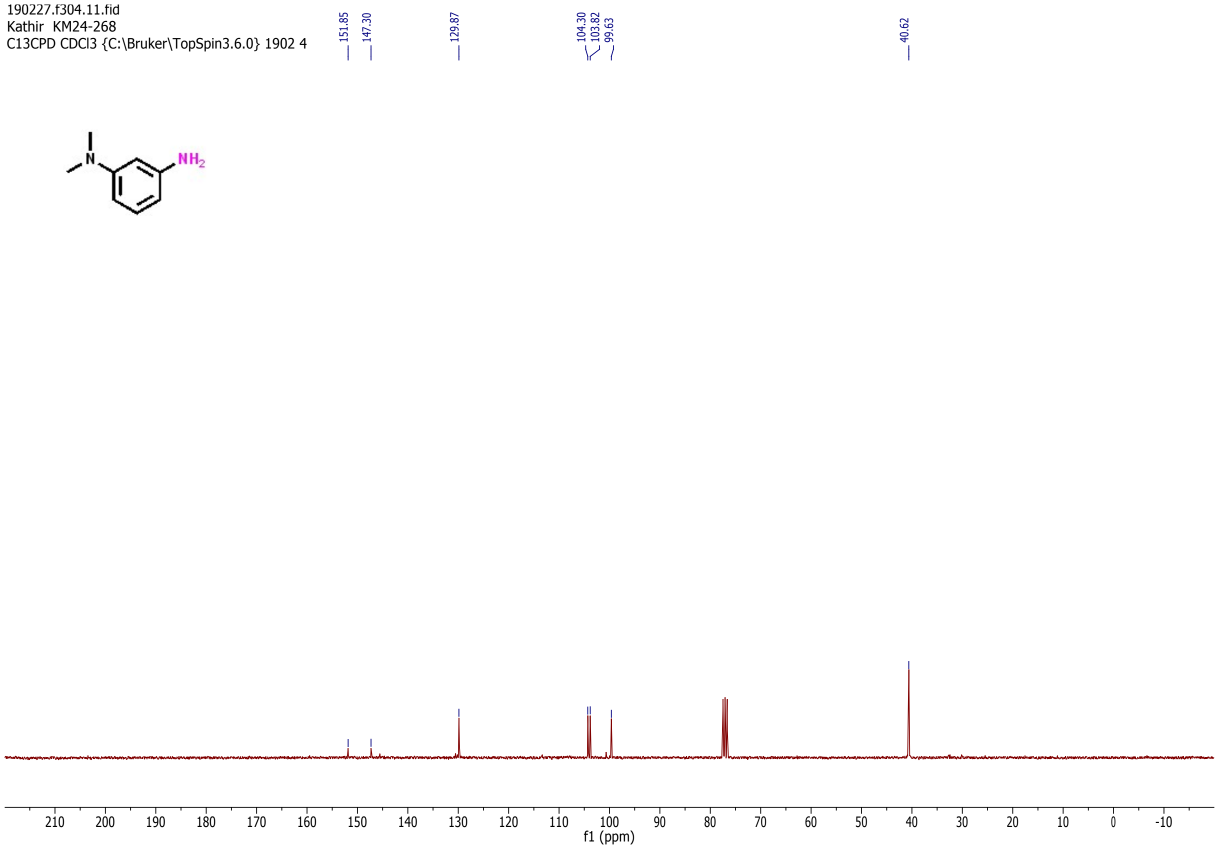
190227.f304.11.fid
Kathir KM24-268
C13CPD CDCl3 {C:\Bruker\TopSpin3.6.0} 1902 4
151.85
147.30
129.87
104.30
103.82
99.63
40.62
NH2
f1 (ppm)

190227.f306.10.fid
Kathir KM24-94
PROTON DMSO {C:\Bruker\TopSpin3.6.0} 1902 6

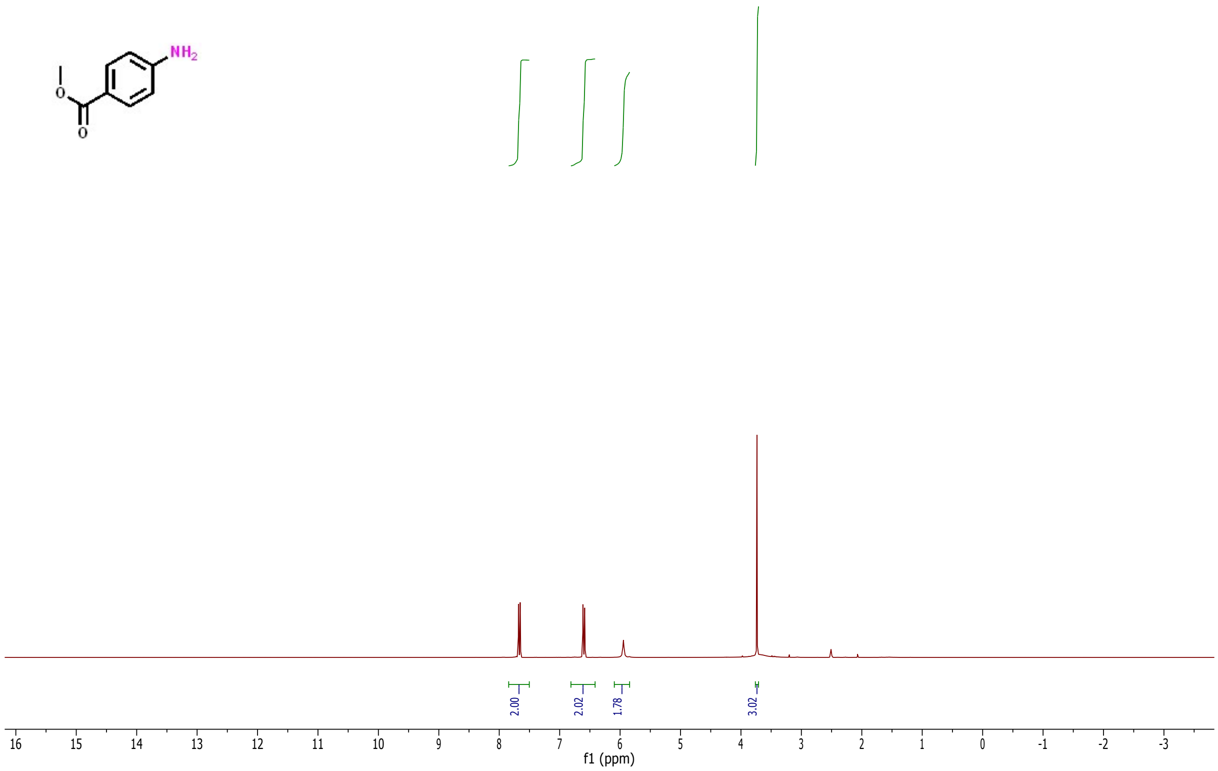

190227.f306.11.fid
Kathir KM24-94
C13CPD DMSO {C:\Bruker\TopSpin3.6.0} 1902 6

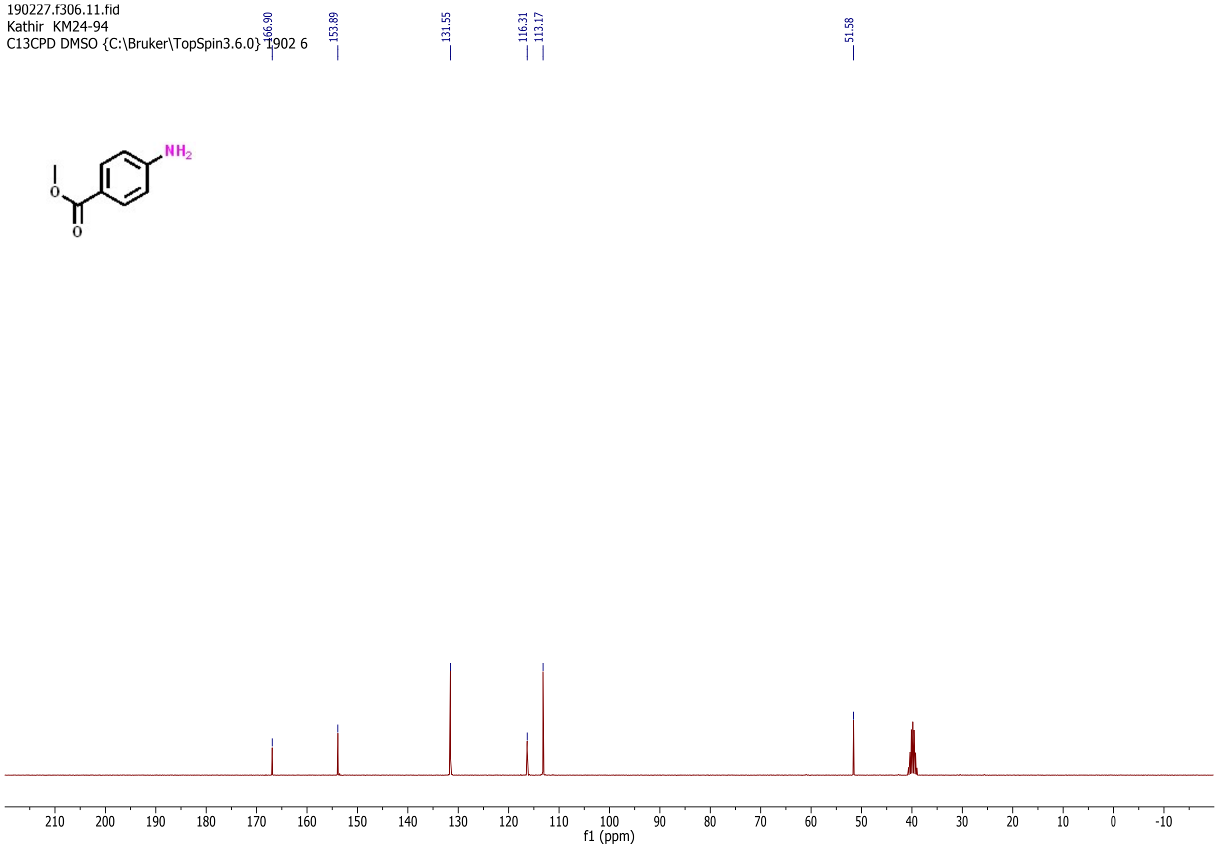

190227.f307.10.fid
Kathir KM24-239
PROTON DMSO {C:\Bruker\TopSpin3.6.0} 1902 7

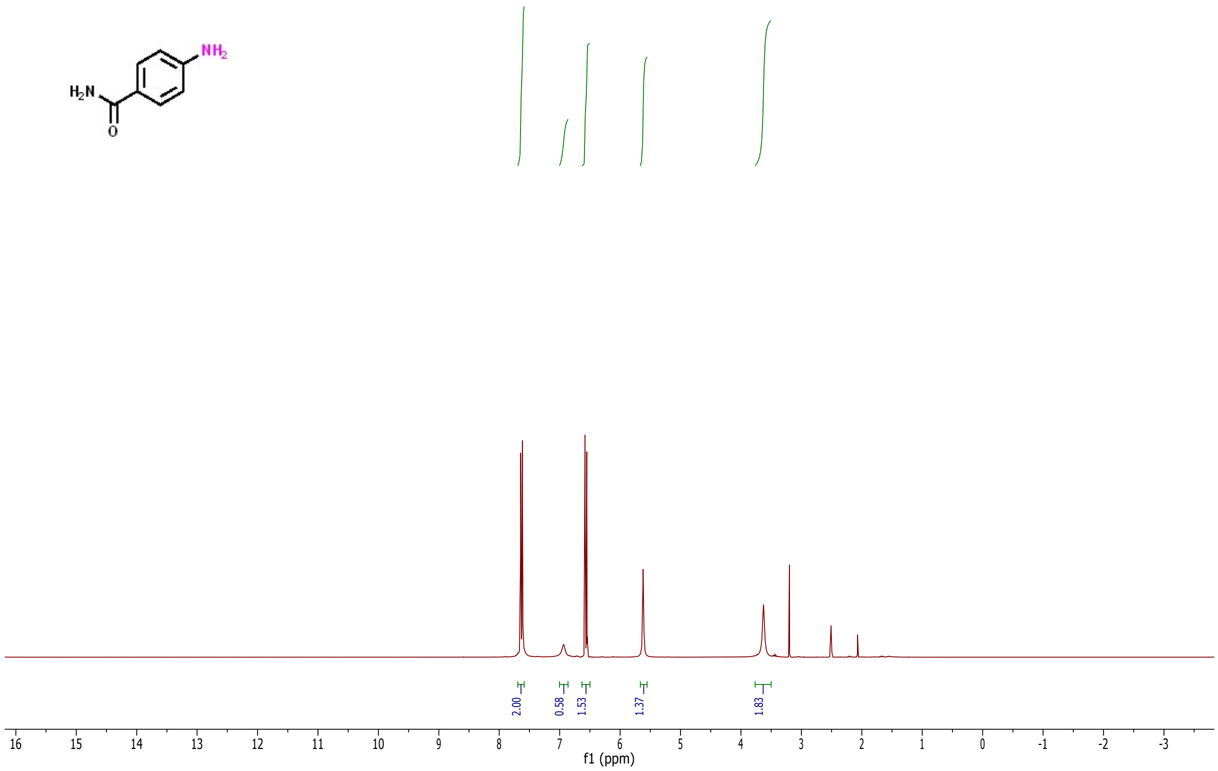

190227.f307.11.fid
Kathir KM24-239
C13CPD DMSO {C:\Bruker\TopSpin3.6.0} 1902 7

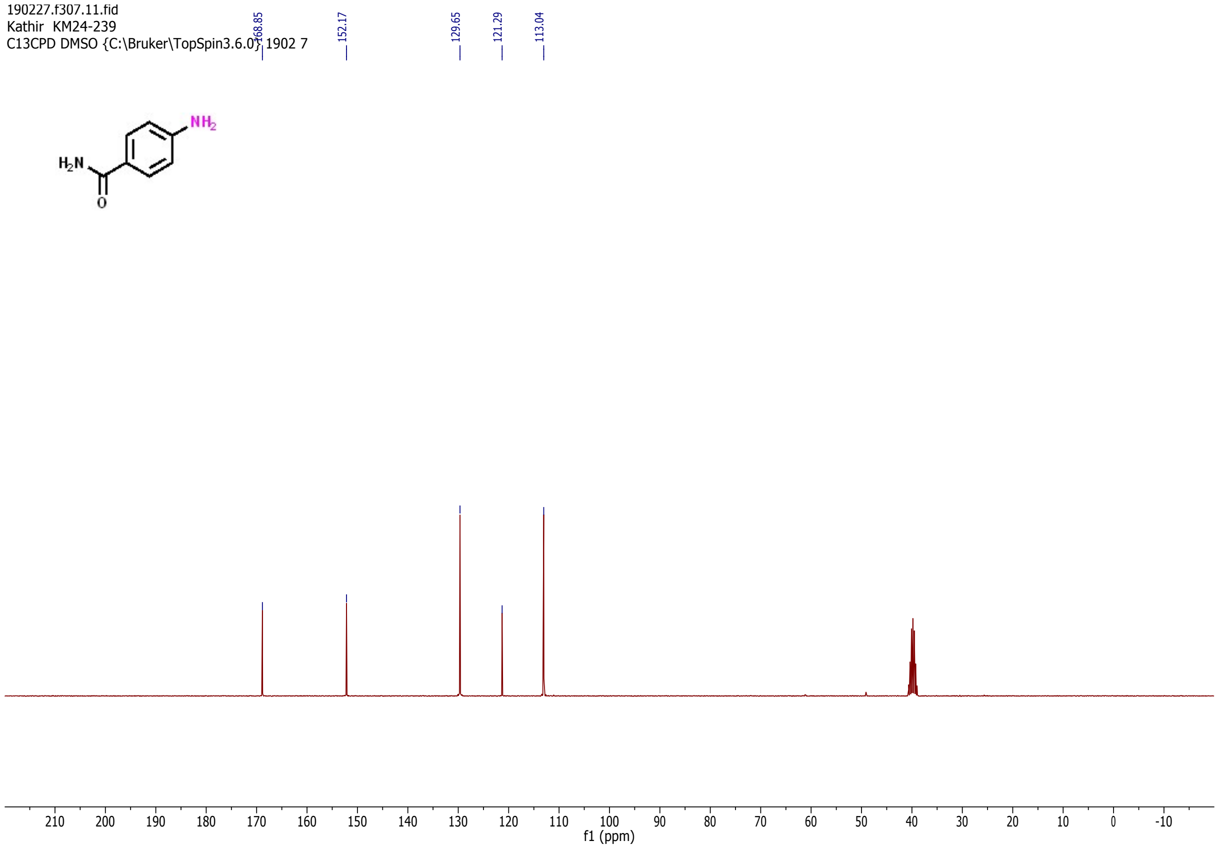

190227.f302.10.fid
Kathir KM24-237
PROTON CDCl3 {C:\Bruker\TopSpin3.6.0} 1902 2

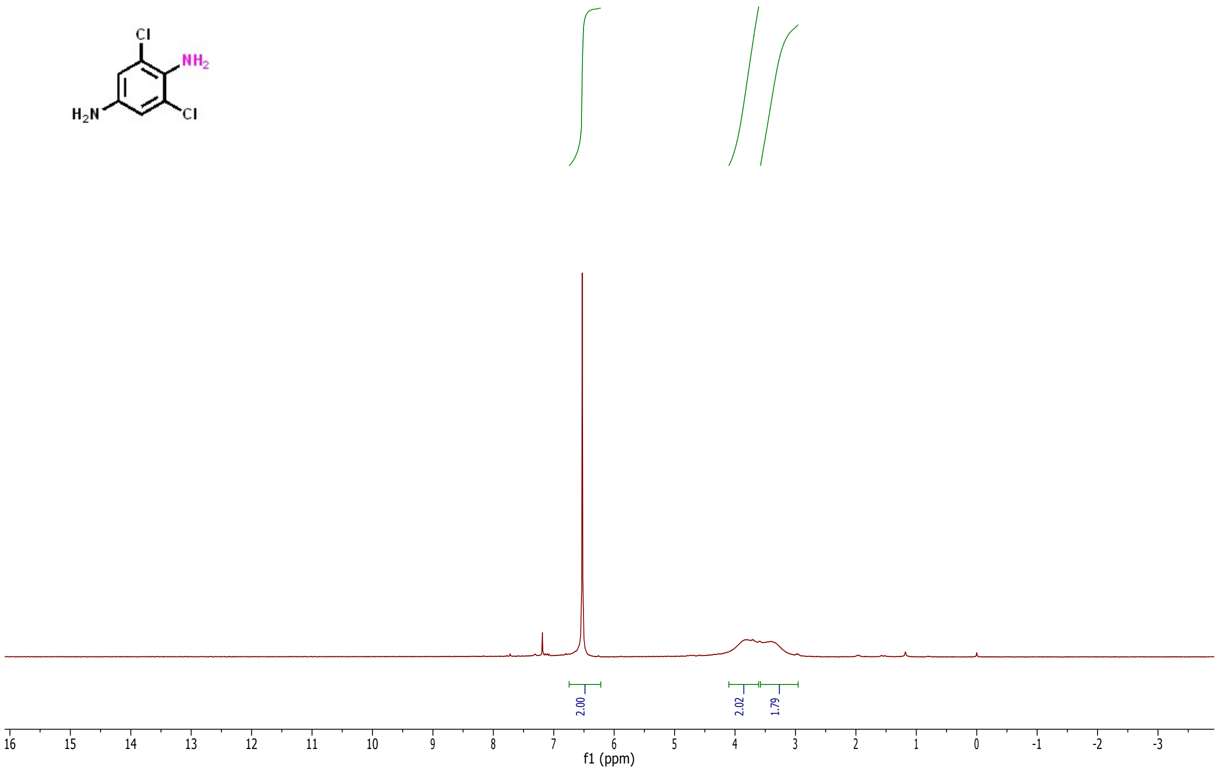

190227.f302.11.fid
Kathir KM24-237
C13CPD CDCl3 {C:\Bruker\TopSpin3.6.0} 1902 2

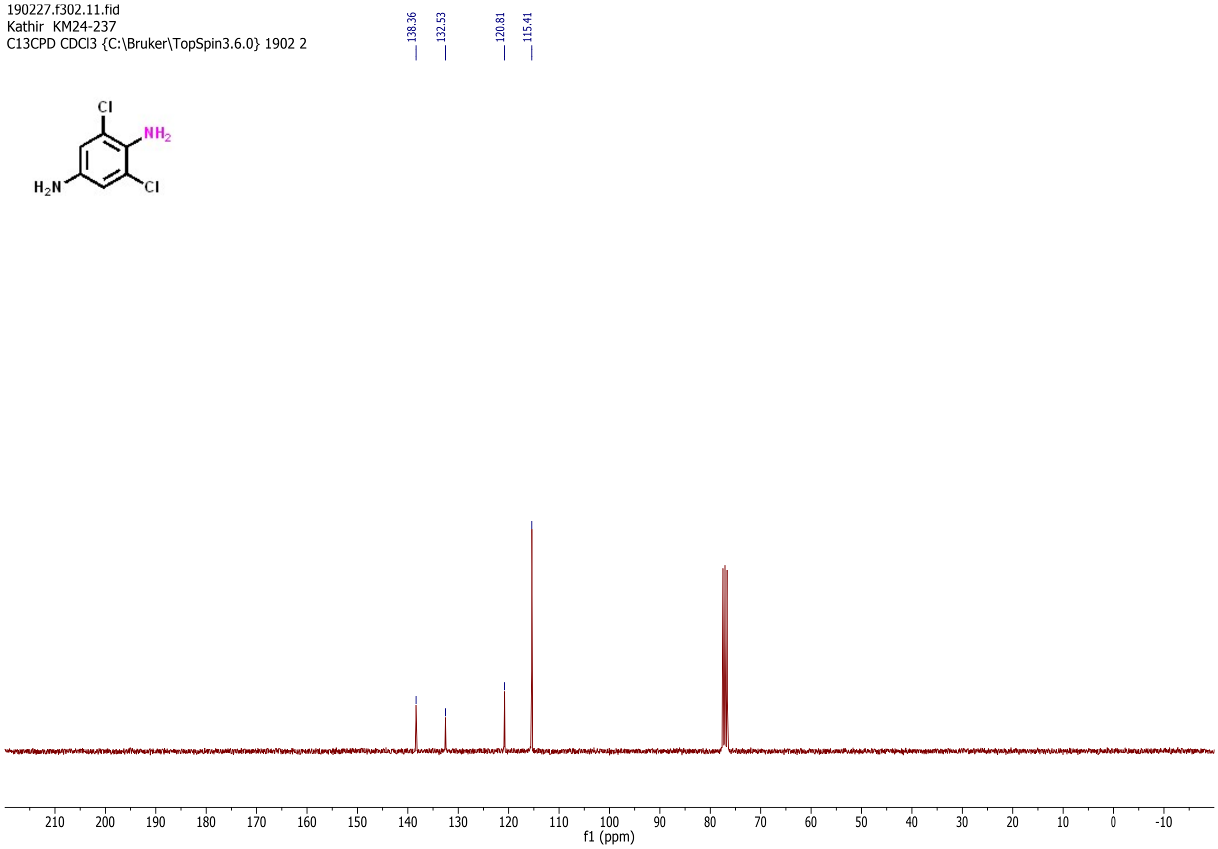

190301.f343.10.fid
Kathir KM24-227
PROTON DMSO {C:\Bruker\TopSpin3.6.0} 1903 43

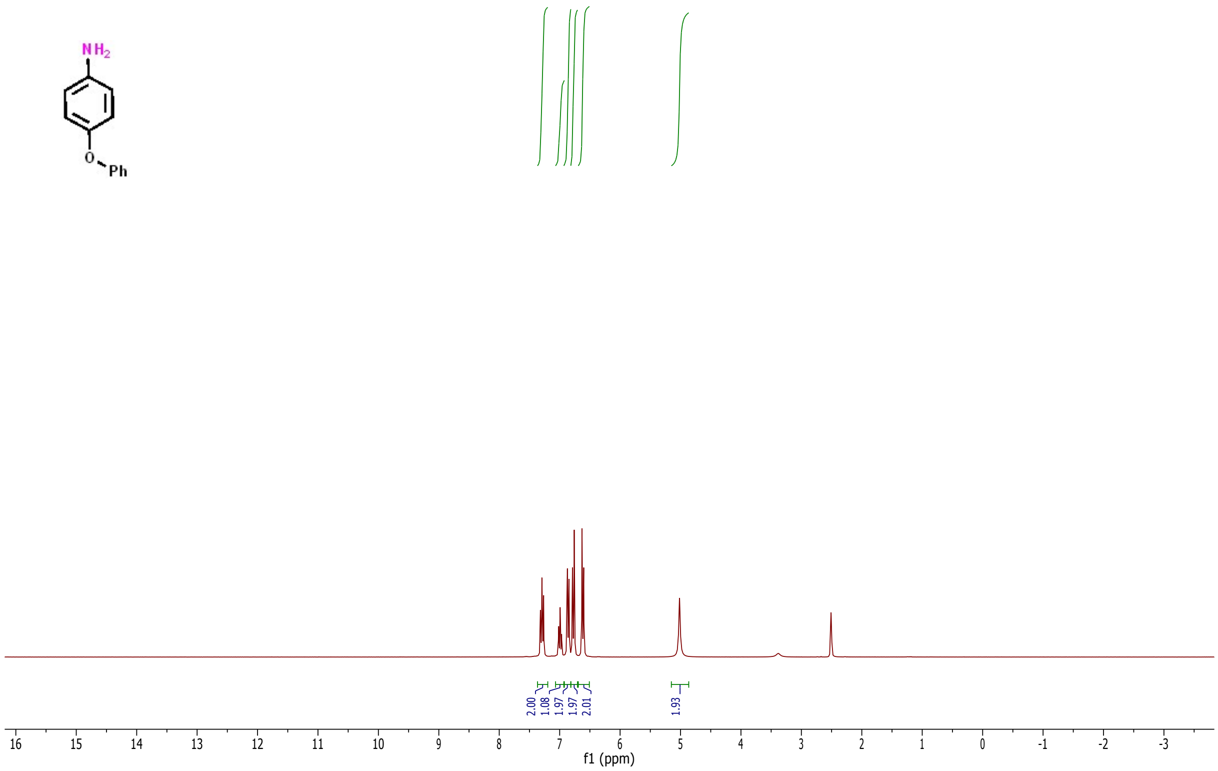

190301.f343.11.fid
Kathir KM24-227
C13CPD DMSO {C:\Bruker\TopSpin3.6.0} 1903 43

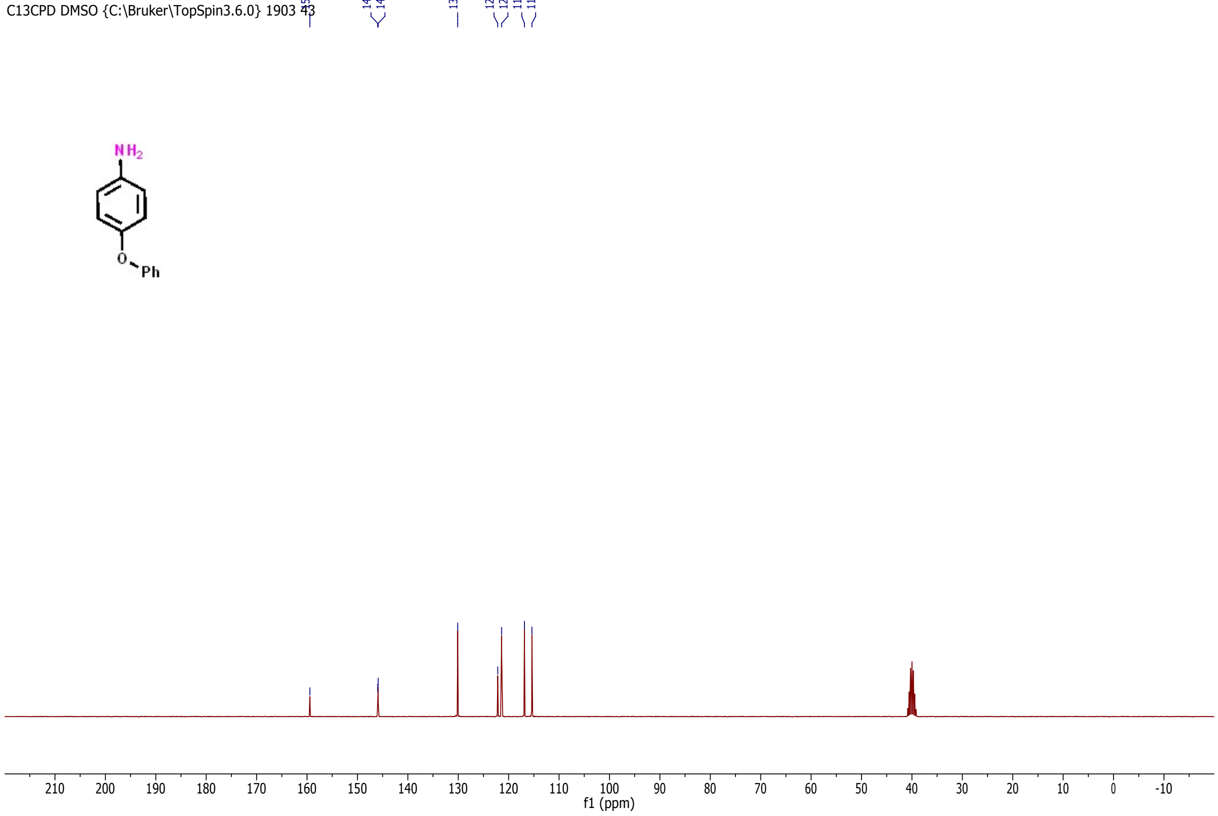

190227.f303.10.fid
Kathir KM24-266
PROTON CDCl3 {C:\Bruker\TopSpin3.6.0} 1902 3

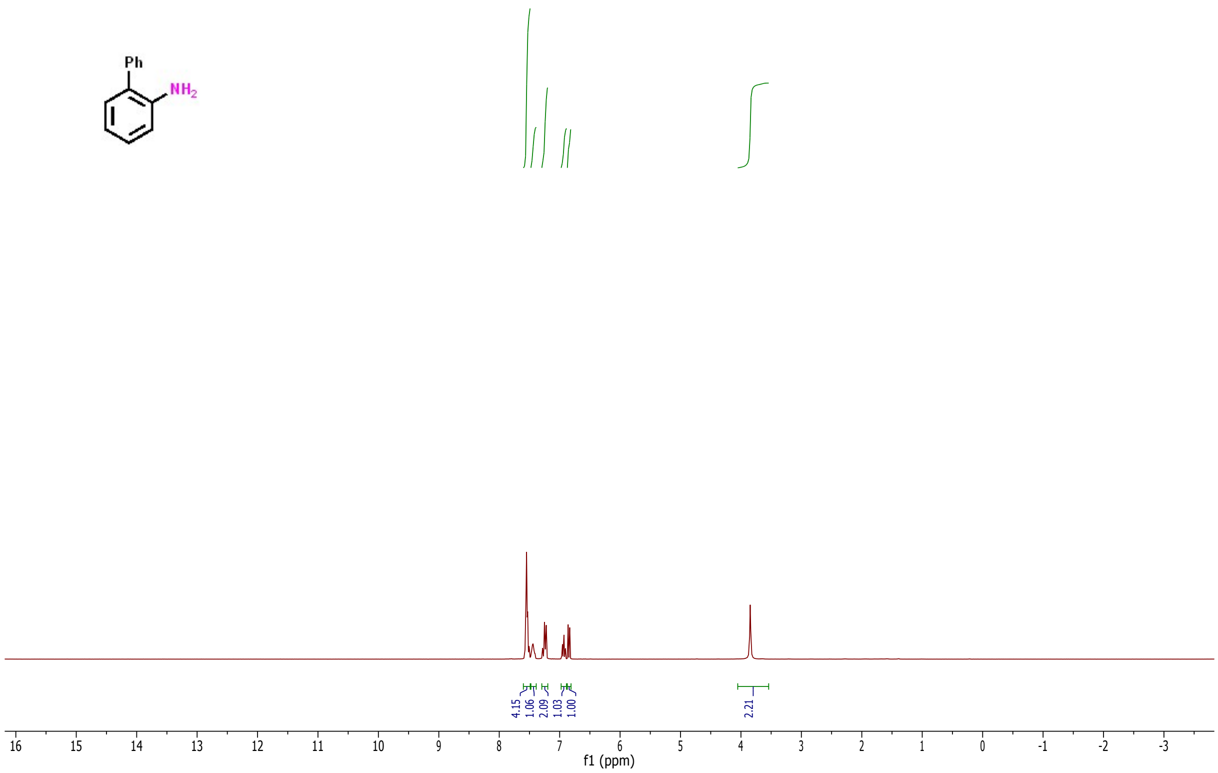

190227.f303.11.fid
Kathir KM24-266
C13CPD CDCl3 {C:\Bruker\TopSpin3.6.0} 1902 3

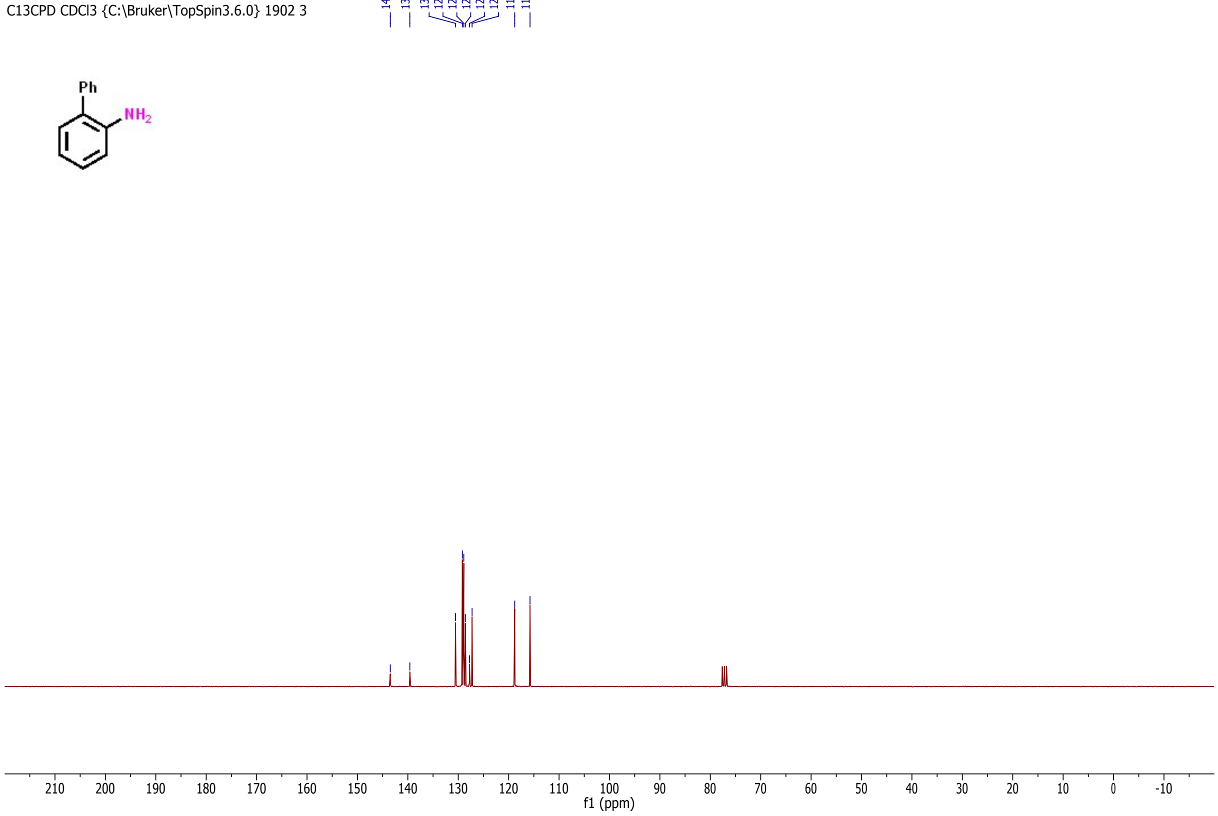

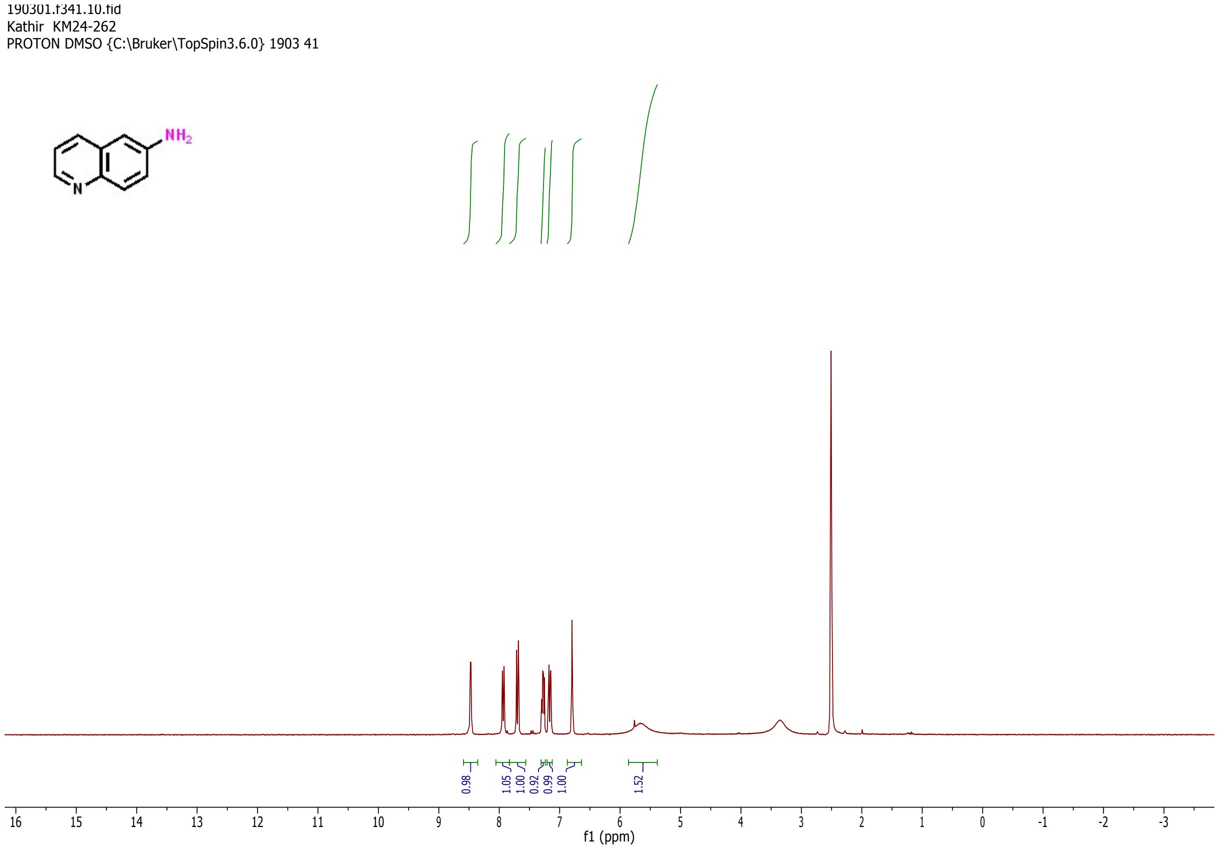
190301.f341.10.fid
Kathir KM24-262
PROTON DMSO {C:\Bruker\TopSpin3.6.0} 1903 41
NH2
N
0.98
1.05
1.00
0.92
0.99
1.00
1.52
16 15 14 13 12 11 10 9 8 7 6 5 4 3 2 1 0 -1 -2 -3
f1 (ppm)

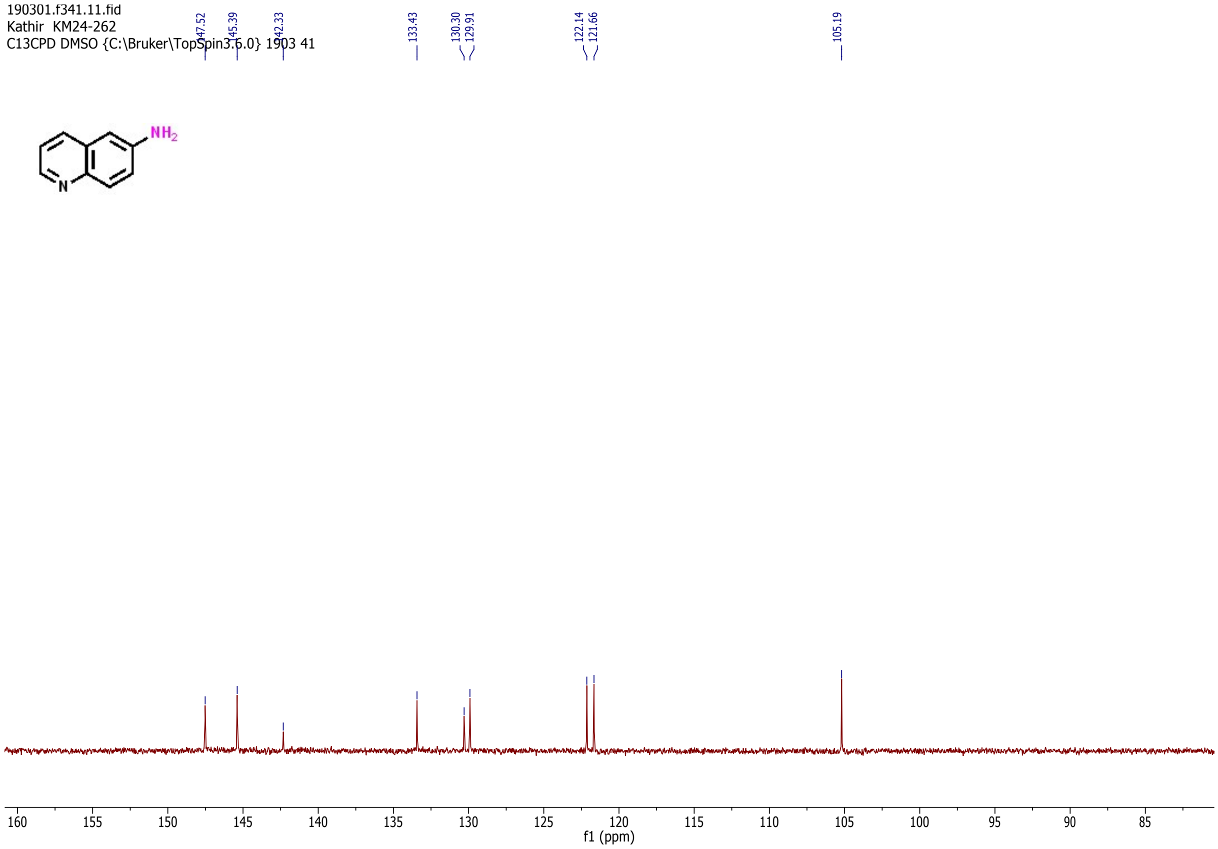
190301.f341.11.fid
Kathir KM24-262
C13CPD DMSO {C:\Bruker\TopSpin3.6.0} 1903 41
147.52
145.39
142.33
133.43
130.30
129.91
122.14
121.66
105.19
NH2
N
160 155 150 145 140 135 130 125 120 115 110 105 100 95 90 85
f1 (ppm)

190301.f342.10.fid
Kathir KM24-264
PROTON DMSO {C:\Bruker\TopSpin3.6.0} 1903 42

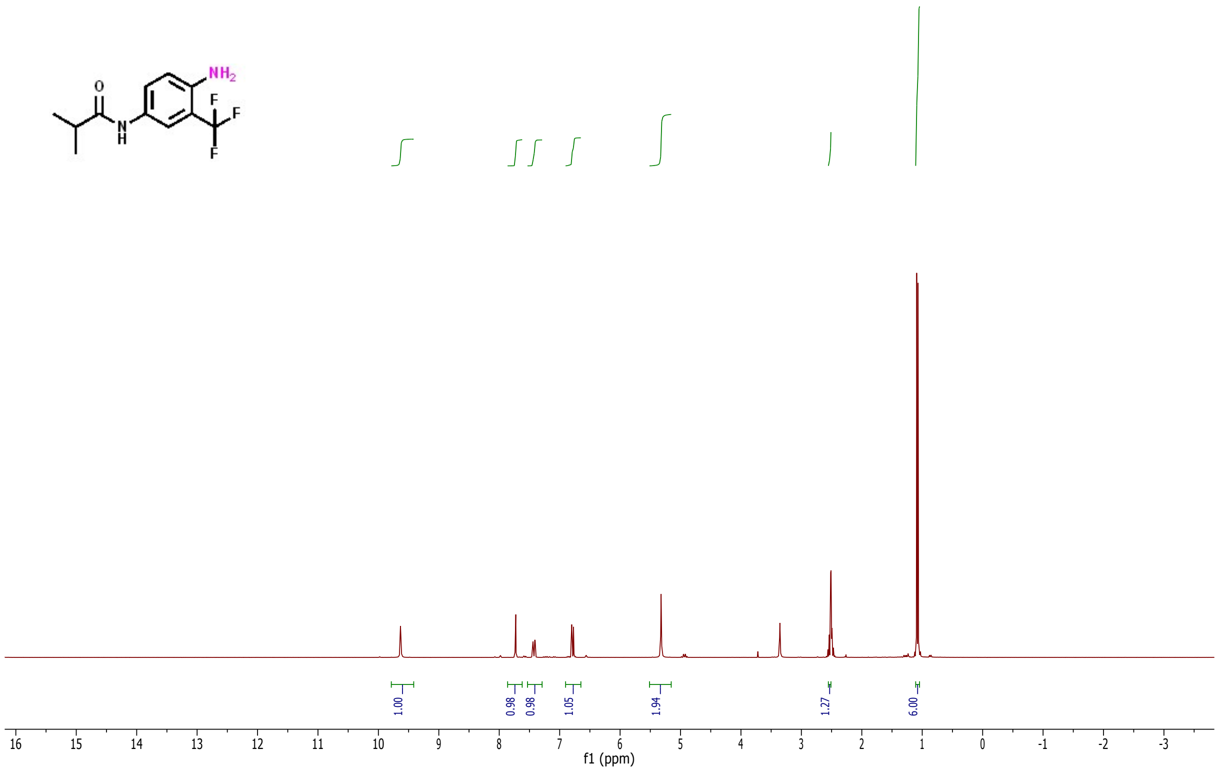

190301.f342.11.fid
Kathir KM24-264
C13CPD DMSO {C:\Bruker\TopSpin3.6.0} 1903 42

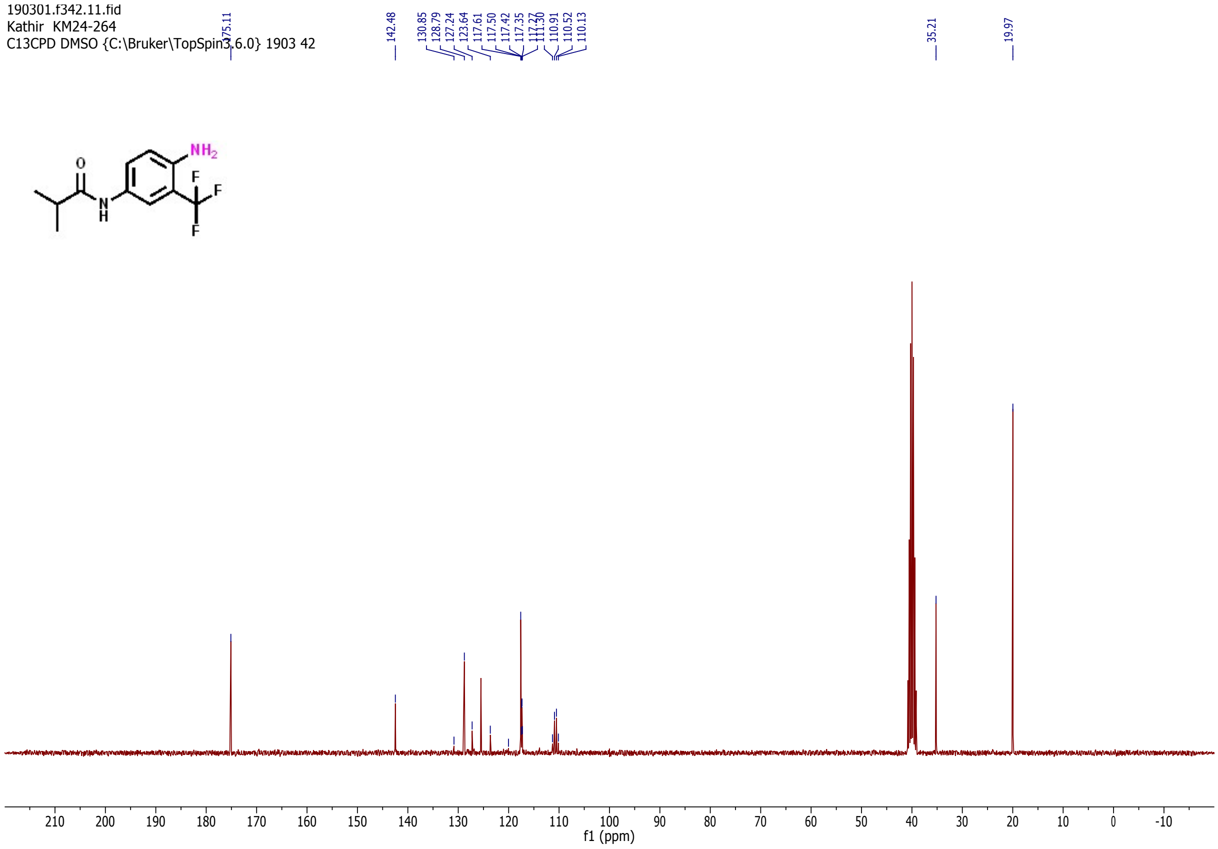

Supplement: SC-011-D0SC01084G-s001 [file SC-011-D0SC01084G-s001.pdf]
